# China trauma treatment statistics 2019: A national retrospective study based on hospitalized cases

**DOI:** 10.3389/fpubh.2023.1116828

**Published:** 2023-02-24

**Authors:** Yanhua Wang, Chu Wang, Pan Hu, Haibo Wang, Lanxia Gan, Guilan Kong, Ying Shi, Tianbing Wang, Baoguo Jiang

**Affiliations:** ^1^Department of Trauma and Orthopedics, Peking University People's Hospital, Beijing, China; ^2^Trauma Medicine Center, Peking University People's Hospital, Beijing, China; ^3^Clinical Trial Unit, First Affiliated Hospital of Sun Yat-sen University, Guangzhou, China; ^4^China Standard Medical Information Research Center, Shenzhen, China; ^5^National Research Institute of Big Data for Health and Medical Care, Peking University, Beijing, China

**Keywords:** trauma treatment, trauma, database, epidemiology, China, hospitalized

## Abstract

**Objective:**

Trauma is China's fifth leading cause of death and ranked first among youths. Trauma databases have been well-established in many countries to announce the current state of trauma rescue, treatment and care. Nevertheless, China hasn't yet established a comparable database. This paper included two national-level databases in China to describe the current situation of trauma treatment and the epidemiological characteristics of trauma incidence, which sought to provide data support for decision-making, resource allocation, trauma prevention, trauma management, and other aspects.

**Methods:**

This study used the diagnosis and treatment data from the Hospital Quality Monitoring System (HQMS) and the China Trauma Rescue and Treatment Association (CTRTA) in 2019. A descriptive analysis was conducted to explore the demographic characteristics, trauma causes, injury degrees of trauma patients, disease burden and mortality rates in the abstracted hospitalized cases.

**Results:**

A total of 4,532,029 trauma patients were included, of which 4,436,653 were from HQMS and 95,376 from CTRTA respectively. The age group with the highest proportion is 50-54 years old (493,320 [11.12%] in HQMS and 12,025 [12.61%] in CTRTA). Fall was the most frequent cause of trauma hospitalization, accounting for 40.51% of all cases, followed by traffic injuries, accounting for 25.22%. However, for trauma patients aged between 20 and 24 years old, the most common cause of injury was traffic accidents (28.20%). Hospital expenses for trauma patients in 2019 exceeded 100.30 billion yuan, which increases significantly with age, and fall costs the most. The mortality rate of trauma inpatients was 0.77%, which gradually increased with age after 30-year-old, and was the highest in the age group above 85 (1.86%).

**Conclusion:**

This paper summarizes the demographic characteristics, trauma causes distribution, disease burden, mortality rate, and other relative data of inpatients in 2019, which can now be used as an up-to-date clinical evidence base for national healthcare prevention and management in China.

## 1. Introduction

Trauma is one of the leading causes of death in the world (1). For the past 5 years in China, trauma has become the fifth cause of death for Chinese citizens and the first for Chinese youths (2). Previous studies have shown that the Chinese proportion of trauma-caused disability-adjusted life years (DALY) has witnessed a significantly increasing trend in recent years, resulting in a heavy burden to both society and families (3). The American College of Surgeons (ACS) established the National Trauma Data Bank (NTDB) as early as 2004 and issued annual reports on a regular basis (4). Trauma data banks have also been built in Australia, New Zealand, Canada, and other countries in order to inform the medical circle, the public, and decision-makers of the status quo of trauma rescue, treatment and care, and further provide guidance and evaluation on epidemiology, research education, resources allocation, and other aspects (5–7). Unlike Europe, the USA, and other developed countries in the world, China, as the world's largest developing country, has its own trauma incidence characteristics. Besides, China accounts for approximately one-fifth of the world's population (8), and understanding the epidemiological characteristics of trauma in China will contribute to the world's trauma rescue and treatment. As China has a vast territory, the regions in the country differ in geographical conditions, economic development, medical and health security, culture and customs, etc. These regions may differ in the epidemiological characteristics of trauma, such as incidence, severity, and other aspects (9–11). Thus, it is particularly important to conduct a survey and research on the nationwide trauma rescue and treatment in China.

The National Center for Trauma Medicine (NCTM) conducted a retrospective study of 4,532,029 trauma inpatients in 2019 using two data resources: (1) the front-page data of hospital medical records collected from 1,879 public tertiary hospitals nationwide by the Hospital Quality Monitoring System (HQMS) of the National Health Commission of the People's Republic of China (NHC); (2) hospital medical records data from 339 hospitals recorded by the China Trauma Rescue and Treatment Association (CTRTA). NCTM analyzed and summarized data on the epidemiological and clinical characteristics, treatment methods, and disease burden in relation to trauma in China. This study aims to comprehensively understand and evaluate the current situation of trauma treatment and the epidemiological characteristics of trauma incidence, which seeks to provide data support for decision-making, resource allocation, trauma prevention, trauma management, and other aspects.

## 2. Methodology

### 2.1. Data source

Two data sources, namely the HQMS of the NHC and the big data platform of the CTRTA, were adopted in this annual report.

The HQMS database is a mandatory national database system for inpatients authorized by NHC. All tertiary hospitals in China are required to submit standardized discharge records of inpatients to HQMS. The front page of each hospital medical record is completed by the patient's physician in charge. It is coded by professional medical coders based on the International Statistical Classification of Diseases and Related Health Problems, 10th revision (ICD-10) for ensuring data integrity, accuracy and consistency. HQMS data provides each patient's clinical diagnosis, surgery or operation, and external causes of poisoning, all coded by ICD-10, and other information such as demographic data, medical expenses, duration of hospitalization, injuries, etc.

The big data platform of CTRTA is a trauma data platform that contains a total of 339 hospitals, of which 197 are tertiary hospitals, 135 are secondary hospitals and 7 are primary hospitals. It mainly involves outpatient and emergency patients, and records more trauma-related information, such as Trauma Index (TI), Glasgow Coma Scale (GCS), than the HQMS database, designed to compensate for the inefficient rescue and treatment in the Chinese medical system. Sorting out and analyzing such more professional information could help us accurately understand the status quo of trauma rescue and treatment, analyze the existing problems, and propose improvement measures for further big data platforms in China and worldwide.

### 2.2. Definitions

#### 2.2.1. Trauma patients

Trauma patients refer to patients whose tissue structures in organisms were damaged and whose dysfunction was caused by the injury-causing factors such as impact, machinery, violence, etc.

For HQMS data, trauma patients were defined as the inpatients whose main discharge diagnosis results on the front pages of their hospital medical records show trauma-related diseases, which were diagnosed to be trauma or injury related in the S segment, T segment and other coding segments of ICD-10 codes and excluded non-trauma related diagnostic codes. A total of 3,265 diagnostic codes were included in ICD-10 codes. All related ICD codes in this report were listed in Supplementary Table 1.

#### 2.2.2. Causes of trauma

Causes of trauma refer to external factors causing damage to an organism's structures or functions. In this report, the causes of trauma for the patients in the HQMS database were classified into eight types according to the Medical Priority Dispatch System (MPDS), namely: ① traffic/transportation incidents; ② falls; ③ stab/gunshot/penetrating trauma; ④ blunt/violent attacks/impact injuries; ⑤ animal attacks/bites; ⑥ explosion/burn injuries; ⑦ machinery-related injuries; ⑧ and other causes of trauma. Based on the ICD-10 codes for “external causes of injuries and poisoning” on the front page of each hospital medical record, the causes of trauma for patients were classified based on the seven types above. The ICD-10 code corresponding to each cause of trauma was detailed in Supplementary Table 2.

The causes of trauma on the big data platform of CTRTA were classified into six categories by reference to MPDS, namely: ① traffic/transportation incidents; ② high altitude falling; ③ stab/gunshot/penetrating trauma; ④ violent attacks; ⑤ animal attacks/bites; ⑥ explosion/burn injuries.

#### 2.2.3. Severe trauma complications

Severe trauma complications refer to the complications in trauma patients that lead to their poor prognosis or endanger their lives. In this research, the diseases shown in the “disease diagnosis in discharge” on the front pages of hospital medical records and occurring after the admission of patients were diagnosed as complications. Definitions were given according to the severe trauma complications specified in government documents. There were a total of 133 severe trauma complications. Details have been shown in Supplementary Table 3.

#### 2.2.4. Glasgow coma scale and trauma index

GCS was used for patients to assess the degree of coma, the highest score was 15 points as awake; 13 to 14 were classified as mild coma; 9 to 12 were classified as moderate coma; not more than 8 were classified as severe coma; the lower the score, the more impaired the consciousness. The TI score was proposed by Kirkpatrick in 1,971 as a simple way to evaluate the degree of trauma. It included five aspects, namely injury site, injury type, circulation, respiration, and consciousness. Higher values were associated with greater injury severity. Values below 9 points indicated minor injury, values between 10 and 16 points indicated moderate injury, and values above 17 points indicated severe injury (12).

#### 2.2.5. Definition of six “regions” in China

We classified the country into six regions, namely North China, Northeast China, East China, Central South China, Southwest China and Northwest China. North China: Beijing, Tianjin, Hebei, Shanxi and Inner Mongolia; Northeast China: Liaoning, Jilin and Heilongjiang; East China: Shanghai, Jiangsu, Zhejiang, Anhui, Fujian, Jiangxi and Shandong; Central South China: Henan, Hubei, Hunan, Guangdong, Guangxi and Hainan; Southwest China: Chongqing, Sichuan, Guizhou, Yunnan and Tibet; Northwest China: Shaanxi, Gansu, Qinghai, Ningxia and Xinjiang.

## 3. Statistical analysis

Descriptive analysis was adopted in this paper. The *p*-value was not presented here because the considerable sample size of the HQMS may result in a statistically significant *p*-value, even though the absolute difference was tiny and might be of no clinical significance. For the quartile table that involved the duration of hospitalization and hospitalization expenses, categorical variables such as gender, age and the cause of trauma were expressed in percentage. SAS 9.4 was used as statistical analysis software (SAS Institute Inc., Cary, NC, USA).

## 4. Results

### 4.1. Age and gender characteristics of trauma patients

**Figure 1:**
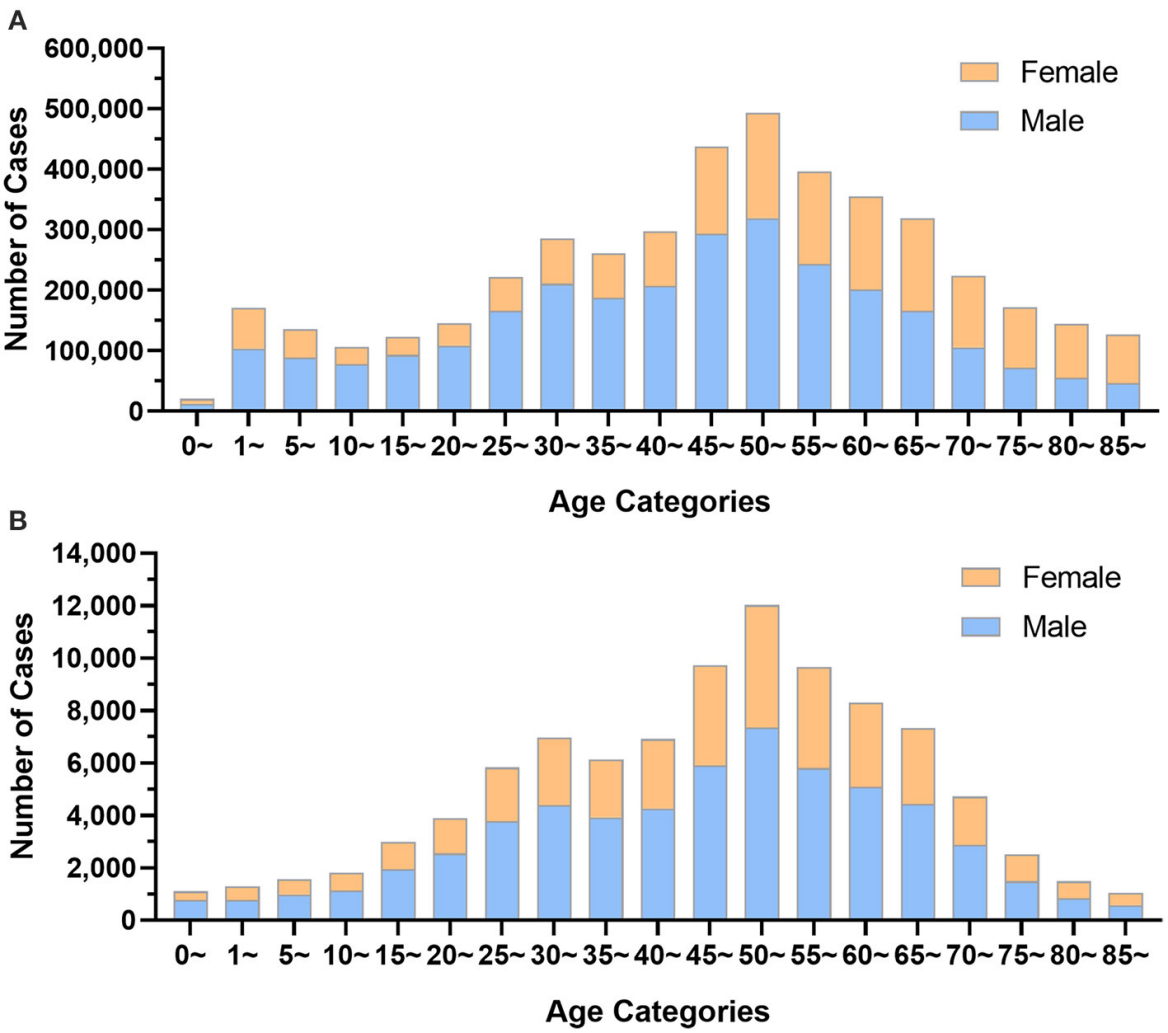
The age distribution of trauma patients by gender group. **(A)** Data from Hospital Quality Monitoring System; **(B)** Data from China Trauma Rescue and Treatment Association.

In 2019, the front page of the hospital medical record for 4,436,653 inpatients with trauma-related diseases was obtained by HQMS from 1,879 public tertiary hospitals nationwide. Each public tertiary hospital received an average of 2,361.18 cases in the year. The male-to-female ratio among the inpatients was 1.65:1, with 62.22% (2,760,694) being men and 37.78% (1,675,959) being women. However, among patients over 70 years old, the percentage of women was higher than that of men. Either for male or female inpatients, the age group 50–54 accounted for the largest proportion of patients of the same gender (male: 11.57%, 319,421 cases; female: 10.38%, 493,320 cases), followed by the age group 45–49 (male: 10.64%, 293,345 cases; female: 8.58%, 143,861 cases) and the age group 55–59 (male: 8.82%, 243,374 cases; female: 9.14%, 153,233 cases). The age group with the highest proportion of trauma was 50–54, accounting for 11.12%, as shown in Figure 1A.

The 2019 data of 95,376 trauma patients in 339 hospitals reported by CTRTA were collected by its big data platform. In the year, each member unit of CTRTA received an average of 281.35 cases, including 58,913 male patients (61.77%) and 36,463 female patients (38.23%), with the ratio of men to women being 1.61:1. Both high-proportion age groups for male (12.47%) and female (12.82%) trauma patients were 50 to 54 years old, as shown in Figure 1B.

### 4.2. Degrees of injuries for trauma patients

**Figure 2:**
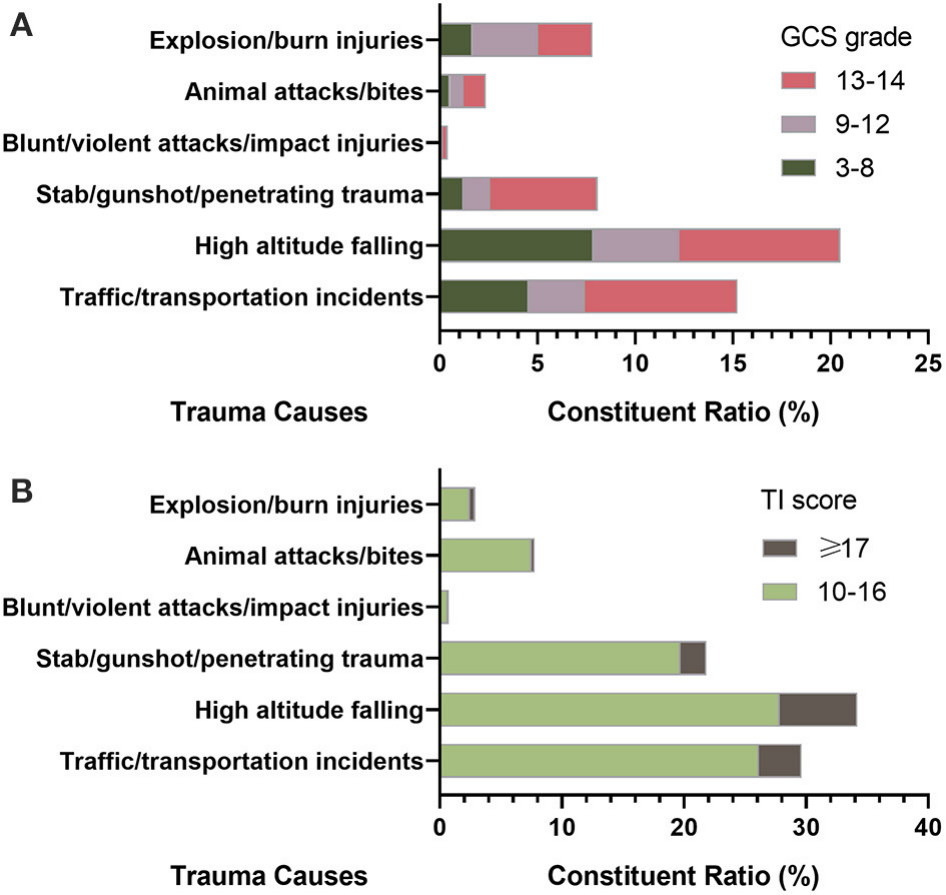
The constituent ratio of causes in trauma patients. **(A)** Patients with mild, moderate and severe coma classified by Glasgow Coma Scale (GCS) grade; **(B)** Patients with moderate and severe injury classified by Trauma Index (TI) score.

GCS was reported in 78,289 cases in the CTRTA database, accounting for 82.08% of total patients, of which those with severe coma (GCS ≤ 8 points) had a share of 4.61%. The constituent ratios of mild, moderate and severe traumas in patients who suffered from high altitude falling were higher than those of other causes, among which the slight coma accounted for 8.25%, moderate coma accounted for 4.39%, and the severe coma accounted for 7.86%, respectively, as shown in Figure 2A.

In the CTRTA database, TI was reported in 78.59% (74,953/95,376) of the hospital medical records, of which patients with severe trauma accounted for 4.63%. The constituent ratio of moderate and severe traumas in patients who suffered from high altitude falling was higher than that of other causes, among which the moderate traumas accounted for 27.83% and the severe traumas accounted for 6.38%, as shown in Figure 2B.

### 4.3. The hospital characteristics of inpatients in HQMS

According to HQMS data, the percentage of patients who sought medical care was 93.80% (4,161,703/4,436,653) in general hospitals, 1.86% (82,493/4,436,653) in orthopedic hospitals, and 1.73% (76,613/4,436,653) in children's hospitals.

### 4.4. The general distribution characteristics of trauma causes for inpatients in HQMS

A total of 3,924,111 (88.45%) hospitalized cases were included in the data given in this research, which exclusively examined the “external causes of injury and poisoning” term retrieved from the front page of the hospital medical record. Falls (1,589,544 cases, 40.51%) and traffic/transportation incidents (989,667 cases, 25.22%) were the most frequent trauma causes among trauma inpatients, accounting for a total of 65.73% of the causes.

#### 4.4.1. The distribution characteristics of trauma causes for different genders of inpatients in HQMS

**Figure 3:**
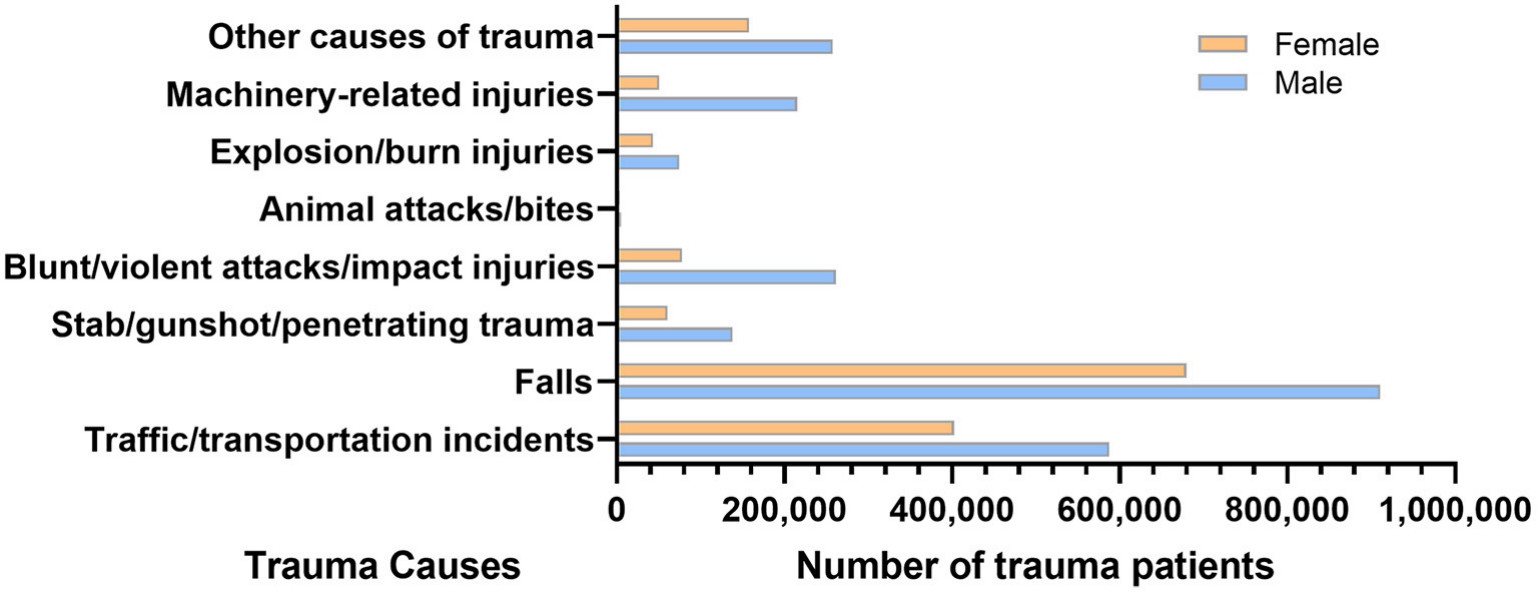
The number of trauma patients by trauma causes and gender in the hospital quality monitoring system.

According to HQMS data, falls were the leading cause of trauma for both male and female inpatients (male: 910,353 cases, 37.17%; female: 679,191 cases, 46.05%), while traffic/transportation events were the second-leading cause (male: 587,138 cases, 23.97%; female: 402,529 cases, 27.29%) (Figure 3, Table 1).

**Table 1:** The number and proportions of patients with different causes of trauma and different genders.

**Cause of trauma**	**Male (*n =* 2,449,258, 62.42%)**	**Female** ** (*n =* 1,474,853, 37.58%)**	**Total (*n =* 3,924,111, 100.00%)**
Traffic/transportation incidents	587,138 (59.33)	402,529 (40.67)	989,667
Falls	910,353 (57.27)	679,191 (42.73)	1,589,544
Stab/gunshot/penetrating trauma	138,182 (69.56)	60,479 (30.44)	198,661
Blunt/violent attacks/impact injuries	261,379 (77.07)	77,774 (22.93)	339,153
Animal attacks/bites	4,989 (58.44)	3,548 (41.56)	8,537
Explosion/burn injuries	74,497 (63.32)	43,163 (36.68)	117,660
Machinery-related injuries	214,943 (81.00)	50,435 (19.00)	265,378
Other causes of trauma	257,777 (62.04)	157,734 (37.96)	415,511

Data reported in this table analyzed only “external causes of injury and poisoning” term collected from front page of hospital medical record, a total of 3,924,111 (88.45%) hospitalized cases were included.

#### 4.4.2. The distribution characteristics of trauma causes for different age groups of inpatients in HQMS

**Figure 4:**
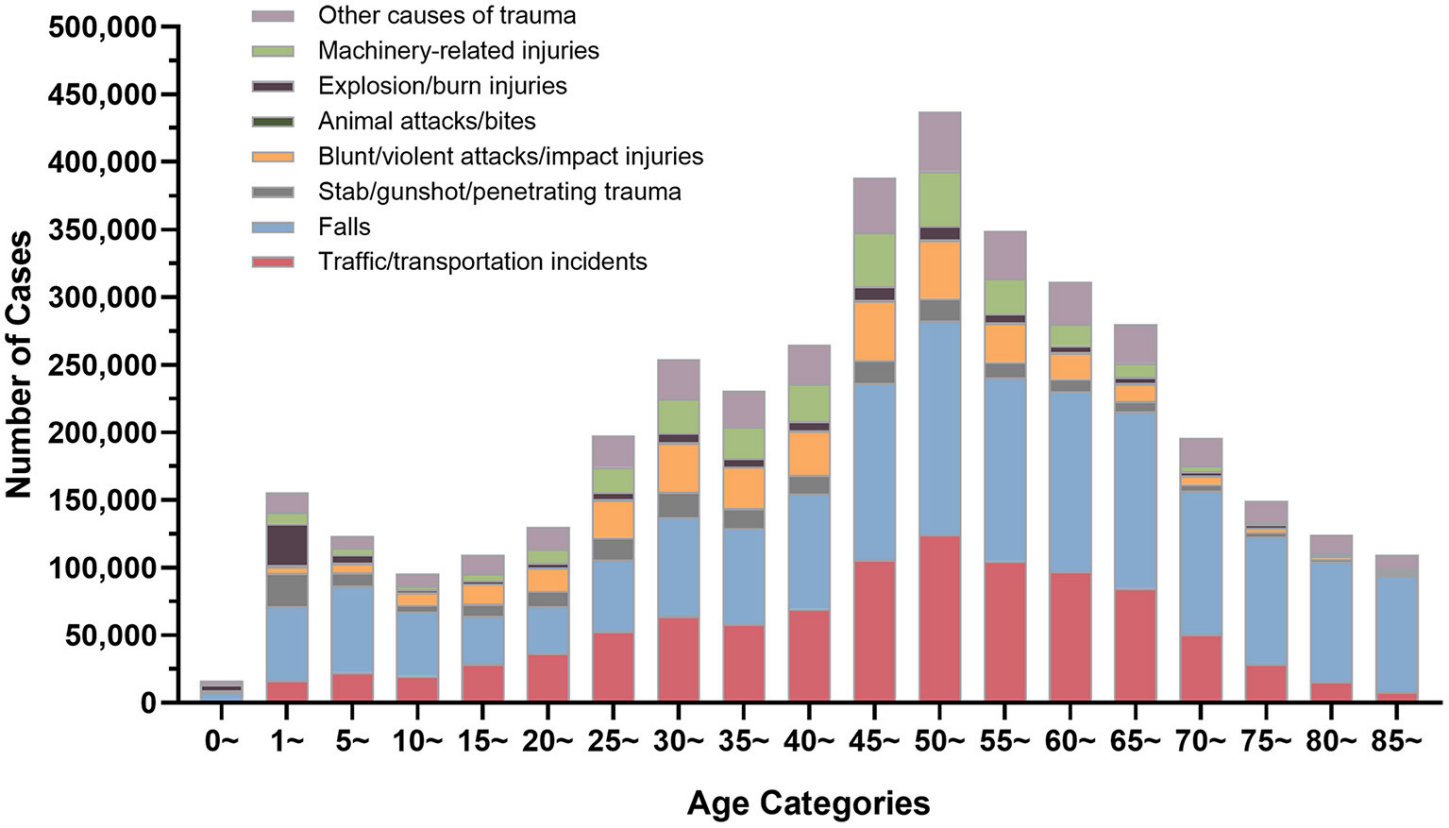
The distribution of trauma causes in trauma patients by age groups in the hospital quality monitoring system.

According to HQMS data, falls were the leading cause of trauma patients' injuries in most age categories (26.86–78.67%), with the exception of the 20–24-year-old group, where traffic/transportation incidents were the leading cause (36,707 cases, 26.33%). Traffic/transportation accidents were the second-leading cause of trauma for the majority of age groups (18.18–31.08%), explosion/burn injuries for children under five years old (0 years: 4,258 cases, 25.71%; 1–4 years: 30,898 cases, 19.88%), and falls for age group 20–24 (34,602 cases, 26.59%). For male patients, falls were the most frequent trauma cause (26.22–73.82%) in all age categories, and traffic/transportation events were the second most prevalent cause in all age groups except those under five. For female patients, traffic/transportation incidents constituted the most common trauma cause and fall the second one in the age group 15–49 analyzed. For other age groups, falls were the first cause of trauma. Regardless of male or female, explosion/burn injuries for under 5 years old were ranked second among all trauma causes (Figure 4).

#### 4.4.3. The distribution characteristics of trauma causes for different regions of inpatients in HQMS

HQMS data showed that among hospitalized patients in all regions, fall/fall injury was the first cause of trauma (38.07–45.72%), while road traffic injuries ranked second (23.08–27.67%).

### 4.5. Common diagnosis and surgical procedures of trauma inpatients in HQMS

The top ten trauma-related diagnoses accounted for 18.30% of all trauma patients, including seven orthopedic diagnoses and three craniocerebral diagnoses, which ranked as femoral neck fractures (137,028 cases, 3.09%), femoral intertrochanter fractures (109,155 cases, 2.46%), clavicle fractures (94,706 cases, 2.13%), lumbar fractures (93,176 cases, 2.10%), concussions (92,340 cases, 2.08%), rib fractures (80,713 cases, 1.82%), brain contusions (66,689 cases, 1.50%), head injuries (63,674 cases, 1.44%), patella fractures (61,297 cases, 1.38%), and thoracic fractures (59,251 cases, 1.34%).

The top ten surgical procedures accounted for 15.60% of all trauma patients. All of them were orthopedic surgeries, which ranked as open reduction internal fixation (ORIF) of tibia fracture (50,122 cases, 1.87%), artificial femoral head replacement (49,852 cases, 1.86%), Closed reduction and intramedullary nailing fixation of femoral fractures (48,498 cases, 1.81%), the ORIF of clavicle fracture (46,373 cases, 1.73%), percutaneous vertebroplasty (44,141 cases, 1.65%), the ORIF of radius fracture (38,432 cases, 1.44%), non-excision debridement of skin and subcutaneous tissue (37,158 cases, 1.39%), the ORIF of humeral fracture (36,921 cases, 1.38%), skin and subcutaneous necrotic tissue excision and debridement (36,298 cases, 1.36%), and total hip replacement (36,152 cases, 1.35%).

### 4.6. Severe trauma complications for trauma inpatients in HQMS

**Figure 5:**
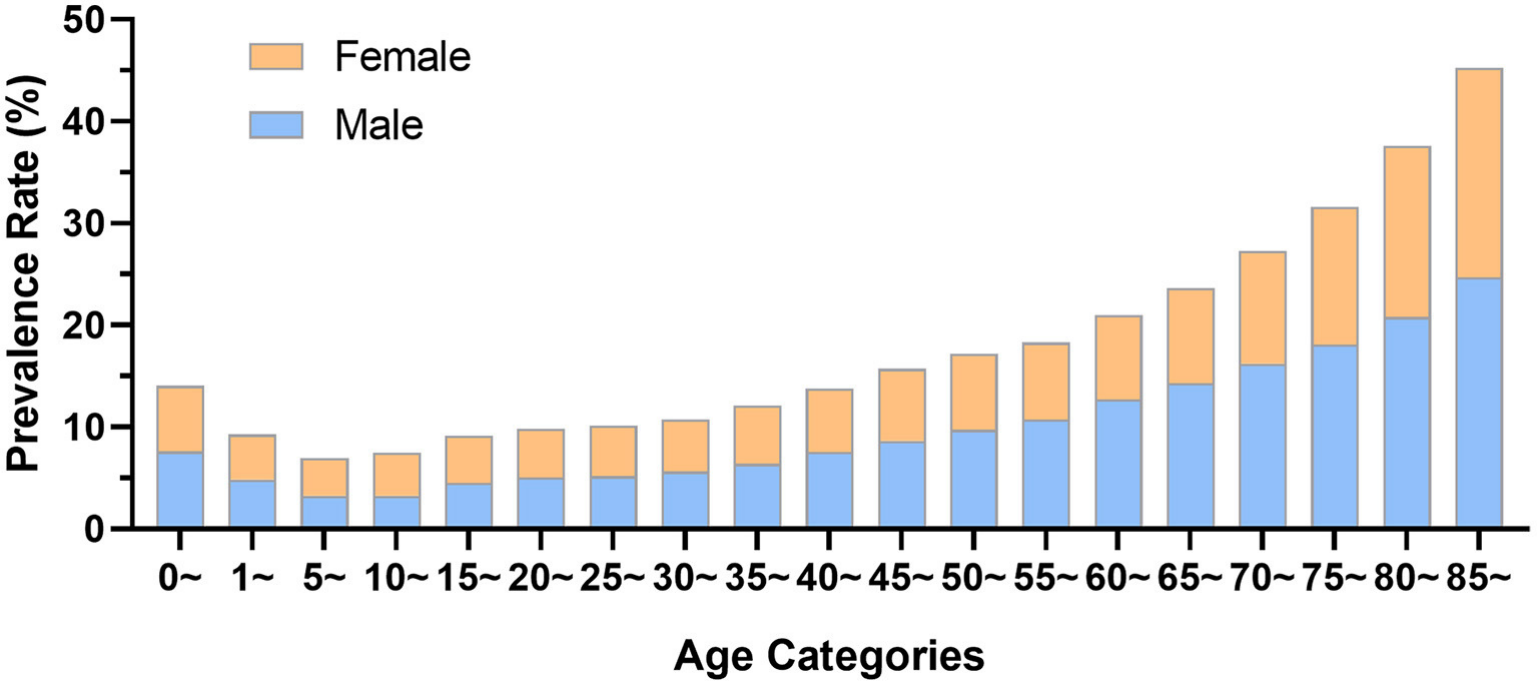
The prevalence rate of severe complications in trauma patients by age and gender in the hospital quality monitoring system.

For the hospitalized patients, the prevalence rate of severe trauma complications was 9.03% (400,408 cases), and this proportion was higher in males (254,676 cases, 9.23%) than in females (145,732 cases, 8.70%). The prevalence rate of severe trauma complications (27,990 cases, 22.11%) was the highest in the age group above 85, both male and female. Except for trauma patients under five-year-old, the prevalence rate of severe trauma complications increased with age in other groups (Figure 5).

### 4.7. The burden of disease and mortality rates of trauma inpatients in HQMS

#### 4.7.1. Hospitalization expenses of trauma inpatients in HQMS

**Figure 6:**
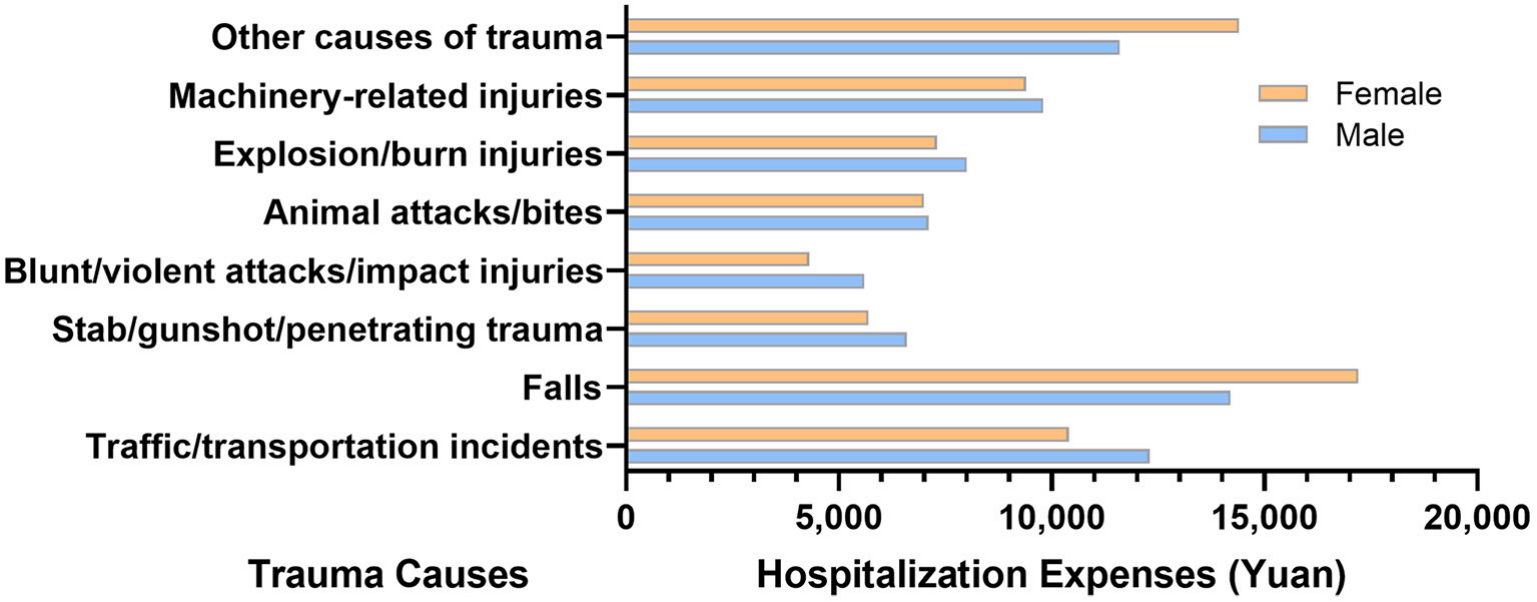
Total hospitalization expenses of trauma patients by trauma causes and gender in the hospital quality monitoring system.

According to HQMS data, the total hospitalization expenses for all trauma patients exceeded 100.38 billion (exactly 100,375,806,841) yuan, with a median of 11,100 (interquartile range [IQR] 4,600–28,700) yuan, 10,600 (IQR 4,600–27,300) yuan for males and 12,000 (IQR 4,600–30,800) yuan for females. It also increased significantly with age. Among all trauma causes, falls arose the highest median of the total hospitalization expenses, which was 15,400 (IQR 5,200–33,300) yuan (Figure 6).

#### 4.7.2. The length-of-hospital stay for trauma inpatients in HQMS

The median length-of-hospital stay for trauma patients was 9 (IQR 5–15) days for both genders, with the longest in the age groups from 50 to above 85 years (10 days for each, IQR 5–17), and the shortest in the age group 1–4 (5 days, IQR 2–8). In Beijing (6 days, IQR 4–11) and Shanghai (6 days, IQR 4–10), the length-of-hospital stay for trauma patients was shorter than that of other provinces. Compared with other trauma causes, traffic/transportation incidents had a relatively longer median length-of-hospital stay (11 days, IQR 6–20).

#### 4.7.3. The mortality rate of trauma inpatients in HQMS

**Figure 7:**
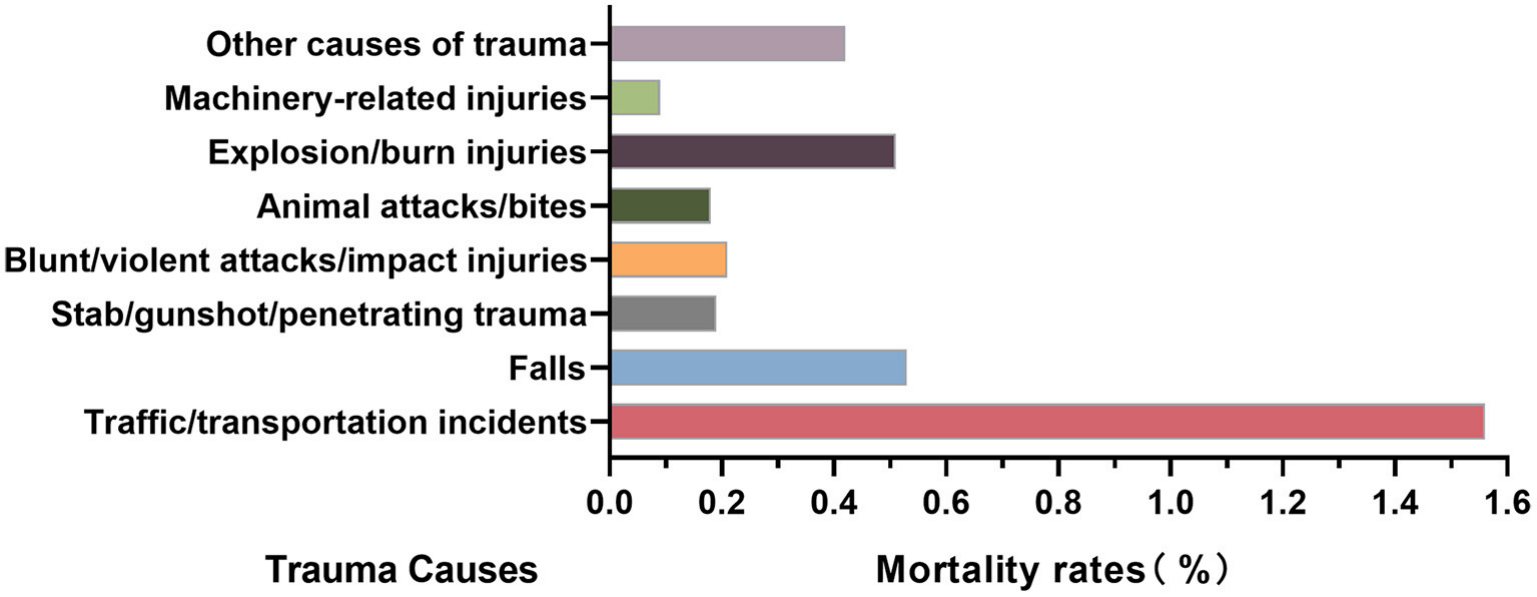
Mortality rates of trauma patients by causes in the hospital quality monitoring system.

The mortality rate of all trauma inpatients was 0.77%, 0.89% for males and 0.56% for females, respectively. It gradually increased with age after 30 year, with the highest in the age group above 85 years old (1.86%). The age groups 5–9 and 10–14 had the lowest mortality rates, which were 0.22% in each group. The mortality rate arising from traffic/transportation incidents was the highest among all trauma causes (1.56%, Figure 7). Among different regions, the mortality rate in Northeast China was the highest (1.13%), while that in East China was the lowest (0.65%). In both male and female patients, the mortality rate in Northeast China was the highest (male 1.27%, female 0.90%), while that in East China was the lowest (male 0.76, female 0.65%, Table 2).

**Table 2:** The number and mortality rate of patients in different region and different genders.

	**Male**		**Female**		**Total**	
	***n*** **(%)**	***N***	***n*** **(%)**	***N***	***n*** **(%)**	***N***
North China	2,779 (0.89)	3,12252	943 (0.52)	1,82133	3,722 (0.75)	494,385
Northeast China	3,033 (1.27)	238,590	1,302 (0.9)	144,037	4,335 (1.13)	382,627
East China	6,529(0.76)	863,423	2,666(0.48)	560,705	9,195 (0.65)	14,241,28
Central South China	6,626 (0.91)	731,580	2,381 (0.56)	424,695	9,007 (0.78)	11,562,75
Southwest China	4,037(1)	404,971	1,536 (0.63)	242,884	5,573 (0.86)	647,855
Northwest China	1,652 (0.79)	209,878	626 (0.52)	121,505	2,278 (0.69)	331,383
Total	24,656(0.89)	276,0694	9,454(0.56)	167,5959	34,110(0.77)	44,366,53

*n*, number of deaths; *N*, number of patients; %, mortality rate.

## 5. Discussions

With high incidence and disability rates, trauma has consumed lots of medical and health resources, and brought a heavy burden of disease to China, families and individuals (5). According to our report, inpatients in Chinese tertiary hospitals spent more than 100.38 billion yuan on hospitalization due to trauma in the year 2019 alone. With the development of the economy, road conditions and society, the composition of trauma has changed progressively. And China is facing increasing challenges in reducing the incidence rate of injuries, the mortality rate, and the burden of disease.

This report sorts out and analyzes data from HQMS and the big data platform of CTRTA. The patient data from the two sources differs in content and has apparent characteristics. For example, HQMS data provide front-page information on discharge hospital medical records of inpatients, while the big data platform of CTRTA contains outpatient and emergency treatment-related information. A comparison of the analysis findings from the two data sources reveals that, despite the content differences between the patient data from the two sources, the partial results of the data are very compatible with one another. For instance, the age range 50–54 shows the highest prevalence of traumatic disorders, which is consistent across both sources. This consistency demonstrates the accuracy of the data provided.

The epidemiological characteristics of trauma in China and the rest of the world have similarities and differences. According to the 2016 data from the National Trauma Data Bank (NTDB), falls and traffic/transportation incidents represented the top two causes of trauma, with percentages of 44.18 and 39.03%, respectively, which were quite close to each other (13, 14). The two causes accounted for 83.21% of all trauma causes. Although falls and traffic/transportation incidents were also considered the two most common causes of trauma in China, the country had different percentages from other countries. In China, the percentage of falls was 41.74%, close to that of international data; while the percentage of traffic/transportation incidents was only 16.99%, far lower than that of the rest of the world. Whether in China or the United States, patients suffering from traffic/transportation incidents had the highest mortality rate. Additionally, the age group with a high incidence of trauma in the United States was 20 to 24 years old (15), while that in China was 50 to 54 years old, which is related to the age distribution characteristics of the population. By comparing the population of all age groups in the sixth census of China in 2010, the age distribution of trauma patients was consistent with the general distribution of the population (16).

In this report, the mortality rate of inpatients was only 0.77%, far lower than that of other countries (17–19), which may be related to unique customs in China. In the country, family members of patients, if possible, hope more that they could return home and die in the presence of their families and relatives, rather than receive final rescue in a hospital. In this case, the mortality rate in China would be much lower than that in other countries (6, 20). The data differences between China and other countries prompt us to think about the reasons behind these differences, and also indicate that it is urgent for China to have its epidemiological investigations or reports on trauma-related diseases.

Successful experience abroad has shown that trauma rescue and treatment is a systematic project that must be implemented based on a complete rescue and treatment system (17, 21). Most of the trauma rescue and treatment systems in European and American countries are based on independent trauma rescue and treatment centers (22, 23). By contrast, although there is a lack of trauma centers with independent organizational structures in major Chinese cities, an ample number of large-scale tertiary general hospitals or secondary general hospitals with relatively complete departments have set up various specialties for trauma rescue and treatment. The NCTM at Peking University People's Hospital has taken the lead in proposing a new model of trauma rescue and treatment featuring “establishing a trauma rescue and treatment team instead of an independent trauma center in a general hospital,” and setting up a “regional closed-loop trauma rescue and treatment system with general hospitals at the core” nationwide (24, 25). According to the data of this research, 93.8% of the patients sought medical treatment in general hospitals and mainly suffered from neurosurgery- and orthopedics-related injuries as their primary post-traumatic injuries. Therefore, the trauma rescue and treatment centers established based on general hospitals, neurosurgery and orthopedics will be more consistent and appropriate to the characteristics of traumatic diseases as well as the basic national conditions of China. Additionally, a multi-center study has confirmed that, under this trauma rescue and treatment model, the average duration of trauma rescue and treatment has been shortened by more than 50% and the in-hospital mortality rate for severe trauma rescue and treatment dropped from 33.8% before intervention to 20.5% after the intervention. At a critical trauma rescue and treatment center, the mortality rate of severe trauma dropped to less than 8%, with a better treatment effect than the average level abroad (24). Thus, it is necessary to continue the promotion of the trauma rescue and treatment system based on this model in a more extensive scope across the country.

This report shows that the length-of-hospital stay for trauma patients in China's economically developed provinces is significantly shorter than in other provinces. This is related to the fact that China's trauma centers and trauma rescue and treatment systems have just got started. Significant differences exist in the trauma rescue and treatment levels of different regions. Therefore, based on the platform of the NCTM, we will further strengthen the construction of trauma centers and regional trauma rescue and treatment systems, promote diagnosis and treatment norms and standards, further increase exchanges and cooperation among different regions and hospitals, learn from each other, make joint progress, and continuously promote the homogenization of trauma rescue and treatment in different regions of China and the continuous improvement of trauma rescue and treatment capabilities (26).

In order to improve the capabilities of trauma treatment centers, attention should be paid to the training of self-rescue and mutual rescue skills of the public. According to the Global Burden of Disease Study 2020, traffic/transportation incidents have a greater impact on disability-adjusted life years than falls and involve a much heavier burden of disease (3). Furthermore, for traffic/transportation incidents, the public is the first witness at the scene in most cases. Before medical personnel arrives, the correct self-rescue and mutual rescue of the public during accidents will lay a solid foundation for the subsequent medical rescue.

Traffic/transportation incidents are the top cause of death from trauma in China. Moreover, reducing the mortality rate arising from traffic/transportation incidents requires not only the efforts of medical and health departments, but also a full collaboration with competent government authorities (27). With reference to China's experience of a significant decrease in the death rate from traffic/transportation incidents following the promulgation of the Law of The People's Republic of China on Road Traffic Safety in 2004, the effect of helmets, seat belts, safety seats, and other related safety facilities may be highlighted through public education and by increasing traffic safety awareness (28, 29). It can be found that the mortality rate and the incidence of complications from injuries due to falls have increased significantly among elderly patients. In order to reduce the risk of falls in the elderly as well as the incidence of trauma-related diseases in elder inpatients, the safety awareness of the public can be improved through science education on the one hand. On the other hand, trauma can be avoided by improving or modifying inappropriate household facilities at home, such as installing ramps, toilet handrails, night lights, etc. With the development of China's road traffic facilities and automobile industry, it is predicted that the percentage of traffic/transportation incidents will rapidly increase in the following 2 decades. Meanwhile, the percentage of fall-related injuries will gradually rise as aging intensifies. Thus, it can be expected that reducing the incidence rates of fall-related and traffic/transportation injuries and improving the success rate of rescue and treatment will become essential topics for China's trauma discipline in the next 20 years.

### 5.1. Limitation

The following factors should be taken into account when interpreting our results: HQMS database involved only tertiary hospitals; ① there may be selection bias due to the limitations of data sampling; ② the ICD codes used for defining specific diseases may be low in sensitivity or high in specificity due to the impact of various diagnosis and coding programs; ③ our report only contains cross-sectional data. Thus, there may be some difficulties in causal inference.

## 6. Conclusion

This report summarizes the demographic characteristics, distribution of trauma causes, complication incidences, disease burden, mortality rate, and other data concerning trauma inpatients in China. The epidemiological characteristics of trauma in China were different from other countries. It should be noted that traffic/transportation incidents and falls require not only the efforts of medical and health departments, but also full collaboration with competent government authorities. At the same time, there is a need to reduce regional differences in the level of trauma rescue and treatment, which means that relative departments and medical institutions should continue to further strengthen the construction of trauma centers and regional trauma rescue and treatment systems. This report provides nationally representative and up-to-date evidence for trauma rescue and treatment that will help guide healthcare resource allocation and provide directions for future research in trauma prevention and management in China.

## Data availability statement

The original contributions presented in the study are included in the article/Supplementary material, further inquiries can be directed to the corresponding authors.

## Ethics statement

The studies involving human participants were reviewed and approved by the Ethics Committee of the Peking University People's Hospital. Written informed consent from the participants' legal guardian/next of kin was not required to participate in this study in accordance with the national legislation and the institutional requirements.

## Author contributions

YW, TW, BJ, and CW designed the research. CW, PH, GK, and HW organized the database. CW, YS, and LG performed the statistical analysis. PH, CW, YW, and LG wrote the first draft of the manuscript. BJ, TW, and GK thoroughly revised the manuscript. All authors contributed to manuscript revision and approved the submitted version.
