# Supplementary material for: China trauma treatment statistics 2019: A national retrospective study based on hospitalized cases
Source: Front Public Health. 2023 Feb 24;11:1116828. doi: 10.3389/fpubh.2023.1116828 (PMC9998676; doi:10.3389/fpubh.2023.1116828)
Supplement: Supplementary file 1 [file Data_Sheet_1.PDF]

Supplementary Material

China trauma treatment statistics 2019: A national retrospective study based on hospitalized cases

Yanhua Wang[1#], Chu Wang[2#], Pan Hu[2#], Haibo Wang[3#], Lanxia Gan[4], Guilan Kong[5], Ying Shi[4], Tianbing Wang[2*], Baoguo Jiang[2*]

*** Correspondence:**

Baoguo Jiang, email: jiangbaoguo@vip.sina.com

Tianbing Wang, email: wangtianbing@pkuph.edu.cn

1 Supplementary Tables

Supplementary Table 1. ICD-10 Code for Trauma Disease Diagnosis

ICD-10 encoding	Diagnosis of Trauma Diseases
S00.000	Superficial scalp injury
S00.000x053	Organization of scalp hematoma
S00.001	Scalp contusion
S00.002	Scalp abrasions
S00.003	Foreign body on scalp
S00.004	Scalp hematoma
S00.100	Contusion of eyelid and periocular area
S00.100x001	blepharal contusion
S00.100x003	Eyelid congestion
S00.100x006	Contusion of eyebrow arch
S00.101	Contusion of periocular region
S00.102	Frontal hematoma
S00.200	Other superficial injuries of eyelid and periocular area
S00.201	Superficial injury of orbital region
S00.202	Eyelid hematoma
S00.300	Superficial nasal injury
S00.300x001	Superficial injury of nose
S00.300x051	Nasal contusion
S00.302	Nosebleed
S00.400	Superficial ear injury
S00.400x051	Contusion of auricle
S00.400x052	External ear contusion
S00.400x053	Auricular hematoma
S00.401	Contusion of auricle
S00.402	Hematoma of auricle
S00.403	Contusion of tympanum
S00.404	Contusion of tympanic membrane
S00.500	Superficial injury of lips and mouth
S00.500x051	Contusion of lip
S00.500x052	Contusion of mandible
S00.501	Superficial oral injury
S00.700	Multiple superficial head injuries
S00.800	Superficial injuries to other parts of the head
S00.800x041	Superficial facial injury with foreign body
S00.800x053	Contusion of cheek
S00.800x054	Contusion of lower jaw
S00.800x055	Contusion of forehead
S00.800x056	Temporal contusion
S00.801	Facial soft tissue contusion
S00.802	Facial abrasions
S00.803	Facial contusion
S00.804	Superficial foreign body on face
S00.900	Superficial head injury

S00.900x001	Superficial head injury
S01.000	Open wound of scalp
S01.000x002	Laceration of eyebrow arch
S01.001	Scalp laceration
S01.100	Open wound of eyelid and periocular area
S01.100x001	Open injury of orbit
S01.100x002	Foreign body of eyelid caused by trauma
S01.101	Eyelid laceration
S01.102	Open eyelid foreign body
S01.103	Orbital laceration
S01.200	Open wound of nose
S01.200x011	Open wound of nasal epidermis
S01.200x021	Open wound of nostril
S01.200x031	Open injury of nasal septum
S01.200x091	Open wound of nose with sphenoid sinus foreign body
S01.200x092	Traumatic nasal defect
S01.300	Open wound of ear
S01.300x002	Open injury of ear canal
S01.300x011	Open injury of external ear wing
S01.300x012	Open injury of auricle
S01.300x031	Open injury of tragus
S01.300x051	Open injury of eustachian tube
S01.300x061	Open injury of auditory ossicles
S01.300x071	Open injury of middle ear
S01.300x081	Open injury of cochlea
S01.301	Open external auditory canal injury
S01.302	Open postauricular injury with foreign body
S01.400	Open wound of cheek and temporomandibular region
S01.400x011	Open injury of cheek
S01.400x021	Open injury of maxilla
S01.400x031	Open injury of palate
S01.401	Open temporomandibular injury
S01.500	Open wound of lips and mouth
S01.500x001	Open mouth injury
S01.500x021	Open injury of oral mucosa
S01.500x022	Open injury in cheek
S01.500x031	Open injury of gingiva
S01.500x042	Open injury of tongue and floor of mouth
S01.500x051	Open injury of upper palate
S01.500x052	Open injury of soft palate
S01.501	Laceration of tongue
S01.502	Open tongue injury
S01.503	Gingival laceration

S01.504	Open lip injury
S01.505	Penetrating wound of soft palate
S01.506	Laceration of lip
S01.700	Multiple open wounds on the head
S01.800	Open wounds in other parts of the head
S01.800x011	Open craniocerebral injury with fracture
S01.800x021	Open craniocerebral injury with dislocation
S01.800x031	Open craniocerebral injury with intracranial injury
S01.800x081	open brain injury
S01.800x082	Intracranial foreign body
S01.800x083	Open injury of skull
S01.800x085	Facial foreign body
S01.800x086	Open injury of forehead
S01.800x087	Open injury of lower jaw
S01.801	Open intracranial foreign body
S01.802	Facial laceration
S01.803	Open facial injury
S01.804	Open rupture of parotid duct
S01.900	Open wound on the head
S02.000	Skull fornix fracture
S02.000x003	Squamous fracture of temporal bone
S02.000x004	Scaly fracture of frontal bone and temporal bone
S02.000x005	Fracture of frontal bone and parietal bone
S02.001	Frontal bone fracture
S02.002	Fracture of parietal bone
S02.011	Open fracture of frontal bone
S02.012	Open fracture of parietal bone
S02.100	Fracture of skull base
S02.100x001	Skull base fracture
S02.100x002	Fracture of anterior cranial fossa
S02.100x003	Fracture of middle cranial fossa
S02.100x004	Posterior fossa fracture
S02.100x006	Orbital roof fracture
S02.100x008	Frontal sinus fracture
S02.100x009	Sphenoid fracture
S02.101	Occipital fracture
S02.102	Temporal bone fracture
S02.103	Ethmoid sinus fracture
S02.111	Open skull base fracture
S02.112	Open occipital fracture
S02.113	Open temporal bone fracture
S02.114	Open fracture of ethmoid sinus

S02.200	Nasal bone fracture
S02.200x002	Fracture of nasal septum
S02.211	Open nasal bone fracture
S02.300	Orbital floor fracture
S02.300x002	Orbital floor comminuted fracture
S02.311	Open fracture of orbital floor
S02.400	Zygomatic and maxillary fractures
S02.400x001	Zygomatic arch fracture
S02.400x003	Maxillary fracture
S02.400x005	Maxillary sinus fracture
S02.401	Zygomatic fracture
S02.411	Open maxillary fracture
S02.412	Open zygomatic fracture
S02.500	Tooth fracture
S02.500x001	Traumatic tooth fracture
S02.500x002	Traumatic tooth breakage
S02.501	Traumatic tooth loss
S02.600	Mandibular fracture
S02.600x011	Condylar fracture
S02.600x021	Fracture of inferior condyle
S02.600x031	Fracture of coronoid process of mandible
S02.600x041	Mandibular ramus fracture
S02.600x051	Fracture of jaw angle
S02.600x061	Fibrochondral body fracture of mandible
S02.600x081	Mandibular body fracture
S02.600x091	Compound fracture of mandible
S02.600x101	Open condylar fracture
S02.611	Open mandibular fracture
S02.700	Multiple fractures involving skull and facial bone
S02.700x001	Multiple fracture of skull
S02.700x002	Multiple fractures of skull and facial bone
S02.700x004	naso-orbital-ethmoid fracture
S02.701	Multiple facial bone fractures
S02.711	Open multiple facial bone fractures
S02.712	Open multiple skull fractures
S02.800	Skull and facial fractures, others
S02.800x003	Fracture of upper palate
S02.801	Orbital bone fracture
S02.802	Alveolar bone fracture
S02.803	Palatal fracture
S02.810	Specifically refers to open skull and facial bone fractures
S02.811	Open fracture of orbital bone
S02.812	Open alveolar bone fracture

S02.813	Open fracture of palate
S02.900	Skull and facial fractures
S02.900x002	Skull fracture
S02.901	Facial bone fracture
S02.902	Depressed fracture of skull
S02.911	Open skull fracture
S02.912	Open facial bone fracture
S03.000	Dislocation of jaw joint
S03.000x001	Dislocation of jaw cartilage
S03.000x002	Dislocation of mandibular joint
S03.000x003	Temporomandibular joint dislocation
S03.100	Dislocation of nasal septal cartilage
S03.200	Tooth dislocation
S03.200x001	Traumatic tooth dislocation
S03.300	Dislocation of other and unspecified parts of the head
S03.301	Head dislocation
S03.400	Sprain and strain of jaw joint
S03.400x001	Temporomandibular joint injury
S03.400x002	Temporomandibular ligament injury
S03.500	Sprain and strain of joints and ligaments in other and unspecified parts of the head
S03.501	Sprain and strain of head joints and ligaments
S04.000	Injury of optic nerve and optic pathway
S04.000x001	Optic nerve injury
S04.000x002	Optic chiasma injury
S04.000x003	Visual pathway damage
S04.000x004	Damage of visual cortex
S04.100	Injury of oculomotor nerve
S04.200	Trochlear nerve injury
S04.300	Trigeminal nerve injury
S04.400	Abducent nerve injury
S04.400x001	Abducent nerve injury
S04.500	Facial nerve injury
S04.501	Facial nerve rupture
S04.502	Injury of infraorbital nerve
S04.600	Auditory nerve injury
S04.700	Injury of accessory nerve
S04.800	Brain nerve damage, others
S04.801	Hypoglossal nerve injury
S04.802	Olfactory nerve injury
S04.803	Injury of glossopharyngeal nerve
S04.804	Vagus nerve injury
S04.900	Brain Nerve Injury

S05.000	Damage of conjunctiva and corneal abrasion, no foreign body mentioned
S05.000x002	Conjunctival injury
S05.001	Corneal abrasion
S05.002	Corneal abrasion
S05.100	Contusion of eyeball and orbital tissue
S05.100x004	Contusion of eyeball
S05.101	Orbital contusion
S05.102	Traumatic hyphema
S05.103	contusion of lens
S05.104	Scleral contusion
S05.200	Eye laceration and rupture with prolapse or loss of intraocular tissue
S05.200x001	Traumatic vitreous incarceration
S05.200x002	Traumatic vitreous overflow
S05.200x003	Traumatic iridodialysis
S05.200x004	Traumatic iris defect
S05.200x005	Traumatic incarceration of lens
S05.201	Corneal penetrating injury with incarceration of iris
S05.202	Corneal penetrating injury with iris prolapse
S05.203	Corneal penetrating injury with lens incarceration
S05.204	Corneal penetrating injury with vitreous incarceration
S05.205	Traumatic iris prolapse
S05.206	Traumatic incarceration of iris
S05.207	Traumatic iris hernia
S05.208	Traumatic ciliary body prolapse
S05.209	Traumatic vitreous prolapse
S05.210	Traumatic vitreous hernia
S05.300	Eye laceration without prolapse or loss of intraocular tissue
S05.300x003	Conjunctival laceration
S05.300x004	Eyeball rupture
S05.300x005	Traumatic anterior chamber angle splitting
S05.300x010	Laceration of eye
S05.300x011	Lamellar laceration of cornea
S05.301	Corneal laceration
S05.302	Full-thickness corneal laceration
S05.303	Iris laceration
S05.304	Scleral laceration
S05.305	Laceration of ciliary body
S05.400	Orbital penetrating injury with or without foreign body
S05.400x001	Intraorbital foreign body
S05.400x002	Eyemuscle foreign body

S05.401	Orbital penetrating wound
S05.500	Penetrating injury of eyeball with foreign body
S05.500x001	Intraocular foreign body
S05.500x002	Penetrating injury of eyeball with magnetic foreign body
S05.500x003	Penetrating injury of eyeball with non-magnetic foreign body
S05.600	Penetrating injury of eyeball without foreign body
S05.600x002	Penetrating injury of eyeball
S05.601	Penetrating wound of cornea
S05.602	Penetrating injury of iris
S05.603	Penetrating wound of lens
S05.604	Penetrating wound of sclera
S05.605	Penetrating injury of retina
S05.700	Eye avulsion injury
S05.800	Other eye and orbital injuries
S05.800x001	Traumatic iris detachment
S05.800x007	Rupture of extraocular muscles
S05.800x008	Rupture of medial rectus muscle
S05.800x009	Rupture of external rectus muscle
S05.801	Ocular concussion
S05.802	Ocular contusion
S05.803	Corneal injury
S05.804	Iris injury
S05.805	Lens injury
S05.806	Traumatic dislocation of lens
S05.807	Scleral injury
S05.808	Retinal concussion
S05.809	Retinal injury
S05.810	Laceration of lacrimal canaliculus
S05.811	Lacrimal duct injury
S05.812	Shock wave blindness
S05.900	Eye and orbital injuries
S05.900x003	Open eye injury
S05.901	Eye injury
S05.902	Traumatic blindness
S05.903	Vitreous injury
S06.000	cerebral concussion
S06.100	Traumatic cerebral edema
S06.100x001	traumatic brain edema
S06.200	Diffuse brain injury
S06.200x001	Diffuse brain injury
S06.200x002	Diffuse cerebellar injury
S06.200x011	Diffuse brain injury with hemorrhage
S06.200x021	Diffuse cerebellar injury with hemorrhage

S06.200x031	Multiple intracerebral hemorrhage
S06.200x032	Multiple cerebral hematoma
S06.200x033	Multiple cerebellar hematoma
S06.200x081	Multiple cerebral contusion and laceration
S06.200x082	Multiple cerebellar contusion and laceration
S06.201	Brain stem contusion
S06.202	Cerebral contusion
S06.203	Laceration of brain
S06.204	Traumatic brain hernia
S06.205	Traumatic brain compression
S06.206	Diffuse axonal injury
S06.211	Open brain contusion
S06.300	Local brain injury
S06.300x001	Focal brain injury
S06.300x002	Focal cerebellar injury
S06.300x011	Focal cerebral contusion with hemorrhage
S06.300x021	Focal cerebellar contusion with hemorrhage
S06.300x031	Focal cerebral contusion with hematoma
S06.300x032	Focal cerebral contusion with massive hemorrhage
S06.300x041	Focal cerebellar contusion with hematoma
S06.300x042	Focal cerebellar contusion with massive hemorrhage
S06.300x081	Focal cerebral contusion and laceration
S06.300x082	Focal cerebellar contusion and laceration
S06.301	Traumatic focal cerebral hemorrhage
S06.302	Traumatic cerebral hematoma
S06.310	Open focal brain injury
S06.400	Epidural hemorrhage
S06.400x001	Traumatic epidural hematoma
S06.400x002	Traumatic epidural hemorrhage
S06.401	Traumatic closed epidural hematoma
S06.410	Open epidural hemorrhage
S06.500	Traumatic subdural hemorrhage
S06.500x001	Traumatic subdural hemorrhage
S06.500x002	Traumatic subdural hematoma
S06.500x004	Acute traumatic subdural hematoma
S06.500x005	Subacute traumatic subdural hemorrhage
S06.500x006	Subacute traumatic subdural hematoma
S06.501	Traumatic acute subdural hemorrhage
S06.510	Open subdural hemorrhage

S06.600	Traumatic subarachnoid hemorrhage
S06.600x001	Traumatic subarachnoid hemorrhage
S06.600x002	Traumatic subarachnoid hematoma
S06.600x011	Open subarachnoid hemorrhage
S06.610	Open subarachnoid hemorrhage
S06.700	Intracranial injury with prolonged coma
S06.700x001	Mild closed craniocerebral injury
S06.700x002	Closed craniocerebral injury medium
S06.700x003	Severe closed craniocerebral injury
S06.700x004	Closed craniocerebral injury
S06.700x005	Mild open craniocerebral injury
S06.700x006	Open craniocerebral injury medium
S06.700x007	Severe open craniocerebral injury
S06.700x008	Very severe open craniocerebral injury
S06.710	Open intracranial injury with prolonged coma
S06.800	Intracranial injuries, others
S06.800x002	traumatic intracerebral hematoma
S06.800x004	Traumatic cerebellar hematoma
S06.800x005	Traumatic cerebellar contusion
S06.800x007	Traumatic intracranial hematoma
S06.800x009	traumatic intracranial aneurysm
S06.800x010	Traumatic cerebral infarction
S06.800x011	Traumatic intracranial pneumatosis
S06.800x012	Traumatic hydrocephalus
S06.800x013	Traumatic subdural effusion
S06.801	Traumatic cerebellar hemorrhage
S06.802	Traumatic cerebral hemorrhage
S06.803	Traumatic brain stem hemorrhage
S06.804	Traumatic intracranial hemorrhage
S06.805	Traumatic intracranial cavernous sinus injury
S06.811	Open cerebral hemorrhage
S06.812	Open brainstem hemorrhage
S06.813	Open cerebellar hemorrhage
S06.814	Open intracranial hemorrhage
S06.900	Intracranial injury
S06.900x002	Traumatic brain injury
S06.901	Brain stem injury
S06.910	Open intracranial injury
S06.911	Open brain stem injury
S06.912	Open intracranial cavernous sinus injury
S07.000	Facial crush injury

S07.000x001	Face thermal crush injury
S07.100	Cranial crush injury
S07.100x001	Thermal crush injury of skull
S07.800	Crushing injury of other parts of the head
S07.900	Head crush injury
S07.900x001	Head thermal crush injury
S08.000	Scalp avulsion
S08.100	Traumatic ototomy
S08.100x001	Traumatic ototomy
S08.800	Traumatic amputation of other parts of the head
S08.801	Traumatic rhinotomy
S08.900	Traumatic amputation of the head
S09.000	Head vascular injury, which cannot be classified elsewhere
S09.000x001	Head vascular injury
S09.100	Head muscle and tendon injuries
S09.100x001	Head muscle injury
S09.101	Head tendon injury
S09.200	Traumatic rupture of eardrum
S09.200x001	Traumatic perforation of tympanic membrane
S09.700	Multiple head injuries
S09.800	Other specific head injuries
S09.800x002	Traumatic primary teeth injury
S09.800x003	Traumatic sinus hematocele
S09.801	Traumatic nasal septum hematoma
S09.900	Head injury
S09.900x006	Lip injury
S09.901	Facial injury
S09.902	Eyebrow injury
S09.903	Nasal injury
S09.904	Ear injury
S09.905	Auricle injury
S09.906	Tongue injury
S09.907	Salivary gland injury
S10.000	Throat contusion
S10.000x003	Contusion of pharynx
S10.001	Laryngeal contusion
S10.002	Vocal cord contusion
S10.003	Contusion of cervical esophagus
S10.004	Contusion of trachea
S10.100	Other and unspecified superficial injuries of throat
S10.101	Pharyngeal hematoma
S10.102	Superficial injury of throat
S10.700	Multiple superficial injuries in the neck

S10.800	Superficial injuries in other parts of the neck
S10.801	Superficial injury of epiglottis
S10.900	Superficial injury of neck
S10.900x001	Superficial injury of neck
S10.900x041	Superficial injury of neck with foreign body
S10.901	neck injury
S10.902	Foreign body in neck
S11.000	Open wound of neck involving throat and trachea
S11.001	Open trachea injury
S11.002	Open laryngeal injury
S11.003	Open cervical trachea rupture
S11.004	Penetrating injury of larynx and trachea
S11.100	Open wound of neck involving thyroid
S11.100x001	Open injury of thyroid
S11.200	Open wound of neck involving pharynx and cervical esophagus
S11.201	Open pharyngeal injury
S11.202	Open cervical esophageal injury
S11.700	Multiple open wounds on the neck
S11.700x001	Multiple open injuries of neck
S11.800	Open wounds in other parts of the neck
S11.800x011	Open neck injury with cervical fracture
S11.800x021	Open neck injury with cervical dislocation
S11.800x081	Open injury of epiglottis
S11.800x082	Open injury of supraclavicular region
S11.900	Open wound of neck
S11.900x001	Open injury of neck
S12.000	Fracture of the first cervical vertebra
S12.000x002	atlas fracture
S12.010	Open fracture of the first cervical vertebra
S12.100	Fracture of the second cervical vertebra
S12.100x001	Ring vertebra fracture (Jefferson fracture)
S12.100x002	Axis fracture
S12.100x003	Axis pedicle fracture [Hangman fracture]
S12.110	Open fracture of the second cervical vertebra
S12.200	Fracture of cervical vertebra, other specific
S12.200x001	Fracture of odontoid process of axis

S12.200x002	Axis fracture with dislocation (Hangman fracture)
S12.200x011	Cervical vertebra fracture C3
S12.200x021	Cervical vertebra fracture C4
S12.200x031	Cervical vertebra fracture C5
S12.200x041	Cervical vertebra fracture C6
S12.200x051	Cervical vertebra fracture C7
S12.210	Open fracture of cervical vertebra
S12.700	Multiple fractures of cervical vertebra
S12.700x001	Multiple fracture of cervical vertebra
S12.710	Open multiple cervical fractures
S12.800	Fractures in other parts of the neck
S12.800x001	Cricoid cartilage fracture
S12.800x002	Hyoid fracture
S12.800x003	Fracture of laryngeal cartilage
S12.800x004	Fracture of thyroid cartilage
S12.800x005	Fracture of cervical trachea cartilage
S12.801	Rupture of laryngeal cartilage
S12.802	Fracture of thyroid cartilage
S12.803	Hyoid fracture
S12.804	Cricoid cartilage rupture
S12.805	Fracture of trachea cartilage
S12.810	Open fracture of specific part of neck
S12.811	Open rupture of laryngeal cartilage
S12.812	Open rupture of thyroid cartilage
S12.813	Open hyoid fracture
S12.814	Open cricoid cartilage rupture
S12.815	Open trachea cartilage rupture
S12.900	Neck fracture
S12.900x001	Cervical vertebra fracture
S12.900x003	Fracture of cervical nerve arch
S12.900x004	Fracture of spinous process of cervical vertebra
S12.900x005	Fracture of cervical transverse process
S12.900x006	Fracture of cervical vertebra arch
S12.910	Open cervical fracture
S13.000	Traumatic rupture of cervical intervertebral disc
S13.000x001	Traumatic rupture of cervical intervertebral disc
S13.100	Cervical dislocation
S13.100x021	Cervical subluxation C2/C3
S13.100x022	Cervical dislocation C2/C3
S13.100x031	Cervical subluxation C3/C4
S13.100x032	Cervical dislocation C3/C4
S13.100x041	Cervical subluxation C4/C5

S13.100x042	Cervical dislocation C4/C5
S13.100x051	Cervical subluxation C5/C6
S13.100x052	Cervical dislocation C5/C6
S13.100x061	Cervical subluxation C6/C7
S13.100x062	Cervical dislocation C6/C7
S13.100x071	Cervicothoracic subluxation C7/T1
S13.100x072	Cervicothoracic dislocation C7/T1
S13.100x081	Atlanto occipital subluxation
S13.100x082	Dislocation of atlanto occipital joint
S13.101	Cervical subluxation
S13.102	Atlantoaxial subluxation
S13.103	Atlantoaxial dislocation
S13.104	Axis dislocation
S13.200	Dislocation of other and unspecified parts of the neck
S13.200x003	Dislocation of thyroid cartilage
S13.201	Cervical dislocation
S13.202	Dislocation of cricoarytenoid joint
S13.203	Dislocation of cricothyroid cartilage joint
S13.300	Multiple dislocation of neck
S13.400	Sprain and strain of cervical vertebra
S13.400x003	Sprain of anterior longitudinal ligament of neck
S13.400x005	Sprain of atlanto occipital joint
S13.400x006	Cervical joint locking
S13.401	Whiplash wound
S13.402	Sprain of cervical ligament
S13.403	Sprain of atlantoaxial joint
S13.500	Sprain and strain in thyroid region
S13.500x003	Sprain of cricoarytenoid ligament
S13.500x004	Sprain of cricothyroid joint
S13.500x005	Sprain of cricoid ligament
S13.501	Sprain of thyroid cartilage
S13.502	Sprain of cricoarytenoid joint
S13.600	Sprain and strain of joints and ligaments in other and unspecified parts of the neck
S13.601	Neck sprain
S14.000	Concussion and edema of cervical spinal cord
S14.001	Cervical spinal cord edema
S14.002	Cervical spinal cord concussion
S14.100	Other and unspecified injuries of cervical spinal cord
S14.100x011	Complete injury of cervical spinal cord

S14.100x021	Central cervical spinal cord injury syndrome
S14.100x022	Spinal cord central canal syndrome
S14.100x031	Cervical anterior cord syndrome
S14.100x032	Incomplete injury of cervical spinal cord
S14.100x033	Cervical posterior cord syndrome
S14.100x701	Cervical spinal cord function injury
S14.100x711	Cervical spinal cord function injury C1
S14.100x721	Cervical spinal cord function injury C2
S14.100x731	Cervical spinal cord function injury C3
S14.100x741	Cervical spinal cord function injury C4
S14.100x751	Cervical spinal cord function injury C5
S14.100x761	Cervical spinal cord function injury C6
S14.100x771	Cervical spinal cord function injury C7
S14.100x781	Cervicothoracic spinal cord function injury
S14.101	Cervical spinal cord injury
S14.200	Injury of Spinal Process Nerve Root of Cervical Spine
S14.200x001	Injury of cervical spinal nerve root
S14.300	Brachial plexus injury
S14.300x001	Injury of brachial plexus
S14.400	Injury of cervical peripheral nerves
S14.500	Cervical sympathetic nerve injury
S14.600	Injury of other and unspecified nerves in the neck
S14.601	Cervical nerve injury
S15.000	Carotid artery injury
S15.000x002	Traumatic carotid fistula
S15.000x011	Common carotid artery injury
S15.000x021	External carotid artery injury
S15.000x031	Injury of internal carotid artery
S15.001	Internal carotid artery laceration
S15.002	Common carotid artery laceration
S15.003	External carotid artery laceration
S15.004	Traumatic carotid aneurysm
S15.005	Traumatic carotid cavernous fistula
S15.100	Injury of vertebral artery
S15.200	External jugular vein injury
S15.300	Injury of internal jugular vein
S15.301	Rupture of internal jugular vein

S15.700	Multiple vascular injuries at the neck level
S15.800	Other vascular injuries at the neck level
S15.800x002	Traumatic vertebral arteriovenous fistula
S15.800x003	Traumatic thyroid vascular injury
S15.801	Traumatic carotid arteriovenous fistula
S15.900	Vascular injury at the neck level
S15.900x001	Neck vascular injury
S16.x00	Muscle and tendon injuries at the neck level
S16.x00x001	Neck muscle injury
S16.x00x002	Cervical tendon injury
S17.000	Crushing injury of larynx and trachea
S17.000x001	Crushing injury of larynx and trachea
S17.000x002	Laryngeal crush injury
S17.001	Crushing injury of trachea
S17.800	Crushing injury in other parts of the neck
S17.801	Crushing injury of throat
S17.900	Neck crush injury
S18.x00	Traumatic amputation at neck level
S18.x00x001	Behead
S19.700	Multiple injuries in the neck
S19.800	Other specific injuries to the neck
S19.800x002	Injury of cervical trachea
S19.800x004	Injury of cervical thoracic duct
S19.801	Laryngeal injury
S19.802	Laryngeal injury
S19.900	Neck injury
S20.000	Contusion of breast
S20.100	Other and unspecified superficial injuries of breast
S20.101	Superficial breast injury
S20.200	chest trauma
S20.200x003	Contusion of anterior sternum
S20.201	Contusion of chest wall
S20.202	Contusion of interscapular region
S20.300	Other superficial injuries of anterior chest wall
S20.300x001	Superficial injury of anterior chest wall
S20.301	Thoracic skin abrasion
S20.400	Other superficial injuries of posterior chest wall
S20.400x001	Superficial injury of posterior chest wall
S20.700	Multiple superficial chest injuries

S20.800	Superficial injuries to other and unspecified parts of the chest
S20.800x002	Superficial injury of anterior chest border
S20.801	Superficial injury of chest wall
S20.802	Superficial chest injury
S20.803	Scratch of chest wall
S21.000	Open wound of breast
S21.100	Open wound of chest wall
S21.100x002	Open injury of anterior sternum
S21.101	Open costal anterior wall injury
S21.200	Open wound of posterior chest wall
S21.200x001	Open injury of back
S21.200x002	External open injury of chest wall
S21.201	Open posterior chest wall injury
S21.202	Open posterior costal wall injury
S21.203	Open injury of interscapular region
S21.700	Multiple open wounds on chest wall
S21.800	Open wounds in other parts of the chest
S21.800x011	Open chest injury with fracture
S21.800x021	Open chest injury with dislocation
S21.800x031	Open chest injury with intrathoracic injury
S21.900	Open wound on chest
S21.900x001	Open injury of chest wall
S21.900x003	Traumatic foreign body in chest
S21.901	Open chest injury
S22.000	Thoracic vertebra fracture
S22.000x003	Compression fracture of thoracic vertebra
S22.000x005	Fracture of thoracic spinal nerve arch
S22.000x006	Thoracic spine process fracture
S22.000x007	Fracture of transverse process of thoracic vertebra
S22.000x009	Fracture of thoracic vertebral arch
S22.000x011	Thoracic vertebra fracture T1/T2
S22.000x021	Thoracic vertebra fracture T3/T4
S22.000x031	Thoracic vertebra fracture T5/T6
S22.000x041	Thoracic vertebra fracture T7/T8
S22.000x051	Thoracic vertebra fracture T9/T10
S22.000x061	Thoracic vertebra fracture T11/T12
S22.010	Open thoracic vertebral fracture
S22.100	Multiple fractures of thoracic vertebrae
S22.110	Open multiple thoracic vertebral fractures

S22.200	Sternal fracture
S22.210	Open sternal fracture
S22.300	Rib fracture
S22.300x011	Fracture of the first rib
S22.310	Open rib fracture
S22.400	Multiple fractures of rib
S22.400x011	Multiple fracture of rib with first rib fracture
S22.400x021	Two rib fractures without the first rib fracture
S22.400x031	Three rib fractures without first rib fracture
S22.400x041	More than four rib fractures without the first rib fracture
S22.410	Open multiple rib fractures
S22.500	Flail chest
S22.800	Bone fracture of other parts of the thorax
S22.810	Open fracture of specific thoracic region
S22.900	Bone fracture of thorax
S22.900x001	Thoracic fracture
S22.910	Open thoracic fracture
S23.000	Traumatic rupture of thoracic intervertebral disc
S23.100	Dislocation of thoracic vertebra
S23.100x011	Thoracic vertebra dislocation T1/T2
S23.100x012	Thoracic vertebra dislocation T2/T3
S23.100x021	Thoracic vertebra dislocation T3/T4
S23.100x022	Thoracic vertebra dislocation T4/T5
S23.100x031	Thoracic vertebra dislocation T5/T6
S23.100x032	Thoracic vertebra dislocation T6/T7
S23.100x041	Thoracic vertebra dislocation T7/T8
S23.100x042	Thoracic vertebra dislocation T8/T9
S23.100x051	Thoracic vertebra dislocation T9/T10
S23.100x052	Thoracic vertebra dislocation T10/T11
S23.100x061	Thoracic vertebra dislocation T11/T12
S23.100x071	Thoracolumbar dislocation T12/L1
S23.101	Traumatic thoracic disc herniation
S23.200	Dislocation of other and unspecified parts of the chest
S23.200x001	Dislocation of costal joint
S23.200x004	Thoracic tracheal dislocation
S23.200x005	Swordbone cartilage dislocation

S23.201	Dislocation of trachea
S23.202	Dislocation of costal cartilage
S23.203	Sternal dislocation
S23.300	Thoracic spine sprain and strain
S23.400	Rib and sternum sprain and strain
S23.401	Sternum sprain and strain
S23.500	Sprain and strain of other and unspecified parts of the chest
S23.501	Chest sprain
S24.000	Thoracic spinal cord concussion and edema
S24.000x002	Thoracic spinal cord concussion
S24.001	Thoracic spinal cord edema
S24.100	Other and unspecified injuries to the thoracic spinal cord
S24.100x011	Thoracic spinal cord injury
S24.100x021	Anterior funicular syndrome of thoracic spinal cord
S24.100x022	Central thoracic spinal cord injury syndrome
S24.100x023	Incomplete injury of thoracic spinal cord
S24.100x024	Thoracic posterior cord syndrome
S24.100x701	Thoracic spinal cord function injury
S24.100x711	Thoracic spinal cord function injury T1
S24.100x721	Thoracic spinal cord function injury T2/T3
S24.100x731	Thoracic spinal cord function injury T4/T5
S24.100x741	Thoracic spinal cord function injury T6/T7
S24.100x751	Thoracic spinal cord function injury T8/T9
S24.100x761	Thoracic spinal cord function injury T10/T11
S24.100x771	Thoracic spinal cord function injury T12
S24.101	Thoracic spinal cord injury
S24.200	Thoracic spinal nerve root injury
S24.300	Peripheral thoracic nerve injury
S24.300x001	Intercostal nerve injury
S24.400	Thoracic sympathetic nerve injury
S24.400x001	Injury of cardiac plexus
S24.400x002	Injury of esophageal plexus
S24.400x003	Injury of pulmonary plexus
S24.400x004	Stellate plexus injury
S24.400x005	Thoracic sympathetic ganglion injury
S24.500	Injuries to other nerves in the chest
S24.500x001	Phrenic nerve injury

S24.600	Injury of thoracic nerves
S25.000	Thoracic aorta injury
S25.001	Traumatic thoracic aortic aneurysm
S25.100	Injury of innominate artery or subclavian artery
S25.100x002	Innominate artery injury
S25.101	Injury of subclavian artery
S25.200	Injury of superior vena cava
S25.200x001	Vena cava injury
S25.201	Traumatic rupture of superior vena cava
S25.300	Injury of innominate vein or subclavian vein
S25.300x001	Injury of innominate vein
S25.301	Injury of subclavian vein
S25.400	Pulmonary vascular injury
S25.401	Traumatic pulmonary artery rupture
S25.500	Intercostal vascular injury
S25.501	Traumatic intercostal artery rupture
S25.700	Multiple vascular injuries in chest
S25.800	Injury of other blood vessels in the chest
S25.800x001	Breast artery injury
S25.800x003	Breast vein injury
S25.801	Azygos vein injury
S25.802	Traumatic rupture of breast artery
S25.900	Injury of thoracic blood vessels
S25.900x001	Thoracic vascular injury
S26.000	Cardiac injury with pericardium
S26.000x001	Traumatic pericardium
S26.000x002	Traumatic pericardial tamponade
S26.010	Open pericardium
S26.800	Other cardiac injuries
S26.800x011	Cardiac contusion
S26.800x021	Cardiac laceration
S26.800x031	Cardiac laceration with ventricular penetration
S26.800x082	Penetrating injury of heart
S26.800x083	Traumatic cardiac rupture
S26.801	Traumatic pericardial rupture
S26.810	Open heart specific injury
S26.811	Open heart penetrating injury
S26.812	Open heart rupture
S26.813	Cardiac foreign body
S26.900	Heart injury
S26.910	Open heart injury
S27.000	Traumatic pneumothorax

S27.010	Open pneumothorax
S27.100	traumatic haemothorax
S27.110	Open hemothorax
S27.200	Traumatic hemopneumothorax
S27.210	open pneumothorax
S27.300	Other lung injuries
S27.300x012	Pulmonary hematoma
S27.300x081	Foreign body in lung caused by trauma
S27.301	Pulmonary contusion
S27.302	Traumatic pulmonary rupture
S27.303	Traumatic rupture of pulmonary ligament
S27.310	Open lung specific injury
S27.311	Open pulmonary rupture
S27.312	Open pulmonary foreign body
S27.313	Penetrating lung injury
S27.400	Bronchial injury
S27.400x001	Rupture of main bronchus
S27.401	Traumatic bronchial rupture
S27.410	Open bronchial injury
S27.500	Thoracic trachea injury
S27.501	Traumatic thoracic tracheal rupture
S27.510	Open chest trachea injury
S27.600	Pleural injury
S27.610	Open pleural injury
S27.700	Multiple injuries of intrathoracic organs
S27.700x001	Multiple organ injuries in chest
S27.710	Open multiple injuries of intrathoracic organs
S27.800	Intrathoracic organ damage, other specific
S27.800x013	Traumatic mediastinal hematoma
S27.801	Scratch of esophageal mucosa
S27.802	Thoracic esophageal injury
S27.803	Cardiac injury
S27.804	Traumatic diaphragmatic rupture
S27.805	Traumatic diaphragmatic hernia
S27.806	Thoracic lymphatic injury
S27.807	Thymus injury
S27.808	Traumatic pleural effusion
S27.810	Open, especially intrathoracic organ injury
S27.811	Esophageal foreign body perforation
S27.812	Open diaphragmatic rupture
S27.900	Injury of intrathoracic organs
S27.910	Open thoracic foreign body
S28.000	Chest crush injury

S28.100	Partial traumatic thoracotomy
S29.000	Muscle and tendon injuries at the chest level
S29.000x001	Thoracic tendon injury
S29.000x002	Chest muscle injury
S29.700	Multiple chest injuries
S29.700x001	Traumatic rupture of thoracic duct
S29.800	Other specific injuries to the chest
S29.900	Chest injury
S30.000	Contusion of lower back and pelvis
S30.000x001	back contusion
S30.000x003	Lumbar contusion
S30.000x004	Contusion of sacral region
S30.001	Low back contusion
S30.002	Sacrococcygeal contusion
S30.003	Contusion of buttock
S30.100	Contusion of abdominal wall
S30.100x001	Abdominal contusion
S30.100x002	Contusion of flank
S30.100x004	Contusion of iliac region
S30.100x005	Contusion of inguinal region
S30.100x007	Hematoma of iliac fossa
S30.101	Traumatic iliac hematoma
S30.102	Traumatic iliopsoas hematoma
S30.104	Contusion of groin
S30.200	Contusion of external genitalia
S30.200x005	Contusion of perineum
S30.200x006	Contusion of vulva
S30.200x007	Contusion of labia majora
S30.200x008	Contusion of labia minora
S30.200x010	Traumatic hematoma of vulva
S30.201	Traumatic perineal hematoma
S30.202	Traumatic scrotal hematoma
S30.203	Traumatic epididymal hematoma
S30.205	Contusion of scrotum
S30.206	Penis contusion
S30.207	Vaginal contusion
S30.208	Testicular contusion
S30.700	Multiple superficial injuries of abdomen, lower back and pelvis
S30.800	Other superficial injuries to the abdomen, lower back and pelvis
S30.800x001	Superficial injury of hip
S30.800x002	Superficial injury of upper abdomen
S30.800x003	Superficial injury of external genitalia
S30.800x004	Superficial injury of flank

S30.801	Superficial foreign body of abdominal wall
S30.900	Superficial injury of abdomen, lower back and pelvis
S30.900x001	Superficial abdominal injury
S30.900x002	Superficial injury of lower back
S30.900x003	Superficial injury of pelvis
S31.000	Open wound of lower back and pelvis
S31.000x003	Open injury of sacrum
S31.000x004	Open injury of pelvis
S31.000x005	Open injury of lower back
S31.000x006	Open injury of buttock with foreign body
S31.001	Traumatic laceration of perineum
S31.002	Skin avulsion injury of lumbar and back
S31.003	Open perineal injury
S31.004	Open hip injury
S31.005	Open lumbar and back injury
S31.006	Foreign body in buttock
S31.100	Open wound of abdominal wall
S31.100x002	Open injury of upper abdomen
S31.100x003	Open injury of flank
S31.100x005	Open injury of iliac region
S31.100x006	Open injury of inguinal region
S31.100x007	Open injury of vulva
S31.101	Open costal injury
S31.102	Open inguinal injury
S31.200	Open wound of penis
S31.300	Open wound of scrotum and testis
S31.300x001	Open injury of scrotum
S31.300x002	Testicular rupture
S31.301	Open testicular injury
S31.400	Open wound of vagina and vulva
S31.400x001	Open injury of vagina
S31.400x002	Open injury of clitoris
S31.400x003	Open injury of vulva
S31.401	Traumatic laceration of vulva
S31.402	Hymen laceration
S31.500	Open wound of external genitalia, other and unspecified
S31.501	Open external genital injury
S31.700	Multiple open wounds in abdomen, lower back and pelvis
S31.800	Open wounds in other and unspecified parts of the abdomen
S31.800x003	Traumatic abdominal foreign body
S31.800x011	Open injury of lower back with fracture

S31.800x012	Open injury of pelvis with fracture
S31.800x021	Open injury of lower back with dislocation
S31.800x022	Open injury of pelvis with dislocation
S31.800x031	Open abdominal injury with intra-abdominal organ injury
S31.801	Open abdominal injury
S31.802	Open abdominal foreign body
S31.803	Penetrating wound of vagina and rectum
S31.804	Traumatic laceration of anal sphincter
S31.805	Traumatic anal laceration
S32.000	Lumbar fracture
S32.000x002	Lumbar compression fracture
S32.000x011	Lumbar fracture L1
S32.000x021	Lumbar fracture L2
S32.000x031	Lumbar fracture L3
S32.000x041	Lumbar fracture L4
S32.000x051	Lumbar fracture L5
S32.010	Open lumbar fracture
S32.100	Sacral fracture
S32.110	Open sacral fracture
S32.200	Coccyx fracture
S32.210	Open coccyx fracture
S32.300	Iliac fracture
S32.310	Open iliac fracture
S32.400	Acetabular fracture
S32.410	Open acetabular fracture
S32.500	Pubic fracture
S32.500x002	Fracture of pubic branch
S32.500x003	Fracture of pubic symphysis
S32.510	Open pubic fracture
S32.700	Multiple fractures of lumbar spine and pelvis
S32.701	Multiple pelvic fractures
S32.702	Multiple lumbar fractures
S32.710	Open multiple fractures of lumbar spine and pelvis
S32.711	Open multiple pelvic fractures
S32.712	Open multiple lumbar fractures
S32.800	Fractures of the lumbar spine and other unspecified parts of the pelvis
S32.800x021	Lumbosacral spinous process fracture
S32.800x022	Fracture of lumbosacral transverse process
S32.800x023	Fracture of lumbosacral arch
S32.800x024	Lumbosacral vertebra fracture

S32.800x091	Fracture of pelvic joint
S32.800x092	Lateral compression fracture of pelvis
S32.800x093	Open book fracture of pelvis
S32.800x094	Vertical shear fracture of pelvis
S32.800x095	Malgeney fracture
S32.801	Ischial fracture
S32.802	Pelvic fracture
S32.803	Lumbosacral spinal fracture
S32.810	Open fracture of lumbar spine and pelvis at specific points
S32.811	Open ischial fracture
S32.812	open pelvic fracture
S32.813	Open fracture of lumbosacral spine
S33.000	Traumatic rupture of lumbar intervertebral disc
S33.100	Lumbar dislocation
S33.100x011	Lumbar dislocation L1/L2
S33.100x021	Lumbar dislocation L2/L3
S33.100x031	Lumbar dislocation L3/L4
S33.100x041	Lumbar dislocation L4/L5
S33.100x051	Lumbosacral dislocation L5/S1
S33.200	Dislocation of sacroiliac joint and sacrococcygeal joint
S33.200x001	Dislocation of coccyx
S33.200x002	Dislocation of sacrum
S33.200x003	Dislocation of sacroiliac joint
S33.201	Dislocation of sacrococcygeal joint
S33.300	Dislocation of the lumbar spine and other unspecified parts of the pelvis
S33.300x001	Dislocation of pubic symphysis
S33.300x004	Dislocation of pelvis
S33.301	Dislocation of lumbar spine and pelvis
S33.400	Traumatic rupture of pubic symphysis
S33.400x001	Traumatic rupture of pubic symphysis
S33.500	Lumbar sprain and strain
S33.500x011	Lumbosacral joint sprain
S33.501	Lumbar sprain
S33.502	Lumbar sprain
S33.600	Sprain and strain of sacroiliac joint
S33.600x001	Sprain of sacroiliac joint
S33.601	Sprain of sacral joint
S33.700	Sprain and strain of lumbar spine and other unspecified parts of pelvis
S33.700x001	Sacrococcygeal ligament strain
S33.700x002	Injury of supraspinous ligament

S33.700x003	Lumbar joint sprain
S33.701	Sprain of sacroiliac region
S33.702	Sprain of pubic symphysis
S33.703	Sprain and strain of lumbar spine and pelvis
S34.000	Lumbar spinal cord concussion and edema
S34.000x002	Lumbar spinal cord concussion
S34.001	Lumbar spinal cord edema
S34.100	Other injuries of lumbar spinal cord
S34.100x001	Lumbar spinal cord injury
S34.100x002	Complete injury of lumbar spinal cord
S34.100x003	Incomplete injury of lumbar spinal cord
S34.100x701	Lumbar spinal cord function injury
S34.100x711	Lumbar spinal cord function injury L1
S34.100x721	Lumbar spinal cord function injury L2
S34.100x731	Lumbar spinal cord function injury L3
S34.100x741	Lumbar spinal cord function injury L4
S34.100x751	Lumbar spinal cord function injury L5
S34.100x761	Sacral spinal cord function injury
S34.200	Injury of lumbosacral nerve root
S34.200x001	Sacral spinal nerve root injury
S34.200x002	Injury of lumbar spinal nerve root
S34.300	Cauda equina injury
S34.400	Lumbosacral plexus injury
S34.500	Waist, sacrum and pelvic sympathetic nerve injury
S34.500x001	Abdominal sympathetic ganglion injury
S34.500x004	Sympathetic nerve injury of inferior mesenteric plexus
S34.500x005	Sympathetic nerve injury of superior mesenteric plexus
S34.500x007	Waist, sacrum and pelvic sympathetic nerve injury
S34.501	Celiac plexus injury
S34.502	Hypogastric plexus injury
S34.503	Mesenteric plexus injury
S34.504	Visceral nerve injury
S34.600	Abdominal, lower back and pelvic peripheral nerve injuries
S34.601	Injury of lower dorsal peripheral nerve
S34.602	Peripheral pelvic nerve injury
S34.800	Other and unspecified nerve injuries at the abdominal, lower back and pelvic levels

S34.800x001	Lumbosacral nerve injury
S34.801	Abdominal nerve injury
S34.802	Injury of inferior dorsal nerve
S34.803	Pelvic nerve injury
S35.000	Abdominal aorta injury
S35.001	Traumatic abdominal aortic aneurysm
S35.100	Injury of inferior vena cava
S35.100x003	Hepatic vein injury
S35.101	Traumatic rupture of inferior vena cava
S35.102	Traumatic rupture of hepatic vein
S35.200	Celiac or mesenteric artery injury
S35.200x001	Celiac artery injury
S35.200x003	Gastroduodenal artery injury
S35.200x004	Hepatic artery injury
S35.200x005	Injury of inferior mesenteric artery
S35.200x006	Injury of superior mesenteric artery
S35.200x007	Splenic artery injury
S35.201	Mesenteric artery injury
S35.202	Gastric artery injury
S35.203	Traumatic rupture of gastric artery
S35.204	Traumatic rupture of hepatic artery
S35.205	Traumatic rupture of splenic artery
S35.300	Injury of portal vein or splenic vein
S35.300x001	Portal vein injury
S35.300x002	Splenic vein injury
S35.300x003	Injury of inferior mesenteric vein
S35.300x004	Injury of superior mesenteric vein
S35.300x005	Mesenteric vein injury
S35.301	Traumatic mesenteric vein rupture
S35.302	Traumatic rupture of splenic vein
S35.400	Renal vascular injury
S35.400x001	Renal artery injury
S35.400x002	Renal vein injury
S35.401	Traumatic rupture of renal vein
S35.402	Traumatic rupture of renal artery
S35.500	Injury of iliac vessels
S35.500x001	Injury of iliac artery
S35.500x002	Traumatic thrombosis of common iliac artery
S35.500x003	Traumatic iliac arteriovenous fistula
S35.500x004	Injury of iliac vein

S35.500x005	Uterine artery injury
S35.500x006	Injury of uterine vein
S35.500x007	Injury of inferior abdominal artery
S35.500x008	Injury of inferior abdominal vein
S35.501	Traumatic rupture of iliac artery
S35.502	Traumatic rupture of iliac vein
S35.503	Traumatic uterine arteriovenous rupture
S35.700	Multiple vascular injuries at the abdominal, lower back and pelvic levels
S35.700x001	Multiple vascular injuries in abdomen, lower back and pelvis
S35.700x003	Injury of presacral venous plexus
S35.700x004	Mesenteric vascular injury
S35.701	Multiple abdominal vascular injuries
S35.800	Other vascular injuries at the abdominal, lower back and pelvic levels
S35.800x001	Ovarian artery injury
S35.800x002	Injury of ovarian vein
S35.801	Ovarian arteriovenous injury
S35.900	Injury of blood vessels at the abdominal, lower back and pelvic levels
S35.900x001	Abdominal vascular injury
S35.901	Injury of inferior dorsal vessels
S35.902	Pelvic vascular injury
S35.903	Traumatic mesenteric vascular injury
S36.000	Splenic injury
S36.000x021	Tear of splenic capsule
S36.000x031	Spleen laceration with soft tissue injury
S36.000x081	Penetrating injury of spleen
S36.001	Traumatic splenic hematoma
S36.002	Traumatic rupture of spleen
S36.011	Open rupture of spleen
S36.100	Liver or gallbladder injury
S36.100x001	Liver injury
S36.100x011	Hepatic contusion
S36.100x013	Traumatic hepatic hematoma
S36.100x021	Laceration of liver
S36.100x031	Mild laceration of liver
S36.100x041	Moderate laceration of liver
S36.100x051	Severe laceration of liver
S36.100x081	Bile duct injury
S36.101	Gallbladder injury
S36.102	Traumatic liver rupture

S36.103	Traumatic rupture of common bile duct
S36.110	Open rupture of liver
S36.111	Open gallbladder injury
S36.112	Open bile duct injury
S36.113	Open common bile duct injury
S36.200	Pancreatic injury
S36.200x001	Pancreatic injury
S36.200x011	Pancreatic head injury
S36.200x021	Pancreatic body injury
S36.200x031	Pancreatic tail injury
S36.200x091	Pancreatic duct injury
S36.200x092	Injury of pancreas and pancreatic duct
S36.201	Traumatic pancreatic rupture
S36.202	Tear of pancreatic capsule
S36.210	Open pancreatic injury
S36.300	Gastric injury
S36.301	Traumatic gastric rupture
S36.310	Open gastric rupture
S36.400	Small intestine injury
S36.400x091	Jejunum injury
S36.400x093	Ileal injury
S36.400x095	Multiple injuries of small intestine
S36.401	Traumatic rupture of duodenum
S36.402	Traumatic jejunal rupture
S36.403	Traumatic rupture of ileum
S36.404	Traumatic rupture of small intestine
S36.405	injury of duodenum
S36.411	Open rupture of small intestine
S36.412	Open rupture of duodenum
S36.413	Open jejunal rupture
S36.414	Open ileal rupture
S36.500	Colon injury
S36.500x011	Ascending colon injury
S36.500x021	Transverse colon injury
S36.500x031	Descending colon injury
S36.500x041	Sigmoid injury
S36.500x091	Multiple injuries of colon
S36.500x092	Appendiceal injury
S36.500x093	Cecal injury
S36.501	Traumatic rupture of colon
S36.511	Open rupture of colon
S36.600	Rectal injury
S36.600x003	Multiple rectal injuries
S36.601	Traumatic rectal rupture
S36.611	Open rectal rupture

S36.700	Multiple intra-abdominal organ injuries
S36.701	Traumatic intra-abdominal multiple organ rupture
S36.800	Intraabdominal organ damage, others
S36.800x022	mesenteric injury
S36.801	Peritoneal injury
S36.802	Mesenteric laceration
S36.803	Traumatic retroperitoneal hematoma
S36.810	Open, especially intra-abdominal organ injury
S36.811	Open mesenteric hematoma
S36.812	Open mesenteric laceration
S36.813	Open retroperitoneal hematoma
S36.814	Open rupture of greater omentum
S36.900	Injury of intra-abdominal organs
S36.900x001	Intra-abdominal organ injury
S36.901	Traumatic intestinal rupture
S36.910	Open intra-abdominal organ injury
S37.000	Renal injury
S37.000x012	Contusion of renal capsule
S37.000x013	Contusion of renal pelvis
S37.000x015	Hematoma of renal cyst
S37.000x016	Hematoma of renal pelvis
S37.000x021	Renal laceration
S37.000x022	Rupture of renal capsule
S37.000x023	Laceration of renal pelvis
S37.000x031	Renal comminuted injury
S37.000x041	Renal pedicle injury
S37.001	Traumatic renal rupture
S37.002	Renal contusion
S37.003	Traumatic renal hematoma
S37.004	Traumatic perirenal hematoma
S37.010	Open renal injury
S37.011	Open renal rupture
S37.100	Ureteral injury
S37.101	Traumatic rupture of ureter
S37.111	Open ureteral rupture
S37.200	Bladder injury
S37.200x011	Contusion of bladder
S37.200x022	Extraperitoneal bladder rupture
S37.200x023	Rupture of intraperitoneal bladder
S37.200x024	Mixed bladder rupture
S37.200x081	Laceration of bladder
S37.201	Traumatic bladder rupture
S37.211	Open bladder rupture
S37.300	Urethral injury

S37.300x004	Complete urethral rupture
S37.300x005	Partial urethral rupture
S37.300x011	Injury of urethral membrane
S37.300x021	Injury of urethral cavernous body
S37.300x031	Injury of urethra and prostate
S37.300x081	Rupture of urethral bulb
S37.300x082	Contusion and laceration of urethral bulb
S37.300x083	Posterior urethral injury
S37.301	Traumatic urethral rupture
S37.302	Urethral contusion
S37.303	Urethral injury with stricture
S37.310	Open urethral injury
S37.400	Ovarian injury
S37.410	Open ovarian injury
S37.500	Fallopian tube injury
S37.510	Open tubal injury
S37.600	Uterine injury
S37.600x002	Traumatic rupture of uterus
S37.601	Traumatic cervical laceration
S37.602	Traumatic uterine perforation
S37.610	Open uterine injury
S37.700	Multiple pelvic organ injuries
S37.710	Open multiple pelvic organ injury
S37.800	Pelvic organ damage, others
S37.801	Vasectomy injury
S37.802	Seminal vesicle injury
S37.803	Adrenal injury
S37.804	Prostate injury
S37.810	Open, especially pelvic organ injury
S37.811	Open injury of vas deferens
S37.812	Open seminal vesicle injury
S37.813	Open adrenal injury
S37.814	Open prostate injury
S37.900	Injury of pelvic organs
S37.910	Open pelvic organ injury
S38.000	Crushing injury of external genitalia
S38.000x001	External genitalia thermal crush injury
S38.000x002	Thermal crush injury of penis
S38.001	Crush wound of penis
S38.100	Crushing injury of abdomen, lower back and other unspecified parts of pelvis
S38.100x002	Abdominal crush injury
S38.100x003	Crushing injury of lower back
S38.100x004	Pelvic crush injury

S38.100x011	Thermal crush injury of abdomen, lower back and pelvis
S38.101	Crushing injury of abdomen, lower back and pelvis
S38.200	Traumatic amputation of external genitalia
S38.200x001	Labia majora amputation
S38.200x002	Labia minora amputation
S38.200x003	Penile amputation
S38.200x004	Scrotal amputation
S38.200x005	Testicular amputation
S38.200x006	Vulva amputation
S38.300	Traumatic amputation of abdomen, lower back and other unspecified parts of pelvis
S38.300x001	Torso amputation
S38.300x002	Abdominal amputation
S38.301	Traumatic thoracotomy
S38.302	Traumatic lower dorsal amputation
S38.303	Traumatic pelvic amputation
S39.000	Abdominal, lower back and pelvic muscle and tendon injuries
S39.000x001	Abdominal muscle injury
S39.000x002	Abdominal tendon injury
S39.000x003	Lower back muscle injury
S39.000x004	Injury of tendon of inferior dorsal muscle
S39.000x005	Pelvic muscle injury
S39.000x006	Pelvic tendon injury
S39.001	Rupture of rectus abdominis
S39.002	Open rupture of psoas major muscle
S39.003	Lower back soft tissue injury
S39.004	Pelvic soft tissue injury
S39.600	Abdominal organs accompanied by pelvic organ damage
S39.600x001	Abdominal organs with pelvic organ damage
S39.700	Multiple injuries in abdomen, lower back and pelvis
S39.800	Other specific injuries to the abdomen, lower back and pelvis
S39.800x001	Abdominal soft tissue injury
S39.800x002	Lower back soft tissue injury
S39.800x004	Soft tissue injury of abdomen, lower back and pelvis
S39.900	Abdominal, lower back and pelvis injuries
S39.900x002	Lower back injury
S39.900x004	Inguinal injury
S39.900x007	Scrotal injury
S39.900x009	Epididymal injury

S39.900x010	Testicular injury
S39.901	Hymen damage
S39.902	Vaginal injury
S39.903	Perineal injury
S39.904	Penis injury
S39.905	Anal injury
S39.906	Lumbar injury
S39.907	Abdominal injury
S39.908	Pelvic injury
S39.909	Metallic foreign body in abdomen
S39.910	Lumbar soft tissue injury
S39.911	Abdominal soft tissue injury
S39.912	Hip soft tissue injury
S40.000	Contusion of shoulder and upper arm
S40.000x001	Contusion of shoulder
S40.000x002	Contusion of scapular region
S40.000x003	Contusion of axillary region
S40.001	Upper arm contusion
S40.700	Multiple superficial injuries of shoulder and upper arm
S40.701	Multiple contusions of shoulder and arm
S40.800	Other superficial injuries of shoulder and upper arm
S40.800x011	Shoulder scratch
S40.800x012	Upper arm abrasions
S40.800x021	Shoulder blister
S40.800x022	Vesicle of upper arm
S40.800x031	Shoulder insect bite
S40.800x032	Upper armworm bite
S40.800x041	Superficial foreign body of shoulder
S40.800x042	Foreign body on the surface of upper arm
S40.900	Superficial injury of shoulder and upper arm
S41.000	Open wound of shoulder
S41.000x002	Open injury of scapular band
S41.100	Open wound of upper arm
S41.700	Multiple open wounds on shoulder and upper arm
S41.800	Open wound of other and unspecified parts of scapular band
S41.800x001	Open injury of axilla
S41.800x011	Open injury of shoulder with fracture
S41.800x012	Open injury of upper arm with fracture
S41.800x021	Open injury of shoulder with dislocation

S41.800x022	Open injury of upper arm with dislocation
S41.801	Open injury of scapular region
S41.802	Open shoulder belt injury
S42.000	Clavicle fracture
S42.000x011	Fracture of sternal end of clavicle
S42.000x021	Fracture of clavicular shaft
S42.000x031	Fracture of acromion end of clavicle
S42.000x091	Multiple fracture of clavicle
S42.010	Open clavicular fracture
S42.100	Scapular fracture
S42.100x011	Fracture of scapular body
S42.100x021	Acromion fracture
S42.100x031	Fracture of coracoid process of scapula
S42.100x041	Fracture of scapular neck and glenoid
S42.100x042	Glenoid fracture of shoulder
S42.100x091	Multiple fractures of scapula
S42.110	Open scapular fracture
S42.200	Fracture of upper humerus
S42.200x001	proximal humeral fractures
S42.200x011	Separation of proximal humeral epiphysis
S42.200x031	Fracture of anatomical neck of humerus
S42.200x041	Fracture of greater tubercle of humerus
S42.200x091	Fracture of humeral tubercle
S42.200x092	Multiple fractures of proximal humerus
S42.200x101	Fracture of humeral neck
S42.202	Fracture of surgical neck of humerus
S42.203	Fracture of humeral head
S42.210	Open fracture of upper humerus
S42.300	Fracture of humeral shaft
S42.300x002	Multiple fractures of humeral shaft
S42.301	Fracture of humerus
S42.310	Open fracture of humeral shaft
S42.311	Open fracture of humerus
S42.400	Fracture of lower end of humerus
S42.400x001	Fracture of distal humerus
S42.400x041	Fracture of medial epicondyle of humerus
S42.400x042	Fracture of lateral epicondyle of humerus
S42.400x043	Separation of distal humeral epiphysis
S42.400x051	T-shaped fracture of distal humerus
S42.400x091	Multiple fractures of distal humerus

S42.400x092	Fracture of trochlear of humerus
S42.400x093	Fracture of capitulum of humerus
S42.401	Supracondylar fracture of humerus
S42.402	Fracture of lateral condyle of humerus
S42.403	Intercondylar fracture of humerus
S42.404	Fracture of medial condyle of humerus
S42.410	Open fracture of lower humerus
S42.700	Multiple fractures of clavicle, scapula and humerus
S42.710	Open multiple fractures of clavicle, scapula and humerus
S42.800	Fracture of shoulder and other parts of upper arm
S42.810	Open fracture of shoulder and upper arm
S42.900	Fracture of scapular band
S42.910	Open shoulder fracture
S43.000	Dislocation of shoulder joint
S43.000x011	Anterior dislocation of humerus
S43.000x021	Posterior dislocation of humerus
S43.000x031	Subluxation of humerus
S43.000x041	Anterior dislocation of shoulder joint
S43.000x042	Posterior dislocation of shoulder joint
S43.001	shoulder subluxation
S43.002	Dislocation of glenohumeral joint
S43.100	Acromioclavicular joint dislocation
S43.200	Dislocation of sternoclavicular joint
S43.300	Dislocation of other and unspecified parts of scapular band
S43.301	Dislocation of scapula
S43.302	Dislocation of scapular band
S43.400	Sprain and strain of shoulder joint
S43.400x001	Sprain of shoulder joint
S43.400x003	Sprain of rotator cuff joint capsule
S43.400x004	Coracohumeral ligament sprain
S43.400x011	Tear of shoulder joint capsule
S43.400x012	Injury of anterior and posterior scapular glenoid (SLAP injury)
S43.400x013	Anterior and inferior injury of scapular glenoid (bankart injury)
S43.401	Strain of shoulder joint
S43.500	Sprain and strain of acromioclavicular joint
S43.500x001	Sprain of acromioclavicular joint

S43.500x002	Sprain of acromioclavicular ligament
S43.501	Injury of acromioclavicular ligament
S43.600	Thoracoclavicular joint sprain and strain
S43.601	Thoracoclavicular joint sprain
S43.700	Sprain and strain of other and unspecified parts of shoulder girdle
S43.700x002	Injury of glenoid lip of shoulder joint
S43.700x011	Sprain and strain in other parts of shoulder girdle
S43.701	Sprain of scapular band
S44.000	Ulnar nerve injury at upper arm level
S44.000x001	Ulnar nerve injury of upper arm
S44.100	Median nerve injury at upper arm level
S44.100x001	Injury of median nerve of upper arm
S44.101	Rupture of median nerve of upper arm
S44.200	Radial nerve injury at upper arm level
S44.200x001	Injury of radial nerve of upper arm
S44.300	Axillary nerve injury
S44.400	Myocutaneous nerve injury
S44.500	Dermatosensory nerve injury at shoulder and upper arm level
S44.500x001	Injury of medial arm cutaneous nerve
S44.500x002	Injury of medial forearm cutaneous nerve
S44.501	Upper arm cutaneous sensory nerve injury
S44.700	Multiple nerve injuries at the shoulder and upper arm levels
S44.700x001	Multiple nerve injuries of shoulder and upper arm
S44.701	Multiple nerve injuries of upper arm
S44.800	Other nerve injuries at the shoulder and upper arm levels
S44.800x001	Injury of suprascapular nerve
S44.900	Nerve injury at shoulder and upper arm level
S44.900x001	Nerve injury of shoulder and upper arm
S44.901	Upper arm nerve injury
S45.000	Axillary artery injury
S45.001	Traumatic rupture of axillary artery
S45.100	Injury of brachial artery
S45.101	Traumatic brachial artery injury
S45.200	Injury of axillary or brachial vein

S45.200x002	Injury of brachial vein
S45.201	Traumatic injury of axillary vein
S45.300	Superficial vein injury at shoulder and upper arm level
S45.300x001	Superficial vein injury of shoulder and upper arm
S45.300x002	Injury of superficial vein of shoulder
S45.301	Superficial vein injury of upper arm
S45.700	Multiple vascular injuries at the shoulder and upper arm levels
S45.700x001	Multiple vascular injuries of shoulder and upper arm
S45.701	Multiple vascular injuries of upper arm
S45.800	Other vascular injuries at the shoulder and upper arm levels
S45.900	Injury of blood vessels at the shoulder and upper arm levels
S45.900x001	Vascular injury of shoulder and upper arm
S46.000	Injury of rotator cuff tendon of shoulder
S46.002	Shoulder sleeve damage
S46.100	Injury of biceps longus muscle and tendon
S46.100x001	Injury of long head of biceps brachii
S46.100x002	Injury of tendon of long head of biceps brachii
S46.100x003	Injury of long head muscle and tendon of biceps brachii
S46.101	Tendon injury of biceps longus
S46.200	Muscle and tendon injuries in other parts of biceps
S46.200x001	Biceps brachii muscle injury
S46.200x002	Injury of biceps tendon
S46.200x003	Injury of biceps muscle and tendon
S46.201	Traumatic rupture of biceps brachii
S46.300	Injury of triceps muscle and tendon
S46.300x001	Muscle injury of triceps brachii
S46.300x002	Injury of triceps tendon
S46.300x003	Injury of triceps muscle and tendon
S46.301	Traumatic rupture of triceps brachii
S46.700	Multiple muscle and tendon injuries at the shoulder and upper arm levels
S46.700x001	Multiple muscle injuries of shoulder and upper arm
S46.700x002	Multiple tendon injuries of shoulder and upper arm
S46.701	Multiple tendon injuries of shoulder

S46.702	Multiple tendon injuries of upper arm
S46.800	Other muscle and tendon injuries at the shoulder and upper arm levels
S46.800x001	Deltoid injury
S46.800x002	Injury of supraspinatus muscle
S46.800x003	Injury of supraspinatus tendon
S46.800x004	Injury of infraspinatus muscle
S46.800x005	Injury of infraspinatus tendon
S46.800x006	Injury of subscapularis muscle
S46.800x007	Injury of subscapularis tendon
S46.801	Traumatic rupture of supraspinatus muscle
S46.802	Traumatic rupture of deltoid muscle
S46.900	Injuries to muscles and tendons at the shoulder and upper arm levels
S46.900x001	Muscle injury of shoulder and upper arm
S46.900x002	Injury of shoulder and upper arm tendons
S47.x00	Crushing injury of shoulder and upper arm
S47.x00x002	Crushing injury of shoulder
S47.x00x011	Shoulder thermal crush injury
S47.x00x012	Upper arm thermal crush injury
S47.x01	Crushing injury of upper arm
S48.000	Traumatic amputation at shoulder joint
S48.000x001	Amputation of shoulder joint
S48.100	Traumatic amputation at the level between shoulder and elbow
S48.100x001	Upper arm cutoff
S48.900	Traumatic amputation at shoulder and upper arm level
S49.700	Multiple injuries of shoulder and upper arm
S49.800	Other specific injuries of shoulder and upper arm
S49.900	Shoulder and upper arm injuries
S49.900x001	shoulder injury
S49.901	Upper arm injury
S50.000	Contusion of elbow
S50.100	Contusion of other and unspecified parts of forearm
S50.101	Forearm contusion
S50.700	Multiple superficial injuries of forearm
S50.701	Multiple abrasions of forearm
S50.800	Other superficial injuries of forearm
S50.800x011	Forearm abrasion
S50.800x021	Vesicle of forearm
S50.800x031	Insect bite of forearm

S50.800x041	Forearm superficial foreign body
S50.800x081	Superficial injury of elbow joint
S50.900	Superficial injury of forearm
S50.901	Superficial injury of elbow
S51.000	Open wound of elbow
S51.700	Multiple open wounds on forearm
S51.800	Open wounds in other parts of the forearm
S51.800x011	Open injury of forearm with fracture
S51.800x021	Open injury of forearm with dislocation
S51.900	Open wound of forearm
S51.901	Open forearm injury
S52.000	Fracture of upper end of ulna
S52.000x001	Fracture of elbow joint
S52.000x002	Fracture of proximal ulna
S52.000x011	Fracture of olecranon of ulna
S52.000x012	Separation of olecranon epiphysis of ulna
S52.000x021	Fracture of coronoid process of ulna
S52.000x091	Multiple fractures of proximal ulna
S52.001	Olecranon fracture
S52.002	Monteggia fracture dislocation
S52.010	Open fracture of upper end of ulna
S52.011	Open fracture of olecranon
S52.100	Fracture of upper radius
S52.100x001	Fracture of proximal radius
S52.100x002	Separation of proximal radius epiphysis
S52.100x012	Separation of epiphysis of radial head
S52.100x091	Multiple fractures of proximal radius
S52.101	Fracture of radial head
S52.102	Fracture of radial neck
S52.110	Open fracture of upper radius
S52.200	Fracture of ulnar shaft
S52.200x011	Monteggia fracture
S52.201	Ulnar fracture
S52.210	Open fracture of ulnar shaft
S52.211	Open fracture of ulna
S52.300	Fracture of radial shaft
S52.300x011	Gai's fracture
S52.310	Open fracture of radial shaft
S52.400	Fracture of both ulna and radius diaphysis
S52.400x001	Fracture of shaft of radius and ulna
S52.410	Open fracture of ulna and radius shaft
S52.500	Fracture of lower end of radius
S52.500x001	Fracture of distal radius
S52.500x002	Fracture of styloid process of radius
S52.500x003	Separation of distal radius epiphysis
S52.500x011	Colles Fracture
S52.500x021	Barton fracture
S52.500x022	Smith fracture
S52.500x091	Intraarticular fracture of radius
S52.501	Flexion fracture of distal radius
S52.502	Straight fracture of lower radius
S52.510	Open fracture of distal radius
S52.600	Both ulna and lower radius fractures
S52.600x001	Distal ulna fracture with distal radius fracture
S52.600x002	Ulnar styloid process fracture with distal radius fracture
S52.610	Open fracture of distal radius of ulna
S52.700	Multiple fractures of forearm
S52.701	Closed fracture of radius and ulna
S52.710	Open multiple fractures of forearm
S52.711	Open fracture of radius and ulna
S52.800	Fracture of other parts of forearm
S52.800x002	Separation of distal ulnar epiphysis
S52.801	Fracture of radius
S52.802	Fracture of styloid process of ulna
S52.803	Fracture of ulnar head
S52.804	Fracture of lower end of ulna
S52.810	Open fracture of forearm
S52.811	Open fracture of radius
S52.812	Open fracture of styloid process of ulna
S52.813	Open fracture of ulnar head
S52.814	Open fracture of lower end of ulna
S52.900	Fracture of forearm
S53.000	Dislocation of radial head
S53.000x003	Subluxation of radial head
S53.001	Dislocation of radiohumeral joint
S53.002	Dislocation of radioulnar joint
S53.100	Dislocation of elbow joint
S53.100x011	Anterior dislocation of elbow joint
S53.100x021	Posterior dislocation of elbow joint

S53.100x031	Internal dislocation of elbow joint
S53.100x041	Lateral dislocation of elbow joint
S53.101	Ulnar humeral joint dislocation
S53.102	Dislocation of ulnar head
S53.200	Traumatic rupture of radial collateral ligament
S53.200x001	Rupture of radial collateral ligament
S53.300	Traumatic rupture of ulnar collateral ligament
S53.300x001	Rupture of ulnar collateral ligament
S53.400	Elbow sprain and strain
S53.400x012	Sprain of radial collateral ligament
S53.400x021	Sprain of ulnar collateral ligament
S53.400x031	Sprain of radiohumeral joint
S53.400x041	Ulnar humeral joint sprain
S53.401	Sprain of annular ligament of radius
S53.402	Elbow sprain
S54.000	Ulnar nerve injury at forearm level
S54.000x001	Ulnar nerve injury of forearm
S54.001	Rupture of ulnar nerve of forearm
S54.100	Median nerve injury at forearm level
S54.100x001	Median nerve injury of forearm
S54.101	Fracture of median nerve of forearm
S54.200	Radial nerve injury at forearm level
S54.200x001	Injury of radial nerve of forearm
S54.300	Dermatosensory nerve injury at forearm level
S54.300x001	Injury of cutaneous sensory nerve of forearm
S54.700	Multiple nerve injuries at forearm level
S54.700x001	Multiple nerve injuries of forearm
S54.800	Other nerve injuries at forearm level
S54.900	Nerve injury at forearm level
S54.900x001	Forearm nerve injury
S55.000	Ulnar artery injury at forearm level
S55.000x001	Ulnar artery injury of forearm
S55.100	Radial artery injury at forearm level
S55.100x001	Radial artery injury of forearm
S55.101	Traumatic rupture of radial artery
S55.200	Venous injury at forearm level
S55.200x001	Forearm vein injury

S55.700	Multiple vascular injuries at forearm level
S55.700x001	Multiple vascular injuries of forearm
S55.800	Other vascular injuries at forearm level
S55.900	Injury of blood vessels at forearm level
S55.900x001	Forearm vascular injury
S56.000	Injury of thumb flexor and tendon at forearm level
S56.000x001	Forearm flexor thumb injury
S56.000x002	Injury of flexor tendon of forearm thumb
S56.000x003	Injury of flexor and tendon of forearm thumb
S56.001	Fracture of flexor muscle of forearm thumb
S56.100	Other finger flexor and tendon injuries at forearm level
S56.100x001	Injury of flexor digitorum of forearm
S56.100x002	Injury of flexor tendon of forearm
S56.100x003	Injury of flexor digitorum and tendon of forearm
S56.200	Other flexor and tendon injuries at forearm level
S56.200x001	Forearm flexor injury
S56.200x002	Injury of flexor tendon of forearm
S56.200x003	Injury of flexor and tendon of forearm
S56.300	Injury of extensor or abductor thumb muscle and tendon at forearm level
S56.300x001	Injury of extensor muscle of forearm thumb
S56.300x002	Injury of extensor tendon of forearm thumb
S56.300x003	Injury of extensor muscle and tendon of forearm thumb
S56.300x004	Injury of abductor thumb muscle of forearm
S56.300x005	Forearm abductor tendon injury
S56.300x006	Injury of abductor and tendon of forearm thumb
S56.301	Fracture of abductor muscle and tendon of forearm thumb
S56.400	Other finger extensor and tendon injuries at forearm level
S56.400x001	Injury of extensor digitorum of forearm
S56.400x002	Injury of extensor tendon of forearm finger
S56.400x003	Injury of extensor muscle and tendon of forearm finger
S56.500	Other extensor and tendon injuries at forearm level
S56.500x001	Forearm extensor injury

S56.500x002	Injury of extensor tendon of forearm
S56.500x003	Injury of forearm extensor and tendon
S56.700	Multiple muscle and tendon injuries at forearm level
S56.700x001	Multiple muscle and tendon injuries of forearm
S56.800	Injuries to other muscles and tendons at the forearm level
S56.801	Injury of forearm muscles and tendons
S57.000	Elbow crush injury
S57.000x001	Crushing injury of elbow
S57.000x011	Elbow thermal crush injury
S57.800	Crushing injury of other parts of forearm
S57.900	Crushing injury of forearm
S57.900x001	Crushing injury of forearm
S57.900x011	Thermal crush injury of forearm
S58.000	Traumatic amputation at elbow level
S58.000x001	Traumatic amputation of elbow
S58.100	Traumatic amputation at the level between elbow and wrist
S58.100x001	Horizontal traumatic amputation between elbow and wrist
S58.900	Traumatic amputation at forearm level
S58.900x001	Traumatic amputation of forearm
S59.700	Multiple injuries of forearm
S59.800	Other specific injuries of forearm
S59.900	forearm injuries
S60.000	Finger contusion without nail damage
S60.000x001	Finger contusion
S60.100	Finger contusion with nail damage
S60.200	Contusion of other parts of wrist and hand
S60.201	Wrist contusion
S60.202	Hand contusion
S60.700	Multiple superficial injuries of wrist and hand
S60.701	Multiple superficial injuries of hands
S60.800	Other superficial injuries of wrist and hand
S60.800x011	Wrist and hand abrasions
S60.800x012	Wrist scratch
S60.800x021	Wrist and hand blisters
S60.800x022	Hand blisters
S60.800x023	Wrist blisters
S60.800x031	Wrist and hand insect bites
S60.800x032	Wrist insect bite

S60.800x033	Insect bites on hands
S60.800x041	Superficial foreign body of wrist and hand
S60.800x042	Foreign body on the surface of wrist
S60.800x043	Foreign body on the surface of hand
S60.801	Foreign body on the surface of finger
S60.900	Superficial injury of wrist and hand
S60.900x001	Superficial injury of wrist and hand
S60.900x002	Superficial wrist injury
S60.901	Superficial hand injury
S60.902	Hand scratch
S61.000	Open wound of finger without nail damage
S61.000x001	Open injury of finger
S61.000x002	Open injury of thumb
S61.100	Open wound of finger with nail damage
S61.100x002	Open injury of thumb with nail injury
S61.700	Multiple open wounds on wrists and hands
S61.701	Open multiple wrist injuries
S61.702	Open multiple hand injuries
S61.800	Open wounds on the wrist and other parts of the hand
S61.800x011	Open injury of wrist and hand with fracture
S61.800x012	Open injury of hand with fracture
S61.800x013	Open injury of wrist with fracture
S61.800x021	Open injury of wrist and hand with dislocation
S61.800x022	Open hand injury with dislocation
S61.800x023	Open injury of wrist with dislocation
S61.800x081	Open injury of palm
S61.900	Open wound of wrist and hand
S61.900x002	Open injury of wrist
S61.900x004	Hand explosion injury
S61.900x005	Hand degloving injury
S61.901	Open hand injury
S61.902	Teared gloves
S62.000	Fracture of scaphoid bone
S62.000x001	Fracture of scaphoid bone of wrist
S62.010	Open fracture of scaphoid bone of hand
S62.100	Carpal fracture, other special
S62.100x011	Lunate fracture
S62.100x021	Triangular fracture

S62.100x031	Fracture of bean bone
S62.100x041	Most angular bone fracture
S62.100x051	Small polygonal bone fracture
S62.100x061	Capitate fracture
S62.100x071	Hamate fracture
S62.100x091	Multiple fracture of carpus
S62.101	Fracture of carpal bone
S62.110	Open fracture of special finger carpus
S62.111	Open carpal fracture
S62.200	Fracture of the first metacarpal bone
S62.200x011	Fracture of the first metacarpal base
S62.200x021	Fracture of the first metacarpal shaft
S62.200x031	Fracture of the first metacarpal neck
S62.200x041	Fracture of the first metacarpal head
S62.201	Bennett fracture
S62.210	Open fracture of the first metacarpal bone
S62.300	Metacarpal fracture, others
S62.300x002	Metacarpal epiphysis separation
S62.300x011	Metacarpal basal fracture
S62.300x021	Metacarpal shaft fracture
S62.300x031	Fracture of metacarpal neck
S62.300x041	Fracture of metacarpal head
S62.301	Metacarpal fracture
S62.310	Open fracture of special metacarpal bone
S62.311	Open metacarpal fracture
S62.400	Multiple metacarpal fractures
S62.410	Open multiple metacarpal fractures
S62.500	Thumb fracture
S62.500x002	Separation of thumb epiphysis
S62.500x011	Proximal segment of thumb fracture
S62.500x021	Fracture of distal segment of thumb
S62.510	Open thumb fracture
S62.600	Finger fracture, others
S62.600x002	Separation of phalangeal epiphysis
S62.600x011	Fracture of proximal phalanx
S62.600x021	Fracture of middle phalanx
S62.600x031	Fracture of distal phalanx
S62.610	Open fracture of special finger bone
S62.611	Open phalangeal fracture
S62.700	Multiple fractures of fingers

S62.710	Open multiple phalangeal fractures
S62.800	Fractures of other and unspecified parts of wrist and hand
S62.801	Hand fracture
S62.802	Phalangeal fracture
S62.810	Open fracture of other and unspecified parts of wrist and hand
S62.811	Open hand fracture
S63.000	Dislocation of wrist joint
S63.000x002	Dislocation of distal radius joint
S63.000x003	Distal joint dislocation of ulna
S63.000x011	Dislocation of lower radioulnar joint
S63.000x021	Dislocation of radiocarpal joint
S63.000x031	Dislocation of carpal joint
S63.000x041	Dislocation of proximal metacarpal joint
S63.000x042	Dislocation of carpometacarpal joint
S63.000x081	Dislocation of carpal bone
S63.000x082	Dislocation of scaphoid bone of wrist
S63.000x083	Perilunate dislocation of scaphoid bone of wrist
S63.100	Dislocation of finger joint
S63.100x001	Dislocation of interphalangeal joint
S63.100x002	Dislocation of thumb joint
S63.100x011	Metacarpophalangeal joint dislocation
S63.100x012	Dislocation of distal metacarpal joint
S63.100x013	Dislocation of thumb metacarpal joint
S63.100x021	Dislocation of distal interphalangeal joint of finger
S63.200	Multiple dislocations of fingers
S63.200x001	Multiple dislocation of finger joint
S63.300	Traumatic rupture of wrist and wrist ligament
S63.300x001	Fracture of carpal collateral ligament
S63.300x002	Rupture of radiocarpal ligament
S63.300x003	Rupture of ulnar carpal ligament
S63.300x004	Fracture of wrist and wrist ligament
S63.400	Traumatic rupture of finger ligaments at metacarpophalangeal and interphalangeal joints
S63.400x001	Fracture of metacarpophalangeal joint ligament
S63.400x002	Fracture of collateral ligament of metacarpophalangeal joint

S63.400x003	Rupture of palmar ligament
S63.400x004	Fracture of palm plate
S63.400x005	Rupture of ligament of interphalangeal joint
S63.400x006	Rupture of collateral ligament of interphalangeal joint
S63.401	Traumatic rupture of metacarpal ligament
S63.500	Wrist sprain and strain
S63.500x002	Wrist joint injury
S63.500x011	Wrain of wrist joint
S63.500x012	Injury of carpal joint
S63.500x021	Sprain of radiocarpal joint
S63.500x022	Injury of radiocarpal joint
S63.500x031	Wrist palm joint sprain
S63.500x032	Injury of carpometacarpal joint
S63.500x081	Sprain of lower radioulnar joint
S63.500x082	Lower radioulnar joint injury
S63.500x101	Injury of triangular fibrocartilage of wrist joint
S63.501	Wrist sprain
S63.600	Finger sprain and strain
S63.600x001	Sprain of thumb
S63.600x002	Sprain of phalange
S63.601	Finger joint sprain
S63.602	Sprain of metacarpophalangeal joint
S63.700	Sprain and strain of other and unspecified parts of hands
S63.700x001	Sprain of middle wrist joint
S63.701	Hand joint sprain
S64.000	Ulnar nerve injury at wrist and hand level
S64.000x001	Ulnar nerve injury of wrist
S64.000x002	Ulnar nerve injury of hand
S64.100	Median nerve injury at wrist and hand level
S64.100x001	Median nerve injury of wrist
S64.100x002	Median nerve injury of hand
S64.200	Radial nerve injury at wrist and hand level
S64.200x001	Wrist radial nerve injury
S64.200x002	Hand radial nerve injury
S64.300	Injury of thumb and finger nerve
S64.400	Finger nerve injury, others
S64.400x001	Finger nerve injury
S64.700	Multiple nerve injuries at the wrist and hand level
S64.700x001	Multiple nerve injuries of wrist and hand
S64.800	Other nerve injuries at the wrist and hand level

S64.900	Nerve injuries at the wrist and hand levels
S64.900x001	Nerve injury of wrist and hand
S65.000	Ulnar artery injury at wrist and hand level
S65.000x001	Ulnar artery injury of hand
S65.000x002	Ulnar artery injury of wrist
S65.100	Radial artery injury at wrist and hand level
S65.100x001	Wrist radial artery injury
S65.100x002	Hand radial artery injury
S65.200	Injury of superficial palmar arteriovenous arch
S65.300	Injury of deep palmar arteriovenous arch
S65.400	Blood vessel injury of thumb
S65.401	Traumatic rupture of thumb artery
S65.500	Finger vascular injury, others
S65.501	Traumatic digital artery rupture
S65.700	Multivascular injuries at the wrist and hand levels
S65.700x001	Multiple vascular injuries of wrist and hand
S65.800	Other vascular injuries at the wrist and hand levels
S65.900	Injury of blood vessels at the wrist and hand level
S65.900x001	Injury of wrist and hand vessels
S66.000	Injury of flexor longus thumb and tendons at wrist and hand levels
S66.000x001	Injury of flexor longus muscle and tendon of wrist and hand
S66.000x002	Injury of flexor longus muscle of wrist and hand
S66.000x003	Injury of flexor longus tendon of wrist and hand
S66.000x004	Injury of flexor pollicis longus and tendon of wrist
S66.000x005	Injury of flexor thumb longus muscle of wrist
S66.000x006	Injury of flexor digitorum longus tendon of wrist
S66.000x007	Injury of flexor longus thumb and tendon of hand
S66.000x008	Injury of flexor longus thumb of hand
S66.000x009	Injury of flexor longus tendon of hand
S66.100	Other finger flexor and tendon injuries at wrist and hand levels
S66.100x001	Wrist and finger flexor and tendon injuries
S66.100x002	Wrist and finger flexor injuries
S66.100x003	Injury of flexor tendon of wrist and finger

S66.100x004	Injury of flexor and tendon of wrist
S66.100x005	Injury of flexor digitorum muscle of wrist
S66.100x006	Injury of flexor tendon of wrist
S66.100x007	Finger flexor and tendon injuries
S66.100x008	Finger flexor injury
S66.100x009	Injury of flexor tendon of hand
S66.200	Injury of extensor and tendon of thumb at wrist and hand level
S66.200x001	Injury of extensor muscle and tendon of wrist and hand
S66.200x002	Injury of extensor muscle of wrist and hand
S66.200x003	Injury of extensor tendon of wrist and hand
S66.200x004	Injury of extensor muscle and tendon of wrist
S66.200x005	Injury of extensor muscle of wrist
S66.200x006	Injury of extensor tendon of wrist
S66.200x007	Injury of extensor muscle and tendon of hand
S66.200x008	Injury of extensor muscle of hand
S66.200x009	Injury of extensor tendon of hand
S66.300	Other finger extensor and tendon injuries at the wrist and hand levels
S66.300x001	Injury of wrist and finger extensors and tendons
S66.300x002	Injury of wrist and finger extensors
S66.300x003	Injury of extensor tendon of wrist and finger
S66.300x004	Injury of extensor digitorum and tendon of wrist
S66.300x005	Injury of extensor digitorum muscle of wrist
S66.300x006	Injury of extensor digitorum tendon of wrist
S66.300x007	Injury of extensor digitorum and tendon of hand
S66.300x008	Hand extensor injury
S66.300x009	Hand extensor tendon injury
S66.400	Injury of internal thumb muscles and tendons at wrist and hand levels
S66.400x001	Injury of internal muscles and tendons of wrist and thumb
S66.400x002	Injury of internal muscles of wrist and thumb
S66.400x003	Internal tendon injury of wrist and thumb
S66.400x004	Injury of internal muscle and tendon of thumb at wrist
S66.400x005	Injury of internal thumb muscle of wrist
S66.400x006	Injury of internal tendon of thumb at wrist

S66.400x007	Injuries of internal thumb muscles and tendons
S66.400x008	Injury of internal thumb muscle of hand
S66.400x009	Injury of internal tendon of thumb
S66.500	Injuries to internal muscles and tendons of other fingers at the wrist and hand levels
S66.500x001	Injury of internal muscles and tendons of wrist and fingers
S66.500x002	Injury of intrinsic muscles of wrist and finger
S66.500x003	Internal tendon injury of wrist and finger
S66.500x004	Injury of internal muscle and tendon of wrist
S66.500x005	Injury of internal muscle of wrist finger
S66.500x006	Injury of internal tendon of wrist finger
S66.500x007	Injury of internal muscles and tendons of the hand
S66.500x008	Injury of internal muscle of hand
S66.500x009	Injury of internal tendon of hand
S66.600	Multiple flexor and tendon injuries at wrist and hand levels
S66.600x001	Multiple flexor and tendon injuries of wrist and hand
S66.601	Multiple rupture of flexor muscle of hand
S66.700	Multiple extensor and tendon injuries at the wrist and hand levels
S66.700x001	Multiple extensor and tendon injuries of wrist and hand
S66.800	Injuries to other muscles and tendons at the wrist and hand levels
S66.900	Muscle and tendon injuries at the wrist and hand levels
S66.900x001	Wrist and hand muscle and tendon injuries
S66.900x002	Wrist muscle injury
S66.900x003	Hand muscle injury
S66.900x004	Finger muscle injury
S67.000	Crushing injury of thumb and other fingers
S67.000x001	Crushing injury of thumb
S67.000x003	Crushing and contusion of fingers
S67.000x011	Thumb thermal crush injury
S67.000x012	Finger thermal crush injury
S67.001	Finger crush injury
S67.800	Crushing injury of wrist and hand in other and unspecified parts
S67.800x001	Wrist crush injury
S67.800x003	Crushing and contusion of hand
S67.800x011	Hand heat crush injury

S67.801	Hand crush injury
S68.000	Traumatic amputation of thumb (complete) (partial)
S68.000x002	Incomplete amputation of thumb
S68.001	Total amputation of thumb
S68.100	Traumatic amputation of single finger (complete) (partial), others
S68.100x001	Single finger incomplete cutoff
S68.100x002	Single finger complete disconnection
S68.200	Traumatic amputation (complete) (partial) of only two or more fingers
S68.200x001	Multi finger incomplete cutting
S68.201	Multiple fingers completely cut off
S68.300	Combined traumatic amputation of finger (part) with wrist and other parts of hand
S68.400	Traumatic amputation of hand at wrist level
S68.400x001	Traumatic amputation of wrist
S68.800	Traumatic amputation of wrist and other parts of hand
S68.800x001	Traumatic amputation of palm
S68.900	Traumatic amputation at wrist and hand level
S69.700	Multiple injuries of wrist and hand
S69.800	Other specific injuries to wrists and hands
S69.900	Wrist and hand injuries
S69.900x001	Wrist injury
S69.900x002	Hand injury
S69.900x003	Thumb injury
S69.900x004	Finger injury
S70.000	Hip contusion
S70.000x001	Hip contusion
S70.100	Thigh contusion
S70.700	Multiple superficial injuries of hip and thigh
S70.700x001	Multiple superficial injuries of thigh
S70.700x002	Multiple superficial injuries of hip
S70.800	Other superficial injuries of hip and thigh
S70.800x011	Hip Scratch
S70.800x012	Scratch of thigh
S70.800x021	Hip blisters
S70.800x022	Femoral vesicles
S70.800x031	Hip worm bite
S70.800x032	Femoral insect bite
S70.800x041	Superficial foreign body of hip

S70.800x042	Foreign body on the surface of thigh
S70.900	Superficial injury of hip and thigh
S70.900x001	Superficial injury of hip
S70.900x002	Superficial injury of thigh
S70.900x003	Superficial injury of thigh
S70.901	Thigh hematoma
S71.000	Open wound of hip
S71.000x001	Open injury of hip
S71.100	Open wound of thigh
S71.101	Avulsion injury of thigh
S71.700	Multiple open wounds on hip and thigh
S71.800	Open wounds in other and unspecified parts of the pelvic band
S71.800x011	Open injury of hip with fracture
S71.800x012	Open injury of thigh with fracture
S71.800x021	Open injury of hip with dislocation
S71.800x022	Open injury of thigh with dislocation
S71.801	Open pelvic band injury
S72.000	Femoral neck fracture
S72.000x011	Intracystic fracture of femoral joint
S72.000x021	Separation of femoral head epiphysis
S72.000x031	Subcapitular fracture of femoral neck
S72.000x041	Transcervical fracture of femoral neck
S72.000x051	Femoral neck basal fracture
S72.000x081	Femoral head fracture
S72.000x082	Femoral hip fracture
S72.010	Open femoral neck fracture
S72.100	Transtrochanteric fracture
S72.100x001	Fracture of greater trochanter of femur
S72.100x002	Fracture of lesser trochanter of femur
S72.100x011	Intertrochanteric fracture of femur
S72.101	Intertrochanteric fracture of femur
S72.110	Open intertrochanteric fracture of femur
S72.200	Subtrochanteric fracture
S72.200x001	Subtrochanteric fracture of femur
S72.210	Open subtrochanteric fracture of femur
S72.300	Femoral shaft fracture
S72.310	Open fracture of femoral shaft

S72.400	Lower femur fracture
S72.400x001	Distal femoral fracture
S72.400x012	Fracture of internal femoral condyle
S72.400x013	Fracture of external femoral condyle
S72.400x021	Separation of distal femoral epiphysis
S72.400x031	Supracondylar fracture of femur
S72.400x041	Femoral intercondylar fracture
S72.401	Fracture of femoral condyle
S72.410	Open fracture of distal femur
S72.700	Multiple fractures of femur
S72.710	Open multiple femoral fractures
S72.800	Fracture of other parts of femur
S72.810	Open fracture of femur at specific site
S72.900	Femoral fracture
S72.900x002	Separation of femoral epiphysis
S72.910	Open femoral fracture
S73.000	ddh
S73.000x002	Dislocation of hip joint
S73.000x003	Acetabular dislocation
S73.000x011	Posterior dislocation of hip joint
S73.000x021	Anterior dislocation of hip joint
S73.001	Subluxation of hip joint
S73.100	Hip sprain and strain
S73.100x001	Sprain of hip joint
S73.100x011	Sprain of iliofemoral ligament
S73.100x021	Sprain of ligament of iliac joint capsule
S73.101	Hip sprain
S74.000	Sciatic nerve injury at hip and thigh level
S74.000x001	Injury of sciatic nerve
S74.000x002	Hip sciatic nerve injury
S74.000x003	Injury of sciatic nerve of thigh
S74.100	Femoral nerve injury at hip and thigh level
S74.100x001	Femoral nerve injury
S74.100x002	Hip femoral nerve injury
S74.100x003	Femoral nerve injury of thigh
S74.200	Dermatosensory nerve injury at hip and thigh level
S74.200x001	Hip cutaneous sensory nerve injury
S74.200x002	Injury of sensory nerve of thigh skin
S74.700	Multiple nerve injuries at hip and thigh levels
S74.700x001	Multiple nerve injuries at hip
S74.700x002	Multiple nerve injuries of thigh

S74.800	Other nerve injuries at hip and thigh levels
S74.801	Obturator nerve injury
S74.900	Nerve injuries at hip and thigh levels
S74.900x001	Hip nerve injury
S74.900x002	Nerve injury of thigh
S75.000	Femoral artery injury
S75.000x002	Injury of superficial femoral artery
S75.000x003	Deep femoral artery injury
S75.000x004	Traumatic femoral aneurysm
S75.000x005	Traumatic femoral pseudoaneurysm
S75.001	Traumatic rupture of deep femoral artery
S75.100	Femoral vein injury at hip and thigh level
S75.100x001	Femoral vein injury
S75.100x002	Femoral vein injury of hip
S75.100x003	Femoral vein injury of thigh
S75.200	Injury of great saphenous vein at hip and thigh level
S75.200x001	Injury of great saphenous vein of thigh
S75.700	Multivascular injuries at hip and thigh levels
S75.700x001	Multiple vascular injuries in hip
S75.700x002	Multiple vascular injuries of thigh
S75.800	Other vascular injuries at hip and thigh levels
S75.900	Injury of blood vessels at hip and thigh levels
S75.900x001	Hip vascular injury
S75.900x002	Injury of thigh blood vessels
S75.901	Traumatic femoral arteriovenous fistula
S76.000	Hip muscle and tendon injuries
S76.000x002	Hip muscle injury
S76.000x003	Hip tendon injury
S76.100	Injury of quadriceps and tendon
S76.100x001	Injury of quadriceps femoris and tendon
S76.100x002	Muscle injury of quadriceps femoris
S76.100x003	Tendon injury of quadriceps femoris
S76.101	Tendon rupture of quadriceps femoris
S76.102	Injury of patellar ligament
S76.200	Injury of adductor muscle and tendon of thigh
S76.200x002	Muscle injury of adductor muscle of thigh
S76.200x003	Injury of adductor tendon of thigh

S76.300	Injury of posterior muscle groups and tendons at thigh level
S76.300x001	Injury of posterior thigh muscle group and tendon
S76.300x002	Muscle injury of posterior thigh muscles
S76.300x003	Tendon injury of posterior thigh muscles
S76.301	Injury of tendon at the back of thigh
S76.400	Other and unspecified muscle and tendon injuries at thigh level
S76.401	Injury of thigh tendon
S76.402	Rupture of thigh muscle
S76.700	Multiple muscle and tendon injuries at the hip joint and thigh level
S76.700x001	Multiple muscle and tendon injuries in the hip and thigh
S77.000	Hip crush injury
S77.000x011	Hip thermal crush injury
S77.100	Crushing injury of thigh
S77.100x001	Hot crush injury of thigh
S77.200	Hip with thigh crush injury
S77.200x001	Crushing injury of hip and thigh
S77.200x011	Hip with hot crush injury of thigh
S78.000	Traumatic amputation of hip
S78.000x001	Hip amputation
S78.100	Traumatic amputation at the level between hip and knee
S78.100x001	Thigh amputation
S78.900	Traumatic amputation at hip and thigh level
S79.700	Multiple injuries of hip and thigh
S79.701	Multiple thigh injuries
S79.800	Other specific injuries of hip and thigh
S79.800x001	Soft tissue injury around hip joint
S79.900	Hip and thigh injuries
S79.900x001	Hip injury
S79.901	Thigh injury
S79.902	Peri hip soft tissue injury
S80.000	Contusion of knee
S80.000x001	Knee contusion
S80.100	Contusion of other and unspecified parts of the lower leg
S80.100x002	Hematoma of lower leg
S80.101	Crural contusion
S80.700	Multiple superficial injuries of lower leg
S80.800	Other superficial injuries of the lower leg
S80.800x011	Crural bruising
S80.800x012	Knee bruising
S80.800x013	Abrasion of popliteal fossa
S80.800x021	Vesicle of calf
S80.800x022	Knee blister
S80.800x023	Popliteal vesicle
S80.800x031	Calf insect bite
S80.800x032	Knee insect bite
S80.800x033	Popliteus bite
S80.800x041	Foreign body on the surface of lower leg
S80.800x042	Superficial foreign body of knee
S80.800x043	Superficial foreign body in popliteal fossa
S80.900	Superficial injury of lower leg
S80.901	Hematoma of knee
S81.000	Open wound of knee
S81.700	Multiple open wounds on the lower leg
S81.800	Open wounds in other parts of the lower leg
S81.800x011	Open injury of lower leg with fracture
S81.800x021	Open injury of lower leg with dislocation
S81.800x081	Open injury of fibula
S81.800x082	Open injury of popliteal fossa
S81.800x083	Open injury of tibia
S81.900	Open wound of lower leg
S81.901	Avulsion injury of lower leg
S82.000	Patella fracture
S82.000x002	Fracture of patella cartilage
S82.000x003	Sleeve avulsion fracture of patella
S82.000x004	Sleeve fracture of patella
S82.010	Open patellar fracture
S82.100	Fracture of upper tibia
S82.100x011	Proximal tibia fracture with fibula fracture
S82.100x012	Tibial plateau with fibular fracture
S82.100x081	proximal tibia fracture
S82.100x082	Separation of proximal tibial epiphysis
S82.100x084	Fracture of tibial condyle
S82.100x085	Fracture of intercondylar spine of tibia
S82.100x086	Fracture of lateral condyle of tibia
S82.100x087	Tibial plateau fracture
S82.100x088	Tibial plateau with intercondylar fracture
S82.100x089	Tibial tubercle fracture
S82.101	Closed fracture of tibial plateau

S82.102	Tibial head fracture
S82.110	Open fracture of upper tibia
S82.111	Open fracture of tibial head
S82.200	Tibial shaft fracture
S82.200x011	Tibial shaft fracture with fibular fracture
S82.200x081	Tibial shaft fracture
S82.201	Fracture of tibiofibular shaft
S82.202	Tibial fracture
S82.203	Closed fracture of tibia and fibula
S82.210	Open fracture of tibial shaft
S82.211	Open tibia fracture
S82.212	Open tibiofibular shaft fracture
S82.300	Fracture of lower tibia
S82.300x011	Distal tibia fracture with fibula fracture
S82.300x012	Separation of lower tibiofibular epiphysis
S82.300x081	Fracture of distal tibia
S82.300x082	Separation of distal tibial epiphysis
S82.300x083	Pilon fracture
S82.301	Fracture of lower end of tibia and fibula
S82.310	Open fracture of lower tibia
S82.311	Open fracture of distal tibia and fibula
S82.400	Fibula fracture only
S82.400x001	Fibular fracture
S82.400x002	Separation of distal fibular epiphysis
S82.400x011	Fracture of proximal fibula
S82.400x012	Fracture of fibular head
S82.400x013	Fracture of fibular neck
S82.400x014	Fracture of fibular capitulum
S82.400x091	Multiple fracture of fibula
S82.401	Fracture of fibular shaft
S82.410	Open fibular fracture
S82.411	Open fracture of fibular shaft
S82.500	Medial malleolus fracture
S82.501	Tibial fracture involving ankle joint
S82.510	Open fracture of medial malleolus
S82.600	Fracture of lateral malleolus
S82.601	Fracture of fibula involving ankle joint
S82.610	Open fracture of lateral malleolus
S82.700	Multiple fractures of lower leg
S82.710	Open multiple fractures of lower leg
S82.800	Fracture of other parts of the lower leg

S82.800x081	Ankle fracture
S82.800x082	ankle fracture
S82.801	cotton fracture
S82.802	Bilateral ankle fracture
S82.803	Closed fracture of ankle
S82.810	Open fracture of specific part of lower leg
S82.811	Open trimalleolar fracture
S82.812	Open fracture of both ankles
S82.900	Crural fracture
S82.910	Open fracture of lower leg
S83.000	Dislocation of patella
S83.001	Subluxation of patella
S83.100	Dislocation of knee joint
S83.100x011	Anterior dislocation of proximal tibia
S83.100x012	Posterior dislocation of distal femur
S83.100x021	Posterior dislocation of proximal tibia
S83.100x031	Medial dislocation of proximal tibia
S83.100x041	Lateral dislocation of proximal tibia
S83.100x081	Dislocation of tibiofibular joint
S83.101	Subluxation of knee joint
S83.102	Proximal dislocation of tibiofibular joint
S83.200	Meniscus tear, recent
S83.200x001	Tear of meniscus of knee
S83.200x002	Barrel handle tear of lateral meniscus of knee
S83.200x003	Barrel handle tear of medial meniscus of knee
S83.200x004	Barrel handle tear of meniscus of knee
S83.200x005	Tear of medial meniscus of knee
S83.200x006	Tear of lateral meniscus of knee
S83.201	Medial meniscus injury of knee
S83.202	Injury of lateral meniscus of knee
S83.300	Tears of knee cartilage, recent
S83.300x001	Tear of cartilage of knee joint
S83.400	Sprain and strain involving the knee joint (fibula) (tibia) collateral ligament
S83.400x001	Sprain of collateral ligament of knee joint
S83.400x002	Rupture of collateral ligament of knee joint
S83.400x003	Injury of collateral ligament of knee joint
S83.400x011	Sprain of lateral collateral ligament of knee joint
S83.400x012	Injury of lateral collateral ligament of knee joint

S83.400x021	Sprain of medial collateral ligament of knee joint
S83.400x022	Injury of medial collateral ligament of knee joint
S83.400x031	Partial rupture of lateral collateral ligament of knee joint
S83.400x032	Complete rupture of lateral collateral ligament of knee joint
S83.400x041	Partial rupture of medial collateral ligament of knee joint
S83.400x042	Complete rupture of medial collateral ligament of knee joint
S83.401	Strain of collateral ligament of knee joint
S83.500	Sprain and strain involving the (anterior) (posterior) cruciate ligament of the knee
S83.500x001	Rupture of cruciate ligament of knee joint
S83.500x002	injuries of crucial ligaments
S83.500x003	Injury of cruciate ligament of knee joint
S83.500x011	Sprain of anterior cruciate ligament of knee joint
S83.500x012	Injury of anterior cruciate ligament of knee joint
S83.500x021	Sprain of posterior cruciate ligament of knee joint
S83.500x022	Injury of posterior cruciate ligament of knee joint
S83.500x031	Partial rupture of anterior cruciate ligament of knee joint
S83.500x032	Complete rupture of anterior cruciate ligament of knee joint
S83.500x041	Partial rupture of posterior cruciate ligament of knee joint
S83.500x042	Complete rupture of posterior cruciate ligament of knee joint
S83.501	Strain of cruciate ligament of knee joint
S83.600	Sprain and strain of other and unspecified parts of the knee
S83.600x002	Knee joint injury
S83.600x004	Sprain of proximal tibiofibular joint
S83.600x005	Injury of proximal tibiofibular joint
S83.600x006	Sprain of proximal tibiofibular ligament
S83.600x007	Injury of proximal tibiofibular ligament
S83.600x009	Rupture of patellar tendon
S83.601	Sprain of knee joint
S83.602	Tear of upper end of tibiofibular ligament
S83.603	Sprain of upper tibiofibular joint
S83.700	Injuries to multiple structures of the knee
S83.700x001	Lateral meniscus of knee with collateral ligament injury

S83.700x002	Lateral meniscus of knee with cruciate ligament injury
S83.700x003	Multiple injuries of knee joint
S83.700x004	Medial meniscus of knee with collateral ligament injury
S83.700x005	Medial meniscus of knee with cruciate ligament injury
S83.700x006	Multiple ligament injuries of knee joint
S84.000	Tibial nerve injury at the lower leg level
S84.000x001	Injury of posterior tibial nerve
S84.000x002	Tibial nerve injury
S84.100	Peroneal nerve injury at the lower leg level
S84.100x001	Peroneal nerve injury
S84.200	Dermatosensory nerve injury at the lower leg level
S84.200x001	Sensory nerve injury of lower leg skin
S84.700	Multiple nerve injuries at the lower leg level
S84.700x001	Multiple nerve injuries in the lower leg
S84.800	Other nerve injuries at the lower leg level
S84.800x001	Common peroneal nerve injury
S84.800x002	Sural nerve injury
S84.900	Nerve injury at the lower leg level
S84.900x001	Nerve injury of lower leg
S85.000	Popliteal artery injury
S85.100	Tibial artery injury (anterior) (posterior)
S85.100x002	Injury of anterior tibial artery
S85.101	Injury of posterior tibial artery
S85.102	Traumatic posterior tibial artery thrombosis
S85.200	Peroneal artery injury
S85.300	Injury of great saphenous vein at lower leg level
S85.300x001	Injury of great saphenous vein of lower leg
S85.400	Injury of small saphenous vein at lower leg level
S85.400x001	Injury of saphenous vein of lower leg
S85.500	Popliteal vein injury
S85.700	Multiple vascular injuries at the lower leg level
S85.700x001	Multiple vascular injuries in the lower leg
S85.800	Other vascular injuries at the lower leg level
S85.800x001	Injury of posterior tibial vessels
S85.801	Traumatic posterior tibial artery and vein injury

S85.900	Injury of blood vessels at the lower leg level
S85.900x001	Crural vascular injury
S86.000	Achilles tendon injury
S86.001	Achilles tendon rupture
S86.100	Other muscle and tendon injuries in the posterior muscle group at the calf level
S86.100x001	Injury of posterior calf muscle group and tendon
S86.100x002	Muscle injury of posterior calf muscles
S86.100x003	Tendon injury of posterior calf muscles
S86.200	Muscle and tendon injuries in the anterior muscle group at the calf level
S86.200x002	Tendon injury of anterior calf muscles
S86.201	Tendon injury of anterior horizontal muscle group of lower leg
S86.300	Muscle and tendon injuries in the peroneal muscle group at the calf level
S86.300x001	Injury of peroneal muscle group and tendon
S86.300x002	Injury of peroneal muscles
S86.300x003	Tendon injury of peroneal muscles
S86.300x004	Gastrocnemius rupture
S86.300x005	Injury of peroneal long and short muscles
S86.300x006	Traumatic peroneal tendon slippage
S86.301	Tendon injury of horizontal peroneal muscle group of lower leg
S86.700	Multiple muscle and tendon injuries at the calf level
S86.700x001	Multiple muscle and tendon injuries in the lower leg
S86.700x002	Tibiofibular tendon rupture
S86.701	Multiple tendon injuries at the lower leg level
S86.800	Other muscle and tendon injuries at the calf level
S86.900	Muscle and tendon injuries at the calf level
S86.901	Crural horizontal muscle injury
S87.000	Knee crush injury
S87.800	Crushing injury of other and unspecified parts of lower leg
S87.800x001	Crural thermal crush injury
S87.801	Crural crush injury
S88.000	Traumatic amputation at knee level
S88.000x001	Knee amputation
S88.100	Traumatic amputation at the level between knee and ankle
S88.100x001	Calf amputation
S88.900	Traumatic amputation at the lower leg level
S89.700	Multiple injuries of lower leg
S89.800	Other specific injuries of the lower leg
S89.900	Lower leg injury
S90.000	Ankle contusion
S90.100	Toe contusion without toenail damage
S90.200	Toe contusion with toenail damage
S90.300	Contusion of other and unspecified parts of the foot
S90.300x001	Injury of accessory navicular bone
S90.300x002	Osteochondral injury of talus
S90.300x003	Injury of posterior trigone of talus
S90.301	Foot contusion
S90.700	Multiple superficial injuries of ankle and foot
S90.800	Other superficial injuries of ankle and foot
S90.800x011	Ankle and foot abrasions
S90.800x012	Ankle scratch
S90.800x013	Foot abrasions
S90.800x021	Ankle and foot blisters
S90.800x022	Ankle blister
S90.800x023	Foot blister
S90.800x031	Ankle and foot insect bites
S90.800x032	Ankle insect bite
S90.800x033	Foot insect bite
S90.800x041	Superficial foreign body of ankle and foot
S90.800x042	Superficial foreign body of ankle
S90.800x043	Foreign body on the surface of foot
S90.900	Superficial injury of ankle and foot
S90.900x001	Superficial injury of ankle and foot
S90.900x002	Superficial ankle injury
S90.900x003	Superficial injury of foot
S90.901	Haematoma of toenail
S91.000	Open wound of ankle
S91.100	Open wound of toe without toenail damage
S91.200	Open wound of toe with nail damage
S91.300	Open wounds in other parts of the foot
S91.300x002	Foot degloving injury
S91.300x003	Open injury of heel

S91.300x811	Open injury of ankle and foot with fracture
S91.300x812	Open injury of ankle with fracture
S91.300x813	Open injury of foot with fracture
S91.300x821	Open injury of ankle and foot with dislocation
S91.300x822	Open injury of ankle with dislocation
S91.300x823	Open injury of foot with dislocation
S91.301	Open foot injury
S91.302	Skin laceration of foot
S91.303	Foot laceration
S91.700	Multiple open wounds on ankle and foot
S91.700x002	Multiple open injuries of ankle
S91.700x003	Multiple open injuries of foot
S92.000	Calcaneal fracture
S92.010	Open calcaneal fracture
S92.100	Talus fracture
S92.100x003	Fracture of talus body
S92.101	Talar neck fracture
S92.110	Open talus fracture
S92.200	Fracture of tarsal bone, others
S92.200x001	Tarsal fracture
S92.200x011	Scaphoid fracture
S92.200x081	Fracture of tarsal joint
S92.201	Fracture of cuboid bone
S92.202	Fracture of scaphoid bone of foot
S92.203	Wedge bone fracture (foot)
S92.210	Open fracture of tarsal bone of special finger
S92.300	Metatarsal fracture
S92.300x001	Fracture of tarsometatarsal joint
S92.300x003	Metatarsal basal fracture
S92.300x004	Metatarsal epiphysis injury
S92.310	Open metatarsal fracture
S92.400	Thumb fracture
S92.410	Open fracture of big toe
S92.500	Phalangeal fracture, others
S92.500x001	Phalangeal fracture
S92.500x002	Injury of phalangeal epiphysis
S92.510	Open fracture of special phalange
S92.700	Multiple fractures of foot
S92.700x001	Multiple fracture of foot bone
S92.710	Open multiple foot fractures
S92.900	Foot fracture
S92.910	Open foot fracture
S93.000	Ankle dislocation

S93.000x004	Talus dislocation
S93.000x005	Dislocation of fibula
S93.001	Subluxation of ankle joint
S93.002	Tibial talus joint dislocation
S93.003	Dislocation of distal tibiofibular joint
S93.100	Toe dislocation
S93.100x001	Dislocation of phalanx
S93.101	Dislocation of toe joint
S93.102	Metatarsophalangeal joint subluxation
S93.103	Metatarsophalangeal joint dislocation
S93.200	Ligament rupture at ankle and foot level
S93.200x001	Fracture of ankle and foot ligaments
S93.200x002	Fracture of ankle ligament
S93.200x003	Fracture of ligament of foot
S93.200x004	Fracture of anterior talofibular ligament of ankle
S93.200x005	Rupture of calcaneus fibular ligament
S93.300	Dislocation of other and unspecified parts of the foot
S93.300x001	Lisfranc damage
S93.300x011	Dislocation of tarsal bone
S93.300x021	Dislocation of middle tarsal joint
S93.300x031	Fracture and dislocation of tarsometatarsal joint [Lisfranc fracture and dislocation]
S93.300x032	Dislocation of tarsometatarsal joint
S93.300x081	Dislocation of talocavicular joint
S93.301	Dislocation of foot
S93.302	Metatarsal dislocation
S93.303	Dislocation of scaphoid bone of foot
S93.400	Ankle sprain and strain
S93.400x002	Ankle joint injury
S93.400x004	Sprain of medial collateral ligament of ankle
S93.400x012	Ankle triangle ligament injury
S93.400x021	Sprain of calcaneus fibular ligament
S93.400x022	Injury of calcaneal fibular ligament
S93.400x031	Distal tibiofibular ligament sprain
S93.400x032	Distal tibiofibular ligament injury
S93.400x041	Ankle cartilage injury
S93.401	Ankle sprain
S93.402	Injury of medial collateral ligament of ankle

S93.403	Triangular ligament rupture
S93.404	Sprain of triangular ligament
S93.405	Tear of distal tibiofibular ligament
S93.500	Sprain and strain of toe
S93.500x001	Sprain of interphalangeal joint
S93.500x002	Injury of interphalangeal joint
S93.500x003	Sprain of metatarsophalangeal joint
S93.500x004	Metatarsophalangeal joint injury
S93.500x005	Sprain of toe
S93.500x006	Toe injury
S93.600	Sprain and strain of other and unspecified parts of the foot
S93.600x001	Sprain of tarsal ligament
S93.600x002	Injury of tarsal ligament
S93.600x003	Sprain of tarsometatarsal ligament
S93.600x004	Injury of tarsometatarsal ligament
S93.601	Sprain of foot
S94.000	Injury of lateral plantar nerve
S94.100	Medial plantar nerve injury
S94.200	Injury of deep peroneal nerve at ankle and foot level
S94.200x001	Injury of deep peroneal nerve of ankle and foot
S94.200x002	End injury of external collateral branch of deep peroneal nerve
S94.300	Dermatosensory nerve injury at ankle and foot level
S94.300x001	Sensory nerve injury of ankle and foot skin
S94.700	Multiple nerve injuries at ankle and foot levels
S94.700x001	Multiple nerve injuries of ankle and foot
S94.800	Other nerve injuries at ankle and foot levels
S94.800x001	Injury of toe nerve
S94.900	Nerve injuries at the ankle and foot levels
S94.900x001	Injury of ankle and foot nerves
S95.000	Injury of dorsalis pedis artery
S95.100	Plantar artery injury
S95.200	Injury of dorsal vein of foot
S95.700	Multivascular injuries at ankle and foot levels
S95.700x001	Multiple vascular injuries of ankle and foot
S95.800	Other vascular injuries at ankle and foot levels
S95.900	Injury of blood vessels at ankle and foot level
S95.900x001	Injury of ankle and foot vessels
S96.000	Injury of flexor digitorum longus and tendon at ankle and foot level

S96.000x001	Injury of flexor longus and tendon of ankle and toe
S96.100	Injury of extensor digitorum longus and tendon at ankle and foot level
S96.100x001	Injury of extensor longus and tendon of ankle and toe
S96.100x002	Tendon injury of long hallucis pedis
S96.101	Rupture of extensor hallucis longus tendon
S96.102	Tendon rupture of extensor digitorum
S96.200	INNER MUSCLE AND TENDON INJURY AT ANKLE AND FOOT LEVEL
S96.200x001	Injury of internal muscles and tendons of ankle and foot
S96.700	Multiple muscle and tendon injuries at ankle and foot levels
S96.700x001	Multiple muscle and tendon injuries of ankle and foot
S96.701	Multiple tendon injuries at ankle and foot levels
S96.800	Other muscle and tendon injuries at ankle and foot levels
S96.800x001	Injury of posterior tibial tendon of ankle
S96.800x002	Injury of tendon of toe
S96.801	Toe tendon rupture
S96.900	Muscle and tendon injuries at ankle and foot levels
S96.900x002	Injuries to ankle and foot muscles and tendons
S97.000	Crushing injury of ankle
S97.000x001	Crushing injury of ankle
S97.000x011	Ankle thermal crush injury
S97.100	Crushing injury of toe
S97.100x001	Crushing injury of toe
S97.100x011	Thermal crush injury of toe
S97.800	Crushing injury of ankle and other parts of foot
S97.800x001	Crushing injury of foot
S97.800x002	Crushing injury of ankle and foot
S97.800x011	Foot thermal crush injury
S97.801	Foot crush injury
S98.000	Traumatic amputation of foot at ankle level
S98.000x001	Ankle amputation
S98.100	Traumatic amputation of one toe
S98.100x001	Single toe amputation
S98.200	Traumatic amputation of two or more toes
S98.200x001	Amputation of two toes
S98.200x002	Mutitoectomy
S98.300	Traumatic amputation of other parts of the foot

S98.400	Traumatic amputation of foot
S99.700	Multiple injuries of ankle and foot
S99.700x001	Multiple foot injuries
S99.700x002	Multiple injuries of ankle
S99.800	Other specific injuries of ankle and foot
S99.800x001	Avulsion injury of foot soft tissue
S99.900	Ankle and foot injuries
S99.900x001	Foot injury
S99.900x002	Ankle injury
T00.000	Superficial injury involving head and neck
T00.000x001	Superficial head and neck injuries
T00.100	Superficial injury involving chest with abdomen, lower back and pelvis
T00.100x001	Thorax with superficial injury of abdomen, lower back and pelvis
T00.200	Superficial injury involving multiple parts of upper limb
T00.200x001	Multiple superficial injuries of upper limbs
T00.300	Superficial injuries involving multiple parts of lower limbs
T00.300x001	Multiple superficial injuries of lower limbs
T00.600	Superficial injury involving upper limbs with multiple parts of lower limbs
T00.600x001	Multiple superficial injuries of upper and lower limbs
T00.800	Superficial injuries involving other complex parts of the body
T00.800x001	Superficial injury of compound body parts
T00.900	Multiple superficial damages
T00.900x001	Multiple superficial skin injuries
T00.900x002	Multiple superficial skin abrasions
T00.900x003	Multiple superficial skin blisters
T00.900x004	Multiple superficial skin bruises
T00.900x005	Multiple superficial skin contusions
T00.900x006	Multiple superficial hematoma of skin
T00.900x007	Multiple superficial non-toxic insect bites
T00.901	Multiple contusions
T00.902	Multiple skin damage
T01.000	Open wound involving head and neck
T01.000x001	Open head and neck injuries
T01.100	Open wound involving chest with abdomen, lower back and pelvis

T01.100x001	Open injury of chest with abdomen, lower back and pelvis
T01.101	Open thoracoabdominal injury
T01.200	Open wound involving multiple parts of upper limb
T01.200x001	Multiple open injuries of upper limbs
T01.300	Open wound involving multiple parts of lower limbs
T01.300x001	Multiple open injuries of lower limbs
T01.301	Lower limb skin avulsion
T01.302	Multiple lacerations of lower limbs
T01.600	Open wound involving upper limb and multiple parts of lower limb
T01.600x001	Multiple open injuries of upper and lower limbs
T01.800	Open wound involving other composite body parts
T01.800x001	Open injury of compound body parts
T01.900	Multiple open wounds
T01.901	Multiple puncture injuries
T01.902	Multiple animal bites
T01.903	Multiple cutting injuries
T01.904	Multiple laceration
T02.000	Involved head with neck fracture
T02.000x001	Head and neck fractures
T02.010	Open head with neck fracture
T02.100	Fracture involving chest with lower back and pelvis
T02.100x001	Multiple fracture of trunk
T02.110	Open multiple trunk fractures
T02.200	Fracture involving multiple parts of single upper limb
T02.200x001	Multiple fractures of single upper limb
T02.210	Open multiple fractures of single upper limb
T02.300	Fracture involving multiple parts of single lower limb
T02.300x001	Multiple fractures of single lower limb
T02.310	Open multiple fractures of single lower limb
T02.400	Fracture involving multiple parts of both upper limbs
T02.400x001	Multiple fractures of both upper limbs
T02.410	Open multiple fractures of both upper limbs
T02.500	Fracture involving multiple parts of both lower limbs
T02.500x001	Multiple fractures of both lower limbs

T02.510	Open multiple fractures of both lower limbs
T02.600	Fracture involving upper limb with multiple parts of lower limb
T02.600x001	Upper limb with multiple fractures of lower limb
T02.600x011	Multiple open fractures of upper limb with lower limb
T02.610	Open multiple limb fractures
T02.700	Fracture involving chest with lower back, pelvis and limbs
T02.700x001	Thorax with fracture of lower back, pelvis and extremities
T02.700x021	Pelvis with bone fracture of lower limb
T02.710	Open chest with fracture of lower back, pelvis and extremities
T02.800	Fracture involving other complex parts of the body
T02.800x001	Fracture of compound part of the body
T02.810	Open fracture of the body, especially the compound part
T02.900	Multiple fractures
T02.910	Open multiple fractures
T03.000	Involved head with dislocation, sprain and strain of neck
T03.000x001	Dislocation of head and neck
T03.000x002	Head and neck sprains
T03.000x003	Head and neck injuries
T03.100	Involve the chest with dislocation, sprain and strain of lower back and pelvis
T03.100x001	Thorax with dislocation of lower back and pelvis
T03.100x002	Thorax with lower back and pelvis sprain
T03.100x003	Thorax with injury of lower back and pelvis
T03.200	Dislocation, sprain and strain involving multiple parts of upper limbs
T03.200x001	Multiple dislocations of upper limbs
T03.200x002	Multiple sprains of upper limbs
T03.200x003	Multiple injuries of upper limbs
T03.300	Dislocation, sprain and strain involving multiple parts of lower limbs
T03.300x001	Multiple dislocations of lower limbs
T03.300x002	Multiple sprains of lower limbs
T03.300x003	Multiple injuries of lower limbs
T03.400	Involved upper limbs with dislocation, sprain and strain of multiple parts of lower limbs
T03.400x001	Multiple dislocations of upper and lower limbs
T03.400x002	Multiple sprains of upper and lower limbs
T03.400x003	Multiple injuries of upper and lower limbs
T03.800	Dislocation, sprain and strain involving other complex parts of the body
T03.800x001	Dislocation of complex parts of the body
T03.800x002	Sprain of compound parts of the body
T03.900	Multiple dislocations, sprains and strains
T03.900x001	Multiple dislocations
T03.900x002	Multiple sprains
T04.000	Crushing injury involving head and neck
T04.000x001	Head and neck crush injury
T04.000x011	Head with heat crush injury of neck
T04.100	Crushing injury involving chest with abdomen, lower back and pelvis
T04.100x001	Crushing injury of trunk
T04.100x011	Thorax with abdominal, lower back and pelvis thermal crush injury
T04.200	Crushing injury involving multiple parts of upper limb
T04.200x001	Multiple crush injuries of upper limbs
T04.200x011	Multi part heat crush injury of upper limb
T04.300	Crushing injury involving multiple parts of lower limbs
T04.300x001	Multiple crush injuries of lower limbs
T04.300x011	Heat crush injury of multiple parts of lower limb
T04.400	Crushing injury involving upper limbs with multiple parts of lower limbs
T04.400x001	Multiple crush injuries of upper and lower limbs
T04.400x011	Upper limb with multiple parts of lower limb thermal crush injury
T04.700	Thorax with crush injury of abdomen, lower back, pelvis and limbs
T04.700x001	Thorax with compression injury of abdomen, lower back and pelvis and extremities
T04.700x011	Thorax with heat crush injury to abdomen, lower back, pelvis and extremities
T04.800	Crushing injury involving other complex parts of the body
T04.800x001	Crushing injury of compound body parts
T04.900	Multiple crushing injuries
T04.900x011	Multiple thermal crush injury
T04.900x012	Systemic thermal crush injury

T04.901	Systemic crush injury
T05.000	Traumatic amputation of hands
T05.100	Traumatic amputation of one hand and the other arm [at any level except for the other hand]
T05.100x001	Traumatic amputation of hand and contralateral arm
T05.200	Traumatic amputation of both arms [at any level]
T05.200x001	Traumatic amputation of both arms
T05.300	Traumatic amputation of both feet
T05.300x002	Partial traumatic amputation of both feet
T05.400	Traumatic amputation of one foot and the other leg [at any level except foot]
T05.400x001	Traumatic amputation of foot and opposite leg
T05.500	Traumatic amputation of both lower legs [at any level]
T05.500x001	Traumatic amputation of both lower legs
T05.600	Traumatic amputation of any combination of upper and lower limbs [at any level]
T05.600x001	Traumatic amputation of upper and lower limbs
T05.800	Traumatic amputation involving other complex parts of the body
T05.800x001	Thoracic traumatic amputation
T05.800x002	Traumatic abdominal amputation
T05.800x003	Traumatic amputation of complex body parts
T05.900	Multiple traumatic amputations
T06.000	Brain nerve injury with nerve and spinal cord injury at the cervical level
T06.000x001	Brain nerve injury with cervical nerve and spinal cord injury
T06.100	Nerve and spinal cord injuries involving other parts of the body
T06.100x001	Multiple nerve and spinal cord injuries
T06.101	Peripheral spinal nerve injury
T06.200	Nerve injuries involving multiple parts of the body
T06.200x001	Multiple nerve injuries
T06.300	Vascular injuries involving multiple parts of the body
T06.300x001	Multiple vascular injuries
T06.400	Muscle and tendon injuries involving multiple parts of the body
T06.400x001	Multiple muscle and tendon injuries
T06.400x002	Multiple muscle injuries
T06.401	Multiple tendon injuries
T06.500	Thoracic organs with damage to abdominal and pelvic organs
T06.500x001	Open injury of intrathoracic organs with abdominal and pelvic organs
T06.500x002	Intrathoracic organs with abdominal and pelvic organ damage
T06.501	Multiple organ injury
T06.800	Other specific injuries involving multiple parts of the body
T06.800x001	Injuries in complex parts of the body
T07.x00	Multiple damages
T08.x00	Spinal fracture
T08.x10	Open spinal fracture
T09.000	Superficial injury of trunk
T09.000x011	Superficial abrasion of trunk
T09.000x021	Superficial blister of trunk
T09.000x031	Superficial insect bite of trunk
T09.000x041	Foreign body on superficial trunk
T09.000x051	Superficial contusion of trunk
T09.100	Open wound of trunk
T09.100x001	Open injury of trunk
T09.200	Dislocation, sprain and strain of trunk joints and ligaments
T09.200x001	Dislocation of trunk joints and ligaments
T09.200x002	Dislocation of trunk joint
T09.200x003	Dislocation of trunk ligament
T09.200x004	Torso joint and ligament sprain
T09.200x005	Torso joint sprain
T09.200x006	Sprain of trunk ligament
T09.200x007	Injury of trunk joints and ligaments
T09.200x008	Torso joint injury
T09.200x009	Injury of trunk ligament
T09.300	Spinal cord injury
T09.300x003	Complete spinal cord injury
T09.300x004	Central spinal cord injury syndrome
T09.300x005	Anterior cord syndrome
T09.300x006	Posterior cord syndrome
T09.300x007	Spinal hematoma
T09.301	Traumatic paraplegia
T09.400	Injury of trunk nerve, spinal nerve root and nerve plexus
T09.400x001	Spinal nerve injury
T09.400x002	Injury of spinal nerve root
T09.400x003	Injury of spinal nerve plexus

T09.500	Injury of trunk muscles and tendons
T09.500x001	Injury of trunk muscles and tendons
T09.500x002	Injury of trunk muscle
T09.500x003	Injury of trunk tendon
T09.600	Traumatic amputation of trunk
T09.800	Other specific injuries to the trunk
T09.900	Torso injury
T10.x00	Fracture of upper limb
T10.x10	Open fracture of upper limb
T11.000	Superficial injury of upper limb
T11.000x011	Superficial abrasion of upper limb
T11.000x021	Superficial vesicles of upper limbs
T11.000x031	Upper limb superficial insect bite
T11.000x041	Superficial foreign body of upper limb
T11.000x051	Superficial contusion of upper limb
T11.001	Upper limb abrasions
T11.100	Open wound of upper limb
T11.101	Skin laceration of upper limb
T11.102	Avulsion injury of upper limb
T11.200	Dislocation, sprain and strain of joints and ligaments of upper limbs
T11.200x001	Dislocation of joints and ligaments of upper limbs
T11.200x002	Dislocation of upper limb joint
T11.200x003	Dislocation of upper limb ligaments
T11.200x004	Sprain of joints and ligaments of upper limbs
T11.200x005	Sprain of upper limb joint
T11.200x006	Sprain of upper limb ligament
T11.200x007	Injuries of joints and ligaments of upper limbs
T11.200x008	Upper limb joint injury
T11.200x009	Ligament injury of upper limb
T11.300	Injury of upper limb nerves
T11.400	Injury of upper limb blood vessels
T11.500	Injury of upper limb muscles and tendons
T11.500x002	Upper limb muscle injury
T11.500x003	Upper limb tendon injury
T11.600	Traumatic amputation of upper limb
T11.600x001	Traumatic amputation of arm
T11.800	Other specific injuries of upper limbs
T11.900	Upper extremity injury

T12.x00	Lower limb fracture
T12.x10	Open fracture of lower limb
T13.000	Superficial injury of lower limb
T13.000x011	Superficial abrasion of lower limb
T13.000x021	Superficial vesicles of lower limbs
T13.000x031	Lower limb superficial insect bite
T13.000x041	Superficial foreign body of lower limb
T13.000x051	Superficial contusion of lower limbs
T13.001	Hematoma of lower limbs
T13.100	Open wound of lower limb
T13.100x003	Avulsion injury of lower limb
T13.100x004	Exfoliation injury of lower limb
T13.101	Lower limb skin laceration
T13.200	Dislocation, sprain and strain of joints and ligaments of lower limbs
T13.200x002	Lower limb joint dislocation
T13.200x003	Dislocation of ligaments of lower limbs
T13.200x005	Lower limb joint sprain
T13.200x006	Lower limb ligament sprain
T13.200x007	Injuries of joints and ligaments of lower limbs
T13.200x008	Lower limb joint injury
T13.200x009	Lower limb ligament injury
T13.201	Dislocation of joints and ligaments of lower limbs
T13.202	Sprain of joints and ligaments of lower limbs
T13.203	Strain of joints and ligaments of lower limbs
T13.300	Nerve injury of lower limbs
T13.400	Injury of lower limb blood vessels
T13.500	Injury of lower limb muscles and tendons
T13.501	Lower limb muscle injury
T13.502	Lower limb tendon injury
T13.600	Traumatic amputation of lower limb
T13.800	Other specific injuries of lower limbs
T13.900	Lower limb injury
T14.000	Superficial damage
T14.000x001	Superficial body injury
T14.000x002	Superficial haematoma of the body
T14.000x003	Surfer tubercle
T14.000x011	Superficial body abrasion
T14.000x021	Superficial blister
T14.000x031	Superficial insect bite

T14.000x041	Superficial foreign body
T14.000x051	Superficial body contusion
T14.001	skin contusion
T14.002	Non toxic spider bite
T14.003	Subcutaneous hematoma
T14.100	Open wound
T14.101	Skin laceration
T14.200	Body fracture
T14.210	Open fracture
T14.300	Dislocation, sprain and strain
T14.400	Nerve injury
T14.500	Vascular injury
T14.501	Traumatic aneurysm
T14.600	Tendon and muscle injuries
T14.601	Tendon injury
T14.602	Muscle injury
T14.700	Crushing injury and traumatic amputation
T14.701	Crush injury
T14.702	Traumatic amputation
T14.800	Other damages
T14.900	damage
T15.000	Corneal foreign body
T15.100	Foreign body of conjunctival capsule
T15.100x001	Foreign body of conjunctiva
T15.100x002	Non traumatic eyelid foreign body
T15.101	Foreign body of eyelid
T15.800	Foreign bodies in other and multiple parts of the external eye
T15.800x001	Foreign body of eyeball
T15.800x002	Foreign body in lacrimal point
T15.800x003	Multiple foreign bodies in the external eye
T15.801	Multiple foreign bodies in the external eye
T15.900	Foreign body of external eye
T16.x00	Intraauricular foreign body
T16.x00x001	foreign bodies
T16.x00x002	Foreign body in middle ear
T17.000	Foreign body in sinus
T17.001	Foreign body in maxillary sinus
T17.002	Foreign body in ethmoid sinus
T17.100	Foreign body in nostril
T17.101	Foreign body in nasal cavity
T17.200	Foreign body in pharynx
T17.200x001	Foreign body in nasopharynx
T17.300	Foreign body in larynx

T17.400	Foreign body in trachea
T17.500	Foreign body in bronchus
T17.501	plastic bronchitis
T17.800	Foreign bodies in other and multiple parts of respiratory tract
T17.801	Multiple foreign body in respiratory tract
T17.802	Foreign body in bronchiole
T17.803	Pulmonary mucus embolism
T17.804	Foreign body in lung
T17.900	Foreign body in respiratory tract
T17.901	Asphyxia due to inhalation of foreign matters
T18.000	Intraoral foreign body
T18.001	Foreign body in oral soft tissue
T18.002	Foreign body of tongue
T18.100	Foreign body in esophagus
T18.200	Intragastric foreign body
T18.300	Foreign body in small intestine
T18.300x003	Foreign body in jejunum
T18.301	Foreign body of duodenum
T18.400	Foreign body in colon
T18.500	Foreign body in anus and rectum
T18.500x004	Foreign body in rectosigmoid junction
T18.501	Intraanal foreign body
T18.502	Foreign body in rectum
T18.800	Foreign bodies in other and multiple parts of digestive tract
T18.801	Multiple foreign bodies in digestive tract
T18.900	Foreign body in digestive tract
T19.000	Foreign body in urethra
T19.100	Foreign body in bladder
T19.200	Foreign body in vulva and vagina
T19.201	Foreign body of vulva
T19.202	Foreign body in vagina
T19.300	Intrauterine foreign body [any part]
T19.800	Foreign bodies in other and multiple parts of urogenital tract
T19.800x001	Foreign body in ureter
T19.800x002	Foreign body in penis
T19.801	Multiple foreign bodies in genitourinary tract
T19.900	Foreign body in genitourinary tract
T20.000	Burns to head and neck
T20.000x002	Burn of head
T20.000x003	Neck burn
T20.000x004	Scalp burn

T20.000x006	Nose burn
T20.000x007	Temporal burn
T20.000x008	Burn of lips
T20.000x010	Eye with head burn
T20.000x011	Eye with neck burn
T20.000x012	Eye with facial burn
T20.000x013	Ear scald
T20.000x014	Facial scald
T20.000x015	Chemical burns on the face
T20.000x021	Scald of head and neck
T20.000x022	Chemical burns to head and neck
T20.002	Ear burn
T20.003	Facial burn
T20.100	First degree burn of head and neck
T20.100x002	First degree burn of head
T20.100x003	First degree burn of neck
T20.100x004	First degree burn of scalp
T20.100x005	First degree facial burn
T20.100x006	First degree burn of nose
T20.100x007	First degree burn of temporal region
T20.100x008	First degree burn of lip
T20.100x009	First degree burn of ear
T20.100x010	Eye with first degree burn of head
T20.100x011	First degree burn of eye with neck
T20.100x012	First degree burn of eye and face
T20.100x021	First degree scald of head and neck
T20.100x022	First degree chemical burn of head and neck
T20.200	Second degree burn of head and neck
T20.200x002	Second degree burn of head
T20.200x003	Second degree burn of neck
T20.200x004	Second degree burn of scalp
T20.200x006	Second degree burn of nose
T20.200x007	Second degree burn of temporal region
T20.200x008	Second degree burn of lip
T20.200x009	Second degree burn of ear
T20.200x010	Eye with second degree burn of head
T20.200x011	Second degree burn of eye with neck
T20.200x012	Second degree burn of eye with face
T20.200x021	Second degree scald of head and neck
T20.200x022	Second degree chemical burn of head and neck
T20.200x023	Second degree facial scald
T20.200x024	Second degree chemical burn on face
T20.201	Second degree burn of face
T20.300	Third degree burn of head and neck
T20.300x002	Third degree burn of head
T20.300x003	Third degree burn of neck
T20.300x004	Third degree burn of scalp
T20.300x005	Third degree facial burn
T20.300x006	Third degree burn of nose
T20.300x007	Third degree burn of temporal region
T20.300x008	Third degree burn of lip
T20.300x009	Third degree burn of ear
T20.300x010	Eye with third degree burn of head
T20.300x011	Third degree burn of eye with neck
T20.300x012	Third degree burn of eye with face
T20.300x021	Third degree scald of head and neck
T20.300x022	Third degree chemical burn of head and neck
T20.400	Head and neck corrosion
T20.400x002	Head corrosion
T20.400x003	Corrosive injury of neck
T20.400x004	Scalp corrosion
T20.400x005	Facial erosion
T20.400x006	Nasal corrosion
T20.400x007	Temporal corrosion
T20.400x008	Lip corrosion
T20.400x009	Ear corrosion
T20.400x010	Eye with head corrosion
T20.400x011	Eye with corrosion injury of neck
T20.400x012	Eye with surface corrosion
T20.401	Chemical burn of ear
T20.500	First degree corrosion injury of head and neck
T20.500x002	Head was once corroded
T20.500x003	First degree corrosion wound on neck
T20.500x004	Once corroded scalp
T20.500x005	Once corrosive wound on face
T20.500x006	Once corrosive wound on nose
T20.500x007	Temporal corrosion
T20.500x008	Once corroded lips
T20.500x009	Ear was once corroded
T20.500x010	Primary corrosion injury of eye and head
T20.500x011	First degree corrosion injury of eye and neck
T20.500x012	One degree of corrosion injury on eye companion surface

T20.600	Second degree corrosion injury of head and neck
T20.600x002	Second degree corrosion of head
T20.600x003	Secondary corrosion injury of neck
T20.600x004	Second degree corrosion injury of scalp
T20.600x005	Second degree corrosion injury on face
T20.600x006	Second degree corrosion wound of nose
T20.600x007	Secondary corrosion injury of temporal region
T20.600x008	Secondary corrosion injury of lip
T20.600x009	Second degree corrosion injury of ear
T20.600x010	Eye with secondary corrosion injury of head
T20.600x011	Eye with secondary corrosion injury of neck
T20.600x012	Secondary corrosion injury of eye associated surface
T20.700	Third degree corrosion injury of head and neck
T20.700x002	Third degree corrosion of head
T20.700x003	Third degree corrosion injury of neck
T20.700x004	Third degree corrosion of scalp
T20.700x005	Third degree facial corrosion
T20.700x006	Third degree corrosion wound of nose
T20.700x007	Third degree corrosion injury of temporal region
T20.700x008	Third degree corrosion injury on lips
T20.700x009	Third degree corrosion injury of ear
T20.700x010	Eye with third degree corrosion injury of head
T20.700x011	Eye with third degree corrosion injury of neck
T20.700x012	Third degree corrosion injury of eye associated surface
T21.000	Burn of trunk
T21.000x011	Burn of breast
T21.000x021	Burn of chest wall
T21.000x031	Abdominal wall burn
T21.000x032	Hypochondriac burn
T21.000x033	Inguinal burn
T21.000x041	Bum of buttock
T21.000x042	Back burn
T21.000x043	Burn of interscapular region
T21.000x051	Burn of labia majora
T21.000x052	Burn of labia minora
T21.000x053	Burn of penis
T21.000x054	Perineal burn
T21.000x055	Scrotal burn
T21.000x056	Testicular burn
T21.000x057	Vulvar burn
T21.000x091	Anal burn
T21.100	First degree burn of trunk
T21.100x011	First degree burn of breast
T21.100x021	First degree burn of chest wall
T21.100x031	First degree burn of abdominal wall
T21.100x032	First degree burn of flank
T21.100x033	First degree burn of groin
T21.100x041	First degree burn of buttock
T21.100x042	First degree burn of back
T21.100x043	First degree burn of interscapular region
T21.100x051	First degree burn of labia majora
T21.100x052	First degree burn of labia minora
T21.100x053	First degree burn of penis
T21.100x054	First degree burn of perineum
T21.100x055	First degree burn of scrotum
T21.100x056	First degree burn of testis
T21.100x057	First degree burn of vulva
T21.100x091	First degree burn of anus
T21.100x101	Once scalded trunk
T21.100x102	One degree of chemical burn on the trunk
T21.200	Second degree burn of trunk
T21.200x011	Second degree burn of breast
T21.200x021	Second degree burn of chest wall
T21.200x031	Second degree burn of abdominal wall
T21.200x032	Second degree burn of flank
T21.200x033	Second degree burn of groin
T21.200x041	Second degree burn of buttock
T21.200x042	Second degree burn of back
T21.200x043	Second degree burn of interscapular region
T21.200x051	Second degree burn of labia majora
T21.200x052	Second degree burn of labia minora
T21.200x053	Second degree burn of penis
T21.200x054	Second degree burn of perineum
T21.200x055	Second degree burn of scrotum
T21.200x056	Second degree burn of testis
T21.200x057	Second degree burn of vulva
T21.200x091	Second degree burn of anus
T21.200x101	Second degree scald of trunk
T21.200x102	Second degree chemical burn of trunk

T21.300	Third degree burn of trunk
T21.300x011	Third degree burn of breast
T21.300x021	Third degree burn of chest wall
T21.300x031	Third degree burn of abdominal wall
T21.300x032	Third degree burn of flank
T21.300x033	Third degree burn of groin
T21.300x041	Third degree burn of buttock
T21.300x042	Third degree burn of back
T21.300x043	Third degree burn of interscapular region
T21.300x051	Third degree burn of labia majora
T21.300x052	Third degree burn of labia minora
T21.300x053	Third degree burn of penis
T21.300x054	Third degree burn of perineum
T21.300x055	Third degree burn of scrotum
T21.300x056	Third degree burn of testis
T21.300x057	Third degree burn of vulva
T21.300x091	Third degree burn of anus
T21.300x101	Third degree scald of trunk
T21.300x102	Third degree chemical burn of trunk
T21.400	Torso corrosion
T21.400x011	Corrosive wound of breast
T21.400x021	Thoracic wall corrosion
T21.400x031	Corrosive wound of abdominal wall
T21.400x032	Flank corrosion
T21.400x033	Inguinal corrosion
T21.400x041	Hip corrosion injury
T21.400x042	Back corrosion
T21.400x043	Corrosion wound of scapular region
T21.400x051	Corrosion of labia majora
T21.400x052	Corrosion wound of labia minora
T21.400x053	Penis corrosion
T21.400x054	Perineal corrosion
T21.400x055	Corrosive wound of scrotum
T21.400x056	Testicular corrosion
T21.400x057	Vulva corrosion
T21.400x091	Erosion of anus
T21.500	One degree of corrosion injury to the trunk
T21.500x011	Once corrosive wound of breast
T21.500x021	Once corrosive wound of chest wall
T21.500x031	First degree corrosion of abdominal wall
T21.500x032	One degree corrosion damage on flank
T21.500x033	One degree of corrosion of groin

T21.500x041	Once corrosive wound on hip
T21.500x042	One degree of corrosion on the back
T21.500x043	One degree corrosion wound in the interscapular region
T21.500x051	The labia majora was once corroded
T21.500x052	The labia minora was once corroded
T21.500x053	Once corroded penis
T21.500x054	First degree corrosion of perineum
T21.500x055	Once corrosive wound of scrotum
T21.500x056	First degree corrosion injury of testis
T21.500x057	One degree of vulva corrosion
T21.500x091	Once corrosive wound of anus
T21.600	Second degree corrosion injury of trunk
T21.600x011	Secondary corrosion injury of breast
T21.600x021	Secondary corrosion injury of chest wall
T21.600x031	Second degree corrosion of abdominal wall
T21.600x032	Secondary corrosion of flank
T21.600x033	Second degree corrosion of groin
T21.600x041	Second degree corrosion injury of hip
T21.600x042	Second degree corrosion on the back
T21.600x043	Second degree corrosion injury of scapular region
T21.600x051	Second degree corrosion of labia majora
T21.600x052	Secondary corrosion of labia minora
T21.600x053	Secondary corrosion injury of penis
T21.600x054	Secondary corrosion injury of perineum
T21.600x055	Secondary corrosion wound of scrotum
T21.600x056	Secondary corrosion injury of testis
T21.600x057	Secondary corrosion of vulva
T21.600x091	Second degree corrosion wound of anus
T21.700	Third degree corrosion injury of trunk
T21.700x011	Third degree corrosion wound of breast
T21.700x021	Third degree corrosion injury of chest wall
T21.700x031	Third degree corrosion of abdominal wall
T21.700x032	Third degree corrosion of flank
T21.700x033	Third degree corrosion of groin

T21.700x041	Third degree corrosion injury of hip
T21.700x042	Third degree corrosion on the back
T21.700x043	Third degree corrosion wound of scapular region
T21.700x051	Third degree corrosion of labia majora
T21.700x052	Third degree corrosion of labia minora
T21.700x053	Third degree corrosion injury of penis
T21.700x054	Third degree corrosion of perineum
T21.700x055	Third degree corrosion wound of scrotum
T21.700x056	Third degree corrosion injury of testis
T21.700x057	Third degree corrosion of vulva
T21.700x091	Third degree corrosion wound of anus
T22.000	Burns to shoulders and upper limbs, except wrists and hands
T22.000x001	Burns of shoulder and upper limbs
T22.000x002	Upper extremity burn
T22.000x003	Shoulder burn
T22.000x004	Burn of scapular region
T22.000x005	Arm burn
T22.000x006	Axillary burn
T22.100	First degree burn of shoulder and upper limb, except wrist and hand
T22.100x001	First degree burn of shoulder and upper limb
T22.100x002	First degree burn of upper limb
T22.100x003	First degree burn of shoulder
T22.100x004	First degree burn of scapular region
T22.100x005	First degree burn of arm
T22.100x006	First degree burn of axilla
T22.100x011	Once scalded shoulder and upper limb
T22.100x012	One degree chemical burn of shoulder and upper limb
T22.200	Second degree burn of shoulder and upper limb, except wrist and hand
T22.200x001	Second degree burn of shoulder and upper limb
T22.200x002	Second degree burn of upper limb
T22.200x003	Second degree burn of shoulder
T22.200x004	Second degree burn of scapular region
T22.200x005	Second degree burn of arm
T22.200x006	Second degree burn of axilla
T22.200x011	Second degree scald of shoulder and upper limb
T22.200x012	Second degree chemical burn of shoulder and upper limb
T22.300	Third degree burn of shoulder and upper limb, except wrist and hand
T22.300x001	Third degree burn of shoulder and upper limb
T22.300x002	Third degree burn of upper limb
T22.300x003	Third degree burn of shoulder
T22.300x004	Third degree burn of scapular region
T22.300x005	Third degree burn of arm
T22.300x006	Third degree burn of axilla
T22.300x011	Third degree scald of shoulder and upper limb
T22.300x012	Third degree chemical burn of shoulder and upper limb
T22.400	Corrosive injuries to shoulders and upper limbs, except wrists and hands
T22.400x001	Corrosion injury of shoulder and upper limb
T22.400x002	Corrosion injury of upper limb
T22.400x003	Shoulder corrosion
T22.400x004	Corrosion injury of scapular region
T22.400x005	Arm corrosion
T22.400x006	Axillary corrosion
T22.500	Once corrosive injury to shoulder and upper limb, except wrist and hand
T22.500x001	Once corrosive wound on shoulder and upper limb
T22.500x002	First degree corrosion injury of upper limb
T22.500x003	Shoulder once corroded
T22.500x004	One degree corrosion wound in scapular region
T22.500x005	One degree corrosion of arm
T22.500x006	First degree corrosion injury of axilla
T22.600	Second degree corrosion injury of shoulder and upper limb, except wrist and hand
T22.600x001	Second degree corrosion injury of shoulder and upper limb
T22.600x002	Second degree corrosion injury of upper limb
T22.600x003	Second degree corrosion of shoulder
T22.600x004	Secondary corrosion injury of scapular region
T22.600x005	Second degree corrosion of arm
T22.600x006	Secondary corrosion injury of axilla

T22.700	Third degree corrosion injury to shoulder and upper limb, except wrist and hand
T22.700x001	Third degree corrosion injury of shoulder and upper limb
T22.700x002	Third degree corrosion injury of upper limb
T22.700x003	Third degree corrosion of shoulder
T22.700x004	Third degree corrosion injury of scapular region
T22.700x005	Third degree corrosion of arm
T22.700x006	Third degree corrosion injury of axilla
T23.000	Burns of wrists and hands of unspecified degree
T23.000x001	Wrist and hand burns
T23.000x002	Burn of wrist
T23.000x003	Hand burns
T23.000x004	Palm burn
T23.000x005	Thumb burn
T23.000x006	Finger burn
T23.000x007	Nail burn
T23.100	First degree burn of wrist and hand
T23.100x002	First degree burn of wrist
T23.100x003	First degree burn of hand
T23.100x004	First degree burn of palm
T23.100x005	First degree burn of thumb
T23.100x006	First degree burn of fingers
T23.100x007	First degree burn of nail
T23.100x011	Wrist and hand once scalded
T23.100x012	First degree chemical burns on wrist and hand
T23.200	Second degree burn of wrist and hand
T23.200x002	Second degree burn of wrist
T23.200x003	Second degree burn of hand
T23.200x004	Second degree burn of palm
T23.200x005	Second degree burn of thumb
T23.200x006	Second degree burn of finger
T23.200x007	Second degree burn of nail
T23.200x011	Second degree scald of wrist and hand
T23.200x012	Second degree chemical burn of wrist and hand
T23.300	Third degree burn of wrist and hand
T23.300x002	Third degree burn of wrist
T23.300x003	Third degree burn of hand
T23.300x004	Third degree burn of palm
T23.300x005	Third degree burn of thumb
T23.300x006	Third degree burn of finger
T23.300x007	Third degree burn of nail

T23.300x011	Third degree scald of wrist and hand
T23.300x012	Third degree chemical burn of wrist and hand
T23.300x013	Deep third degree burn of wrist and hand
T23.300x014	Third degree deep scald of wrist and hand
T23.300x015	Deep third degree chemical burn of wrist and hand
T23.400	Wrist and hand corrosion
T23.400x002	Wrist corrosion
T23.400x003	Hand corrosion
T23.400x004	Palm corrosion
T23.400x005	Corrosion injury of thumb
T23.400x006	Finger corrosion
T23.400x007	Nail corrosion
T23.500	Wrist and hand once corroded
T23.500x002	Wrist was once corroded
T23.500x003	Hand was once corroded
T23.500x004	Palm was once corroded
T23.500x005	Once corroded thumb
T23.500x006	Finger once corroded
T23.500x007	Nail once corroded
T23.600	Secondary corrosion injury of wrist and hand
T23.600x002	Second degree corrosion injury of wrist
T23.600x003	Second degree corrosion injury of hand
T23.600x004	Second degree corrosion injury of palm
T23.600x005	Second degree corrosion injury of thumb
T23.600x006	Second degree corrosion of fingers
T23.600x007	Secondary corrosion of nail
T23.700	Third degree corrosion injury of wrist and hand
T23.700x002	Third degree corrosion injury of wrist
T23.700x003	Third degree corrosion injury of hand
T23.700x004	Third degree corrosion of palm
T23.700x005	Third degree corrosion injury of thumb
T23.700x006	Third degree corrosion of fingers
T23.700x007	Third degree corrosion of nail
T24.000	Burns of the hip and lower limbs, except the ankle and foot
T24.000x001	Hip and lower limb burns
T24.000x002	Hip burn
T24.000x003	Burn of lower limbs
T24.000x004	Calf burn

T24.100	First degree burn of the hip and lower limbs, except the ankle and foot
T24.100x001	First degree burn of hip and lower limb
T24.100x002	First degree burn of hip
T24.100x003	First degree burn of lower limb
T24.100x004	First degree burn of lower leg
T24.100x011	Once scalded hip and lower limbs
T24.100x012	First degree chemical burn of hip and lower limb
T24.200	Second degree burn of hip and lower limb, except ankle and foot
T24.200x001	Second degree burn of hip and lower limb
T24.200x002	Second degree burn of hip
T24.200x003	Second degree burn of lower limb
T24.200x004	Second degree burn of lower leg
T24.200x011	Second degree scald of hip and lower limb
T24.200x012	Second degree chemical burn of hip and lower limb
T24.300	Third degree burn of hip and lower limb, except ankle and foot
T24.300x001	Third degree burn of hip and lower limb
T24.300x002	Third degree burn of hip
T24.300x003	Third degree burn of lower limb
T24.300x004	Third degree burn of lower leg
T24.300x011	Third degree scald of hip and lower limb
T24.300x012	Third degree chemical burn of hip and lower limb
T24.400	Corrosion injury of hip and lower limb, except ankle and foot
T24.400x001	Corrosion injury of hip and lower limb
T24.400x002	Hip corrosion
T24.400x003	Lower limb corrosion injury
T24.400x004	Crural corrosion
T24.500	Once corrosive injury of hip and lower limb, except for ankle and foot
T24.500x001	First degree corrosion injury of hip and lower limb
T24.500x002	One time corrosion wound of hip
T24.500x003	First degree corrosion injury of lower limb
T24.500x004	One time corrosion injury of lower leg
T24.600	Second degree corrosion injury of hip and lower limb, except ankle and foot
T24.600x001	Second degree corrosion injury of hip and lower limb
T24.600x002	Second degree corrosion injury of hip
T24.600x003	Secondary corrosion injury of lower limb
T24.600x004	Secondary corrosion injury of lower leg
T24.700	Third degree corrosion injury of hip and lower limb, except ankle and foot
T24.700x001	Third degree corrosion injury of hip and lower limb
T24.700x002	Third degree corrosion injury of hip
T24.700x003	Third degree corrosion injury of lower limb
T24.700x004	Third degree corrosion injury of lower leg
T25.000	Burns to ankle and foot
T25.000x002	Burn of ankle
T25.000x003	Foot burn
T25.100	First degree burn of ankle and foot
T25.100x002	First degree burn of ankle
T25.100x003	First degree burn of foot
T25.100x011	Once scalded ankle and foot
T25.100x012	First degree chemical burn of ankle and foot
T25.200	Second degree burn of ankle and foot
T25.200x002	Second degree burn of ankle
T25.200x003	Second degree burn of foot
T25.200x011	Second degree scald of ankle and foot
T25.200x012	Secondary chemical burn of ankle and foot
T25.300	Third degree burn of ankle and foot
T25.300x002	Third degree burn of ankle
T25.300x003	Third degree burn of foot
T25.300x011	Third degree scald of ankle and foot
T25.300x012	Third degree chemical burn of ankle and foot
T25.400	Corrosion injury of ankle and foot
T25.400x002	Corrosion injury of ankle
T25.400x003	Foot corrosion
T25.500	First degree corrosion injury of ankle and foot
T25.500x002	Once corroded ankle
T25.500x003	One time corrosion injury on foot
T25.600	Secondary corrosion injury of ankle and foot
T25.600x002	Second degree corrosion injury of ankle
T25.600x003	Second degree corrosion injury of foot

T25.700	Third degree corrosion injury of ankle and foot
T25.700x002	Third degree corrosion injury of ankle
T25.700x003	Third degree corrosion injury of foot
T26.000	Burns of eyelid and periocular area
T26.001	Eyelid burn
T26.001x011	Scald of eyelid
T26.001x012	Chemical burn of eyelid
T26.001x013	Scald around eyes
T26.001x014	Chemical burns around the eye
T26.002	Burn of periocular region
T26.100	Burns of cornea and conjunctival sac
T26.100x001	Burns of cornea and conjunctiva
T26.100x003	Conjunctival burn
T26.101	Corneal burn
T26.102	Burn of conjunctival sac
T26.200	Burn accompanied by eyeball rupture and destruction
T26.200x001	Eye burn with eyeball rupture
T26.300	Burns to other parts of eyes and appendages
T26.301	Scleral burn
T26.400	Burns of eyes and appendages
T26.400x001	Ocular burn
T26.400x011	Eye and accessory burns
T26.400x012	Chemical burns to eyes and appendages
T26.400x013	Ocular scald
T26.400x014	Chemical burn of eyeball
T26.401	Eyeball burn
T26.500	Corrosive wound of eyelid and periocular area
T26.500x002	Eyelid corrosion
T26.500x003	Periocular corrosion
T26.600	Corrosive wound of cornea and conjunctival capsule
T26.600x001	Corrosive wound of cornea and conjunctiva
T26.600x002	Corneal erosion
T26.600x003	Conjunctival corrosion
T26.601	Acid burn of cornea with conjunctiva
T26.602	Chemical burn of cornea
T26.603	Alkaline burn of cornea
T26.604	Acid burn of cornea
T26.605	Acid burn of conjunctiva
T26.700	Corrosive injury accompanied by eyeball rupture and destruction
T26.700x001	Eye corrosion injury with eyeball rupture

T26.800	Other parts of eyes and appendages are corroded
T26.800x001	Scleral corrosion
T26.900	Eye and accessory corrosion
T26.900x001	Eye corrosion
T26.901	Acid burn of eyeball
T26.902	Alkaline burn of eyeball
T27.000	Burns of larynx and trachea
T27.000x002	Laryngeal burn
T27.000x003	Trachea burn
T27.100	Burns involving larynx, trachea and lung
T27.100x001	Burns of larynx, trachea and lung
T27.200	Burns in other parts of the respiratory tract
T27.200x001	Thoracic burn
T27.300	Respiratory tract burn
T27.400	Corrosion injury of throat and trachea
T27.400x002	Throat corrosion
T27.400x003	Corrosion injury of trachea
T27.401	Chemical burn of larynx
T27.402	Chemical burn of trachea
T27.500	Corrosive injury involving throat, trachea and lung
T27.500x001	Corrosive injury of larynx, trachea and lung
T27.600	Corrosion injury of other parts of respiratory tract
T27.600x001	Thoracic corrosion injury
T27.700	Corrosive injury of respiratory tract
T28.000	Burns of mouth and pharynx
T28.000x002	Oral burn
T28.000x003	Pharyngeal burn
T28.100	Esophageal burn
T28.200	Burns in other parts of digestive tract
T28.200x001	Gastric burn
T28.200x002	Burn of digestive tract
T28.300	Internal burn of genitourinary organs
T28.300x001	Burns of vagina and uterus
T28.300x002	Vaginal burn
T28.300x003	Uterine burn
T28.400	Burns of internal organs, other and unspecified
T28.401	Burn of internal organs
T28.500	Corrosive wound of mouth and pharynx
T28.500x002	Oral erosion
T28.500x003	Pharyngeal corrosion
T28.501	Chemical burn of oral mucosa

T28.502	Chemical burn of pharynx
T28.600	Esophageal corrosion injury
T28.700	Corrosion injury in other parts of digestive tract
T28.700x001	Gastric corrosion
T28.700x002	Corrosive injury of digestive tract
T28.701	Chemical burn of stomach
T28.702	Corrosive injury of intestine
T28.800	Internal corrosion of genitourinary organs
T28.800x001	Corrosive wound of vagina and uterus
T28.800x002	Corrosive wound of vagina
T28.800x003	Erosive wound of uterus
T28.900	Corrosion of internal organs, others
T28.900x001	Internal organ corrosion
T28.901	Chemical burn of internal organs
T29.000	Burns at multiple sites
T29.000x001	Multiple burns
T29.100	Burns on multiple parts, and the mentioned burns do not exceed one degree
T29.100x001	Multiple first degree burns
T29.100x011	Several first degree burns
T29.200	Burns on multiple parts, and the mentioned burns are not more than two degrees
T29.200x001	Multiple second degree burns
T29.200x011	Multiple secondary burns
T29.300	Burns on multiple parts, at least one third degree burn
T29.300x001	Multiple third degree burns
T29.300x011	Multiple burns At least one third degree burn
T29.400	Corrosion damage of multiple parts
T29.400x001	Multiple corrosion damages
T29.500	Corrosion damage of multiple parts, and the corrosion damage mentioned shall not exceed one degree
T29.500x001	Several primary corrosion damages
T29.600	Corrosion damage at multiple parts, and the corrosion damage mentioned shall not exceed two degrees
T29.600x001	Multiple secondary corrosion injuries
T29.700	Corrosion damage at multiple parts, and at least one third degree corrosion damage
T29.700x001	Multiple third degree corrosion injuries

T30.000	Body burns
T30.000x001	burn
T30.100	First degree burn
T30.100x011	First degree scald
T30.200	Second degree burn
T30.200x011	Second degree scald
T30.300	Third degree burn
T30.300x011	Third degree scald
T30.400	Body corrosion
T30.400x001	Corrosion damage
T30.500	First degree corrosion damage
T30.600	Secondary corrosion damage
T30.700	Third degree corrosion damage
T31.000	Burns involving less than 10% of body surface
T31.000x001	Burns on body surface less than 10%
T31.100	Burns involving 10%~19% of body surface
T31.100x001	Burns on body surface 10-19%
T31.200	Burns involving 20%~29% of body surface
T31.200x001	Burns on body surface 20-29%
T31.300	Burns involving 30%~39% of body surface
T31.300x001	Burns of 30-39% of body surface
T31.400	Burns involving 40%~49% of body surface
T31.400x001	40-49% burn on body surface
T31.500	Burns involving 50%~59% of body surface
T31.500x001	Burns on body surface 50-59%
T31.600	Burns involving 60%~69% of body surface
T31.600x001	Burns on body surface 60-69%
T31.700	Burns involving 70%~79% of body surface
T31.700x001	70-79% burn on body surface
T31.800	Burns involving 80%~89% of body surface
T31.800x001	80-89% of body surface burns
T31.900	Burns involving 90% or more of the body surface
T31.900x001	Burns greater than 90% of the body surface
T32.000	Corrosion damage involving less than 10% of body surface
T32.000x001	Corrosion damage on body surface less than 10%
T32.100	Corrosion injury involving 10%~19% of body surface
T32.100x001	10-19% of the body surface is corroded
T32.200	Corrosion damage involving 20%~29% of body surface

T32.200x001	20-29% of the body surface is corroded
T32.300	Corrosion damage involving 30%~39% of body surface
T32.300x001	30-39% of body surface corrosion
T32.400	Corrosion damage involving 40%~49% of body surface
T32.400x001	40-49% of the body surface is corroded
T32.500	Corrosion damage involving 50%~59% of body surface
T32.500x001	50-59% corrosion damage on body surface
T32.600	Corrosion damage involving 60%~69% of body surface
T32.600x001	60-69% of the body surface is corroded
T32.700	Corrosion damage involving 70%~79% of body surface
T32.700x001	70-79% of the body surface is corroded
T32.800	80%~89% corrosion damage on body surface
T32.800x001	80-89% of the body surface is corroded
T32.900	Corrosion damage involving 90% or more of the body surface
T32.900x001	Corrosion damage greater than 90% on the body surface
T33.000	Superficial frostbite of head
T33.100	Superficial frostbite of neck
T33.200	Superficial frostbite of chest
T33.300	Superficial frostbite of abdominal wall, lower back and pelvis
T33.300x001	Superficial frostbite of abdominal wall
T33.300x002	Superficial frostbite of back
T33.300x003	Pelvic superficial frostbite
T33.400	Superficial frostbite of arm
T33.500	Superficial frostbite of wrist and hand
T33.500x002	Superficial frostbite of wrist
T33.500x003	Superficial frostbite of hands
T33.600	Superficial frostbite of hip and thigh
T33.600x002	Superficial frostbite of hip
T33.600x003	Superficial frostbite of thigh
T33.700	Superficial frostbite of knee and lower leg
T33.700x002	Superficial frostbite of knee
T33.700x003	Superficial frostbite of lower leg
T33.800	Superficial frostbite of ankle and foot
T33.800x002	Superficial frostbite of ankle
T33.800x003	Superficial frostbite of foot
T33.900	Superficial frostbite, other and unspecified

T33.900x003	Superficial frostbite of trunk
T33.901	Superficial frostbite
T34.000	Head frostbite with tissue necrosis
T34.100	Cervical frostbite with tissue necrosis
T34.200	Chest frostbite with tissue necrosis
T34.300	Frostbite of abdominal wall, lower back and pelvis with tissue necrosis
T34.300x001	Frostbite of abdominal wall with tissue necrosis
T34.300x002	Frostbite of back with tissue necrosis
T34.300x003	Pelvic frostbite with tissue necrosis
T34.400	Frostbite of arm with tissue necrosis
T34.500	Frostbite of wrist and hand with tissue necrosis
T34.500x002	Frostbite of wrist with tissue necrosis
T34.500x003	Hand frostbite with tissue necrosis
T34.600	Frostbite of hip and thigh with tissue necrosis
T34.600x002	Hip frostbite with tissue necrosis
T34.600x003	Frostbite of thigh with tissue necrosis
T34.700	Frostbite of knee and lower leg with tissue necrosis
T34.700x002	Frostbite of knee with tissue necrosis
T34.700x003	Frostbite of lower leg with tissue necrosis
T34.800	Frostbite of ankle and foot with tissue necrosis
T34.800x002	Frostbite of ankle with tissue necrosis
T34.800x003	Frostbite of foot with tissue necrosis
T34.900	Frostbite with tissue necrosis, other and unspecified
T34.900x002	Frostbite with tissue necrosis
T34.900x003	Frostbite of trunk with tissue necrosis
T35.000	Superficial frostbite involving multiple parts of the body
T35.000x001	Multiple superficial frostbite
T35.100	Frostbite involving multiple parts of the body with tissue necrosis
T35.100x001	Multiple frostbite with tissue necrosis
T35.200	Frostbite of head and neck
T35.300	Frostbite of chest, abdomen, lower back and pelvis
T35.300x001	Frostbite of trunk
T35.300x002	Chest frostbite
T35.300x003	Abdominal frostbite

T35.300x004	Back frostbite
T35.300x005	Pelvic frostbite
T35.300x006	Frostbite of abdominal wall, lower back and pelvis
T35.400	Frostbite of upper limbs
T35.500	Frostbite of lower limbs
T35.600	Frostbite involving multiple parts of the body
T35.600x001	Multiple frostbite
T35.700	frostbite
T35.700x002	Cold injury
T35.700x003	Occupational frostbite
T35.700x004	Systemic cold injury
T35.700x005	Local frostbite
T35.700x006	Local once frostbite
T35.700x007	Local secondary frostbite
T35.700x008	Partial third degree frostbite
T35.700x009	Partial fourth degree frostbite
T36.000	Penicillins Poisoning
T36.100	Cephalosporins and others β Poisoning by lactam antibiotics
T36.100x003	β Poisoning by lactam antibiotics
T36.101	Poisoning by cephalosporins
T36.102	Poisoning by cephalosporin
T36.200	Chloramphenicol poisoning
T36.300	Macrolide poisoning
T36.300x001	Erythromycin poisoning
T36.400	Tetracycline poisoning
T36.500	Poisoning by aminoglycosides
T36.500x003	Streptomycin poisoning
T36.501	Poisoning by amikacin
T36.502	Gentamicin poisoning
T36.600	Rifamycin poisoning
T36.700	Systemic antifungal antibiotic poisoning
T36.800	Systemic antibiotic poisoning, others
T36.900	Systemic antibiotic poisoning
T36.900x001	Antibiotic poisoning
T37.000	Sulfonamide poisoning
T37.100	Poisoning by antimycobacterial drugs
T37.100x001	Poisoning by ramifene
T37.200	Antimalarial and drug poisoning with effect on other blood protozoa
T37.300	Antigen worm drug poisoning, others
T37.300x001	Poisoning by antigen living substance
T37.400	Poisoning by worm repellent

T37.500	Poisoning by antiviral drugs
T37.800	Poisoning by systemic anti infective and antiparasitic drugs, other specific
T37.800x001	Poisoning by hydroxyquinoline derivative
T37.900	Systemic anti infective and antiparasitic poisoning
T37.900x001	Systemic anti infective poisoning
T37.900x002	Systemic antiparasitic poisoning
T38.000	Poisoning by sugar [adrenal] corticosteroids and their synthetic analogues
T38.000x001	Iatrogenic steroid diabetes
T38.100	Poisoning by thyroid hormones and their substitutes
T38.100x001	Poisoning by thyroid hormone and its derivatives
T38.200	Poisoning by antithyroid drugs
T38.300	Insulin and oral hypoglycemia [anti diabetes] drug poisoning
T38.300x001	Iatrogenic hyperinsulinemia
T38.300x003	Poisoning by oral antidiabetic drugs
T38.301	Insulin poisoning
T38.400	Poisoning by oral contraceptives
T38.401	Gossypol poisoning
T38.500	Estrogen and progesterone poisoning, others
T38.500x001	Estrogen poisoning
T38.500x002	Progesterone poisoning
T38.501	Diethylstilbestrol poisoning
T38.600	Poisoning caused by anti gonadotropins, anti estrogen drugs and anti androgen drugs, which cannot be classified elsewhere
T38.600x001	Tamoxifen poisoning
T38.700	Poisoning by androgens and similar drugs that promote metabolism
T38.800	Poisoning by hormones and their synthetic substitutes, other and unspecified
T38.800x001	Poisoning of anterior pituitary hormones
T38.801	Poisoning by hormones and their synthetic substitutes
T38.900	Poisoning by hormone antagonists, other and unspecified
T38.901	Poisoning by hormone antagonists
T39.000	Poisoning by salicylate
T39.100	Poisoning by 4-aminophenol derivative
T39.101	Poisoning by acetaminophen

T39.200	Poisoning by pyrazolone derivative
T39.200x001	Poisoning by metamizole
T39.201	Poisoning by aminopyrine
T39.300	Poisoning by non steroid anti-inflammatory drugs [NSAID]
T39.300x002	Poisoning by tramadol
T39.300x003	Indomethacin poisoning
T39.400	Poisoned by anti rheumatic drugs, which cannot be classified elsewhere
T39.400x001	Poisoning by antirheumatic drugs
T39.800	Poisoning by non opioid analgesics and antipyretics, others, which cannot be classified elsewhere
T39.801	Poisoning by Tongkening
T39.802	Poisoning by the root of kidney bean
T39.900	Poisoning by non opioid analgesics, antipyretics and anti rheumatic drugs
T39.901	Poisoning by non opioid analgesics
T39.902	Antipyretic poisoning
T40.000	Opioid poisoning
T40.100	Heroin poisoning
T40.200	Opioid poisoning, other
T40.200x001	Codeine poisoning
T40.201	Morphine poisoning
T40.300	Poisoning by Meisantong
T40.400	Poisoning by synthetic narcotics, others
T40.400x002	Aristolochia (ten thousand zhang long) poisoning
T40.401	Poisoning by dolantine
T40.500	Cocaine poisoning
T40.600	Poisoning by narcotics, others
T40.601	Poisoning by narcotics
T40.700	Cannabis (derivative) poisoning
T40.700x001	cannabis intoxication
T40.800	Diethyllysergamide [LSD] poisoning
T40.900	Hallucinogen [Hallucinogen] poisoning, other and unspecified
T40.900x001	Poisoning by poisonous alkali of South American cactus
T40.900x002	Poisoning by dimethyl-4-hydroxytryptamine
T40.900x003	Cilosibine poisoning
T40.901	Hallucinogenic drug poisoning
T41.000	Inhalation anesthetic poisoning
T41.100	Poisoning by intravenous anesthetics
T41.100x002	Poisoning by thiobarbital salts

T41.200	Poisoning by general anesthetics, other and unspecified
T41.200x002	Poisoning by ketamine
T41.201	Poisoning by general anesthetic
T41.300	Toxicosis of local Anaesthetic
T41.400	Poisoning by narcotics
T41.500	Therapeutic gas poisoning
T41.500x001	Therapeutic oxygen poisoning
T41.500x003	Therapeutic carbon dioxide poisoning
T41.501	Oxygen poisoning, accident
T42.000	Poisoning by hydantoin derivative
T42.001	Poisoning by phenytoin sodium
T42.100	Poisoning by iminodistyrene
T42.101	Poisoning by carbamazepine
T42.200	Poisoning by succinimide and oxazolidinedione
T42.200x001	Oxazolidine copper poisoning
T42.200x002	Poisoning by succinimide
T42.300	Poisoning by barbiturate salts
T42.301	Poisoning by phenobarbital
T42.302	Tachysomia poisoning
T42.400	Benzenediazepines poisoning
T42.401	Diazepam poisoning
T42.402	Poisoning by Jiajing Diazepam
T42.403	Poisoning by salbutamol
T42.404	Poisoning by Limianning
T42.405	Nitrazepam poisoning
T42.406	Poisoning by clozapine
T42.500	Poisoning by mixed antiepileptic drugs, which cannot be classified elsewhere
T42.500x001	Mixed antiepileptic drug poisoning
T42.600	Poisoning by antiepileptic drugs and sedative hypnotics, others
T42.600x002	Zopiclone poisoning
T42.600x004	Poisoning by sodium valproate
T42.600x005	Valproic acid poisoning
T42.600x006	Poisoning by phenethylpiperidone
T42.601	Poisoning by methaqualone
T42.602	Poisoning by phenanthrol
T42.700	Poisoning by antiepileptic drugs and sedative hypnotics
T42.700x001	Hypnotic poisoning
T42.700x003	Poisoning by tranquilizers
T42.701	poisoning of sedativehypnotic drugs
T42.702	Poisoning by sleeping pills

T42.800	Poisoning by antiparkinsonian drugs and other central nervous system muscle tone inhibitors
T42.800x001	Poisoning by antiparatic drugs
T42.800x002	Poisoning by central nervous system muscle tension inhibitor
T42.800x003	Amantadine poisoning
T43.000	Poisoning by tricyclic and tetracyclic antidepressants
T43.000x002	Poisoning by tricyclic antidepressant
T43.000x003	Poisoning by tetracycline antidepressant
T43.001	Poisoning by amitriptyline
T43.002	Poisoning by doxepin
T43.100	Monoamine oxidase inhibitor antidepressant poisoning
T43.200	Antidepressant poisoning, other and unspecified
T43.200x001	Poisoning by fluoxetine
T43.201	Poisoning by antidepressants
T43.300	Poisoning by phenthiazide antipsychotics and antipsychotics
T43.300x001	Perphenazine poisoning
T43.300x003	Poisoning by phenol thiazide type antipsychotic drugs
T43.301	Fenergan poisoning
T43.302	Poisoning by chlorpromazine
T43.400	Poisoning by butylbenzene and thioanthracene
T43.400x002	Butyryl benzene poisoning
T43.400x003	Poisoning by thioanthracene antipsychotic
T43.401	Poisoning by haloperidol
T43.500	Poisoning by antipsychotics and tranquilizers, other and unspecified
T43.500x001	Tranquility poisoning
T43.500x002	Poisoning by quetiapine fumarate
T43.500x003	Lithium carbonate poisoning
T43.500x004	Poisoning by antipsychotics
T43.500x005	Poisoning by pentafluridol
T43.501	Poisoning by antipsychotics and antipsychotics
T43.502	Poisoning by Miaoltong
T43.600	Mental stimulant poisoning with abuse potential
T43.600x003	Methamphetamine poisoning
T43.600x004	Ecstasy poisoning
T43.601	Caffeine poisoning
T43.800	Poisoning caused by drugs that have an impact on the spirit, and others that cannot be classified elsewhere

T43.900	Poisoning by drugs with mental effects
T44.000	Poisoning by anticholinesterase agent
T44.001	Poisoning by pyrimethamine
T44.100	Parasympathetic drugs [cholinergic drugs] poisoning, others
T44.100x001	Parasympathetic intoxication
T44.200	Poisoned by ganglion blockers, which cannot be classified elsewhere
T44.200x001	Poisoning by ganglion blockers
T44.300	Parasympathetic depressants [anticholinergics and anti muscarinic drugs] those poisoned by antispasmodics, others, which cannot be classified elsewhere
T44.301	Atropine poisoning
T44.302	Anisodamine poisoning
T44.303	Poisoning by artan
T44.400	Mainly α Poisoned by a highly effective adrenergic receptor drug, which cannot be classified elsewhere
T44.400x001	α Poisoning by highly effective adrenergic receptor drugs
T44.400x002	Alamin poisoning
T44.500	Mainly β Poisoned by a highly effective adrenergic receptor drug, which cannot be classified elsewhere
T44.500x001	β Poisoning by highly effective adrenergic receptor drugs
T44.600	α Poisoning by adrenergic receptor antagonist, which cannot be classified elsewhere
T44.600x001	α Poisoning by adrenergic receptor antagonist
T44.700	β Poisoning by adrenergic receptor antagonist, which cannot be classified elsewhere
T44.700x001	β Poisoning by adrenergic receptor antagonist
T44.701	Poisoning by propranolol
T44.800	Central effect and adrenergic neuron blocker poisoning, which cannot be classified elsewhere
T44.800x001	Central effects and adrenergic neuron blocker poisoning
T44.900	Other and unspecified drug poisoning mainly affecting the autonomic nervous system
T44.900x001	α and β Poisoning by adrenergic receptor drugs
T44.900x002	Ephedrine poisoning
T44.900x003	Poisoning by angiotensin receptor inhibitor

T44.901	Drug poisoning mainly affecting autonomic nervous system
T45.000	Poisoning by antiallergic drugs and antiemetics
T45.000x001	Poisoning by antiallergic drugs
T45.001	Poisoning by antiemetic
T45.002	Metoclopramide poisoning
T45.003	Poisoning by Chenghuining
T45.100	Poisoning by antineoplastic drugs and immunosuppressants
T45.100x001	Antineoplastic antibiotic poisoning
T45.100x002	Poisoning by cytarabine
T45.100x003	Cyclosporine poisoning
T45.100x004	Hypermethotrexaemia
T45.101	Poisoning by methotrexate
T45.102	Poisoning by vincristine
T45.200	Vitamin poisoning, which cannot be classified elsewhere
T45.200x001	Vitamin poisoning
T45.201	Poisoning by vitamin A
T45.202	Vitamin D poisoning
T45.300	Enzyme poisoning, which cannot be classified elsewhere
T45.300x001	Enzyme poisoning
T45.400	Poisoning by iron and its compounds
T45.500	Anticoagulant poisoning
T45.500x002	Poisoning by anticoagulants
T45.501	Poisoning by new anticoagulant tablets
T45.600	Affect the poisoning of fibrinolytic drugs
T45.700	Poisoning by anticoagulant antagonists, vitamin K and other clotting drugs
T45.700x001	Poisoning by anticoagulant antagonist
T45.700x002	Poisoning by vitamin K
T45.700x003	Coagulant poisoning
T45.800	Poisoning, others are mainly systemic and hematological preparations
T45.800x001	Natural blood poisoning
T45.800x002	Poisoning of blood products
T45.800x003	Plasma substitute poisoning
T45.900	Mainly systemic and hematological agent poisoning
T46.000	Poisoning by cardiac excitatory glycosides and similar drugs
T46.001	Digoxin poisoning
T46.002	Digitalis poisoning
T46.100	Poisoning by calcium channel blockers
T46.100x001	Poisoning by verapamil

T46.200	Poisoning by antiarrhythmic drugs, others, which cannot be classified elsewhere
T46.200x001	Poisoning by antiarrhythmic drugs
T46.300	Coronary vasodilator poisoning, which cannot be classified elsewhere
T46.300x002	Poisoning by coronary vasodilator
T46.300x003	Poisoning by dipyridamole
T46.301	Nitroglycerin poisoning
T46.302	Imdor poisoning
T46.400	Poisoning by angiotensin-converting enzyme inhibitor
T46.500	Poisoned by antihypertensive drugs, others, not to be classified elsewhere
T46.500x002	Poisoning by guanethidine
T46.500x003	Poisoning by Rauwolfia
T46.500x004	Poisoning by reserpine
T46.500x005	Poisoning by antihypertensive drugs
T46.501	Poisoning by clonidine
T46.600	Poisoning by anti hyperlipidemia and anti atherosclerosis drugs
T46.600x001	Poisoning by anti atherosclerosis drugs
T46.700	Poisoning by peripheral vasodilator
T46.700x001	Poisoning by compound hypotensive tablets
T46.700x002	Nicotinic acid poisoning
T46.800	Poisoning by anti varicose drugs (including sclerosing agents)
T46.800x001	Poisoning by anti varicose drugs
T46.900	Poisoning by other agents mainly affecting cardiovascular system
T46.900x001	Aconitine poisoning
T46.901	Poisoning of preparations mainly affecting cardiovascular system
T47.000	Poisoning by histamine H2 receptor antagonist
T47.100	Poisoning by antacids and anti gastric secreting drugs, others
T47.100x001	Poisoning by antacids and anti gastric secreting drugs
T47.200	Irritating laxative poisoning
T47.200x002	Aloe poisoning
T47.300	Poisoning by saline and osmotic laxative
T47.300x001	Pervious laxative poisoning
T47.400	Poisoning by laxative, others
T47.400x001	Poisoning by intestinal relaxant
T47.500	Digestive drug poisoning
T47.600	Poisoning by antidiarrheal
T47.700	Poisoning by emetics

T47.800	Poisoning by other agents mainly affecting gastrointestinal system
T47.900	Poisoning of preparations mainly affecting gastrointestinal system
T48.000	Poisoning by oxytocin
T48.100	Skeletal muscle relaxant [neuromuscular blocker] poisoning
T48.100x001	Poisoning by skeletal muscle relaxant
T48.200	Poisoning of other and unspecified preparations mainly acting on muscles
T48.201	Poisoning by acting on muscle preparations
T48.300	Poisoning by antitussive
T48.400	Poisoning by expectorants
T48.500	Poisoning by anti cold drugs
T48.600	Poisoned by anti asthma drugs, which cannot be classified elsewhere
T48.600x002	Datura poisoning
T48.600x003	Poisoning by salbutamol
T48.601	Poisoning by aminophylline
T48.602	Poisoning by amlodipine
T48.603	Poisoning by clenbuterol
T48.700	Poisoning of other and unspecified preparations mainly acting on respiratory system
T48.701	Poisoning by respiratory system agents
T49.000	Local antifungal, anti infective and anti-inflammatory drug poisoning, which cannot be classified elsewhere
T49.000x003	Iodine intoxication
T49.000x005	Poisoning by bromogeramine
T49.001	Cresol poisoning
T49.002	Poisoning by mercurous chloride
T49.003	Lysus poisoning
T49.100	Poisoning by antipruritic drugs
T49.200	Poisoning by local astringent and local detergent
T49.200x001	Poisoning by local astringent
T49.201	Local detergent poisoning
T49.300	Poisoning by lubricant, palliative and protective drugs
T49.300x001	Lubricant poisoning
T49.300x002	Poisoning by moderator
T49.300x003	Poisoning by gastric mucosal protective agent
T49.400	Poisoning by cuticle separating agents, cuticle hyperplasia agents and other drugs and preparations for hair treatment
T49.400x001	Poisoning by cuticle separating drug
T49.400x002	Poisoning by cuticle hyperplasia drugs
T49.400x003	Poisoning of drugs and preparations for hair treatment
T49.500	Ophthalmic drugs and preparations poisoning
T49.600	Poisoning by otorhinolaryngological drugs and preparations
T49.700	Dental drug poisoning, topical
T49.700x001	Local drug poisoning in stomatology department
T49.800	Poisoning by local preparation, others
T49.800x001	Poisoning by spermicide
T49.801	Cosmetic poisoning
T49.900	Poisoning by local preparation
T50.000	Poisoning by halocorticoids and their antagonists
T50.000x001	Poisoning by mineralocorticoids and their antagonists
T50.100	Diuretic poisoning
T50.100x001	Loop diuretic poisoning
T50.200	Poisoning by carbonic acid dehydratase inhibitors, benzothiadiazines and other diuretics
T50.200x001	Poisoning by acetamide
T50.200x002	Mercury diuretic poisoning
T50.300	Poisoning by electrolyte, heat and water balance agent
T50.300x001	Poisoning by potassium chloride
T50.300x002	Oral rehydration salt poisoning
T50.400	Drug poisoning affecting uric acid metabolism
T50.400x001	Poisoning by uric acid metabolite
T50.500	Poisoning by appetite inhibitor
T50.600	Poisoned by antidote and chelating agent, which cannot be classified elsewhere
T50.600x001	Poisoning by antialcoholic drugs
T50.600x002	Poisoning by antidote
T50.600x003	Chelating agent poisoning
T50.700	Poisoning by stimulants and opioid receptor antagonists
T50.700x001	Excitator poisoning
T50.700x002	Poisoning by opioid receptor antagonist
T50.800	Diagnostic agent poisoning
T50.900	Poisoning by drugs, pharmaceuticals and biological products, other and unspecified
T50.900x001	Drug poisoning

T50.900x002	Acidifier poisoning
T50.900x003	Alkaline poisoning
T50.900x004	Immunoglobulin poisoning
T50.900x005	Poisoning by immune agents
T50.900x006	Lipid regulating drug poisoning
T50.900x007	Poisoning by parathyroid hormones
T51.000	Toxic effect of ethanol
T51.000x001	Alcoholism
T51.001	alcoholism
T51.100	Toxic effect of methanol
T51.100x001	Methanol poisoning
T51.200	Toxic effect of 2-propanol
T51.200x001	Isopropyl alcohol poisoning
T51.300	Toxic effect of fusel oil
T51.300x001	Fusel oil poisoning
T51.300x002	Poisoning by amyl alcohol
T51.300x003	Butyl alcohol poisoning
T51.300x004	Poisoning by propyl alcohol
T51.800	Toxic effects of alcohols, others
T51.800x001	Poisoning by sodium trichloropyridinol
T51.900	Toxic effect of alcohol
T51.900x001	Alcoholism
T52.000	Toxic effects of petroleum products
T52.000x001	Poisoning by petroleum products
T52.000x002	Naphtha poisoning
T52.000x003	Kerosene poisoning
T52.000x004	Gasoline poisoning
T52.000x005	Ether poisoning
T52.000x006	Poisoning by petroleum essence
T52.100	Toxic effects of benzene
T52.101	Benzene poisoning
T52.200	Toxic effects of benzene analogues
T52.200x001	Poisoning by benzene compounds
T52.200x002	Toluene poisoning
T52.200x003	Xylene poisoning
T52.300	Toxic effects of aliphatic diols
T52.300x001	Aliphatic diols poisoning
T52.400	Toxic effects of ketones
T52.400x001	Ketosis
T52.800	Toxic effects of other organic solvents
T52.800x001	Dimethyl Formamide Poisoning
T52.800x002	Poisoning by formaldehyde aqueous solution
T52.800x003	Poisoning by dichloroethane
T52.800x004	n-hexane poisoning
T52.800x005	Banana water poisoning
T52.800x006	Poisoning by dimethyl sulfate
T52.900	Toxic effects of organic solvents
T52.900x001	Poisoning by organic solvent
T53.000	Toxic effect of carbon tetrachloride
T53.000x001	Carbon tetrachloride poisoning
T53.000x002	Tetrachloromethane poisoning
T53.100	Toxic effect of chloroform
T53.100x001	Chloroform poisoning
T53.100x002	Poisoning by chloroform
T53.200	Toxic effect of trichloroethylene
T53.200x001	Poisoning by trichloroethylene
T53.200x002	Poisoning by trichloroethane
T53.300	Toxic effect of tetrachloroethylene
T53.300x001	Perchloroethylene poisoning
T53.300x002	Tetrachloroethylene poisoning
T53.400	Toxic effect of dichloromethane
T53.400x001	Dichloromethane poisoning
T53.500	Toxic effects of chlorofluorocarbons
T53.500x001	Poisoning by chlorofluorocarbons
T53.600	Toxic Effects of Other Halogen Derivatives of Alicyclic Hydrocarbons
T53.600x001	Poisoning by vinyl chloride
T53.600x002	Poisoning by trichloropropane
T53.600x003	Poisoning by chloroprene
T53.600x004	Poisoning by allyl chloride
T53.700	Toxic Effects of Other Halogen Derivatives of Aromatic Hydrocarbons
T53.700x001	Poisoning by chlorophenol
T53.900	Toxic Effects of Halogen Derivatives of Alicyclic Hydrocarbons and Aromatic Hydrocarbons
T54.000	Toxic effects of phenol and its analogues
T54.000x001	Poisoning by phenol and its analogues
T54.000x002	Phenol poisoning
T54.100	Toxic effects of corrosive organic compounds, others
T54.200	Toxic effects of corrosive acids and acid like substances
T54.200x001	Sulfuric acid chemical injury
T54.200x002	Nitrite chemical injury
T54.200x003	Hydrochloric acid chemical injury
T54.200x004	Acid chemical injury
T54.201	Sulfuric acid poisoning

T54.202	Acid poisoning
T54.203	Nitrite poisoning
T54.300	Toxic effects of corrosive alkali and alkali like substances
T54.300x002	Potassium hydroxide chemical injury
T54.300x003	Sodium hydroxide chemical injury
T54.301	Caustic alkali poisoning
T54.900	Toxic effects of corrosive substances
T54.900x001	Chemical injury caused by corrosive substances
T54.900x002	Brine poisoning
T55.x00	Toxic effects of soaps and detergents
T55.x00x001	Soap poisoning
T55.x00x002	Detergent poisoning
T55.x00x003	Detergent poisoning
T56.000	Toxic effects of lead and its compounds
T56.000x002	Poisoning by lead compounds
T56.000x003	Tetraethyl lead poisoning
T56.001	lead poisoning
T56.100	Toxic effects of mercury and its compounds
T56.100x002	Poisoning by mercury compounds
T56.101	Mercury poisoning
T56.200	Toxic effects of chromium and its compounds
T56.200x001	Chromium poisoning
T56.200x002	Poisoning by chromium compounds
T56.300	Toxic effects of cadmium and its compounds
T56.300x001	Cadmium poisoning
T56.300x002	Poisoning by cadmium compounds
T56.400	Toxic effects of copper and its compounds
T56.400x002	Poisoning by copper compounds
T56.401	Copper poisoning
T56.500	Toxic effects of zinc and its compounds
T56.500x001	Zinc poisoning
T56.500x002	Poisoning by zinc compounds
T56.600	Toxic effects of tin and its compounds
T56.600x001	Tin poisoning
T56.600x002	Poisoning by tin compounds
T56.700	Toxic effects of beryllium and its compounds
T56.700x002	Poisoning by beryllium compound

T56.800	Toxic effects of metals, others
T56.800x001	Vanadium poisoning
T56.800x002	Poisoning by vanadium compounds
T56.800x003	Uranium poisoning
T56.800x004	Poisoning by uranium compounds
T56.800x005	nickel car-bonyI poisoning
T56.800x006	Indium poisoning
T56.800x007	Indium compound poisoning
T56.801	Thallium poisoning
T56.900	Toxic effects of metals
T56.900x001	Metal poisoning
T56.900x002	Metal fume heat
T56.900x003	Metal vapor poisoning
T57.000	Toxic effects of arsenic and its compounds
T57.000x001	Arsenism
T57.000x002	Arsenic poisoning
T57.000x003	Arsine poisoning
T57.001	arsenic poisoning
T57.100	Toxic effects of phosphorus and its compounds
T57.100x001	Phosphorus poisoning
T57.100x002	Poisoning by phosphorus compounds
T57.100x003	Phosphine poisoning
T57.100x004	Zinc phosphide poisoning
T57.100x005	Aluminum phosphide poisoning
T57.200	Toxic effects of manganese and its compounds
T57.200x001	Manganese poisoning
T57.200x002	Poisoning by manganese compounds
T57.201	Manganide poisoning
T57.300	Toxic effect of hydrogen cyanide
T57.300x001	Hydrogen cyanide poisoning
T57.800	Toxic effects of inorganic substances, other specific
T57.800x002	Poisoning by barium compound
T57.800x003	Barium poisoning
T57.900	Toxic effects of inorganic substances
T58.x00	Toxic effects of carbon monoxide
T58.x00x001	carbon monoxide poisoning
T59.000	Toxic effects of nitrogen oxides
T59.000x001	Nitrogen poisoning
T59.000x002	Nitrogen oxide poisoning
T59.100	Toxic effect of sulfur dioxide
T59.100x001	Sulfur dioxide poisoning
T59.101	Smoke poisoning

T59.200	Toxic effect of formaldehyde
T59.200x001	Formaldehyde poisoning
T59.300	Toxic effects of tear gas
T59.300x001	Tear gas poisoning
T59.400	Toxic effect of chlorine
T59.401	Chlorine poisoning
T59.500	Toxic effects of fluorine gas and hydrogen fluoride
T59.500x001	Fluorine poisoning
T59.500x002	Fluoride poisoning
T59.600	Toxic effect of hydrogen sulfide
T59.601	Hydrogen sulfide poisoning
T59.700	Toxic effects of carbon dioxide
T59.700x001	Carbon dioxide poisoning
T59.800	Toxic effects of gases, smoke and vapours, other specific
T59.800x001	Biogas poisoning
T59.800x002	LPG poisoning
T59.800x004	Natural gas poisoning
T59.800x005	Methyl bromide poisoning
T59.800x006	Mustard gas poisoning
T59.800x007	Phosgene poisoning
T59.800x008	Monomethylamine Poisoning
T59.800x009	Bromopropane poisoning
T59.800x010	Ethylene oxide poisoning
T59.801	Ammonia poisoning
T59.802	Liquefied gas poisoning
T59.803	Total hydrocarbon oil vapor poisoning
T59.900	Toxic effects of gases, fumes and vapours
T59.900x001	Irritating gas poisoning
T59.900x002	Poisoning by aerosol
T59.900x003	Volatile poisoning
T59.900x004	Mixed gas poisoning
T59.900x005	Asphyxiating gas poisoning
T60.000	Toxic effects of organophosphate and carbamate insecticides
T60.000x003	Phoxim poisoning
T60.000x004	Carbamate insecticide poisoning
T60.001	Organophosphorus poisoning
T60.002	Dichlorvos poisoning
T60.100	Toxic effects of halogenated pesticides
T60.100x001	Halogenated insecticide poisoning
T60.101	Deltamethrin poisoning
T60.200	Toxic effects of pesticides, others
T60.200x001	Poisoning by cockroach killing drug
T60.300	Toxic effects of herbicides and fungicides

T60.300x001	Poisoning by herbicide
T60.300x002	Fungicide poisoning
T60.300x003	Poisoning by chloroacetic acid
T60.400	Toxic effect of rodenticide
T60.400x003	Thallium poisoning
T60.401	Poisoning by rodenticide
T60.800	Toxic effects of crop pesticides, others
T60.900	Toxic effects of pesticides
T60.900x001	Pesticide poisoning
T60.900x002	pesticide poisoning
T60.900x003	Poisoning by wood preservatives
T61.000	Fish poisoning
T61.001	Fish gall poisoning
T61.100	Poisoning by mackerel
T61.100x002	Histamine like syndrome
T61.200	Poisoning of fish and aquatic shellfish, others
T61.200x001	Fish poisoning
T61.200x003	Shellfish poisoning
T61.201	Globefish poisoning
T61.800	Toxic effects of marine products, others
T61.900	Toxic effects of marine products
T61.900x001	Poisoning of seafood
T62.000	Toxic effects of mushroom intake
T62.000x001	Mushroom poisoning
T62.000x002	Mushroom poisoning
T62.001	Poisoned by eating poisonous mushrooms
T62.002	Boletus poisoning
T62.100	Toxic effects of intake of berries
T62.100x001	Berry poisoning
T62.200	Toxic effects of ingestion of other plants (or parts of plants)
T62.200x002	Poisoning by castor bean
T62.200x003	Botanical poisoning
T62.202	Solanum fruit poisoning
T62.800	Toxic effects of other specifically harmful substances in food intake
T62.800x002	Nitrite poisoning
T62.801	Poisoning by bitter almond
T62.802	Lentil poisoning
T62.900	Toxic effects of harmful substances in food intake
T62.900x002	food poisoning
T63.000	Toxic effect of snake venom
T63.000x001	Poisoning by snake venom
T63.001	Poisonous snake bite
T63.100	Toxic effects of reptile venom, others

T63.100x001	Poisoning by lizard venom
T63.200	Toxic effect of scorpion venom
T63.200x001	Poisoning by scorpion venom
T63.300	Toxic effect of spider venom
T63.300x001	Spider venom poisoning
T63.400	Toxic effects of arthropod venom, others
T63.400x002	Poisonous insect bites
T63.400x003	Poisonous insect sting
T63.400x004	Sandfly Bite
T63.401	Bee sting
T63.402	Arthropod bites
T63.500	Toxic effects in contact with fish
T63.500x001	Poisoning after contact with fish
T63.600	Toxic effects of contact with other marine animals
T63.600x001	Poisoning after contact with jellyfish
T63.600x002	Poisoned after contacting sea anemone
T63.600x003	Poisoning after contact with aquatic shellfish
T63.600x004	Poisoning after contact with marine animals
T63.600x005	Poisoned after contacting starfish
T63.800	Toxic effects in contact with other toxic animals
T63.800x001	Amphibian venom poisoning
T63.900	Toxic effects in contact with toxic animals
T64.x00	Toxic effects of aflatoxin and other mycotoxins on food
T64.x00x001	Aflatoxin poisoning
T64.x00x002	Toxic effect of mycotoxin on food
T64.x01	Toxic effects of other mycotoxins on food
T64.x02	Toxic effect of aflatoxin on food
T65.000	Toxic effect of cyanide
T65.000x001	Cyanide poisoning
T65.100	Toxic effect of Shi Di Nian and its salts
T65.100x001	Poisoning by strychnine and its salts
T65.200	Toxic effects of tobacco and nicotine
T65.200x001	Tobacco poisoning
T65.200x002	Nicotine poisoning
T65.300	Toxic effects of nitrogen derivatives and amine derivatives of benzene and its analogues
T65.300x001	Aniline poisoning
T65.300x002	Nitrobenzene poisoning
T65.300x003	Poisoning by trinitrotoluene
T65.300x004	Poisoning by nitro compounds
T65.400	Toxic effect of carbon disulfide
T65.400x001	Carbon disulfide poisoning
T65.500	Toxic effects of nitroglycerin and other nitric acids and esters
T65.500x001	Nitroglycerol poisoning
T65.500x002	Poisoning by trinitroglycerin
T65.501	Specifically refers to the toxic effects of nitric acid and esters
T65.600	Toxic effects of paints and dyes, which cannot be classified elsewhere
T65.600x001	Varnish poisoning
T65.600x002	Paint poisoning
T65.600x003	Dye poisoning
T65.800	Toxic effects of substances, others specifically
T65.800x002	Poisoning by hair dye
T65.800x003	Poisoning by potassium permanganate
T65.800x004	Dimethylhydrazine Poisoning
T65.800x005	Poisoning by disinfectant
T65.800x006	Acrylamide poisoning
T65.800x007	Methyl iodide poisoning
T65.801	Humidifier disinfectant poisoning
T65.900	Toxic effects of substances
T65.900x001	Antifreeze poisoning
T65.901†	Toxic dementia
T66.x00	Effect of radiation
T66.x00x001	Radiation sickness
T66.x00x002	Occupational radiation sickness
T66.x01	Radiation damage
T66.x02	Radiation optic nerve damage
T67.000	Heat stroke and sunstroke
T67.000x001	Heat apoplexy
T67.000x002	Heat stroke
T67.001	Thermal fever
T67.002	Sunstroke
T67.100	Febrile syncope
T67.100x002	Thermal collapse
T67.200	Heat cramp
T67.300	Dehydration heat exhaustion
T67.300x001	Heatstroke dehydration
T67.300x002	Dehydrative heat exhaustion
T67.400	Heat exhaustion caused by salt deficiency
T67.400x001	Heat exhaustion due to salt deficiency
T67.500	Heat exhaustion
T67.500x001	Heat exhaustion

T67.600	Transient heat exhaustion
T67.700	Heatstroke edema
T67.800	Other effects of heat and light
T67.900	Effect of heat and light
T67.901	Heat stroke
T68.x00	Hypothermia
T68.x00x002	Accidental hypothermia
T69.000	Soak hands and feet
T69.000x001	Soak hands
T69.000x002	Soaking foot
T69.000x003	Trench foot
T69.000x004	Hunting reaction
T69.100	chilblains
T69.100x002	Chilblain of auricle
T69.100x003	Chilblain of foot
T69.100x004	Facial chilblain
T69.100x005	Chilblain of hand
T69.800	Other specific effects of cooling
T69.800x001	Chap
T69.800x002	Chapped hand
T69.800x003	Chapped foot
T69.900	Cooling effect
T70.000	Aerootitis media
T70.100	Aviation sinusitis
T70.200	Other and unspecified effects of high altitude
T70.200x005	Barotrauma
T70.200x006	Alpine disease
T70.200x007	High altitude heart disease
T70.201	High altitude pulmonary edema
T70.202	High altitude hypertension
T70.203	High altitude cerebral edema
T70.204	Mountain sickness
T70.205	Aviation disease
T70.206	Pilot disease (caused by change of flight air pressure)
T70.207	High altitude effect
T70.300	Diver's disease [decompression sickness]
T70.300x002	Diver paralyzed
T70.300x004	Diver paralysis
T70.400	Effect of high pressure liquid
T70.800	Other effects of air pressure and water pressure
T70.800x001	Shock wave injury syndrome
T70.900	Effect of air pressure and water pressure
T71.x00	Extrinsic asphyxia
T71.x00x001	Anoxic asphyxia
T71.x00x002	Traumatic asphyxia

T71.x00x003	Strangulation asphyxia
T71.x00x004	Mechanical asphyxia
T73.000	starvation effect
T73.000x001	Hunger strike
T73.100	Thirst effect
T73.200	Failure caused by exposure to adverse environment
T73.300	Exhaustion caused by overwork
T73.800	Other effects lacking
T73.900	Effect of lack
T74.000	Neglected or abandoned
T74.000x001	Neglected syndrome
T74.000x002	Abandonment syndrome
T74.100	Physical abuse
T74.100x001	Child abuse syndrome
T74.100x002	Child abuse syndrome
T74.100x003	Spouse abuse syndrome
T74.100x004	Somatic Abuse Syndrome
T74.200	sexual abuse
T74.800	Abuse syndrome, other
T74.800x001	Mixed maltreatment syndrome
T74.900	Abuse syndrome
T74.900x001	Adult Abuse Syndrome
T74.900x002	Child abuse syndrome
T75.000	Lightning effect
T75.000x001	Lightning strike
T75.000x002	Lightning shock
T75.100	Drowning and non fatal drowning
T75.100x001	drowning
T75.100x002	Swimmer's cramp
T75.101	Drowning pulmonary edema
T75.200	Vibration effect
T75.200x001	Air hammer syndrome
T75.200x002	Subsonic vertigo
T75.200x003	Traumatic vasospasm syndrome
T75.200x004	Local vibration disease
T75.200x005	Arm vibration disease
T75.300	Motion sickness
T75.300x002	Airsickness
T75.300x003	Seasickness
T75.300x004	Carsickness
T75.400	Current effect
T75.400x001	Electric injury
T75.800	Other specific effects of external factors
T75.800x001	Anomalous gravity effect
T75.800x002	Weightlessness effect

T78.000	Allergic shock caused by harmful food reaction
T78.000x001	Food induced anaphylactic shock
T78.100	Harmful food reactions, others that cannot be classified elsewhere
T78.100x001	Food induced allergic reaction
T78.100x011	food allergy
T78.101	Milk allergy
T78.200	Anaphylactic shock
T78.201	Hexheimer reaction
T78.300	Angioneurotic edema
T78.300x003	Vaso neurogenic reaction of auricle
T78.300x004	Giant urticaria
T78.301	Acute idiopathic edema
T78.400	allergy
T78.400x002	Anaphylactic reaction
T78.800	Harmful effects, others that cannot be classified elsewhere
T78.900	Harmful effect
T79.000	Air embolism (traumatic)
T79.100	Fat embolism (traumatic)
T79.100x002	Traumatic cerebral fat embolism
T79.101	Fat embolism syndrome
T79.200	Secondary and recurrent bleeding from trauma
T79.201	Traumatic recurrent hemorrhage
T79.202	Traumatic secondary bleeding
T79.300	Wound infection after trauma, which cannot be classified elsewhere
T79.300x001	Post traumatic wound infection
T79.400	Traumatic shock
T79.500	Traumatic anuria
T79.500x002	Renal failure after extrusion
T79.501	Crush syndrome
T79.600	Traumatic muscle ischemia
T79.600x003	Lacunar syndrome
T79.600x004	Upper limb osteofascial compartment syndrome
T79.600x006	Lower limb osteofascial compartment syndrome
T79.601	Traumatic osteofascial compartment syndrome
T79.602	Folkman ischemic contracture
T79.603	Abdominal compartment syndrome
T79.700	Traumatic subcutaneous emphysema
T79.800	Other early complications of trauma
T79.800x001	Traumatic lipoliquefaction
T79.800x002	Traumatic necrosis of lower limbs
T79.800x003	Traumatic necrosis of fingers
T79.800x004	Traumatic necrosis of scalp
T79.800x005	Traumatic coagulopathy
T79.800x006	Traumatic hypotony
T79.800x007	Traumatic pneumonia
T79.801	Traumatic meningitis
T79.900	Early complications of trauma
T81.601	Chemical peritonitis
T90.503	Traumatic epilepsy
T92.600x003	Traumatic bone defect of upper limb
T98.200x011	Open injury with foreign body
T98.200x012	Open injury with foreign body and infection
T98.200x021	Open injury with infection
T98.200x031	Delayed healing of open injury
T98.200x032	Delayed treatment of open injury
T98.200x033	Poor healing of open wound
G62.809	Traumatic peripheral neuropathy
G93.200x001	Intracranial hypertension
G93.501	Cerebral hernia
G93.808	Ventricular dilation
G95.106	Spinal cord edema
G95.200	Spinal cord compression
G96.000x006	Traumatic cerebrospinal fluid leakage
G96.001	Cerebrospinal rhinorrhea
G96.002	Cerebrospinal fluid otorrhea
H18.800x009	Corneal epithelial injury
H20.802	Traumatic iridocyclitis
H31.403	Traumatic choroidal detachment
H33.302	Traumatic retinal tear
H33.503	Traumatic retinal detachment
H35.703	Traumatic chorioretinopathy
H40.301	Traumatic glaucoma
H83.301	Acoustic trauma
H91.801	traumatic deafness
I31.800x003	Pericardial pneumatosis
I63.908	Traumatic cerebral infarction
I74.303	Traumatic femoral artery thrombosis
J38.702	Traumatic laryngeal web
J81.x00x001	Traumatic wet lung
K05.500x001	occlusal trauma
K06.200	Injury related gingival and edentulous ridge damage
K12.101	Traumatic oral mucosal ulcer

K14.000x006	Traumatic ulcer of tongue
K20.x00x003	Traumatic esophagitis
K22.207	Traumatic esophageal stricture
K22.301	Esophageal rupture
K85.802	Acute Traumatic Pancreatitis, Mild
K85.815	Acute Traumatic Pancreatitis, Severe
M12.500	Traumatic arthropathy
M25.000	Joint hematocele
M41.501	Traumatic scoliosis
M43.101	Traumatic spondylolisthesis
M48.300	Traumatic spondylosis
M48.304	Traumatic lumbar spondylopathy
M89.820	Bone exposure
R40.200	coma
R57.100	Hypovolemic shock
R57.101	Hemorrhagic shock

Supplementary Table 2. ICD-10 Code of Trauma Etiology

ICD-10 encoding	External factors of injury and poisoning
V01.x00	Pedestrian injuries in collisions with bicycles
V02.x00	Pedestrian injury in collision with two or three wheeled motorcycles
V03.x00	Injury of pedestrians in collision with cars, light trucks or caravans
V04.x00	Pedestrian injuries in collisions with heavy transport vehicles or buses
V05.x00	Injury of pedestrians in collision with trains or railway vehicles
V06.x00	Pedestrian injuries in collisions with other non motor vehicles
V09.000	Pedestrian injuries in non traffic accidents involving other and unspecified motor vehicles
V09.100	Pedestrian injuries in non traffic accidents
V09.200	Pedestrian injuries in traffic accidents involving other and unspecified motor vehicles
V09.300	Pedestrian injuries in traffic accidents
V09.900	Pedestrian injuries in transport accidents
V10.x00	Injuries of cyclists in collisions between bicycles and pedestrians or livestock
V11.x00	Injuries of cyclists in collisions between bicycles and other bicycles
V12.x00	Injuries of cyclists in collisions between bicycles and two or three wheeled motor vehicles
V13.x00	Injuries of cyclists in collisions between bicycles and cars, light trucks or caravans
V14.x00	Injuries of cyclists in collisions between bicycles and heavy transport vehicles or buses
V15.x00	Injuries of cyclists in the collision between bicycles and trains or railway vehicles
V16.x00	Injuries of cyclists in collisions between bicycles and other non motor vehicles
V17.x00	Injuries of cyclists in the collision between bicycles and fixed or stationary objects
V18.x00	Injury of cyclists in non collision transport accidents
V19.000	Injuries to cyclists caused by bicycle collision with other motor vehicles in non traffic accidents
V19.100	Injuries to cyclists caused by bicycle collision with other motor vehicles in non traffic accidents
V19.200	Injury to cyclists caused by collision between bicycle and other motor vehicles in non traffic accidents

V19.300	Injuries of cyclists in non traffic accidents
V19.400	Injuries to cyclists caused by bicycle collision with other and unspecified motor vehicles in traffic accidents
V19.500	Injuries to cyclists caused by collisions between bicycles and other motor vehicles in traffic accidents
V19.600	In the traffic accident, the collision between the bicycle and other or unspecified motor vehicles causes injuries to the driver and passengers who ride the bicycle indistinctly
V19.800	Injuries of cyclists in other specific transport accidents
V19.900	Injuries of cyclists in traffic accidents
V20.x00	Injuries of motorcycle riders in collisions between motorcycles and pedestrians or livestock
V21.x00	Injuries of Motorcyclists in Collision between Motorcycle and Bicycle
V22.x00	Injuries of motorcycle riders in the collision between motorcycles and two or three wheeled vehicles
V23.x00	Injuries of motorcycle riders in the collision between motorcycles and cars, light trucks or vans
V24.x00	Injury of motorcycle rider in collision between motorcycle and heavy transport vehicle or bus
V25.x00	Injury of motorcycle rider in collision between motorcycle and train or railway vehicle
V26.x00	Injury of motorcycle rider in collision between motorcycle and other non motor vehicles
V27.x00	Injury of motorcycle rider in collision between motorcycle and fixed or stationary objects

V28.x00	Injuries of Motorcyclists in Non collision Transportation Accidents
V29.000	Injuries to motorcycle riders caused by collision between motorcycles and other motor vehicles in non traffic accidents
V29.100	Injuries to motorcycle riders caused by collision between motorcycles and other motor vehicles in non traffic accidents
V29.200	Injuries to motorcycle riders caused by collision between motorcycles and other motor vehicles in non traffic accidents
V29.300	Injuries of Motorcyclists in Non traffic Accidents
V29.400	Injuries to motorcycle riders caused by collision of motorcycles with other and unspecified motor vehicles in traffic accidents
V29.500	Injuries to motorcycle riders caused by collision of motorcycles with other and unspecified motor vehicles in traffic accidents
V29.600	Injuries caused by collisions between motorcycles and other motor vehicles in traffic accidents and not specifically those of motorcycle riders
V29.800	Injuries of motorcycle riders in other specific transport accidents
V29.900	Injuries of Motorcyclists in Traffic Accidents
V30.x00	Injuries of three wheeled vehicle occupants in collisions between three wheeled vehicles and pedestrians or livestock
V31.x00	Injury of tricycle occupants in the collision between tricycle and bicycle
V32.x00	Injuries of three wheeled vehicle occupants in the collision between three wheeled vehicles and two or three wheeled vehicles

V33.x00	Injuries of three wheeled vehicle occupants in the collision between three wheeled vehicles and cars, light trucks or caravans
V34.x00	Injury of tricycle occupants in the collision between tricycle and heavy transport vehicle or bus
V35.x00	Injury of tricycle occupants in the collision between tricycle and train or railway vehicle
V36.x00	Injury of three wheeled motor vehicle occupants in the collision between three wheeled motor vehicles and other non motor vehicles
V37.x00	Injury of tricycle occupants in the collision between tricycle and fixed or stationary objects
V38.x00	Injury of three wheeled vehicle occupants in non collision transport accidents
V39.000	Injuries caused by the collision of three wheeled motor vehicles with other motor vehicles in non traffic accidents
V39.100	Injuries caused by the collision of three wheeled motor vehicles with other motor vehicles in non traffic accidents
V39.200	Injury of tricycle occupants caused by collision between tricycle and other motor vehicles in non traffic accidents
V39.300	Injuries of three wheeled vehicle occupants in non traffic accidents
V39.400	Injuries to the driver of three wheeled motor vehicles caused by the collision between three wheeled motor vehicles and other unspecified motor vehicles in traffic accidents
V39.500	Injuries to three wheeled motor vehicle passengers caused by collisions between three wheeled motor vehicles and other unspecified motor vehicles in traffic accidents
V39.600	Injuries of three wheeled motor vehicle occupants caused by collisions between three wheeled motor vehicles and other unspecified motor vehicles in traffic accidents
V39.800	Injuries of tricycle occupants in other special transport accidents
V39.900	Injuries of three wheeled vehicle occupants in traffic accidents
V40.x00	Injuries of car occupants in collisions between cars and pedestrians or livestock
V41.x00	Injury of car occupants in collision between car and bicycle
V42.x00	Injury of car occupants in the collision between a car and a two wheel or three wheel motor vehicle
V43.x00	Injury of car occupants in the collision between car and car, light truck or van
V44.x00	Injury of car occupants in the collision between car and heavy transport vehicle or bus
V45.x00	Injury of car occupants in the collision between car and train or railway vehicle
V46.x00	Injuries of car occupants in collisions between cars and other non motor vehicles
V47.x00	Injury of car occupants in the collision between car and fixed or stationary objects
V48.x00	Injury of car occupants in non collision transport accidents
V49.000	The collision between small and medium-sized vehicles and other motor vehicles in non traffic accidents causes damage to the driver
V49.100	The collision between small and medium-sized cars and other motor vehicles in non traffic accidents causes injuries to car passengers
V49.200	Injury of car occupants caused by collision between small cars and other motor vehicles in non traffic accidents

V49.300	Injuries of car occupants in non traffic accidents
V49.400	The collision between small and medium-sized cars and other motor vehicles in traffic accidents causes injuries to car drivers
V49.500	The collision between small cars and other motor vehicles in traffic accidents causes injuries to car passengers
V49.600	Injuries to car occupants caused by collisions between small and medium-sized cars and other unspecified motor vehicles in traffic accidents
V49.800	Injuries of car occupants in other special transport accidents
V49.900	Injuries of car occupants in traffic accidents
V50.x00	Injury of light truck or caravan occupants in the collision between light truck or caravan and pedestrians or livestock
V51.x00	Injury of light truck or caravan occupants in the collision between light truck or caravan and bicycle
V52.x00	Injury of light truck or caravan occupants in the collision between light truck or caravan and two or three wheeled motor vehicles
V53.x00	Injury of light truck or caravan occupants in the collision between light truck or caravan and car, light truck or caravan
V54.x00	Injury of light truck or caravan occupants in the collision between light truck or caravan and heavy transport vehicle or bus
V55.x00	Injury of light truck or caravan passengers in the collision between light truck or caravan and train or railway vehicle
V56.x00	Injury of light truck or caravan occupants in the collision between light truck or caravan and other non motor vehicles
V57.x00	Injury of light truck or caravan occupants in the collision between light truck or caravan and fixed or stationary objects
V58.x00	Injury of light truck or caravan occupants in non collision transport accidents
V59.000	Damage to the driver of light truck or caravan caused by collision between light truck or caravan and other motor vehicles in non traffic accidents
V59.100	Injury to passengers of light trucks or caravans caused by collision between light trucks or caravans and other motor vehicles in non traffic accidents
V59.200	Injury to the occupants of light trucks or caravans caused by collisions between light trucks or caravans and other motor vehicles in non traffic accidents
V59.300	Injury of light truck or caravan occupants in non traffic accidents
V59.400	Damage to the driver of light truck or caravan caused by collision between light truck or caravan and other and unspecified motor vehicles in traffic accidents
V59.500	Injuries to passengers of light trucks or caravans caused by collision of light trucks or caravans with other and unspecified motor vehicles in traffic accidents
V59.600	Injuries to the occupants of light trucks or caravans caused by collisions between light trucks or caravans and other unspecified motor vehicles in traffic accidents
V59.800	Injuries of light truck or caravan passengers in other special transport accidents

V59.900	Injury of light truck or caravan occupants in traffic accidents
V60.x00	Injury of heavy transport vehicle occupants in the collision between heavy transport vehicle and pedestrians or livestock
V61.x00	Injury of heavy transport vehicle occupants in the collision between heavy transport vehicle and bicycle
V62.x00	Injury of heavy transport vehicle occupants in the collision between heavy transport vehicle and two or three wheeled motor vehicles
V63.x00	Injury of heavy transport vehicle occupants in the collision between heavy transport vehicle and cars, light trucks or caravans
V64.x00	Injury of heavy transport vehicle occupants in the collision between heavy transport vehicle and heavy transport vehicle or bus
V65.x00	Injury of heavy transport vehicle occupants in the collision between heavy transport vehicle and train or railway vehicle
V66.x00	Injury of heavy transport vehicle occupants in the collision between heavy transport vehicle and other non motor vehicles
V67.x00	Injury of heavy transport vehicle occupants in the collision between heavy transport vehicle and fixed or stationary objects
V68.x00	Injury of heavy transport vehicle occupants in non collision transport accidents
V69.000	In non traffic accidents, heavy transport vehicle driver is injured due to collision with other motor vehicles
V69.100	In non traffic accidents, heavy transport vehicle passengers are injured due to collision with other motor vehicles
V69.200	Injury of heavy transport vehicle occupants caused by collision between heavy transport vehicle and other motor vehicles in non traffic accidents
V69.300	Injuries of heavy transport vehicle occupants in non traffic accidents
V69.400	The heavy transport vehicle driver is injured due to the collision between the heavy transport vehicle and other motor vehicles in traffic accidents
V69.500	In traffic accidents, heavy transport vehicles collide with other motor vehicles, causing injuries to heavy transport vehicle passengers
V69.600	Injuries of heavy transport vehicle occupants caused by collision between heavy transport vehicle and other vehicles and motor vehicles in traffic accidents
V69.800	Injuries of heavy transport vehicle occupants in other special transport accidents
V69.900	Injuries of heavy transport vehicle occupants in traffic accidents
V70.x00	Injury of bus passengers in the collision between bus and pedestrians or livestock
V71.x00	Injury of bus passengers in the collision between bus and bicycle
V72.x00	Injury of bus occupants in the collision between a bus and a two wheel or three wheel motor vehicle
V73.x00	Injuries of bus passengers in the collision between buses and cars, light trucks or vans
V74.x00	Injury of bus passengers in the collision between bus and heavy transport vehicle or bus
V75.x00	Injury of bus passengers in the collision between bus and train or railway vehicle
V76.x00	Injury of bus occupants in the collision between bus and other non motor vehicles

V77.x00	Injury of bus passengers in the collision between bus and fixed or stationary objects
V78.x00	Injury of Bus Crew in Non collision Transportation Accidents
V79.000	Injury to bus drivers caused by collision between buses and other motor vehicles in non traffic accidents
V79.100	Injury to bus passengers caused by collision between bus and other motor vehicles in non traffic accidents
V79.200	Injury of bus passengers caused by collision between bus and other motor vehicles in non traffic accidents
V79.300	Injury of Bus Crew in Non traffic Accidents
V79.400	The bus driver is injured when the bus collides with other motor vehicles in a traffic accident
V79.500	Injuries to bus passengers caused by bus collision with other motor vehicles in traffic accidents
V79.600	Injuries to bus passengers caused by bus collision with other motor vehicles in traffic accidents
V79.800	Injuries of bus passengers in other special transport accidents
V79.900	Injury of Bus Crew in Traffic Accidents
V80.000	In a non collision accident, a rider or a passenger falls or throws from a livestock or animal drawn vehicle
V80.100	Injuries of riders or passengers in collision with pedestrians or livestock
V80.200	Injury of rider or passenger in collision with bicycle
V80.300	Injury of rider or passenger in collision with two or three wheeled motor vehicles
V80.400	Injuries of riders or passengers in collision with cars, light trucks, caravans, heavy transport vehicles or buses
V80.500	Injury of rider or passenger in collision with other specially designated motor vehicles
V80.600	Injuries of riders or passengers in collision with trains or railway vehicles
V80.700	Injury of rider or passenger in collision with other non motor vehicles
V80.800	Injury of rider or passenger in collision with fixed or stationary objects
V80.900	Injuries of riders or passengers in other and unspecified transport accidents
V81.000	Injuries to train or railway vehicle passengers due to collision with motor vehicles in non traffic accidents
V81.100	Injuries to train or railway vehicle passengers due to collision with motor vehicles in traffic accidents
V81.200	Injury to train or railway vehicle passengers due to collision with or being struck by railway vehicles
V81.300	Injury to train or railway vehicle passengers due to collision with other objects
V81.400	Personal injury when getting on and off the train or railway vehicle
V81.500	Injuries caused by train or railway vehicle passengers falling on the train or railway vehicle
V81.600	Injury caused by the fall of train or railway vehicle occupants from the train or railway vehicle
V81.700	Injury to train or railway vehicle passengers caused by derailment without prior collision
V81.800	Injuries to passengers of trains or railway vehicles in railway accidents, others specifically

V81.900	Injuries to passengers of trains or railway vehicles in railway accidents
V82.000	Injuries of tram passengers (urban tram) caused by collision with motor vehicles in non traffic accidents
V82.100	Tram passenger injuries (urban tram) caused by collision with motor vehicles in traffic accidents
V82.200	Injuries to the passengers of trams (urban trams) caused by collision with or by vehicles
V82.300	Injuries to passengers of trams (urban trams) due to collision with other objects
V82.400	Personal injury when getting on and off (urban tram)
V82.500	Injuries caused by falls of urban tram passengers
V82.600	Injuries caused by urban tram passengers falling from urban trams
V82.700	No injury to urban tram passengers caused by derailment due to prior collision
V82.800	Tram passenger injuries in transportation accidents (urban tram), other special
V82.900	Injuries of tram passengers in traffic accidents (urban tram)
V83.000	Injuries of drivers on special industrial vehicles in traffic accidents
V83.100	Injuries of passengers on special industrial vehicles in traffic accidents
V83.200	Injuries of external personnel of special industrial vehicles in traffic accidents
V83.300	Injuries of passengers on special industrial vehicles in traffic accidents
V83.400	Personal injury when getting on and off special industrial vehicles

V83.500	Injuries of drivers on special industrial vehicles in non traffic accidents
V83.600	Injury of passengers on special industrial vehicles in non traffic accidents
V83.700	Injury of external personnel of special industrial vehicles in non traffic accidents
V83.900	Injury of passengers on special industrial vehicles in non traffic accidents
V84.000	Injuries of drivers on special agricultural vehicles in traffic accidents
V84.100	Injuries of passengers on special agricultural vehicles in traffic accidents
V84.200	Injuries of external personnel of special agricultural vehicles in traffic accidents
V84.300	Injuries of occupants of special agricultural vehicles in traffic accidents
V84.400	Personnel injury when getting on and off special agricultural vehicles
V84.500	Injuries of drivers on special agricultural vehicles in non traffic accidents
V84.600	Injury of passengers on special agricultural vehicles in non traffic accidents
V84.700	Injury of external personnel of special agricultural vehicles in non traffic accidents
V84.900	Injuries of passengers on special agricultural vehicles in non traffic accidents
V85.000	Injuries of drivers on special construction vehicles in traffic accidents
V85.100	Injuries of passengers on special construction vehicles in traffic accidents

V85.200	Injuries of external personnel of special construction vehicles in traffic accidents
V85.300	Injury of passengers on special construction vehicles in traffic accidents
V85.400	Personal injury when getting on and off special construction vehicles
V85.500	Injuries of drivers on special construction vehicles in non traffic accidents
V85.600	Injury of passengers on special construction vehicles in non traffic accidents
V85.700	Injury of external personnel of special construction vehicles in non traffic accidents
V85.900	Injury of passengers on special construction vehicles in non traffic accidents
V86.000	Injuries to drivers in the whole area or on other off-road vehicles in traffic accidents
V86.100	Injuries to passengers in the whole area or on other off-road vehicles in traffic accidents
V86.200	Injuries to persons outside the whole area or other off-road vehicles in traffic accidents
V86.300	Injuries to passengers in the whole area or on other off-road vehicles in traffic accidents
V86.400	Personal injury when going up and down the whole area or other off-road vehicles
V86.500	Injuries to drivers in the whole area or other off-road vehicles in non traffic accidents
V86.600	Injuries to passengers on all terrain or other off-road vehicles in non traffic accidents
V86.700	Injuries to persons outside the whole area or other off-road vehicles in non traffic accidents
V86.900	Injuries to occupants of all terrain or other off-road vehicles in non traffic accidents
V87.000	Personal injury caused by collision between cars and two or three wheeled vehicles (traffic)
V87.100	Personal injury caused by collision between motor vehicles and two or three wheeled motor vehicles (traffic related), others
V87.200	Personal injury caused by collision between cars and light trucks or caravans (traffic)
V87.300	Personal injury caused by collision between cars and buses (traffic)
V87.400	Personal injury caused by collision between car and heavy transport vehicle (traffic)
V87.500	Personal injury caused by collision between heavy transport vehicle and bus (traffic)
V87.600	Personal injury caused by collision between train or railway vehicle and car (traffic)
V87.700	Personal injury caused by collision between motor vehicles (traffic), other specific
V87.800	Personal injury (traffic) in non collision transport accidents involving motor vehicles
V87.900	Personal injury (traffic) in non motor vehicle (collision) (non collision) transport accidents
V88.000	Personal injury caused by collision between cars and two or three wheeled motor vehicles, non traffic
V88.100	Personal injury caused by collision between motor vehicle and two or three

	wheeled motor vehicle, non traffic, other
V88.200	Personal injury caused by collision between cars and light trucks or caravans, non traffic
V88.300	Personal injury caused by collision between cars and buses, non traffic
V88.400	Personal injury caused by collision between car and heavy transport vehicle, non traffic
V88.500	Personal injury caused by collision between heavy transport vehicle and bus, non traffic
V88.600	Personal injury caused by collision between train or railway vehicle and car, non traffic
V88.700	Personal injury caused by collision between motor vehicles, non traffic, other specific
V88.800	Personal injury in non collision transport accidents involving motor vehicles, non traffic, other special
V88.900	Personal injury in non motor vehicle (collision) (non collision) transport accidents, non traffic, other specific
V89.000	Personal injury in motor vehicle accidents, non traffic
V89.100	Personal injury in non motor vehicle accidents, non traffic
V89.200	Personal injury in motor vehicle accidents, traffic
V89.300	Personal injury in non motor vehicle accidents, traffic
V89.900	Personal injuries in vehicle accidents
V90.x00	Drowning and sinking caused by ship accidents
V91.x00	Other damages caused by ship accidents

V92.x00	Drowning and sinking of non vessel accidents related to water transportation
V93.x00	Shipboard accidents other than ship accidents, without causing drowning and sinking
V94.x00	Water transport accidents, others
V95.000	Helicopter accident injured passengers
V95.100	Passengers injured by accidents of super light, light or power gliders
V95.200	Private aircraft with fixed wing accident injured passengers, others
V95.300	Occupants injured in accidents of commercial aircraft with fixed wings
V95.400	Spacecraft accident injured crew
V95.800	Crew injured in aircraft accident, others
V95.900	Crew injured by aircraft accident
V96.000	Crew injured by balloon accident
V96.100	Suspended glider accident injured passengers
V96.200	Unpowered glider accident injured passengers
V96.800	Unpowered aircraft accident injured passengers, others
V96.900	Crew injured by unpowered aircraft accident
V97.000	Injuries of aircraft crew in other specific air transport accidents
V97.100	Personal injury when getting on and off the aircraft
V97.200	Injuries of skydivers in air transport accidents
V97.300	Damage of ground personnel in air transport accidents

V97.800	Air transport accident, other, not classified elsewhere
V98.x00	Transportation accident, other specific
V99.x00	Transportation accident
W00.x00	Fall on the same plane involving ice and snow
W01.x00	Slips, trips and falls on the same plane
W02.x00	Falls involving skating, skiing, roller skating or skateboarding
W03.x00	Other falls on the same plane caused by collision or pushing by others
W04.x00	Fall when being carried or supported by others
W05.x00	Involving a fall from a wheelchair
W06.x00	Involving a fall on the bed
W07.x00	Involving a fall from a chair
W08.x00	Falling on other furniture
W09.x00	Falling on playground facilities
W10.x00	Falls and falls on stairs or steps
W11.x00	Falls and falls on ladders
W12.x00	Falls and falls on scaffold
W13.x00	Falling from or out of a house or building structure
W14.x00	Fall from a tree
W15.x00	Fall off a cliff
W16.x00	Damage caused by diving or diving, except drowning and sinking
W17.x00	Other drops from one plane to another
W18.x00	Other falls on the same plane
W19.x00	fall

W20.x00	Hit by throwing, throwing or falling objects
W21.x00	Hit or be hit by sports facilities
W22.x00	Hit or be hit by other objects
W23.x00	Get caught, squeezed, jammed or clamped by objects
W24.x00	Contact with lifting and conveying devices, which cannot be classified elsewhere
W25.x00	Contact with sharp glass
W26.x00	Touch a knife, sword or dagger
W27.x00	Contacting unpowered hand tools
W28.x00	Contact powered mower
W29.x00	Contact with other power hand tools and household machinery
W30.x00	Contact agricultural machinery
W31.x00	Contact with other machinery
W32.x00	Pistol firing
W33.x00	Rifle, shotgun and firearm firing
W34.x00	Firearms fired, others
W35.x00	Boiler explosion and rupture
W36.x00	Explosion and rupture of high-pressure gas tank
W37.x00	Explosion and rupture of compressed tires, pipes and hoses
W38.x00	Explosion and rupture of compression device, other specific
W39.x00	Pyrotechnic emission
W40.x00	Material explosion, others
W41.x00	Exposed to high pressure jet
W42.x00	Exposure to noise

W43.x00	Exposure to vibration
W44.x00	Foreign matters enter or penetrate into eyes or natural cavities
W45.x00	Foreign objects or objects enter through the skin
W46.x00	Contact hypodermic syringe needle
W49.x00	Exposure to other nonliving mechanical forces
W50.x00	Being beaten, kicked, twisted, bitten or scratched by others
W51.x00	Bumping into others or being accidentally bumped by others
W52.x00	Being squeezed, pushed or trampled by the crowd
W53.x00	Bitten by rats
W54.x00	Dog bites or scratches
W55.x00	Bitten or scratched by other mammals
W56.x00	Injuries from contact with marine animals
W57.x00	Bite or sting by non-toxic insects and other non-toxic arthropods
W58.x00	Bitten or scratched by crocodiles or alligators
W59.x00	Bitten or crushed by other reptiles
W60.x00	Damage to plant thorns and thorns and sharp leaves
W64.x00	Exposed to other living mechanical forces
W65.x00	Drowning and sinking in the bathtub
W66.x00	Drowning and sinking after falling into the bathtub
W67.x00	Drowning and sinking in the swimming pool
W68.x00	Drowning and sinking after falling into the swimming pool

W69.x00	Drowning and sinking in natural waters
W70.x00	Drowning and sinking after falling into natural waters
W73.x00	Drowning and sinking, other specific
W74.x00	Drowning and submergence
W75.x00	Accidental suffocation and strangulation in bed
W76.x00	Accidental suspension and strangulation, others
W77.x00	Danger to breathing caused by landslides, falling clods and other substances
W78.x00	Inhalation of gastric contents
W79.x00	Respiratory tract obstruction caused by inhalation or ingestion of food
W80.x00	Respiratory tract obstruction caused by inhalation and swallowing of other objects
W81.x00	Enclosed or trapped in a hypoxic environment
W83.x00	Threats to breathing, other specific
W84.x00	Threat to breathing
W85.x00	Exposed to transmission lines
W86.x00	Exposure to other specified currents
W87.x00	Exposed to current
W88.x00	Exposure to ionizing radiation
W89.x00	Exposure to artificial visible light and ultraviolet light
W90.x00	Exposure to other non ionizing radiation
W91.x00	Exposure to radiation
W92.x00	Exposure to human induced overheating

W93.x00	Exposure to human induced supercooling
W94.x00	Exposure to high, low and changing atmospheric pressure
W99.x00	Exposure to other human environmental factors
X00.x00	Exposed to uncontrolled flame in the house or building structure
X01.x00	Exposed to uncontrolled flame outside the house or building structure
X02.x00	Exposed to controlled flame in the house or building structure
X03.x00	Exposed to controlled flame outside the house or building structure
X04.x00	Exposure to fire from highly flammable materials
X05.x00	Exposed to fire or burning of pajamas
X06.x00	Exposed to fire or burning of other clothing and decorations
X08.x00	Exposure to other specified smoke, fire and flame
X09.x00	Exposure to smoke, fire and flame
X10.x00	Contact with hot drinks, food, animal and vegetable oils
X11.x00	Contact with hot tap water
X12.x00	Contact with other hot liquids
X13.x00	Contact with steam and hot steam
X14.x00	Contact with hot air and gas
X15.x00	Household appliances exposed to heat
X16.x00	Heating appliances, radiators and pipes exposed to heat
X17.x00	Engines, machinery and tools exposed to heat
X18.x00	Contact with other hot metals
X19.x00	Contact with other hot and hot substances
X20.x00	Contact with poisonous snakes and lizards
X21.x00	Contact with poisonous spiders
X22.x00	Contact scorpion
X23.x00	Contact with bumblebees, wasps and bees
X24.x00	Contact centipedes and poisonous millipedes (tropical)
X25.x00	Contact with other poisonous arthropods
X26.x00	Contact with toxic marine animals and plants
X27.x00	Contact with other designated toxic animals
X28.x00	Contact with other specific poisonous plants
X29.x00	Contact with poisonous animals or plants
X30.x00	Exposure to excessive natural heat
X31.x00	Exposure to excessive natural cold
X32.x00	Exposure to sunlight
X33.x00	The victim of lightning
X34.x00	Earthquake victims
X35.x00	Volcanic eruption victims
X36.x00	Victims of avalanches, landslides and other ground movements
X37.x00	Catastrophic storm victims
X38.x00	Flood victims
X39.x00	Exposed to other natural forces

X40.x00	Accidental poisoning and exposure to non opioid analgesics, antipyretics and anti rheumatic drugs
X41.x00	Accidental poisoning of and exposure to antiepileptic drugs, sedative hypnotics, antiparkinsonian drugs and psychoactive drugs, which cannot be classified elsewhere
X42.x00	Narcotics and hallucinogens [Hallucinogens] Those who are accidentally poisoned and exposed to such drugs and cannot be classified elsewhere
X43.x00	Accidental poisoning and exposure to other drugs acting on the autonomic nervous system
X44.x00	Accidental poisoning of drugs, pharmaceuticals and biological products and exposure to such substances, others
X45.x00	Accidental poisoning and exposure to alcohol
X46.x00	Accidental poisoning and exposure to organic solvents, halocarbons and their vapors
X47.x00	Accidental poisoning of gas and steam and exposure to such substances, others
X48.x00	Accidental poisoning and exposure to pesticides
X49.x00	Accidental poisoning of chemicals and harmful substances and exposure to such substances, others
X50.x00	Excessive exertion and strenuous or repetitive exercise
X51.x00	Travel and Sports
X52.x00	Long term stay in weightlessness environment
X53.x00	Lack of food
X54.x00	Water scarcity
X57.x00	poor

X58.x00	Exposure to other specific factors
X59.000	Fracture caused by exposure to factors
X59.900	Other damages caused by exposure to factors
X60.x00	Intentional self poisoning of non opioid analgesics, antipyretics and anti rheumatic drugs and exposure to such drugs
X61.x00	Intentional self poisoning of antiepileptic drugs, sedative hypnotics, antiparkinsonian drugs and psychoactive drugs, and those exposed to such drugs, which cannot be classified elsewhere
X62.x00	Narcotics and hallucinogens [Hallucinogens] Those who intentionally poison themselves and are exposed to such drugs and cannot be classified elsewhere
X63.x00	Intentional self poisoning of other drugs acting on the autonomic nervous system and exposure to such drugs
X64.x00	Intentional self poisoning of drugs, pharmaceuticals and biological products and exposure to such drugs
X65.x00	Intentional self poisoning of alcohol and exposure to alcohol
X66.x00	Intentional self poisoning of organic solvents, halohydrocarbons and their vapors and their exposure to such substances
X67.x00	Intentional self poisoning of gases and vapours and exposure to such substances
X68.x00	Intentional self poisoning of pesticides and exposure to pesticides
X69.x00	Intentional self poisoning of chemicals and harmful substances and exposure to such substances
X70.x00	Intentional self harm by means of suspension, strangulation and suffocation

X71.x00	Intentional self harm by drowning and sinking
X72.x00	Intentional self harm by pistol firing
X73.x00	Intentional self harm by firing rifles, shotguns and large firearms
X74.x00	Intentionally harming oneself by firing other firearms
X75.x00	Intentional self harm by means of explosives
X76.x00	Intentional self harm by means of smoke, fire and flame
X77.x00	Intentional self harm by means of steam, hot gas and hot objects
X78.x00	Intentionally harm oneself with sharp objects
X79.x00	Intentional self harm by blunt force
X80.x00	Intentionally harm oneself by jumping from a high place
X81.x00	Intentionally harm oneself by jumping down or lying down in front of moving objects
X82.x00	Intentional self harm by means of motor vehicle collision
X83.x00	Intentionally harm oneself in other specific ways
X84.x00	Intentional self harm
X85.x00	Harm with drugs, medicaments and biological products
X86.x00	Harm with corrosive substances
X87.x00	Harm with insecticide
X88.x00	Harm with gas and steam
X89.x00	Harm with other specified chemicals and harmful substances
X90.x00	Harm with chemicals or harmful substances

X91.x00	Harm with suspension, strangulation and suffocation
X92.x00	Harm by drowning and sinking
X93.x00	Shoot with a pistol to inflict damage
X94.x00	Shoot with rifles, shotguns and large firearms
X95.x00	Shoot with other firearms for harm
X96.x00	Harm with explosives
X97.x00	Harm with smoke, fire and flame
X98.x00	Harm with steam, hot gas and hot objects
X99.x00	Harm with sharp objects
Y00.x00	Harm with blunt instrument
Y01.x00	Damage by pushing down from a high place
Y02.x00	Use to push or place the victim in front of a moving object for harm
Y03.x00	Harm by motor vehicle collision
Y04.x00	Aggravate with violence
Y05.x00	Sexual victimization of violence
Y06.000	Neglect and abandonment by spouse or partner
Y06.100	Neglected and abandoned by parents
Y06.200	Neglected and abandoned by acquaintances or friends
Y06.800	Neglected and abandoned by other designated personnel
Y06.900	Neglected care and abandonment
Y07.000	Abuse by spouse or partner
Y07.100	Abused by parents
Y07.200	Abused by acquaintances or friends

Y07.300	Abused by official institutions
Y07.800	Abused by other designated persons
Y07.900	Abuse
Y08.x00	Use other specific means to inflict harm
Y09.x00	Harm by means
Y10.x00	Poisoning and exposure to non opioid analgesics, antipyretics and anti rheumatic drugs with uncertain intentions
Y11.x00	Poisoning by and exposure to antiepileptic drugs, sedative hypnotics, antiparkinsonian drugs and psychoactive drugs, which cannot be classified elsewhere with uncertain intentions
Y12.x00	Poisoning of narcotics and hallucinogens [hallucinogens] and exposure to such drugs cannot be classified elsewhere, and the intention is uncertain
Y13.x00	Poisoning and exposure to other drugs acting on the autonomic nervous system with uncertain intentions
Y14.x00	Poisoning of drugs, pharmaceuticals and biological products and exposure to such drugs with uncertain intentions
Y15.x00	Alcoholism and exposure to alcohol with uncertain intention
Y16.x00	Poisoning and exposure to organic solvents, halohydrocarbons and their vapours with uncertain intentions
Y17.x00	Poisoning by gas and steam and exposure to such substances with uncertain intentions, others
Y18.x00	Poisoning and exposure to pesticides with uncertain intentions
Y19.x00	Poisoning of chemicals and harmful substances and exposure to such substances with uncertain intentions

Y20.x00	Suspension, strangulation and suffocation with uncertain intention
Y21.x00	Drowning and sinking with uncertain intention
Y22.x00	Pistol fired with uncertain intention
Y23.x00	Rifles, shotguns and large firearms are fired with uncertain intentions
Y24.x00	Firearms fired with uncertain intention, others
Y25.x00	Contact with explosives with uncertain intention
Y26.x00	Exposed to smoke, fire and flame with uncertain intention
Y27.x00	Contact with steam, hot gas and hot objects with uncertain intention
Y28.x00	Contact with sharp objects with uncertain intention
Y29.x00	Contact with blunt object, with uncertain intention
Y30.x00	Falling, jumping or being pushed down from a high place with uncertain intention
Y31.x00	Falling, lying down or running before moving objects and entering moving objects with uncertain intentions
Y32.x00	Collision of motor vehicles with uncertain intention
Y33.x00	Events, other specific intentions are uncertain
Y34.x00	Event with uncertain intention
Y35.000	Legal disposal involving the firing of firearms
Y35.100	Legal disposal involving explosives
Y35.200	Legal disposal of gas involved
Y35.300	Legal disposal involving blunt instruments

Y35.400	Legal disposal of sharps involved
Y35.500	To impose the death penalty according to law
Y35.600	Legal disposal involving other specified means
Y35.700	Dispose according to law
Y36.000	Operations involving underwater weapon explosions
Y36.100	Operations involving destruction of aircraft
Y36.200	Operations involving other explosions and shrapnel
Y36.300	Operations involving artillery, fire and hot materials
Y36.400	Firearms launch and other forms of operations involving conventional warfare
Y36.500	Operations involving nuclear weapons
Y36.600	Operations involving biological weapons
Y36.700	Chemical weapons or other forms of operations involving unconventional warfare
Y36.800	Operations occurring after the cessation of hostilities
Y36.900	Operational Action
Y40.000	Harmful effects of penicillin
Y40.100	Cephalosporins and others β Harmful effects of lactam antibiotics
Y40.200	Harmful effects of chloramphenicols
Y40.300	Harmful effects of macrolides
Y40.400	Harmful effects of tetracyclines
Y40.500	Harmful effects of aminoglycosides
Y40.600	Harmful effects of rifamycins

Y40.700	Harmful effects of systemic antifungal antibiotics
Y40.800	Harmful effects of systemic antibiotics, others
Y40.900	Harmful effects of systemic antibiotics
Y41.000	Harmful effects of sulfonamides
Y41.100	Harmful effects of antimycobacterial drugs
Y41.200	Harmful effects of drugs against malaria and other blood protozoa
Y41.300	Harmful effects of antigen worm drugs, others
Y41.400	Harmful effects of helminth repellents
Y41.500	Harmful effects of antiviral drugs
Y41.800	Harmful effects of systemic anti infectives and antiparasitic drugs, other specific
Y41.900	Harmful effects of systemic anti infective and anti parasitic drugs
Y42.000	Harmful effects of glucocorticoids and their synthetic analogues
Y42.100	Harmful effects of thyroid hormones and their substitutes
Y42.200	Harmful effects of antithyroid drugs
Y42.300	Harmful effects of insulin and oral hypoglycemic (anti diabetes) drugs
Y42.400	Harmful effects of oral contraceptives
Y42.500	Harmful effects of estrogen and progesterone, others
Y42.600	The harmful effects of anti gonadotropins, anti estrogens and anti androgens cannot be classified elsewhere
Y42.700	Harmful Effects of Androgens and Their Metabolic Agents

Y42.800	Harmful effects of hormones and their synthetic substitutes, others
Y42.900	Harmful effects of hormone antagonists, others
Y43.000	Harmful effects of antiallergic and antiemetic drugs
Y43.100	Harmful effects of antineoplastic antimetabolic drugs
Y43.200	Harmful effects of anti-tumor natural products
Y43.300	Harmful effects of antineoplastic drugs, others
Y43.400	Harmful effects of immunosuppressants
Y43.500	Harmful effects of acidification and alkalization agents
Y43.600	The harmful effects of enzymes cannot be classified elsewhere
Y43.800	Mainly harmful effects of systemic preparations, others that cannot be classified elsewhere
Y43.900	Mainly harmful effects of systemic preparations
Y44.000	Harmful effects of iron preparations and other anti hemoglobin anemia preparations
Y44.100	Harmful effects of vitamin B12, folic acid and other anti megaloblastic anemia preparations
Y44.200	Harmful effects of anticoagulants
Y44.300	Harmful Effects of Anticoagulant Antagonists, Vitamin K and Other Coagulants
Y44.400	Harmful effects of antithrombotic drugs [platelet aggregation inhibitors]
Y44.500	Harmful effects of thrombolytic drugs
Y44.600	Harmful effects of natural blood and blood products

Y44.700	Harmful effects of plasma substitutes
Y44.900	Harmful effects of other and unspecified preparations affecting blood composition
Y45.000	Harmful effects of opioids and related analgesics
Y45.100	Harmful effects of salicylates
Y45.200	Harmful effects of propionic acid derivatives
Y45.300	Harmful effects of non steroid anti-inflammatory drugs [NSAID], others
Y45.400	Harmful effects of anti rheumatic drugs
Y45.500	Harmful effects of 4-aminophenol derivatives
Y45.800	Harmful effects of analgesics and antipyretics, others
Y45.900	Harmful effects of analgesics, antipyretics and anti-inflammatory drugs
Y46.000	Harmful effects of succinimide
Y46.100	Harmful effects of oxazolidinedione
Y46.200	Harmful effects of hydantoin derivatives
Y46.300	Harmful Effects of Deoxybarbital Salts
Y46.400	Harmful effects of imino stilbenes
Y46.500	Harmful effects of valproic acid
Y46.600	Harmful effects of antiepileptic drugs, other and unspecified
Y46.700	Harmful effects of antiparkinsonian drugs
Y46.800	Harmful effects of antispasmodics
Y47.000	The harmful effects of barbiturate salts cannot be classified elsewhere
Y47.100	Harmful effects of benzodiazepines

Y47.200	Harmful effects of chloral derivatives
Y47.300	Harmful effects of paraldehyde
Y47.400	Harmful effects of bromine compounds
Y47.500	The harmful effects of mixed sedatives and hypnotics cannot be classified elsewhere
Y47.800	Harmful effects of sedatives, hypnotics and anti anxiety drugs, others
Y47.900	Harmful effects of sedatives, hypnotics and anti anxiety drugs
Y48.000	Harmful effects of inhalation anesthetics
Y48.100	Harmful effects of parenteral anesthetics
Y48.200	Harmful effects of general anesthetics, others
Y48.300	Harmful effects of local anesthetics
Y48.400	Harmful effects of anesthetics
Y48.500	Harmful effects of therapeutic gases
Y49.000	Harmful effects of tricyclic and tetracyclic antidepressants
Y49.100	Harmful effects of monoamine oxidase inhibitor antidepressants
Y49.200	Adverse effects of antidepressants, others
Y49.300	Harmful effects of phenothiazine antipsychotics and antipsychotics
Y49.400	Harmful effects of butylbenzene and thioanthracene antipsychotics
Y49.500	Harmful effects of antipsychotics and antipsychotics, others
Y49.600	Harmful effects of hallucinogens
Y49.700	Harmful effects of mental stimulants with the possibility of abuse

Y49.800	The harmful effects of drugs that have an impact on the spirit, and others cannot be classified elsewhere
Y49.900	The harmful effects of psychoactive drugs
Y50.000	Harmful effects of resuscitation drugs
Y50.100	Harmful effects of opioid receptor antagonists
Y50.200	The harmful effects of methylxanthines cannot be classified elsewhere
Y50.800	Harmful effects of central nervous system stimulants, others
Y50.900	Harmful effects of central nervous system stimulants
Y51.000	Harmful effects of anticholinesterase agents
Y51.100	Harmful effects of parasympathetic drugs [cholinergic drugs], others
Y51.200	The harmful effects of ganglion blockers cannot be classified elsewhere
Y51.300	Parasympathetic depressants [anticholinergics and antimagiotics] harmful effects of antispasmodics, others, which cannot be classified elsewhere
Y51.400	Mainly α The harmful effects of adrenergic receptor drugs can not be classified elsewhere
Y51.500	Mainly β The harmful effects of adrenergic receptor drugs can not be classified elsewhere
Y51.600	α The harmful effects of adrenergic receptor antagonists cannot be classified elsewhere
Y51.700	β The harmful effects of adrenergic receptor antagonists cannot be classified elsewhere

Y51.800	The harmful effects of central action and adrenergic neuron blocker cannot be classified elsewhere
Y51.900	Harmful effects of other and unspecified drugs that mainly affect the autonomic nervous system
Y52.000	Harmful effects of cardiac excitatory glycosides and similar drugs
Y52.100	Harmful effects of calcium channel blockers
Y52.200	Harmful effects of antiarrhythmic drugs, others, which cannot be classified elsewhere
Y52.300	The harmful effects of coronary vasodilators cannot be classified elsewhere
Y52.400	Harmful Effects of Angiotensin Converting Enzyme Inhibitors
Y52.500	Harmful effects of antihypertensive drugs, others, which cannot be classified elsewhere
Y52.600	Harmful effects of anti hyperlipidemia and anti atherosclerosis drugs
Y52.700	Harmful effects of peripheral vasodilators
Y52.800	Harmful effects of anti varicose drugs (including sclerosing agents)
Y52.900	Harmful effects of other and unspecified preparations mainly affecting cardiovascular system
Y53.000	Harmful effects of histamine H2 receptor antagonists
Y53.100	Harmful effects of antacids and anti gastric secretions, others
Y53.200	Harmful effects of irritant laxatives
Y53.300	Harmful effects of saline and osmotic laxatives
Y53.400	Harmful effects of laxatives, others

Y53.500	Harmful effects of digestive aids
Y53.600	Harmful effects of antidiarrheal drugs
Y53.700	Harmful effects of emetics
Y53.800	It mainly affects the harmful effects of gastrointestinal system preparations, and others
Y53.900	It mainly affects the harmful effects of gastrointestinal system preparations
Y54.000	Harmful Effects of Salt (Adrenal) Corticosteroids
Y54.100	Harmful effects of salt (adrenal) corticosteroid antagonists [aldosterone antagonists]
Y54.200	Harmful Effects of Carbonic Dehydratase Inhibitors
Y54.300	Harmful effects of benzothiadiazine derivatives
Y54.400	Harmful effects of diuretics
Y54.500	Harmful effects of diuretics, others
Y54.600	Harmful effects of electrolyte, heat and leveling agent
Y54.700	Harmful effects of preparations affecting calcification
Y54.800	Harmful effects of preparations affecting uric acid metabolism
Y54.900	The harmful effects of mineral salts cannot be classified elsewhere
Y55.000	Harmful effects of oxytocic drugs
Y55.100	Harmful effects of skeletal muscle relaxants [neuromuscular blockers]
Y55.200	Harmful effects of other preparations mainly acting on muscles
Y55.300	Harmful effects of antitussive
Y55.400	Harmful effects of expectorants
Y55.500	Harmful effects of anti cold drugs

Y55.600	The harmful effects of anti asthma drugs cannot be classified elsewhere
Y55.700	Harmful effects of other preparations mainly acting on respiratory system
Y56.000	The harmful effects of local antifungal, anti infective and anti-inflammatory drugs cannot be classified elsewhere
Y56.100	Harmful effects of antipruritic drugs
Y56.200	Harmful effects of local astringents and local detergents
Y56.300	Harmful effects of lubricating agents, moderators and protective agents
Y56.400	Harmful effects of cuticle separating agents, cuticle hyperplasia agents and other hair treatment drugs and preparations
Y56.500	Harmful effects of ophthalmic drugs and preparations
Y56.600	Harmful effects of otorhinolaryngological drugs and preparations
Y56.700	Harmful effects of topical dental drugs
Y56.800	Harmful effects of local preparations, others
Y56.900	Harmful effects of topical preparations
Y57.000	Harmful effects of appetite inhibitors
Y57.100	Harmful effects of anti fatty liver drugs
Y57.200	The harmful effects of antidotes and chelating agents cannot be classified elsewhere
Y57.300	Harmful effects of antialcoholics
Y57.400	Harmful effects of pharmaceutical excipients
Y57.500	Harmful effects of X-ray contrast agent
Y57.600	Harmful effects of diagnostic preparations, others

Y57.700	The harmful effects of vitamins cannot be classified elsewhere
Y57.800	Harmful effects of drugs and agents, others
Y57.900	Harmful effects of drugs or agents
Y58.000	Harmful effects of BCG vaccine
Y58.100	Harmful effects of typhoid and paratyphoid vaccines
Y58.200	Harmful effects of cholera vaccine
Y58.300	Harmful effects of pestis vaccine
Y58.400	Harmful effects of tetanus vaccine
Y58.500	Harmful effects of diphtheria vaccine
Y58.600	Harmful effects of pertussis vaccine (including combined vaccine containing pertussis ingredient)
Y58.800	Harmful effects of combined bacterial vaccines (except those containing pertussis)
Y58.900	Harmful effects of bacterial vaccines, other and unspecified
Y59.000	Harmful effects of viral vaccines
Y59.100	Harmful effects of Rickettsia vaccines
Y59.200	Harmful effects of protozoal vaccines
Y59.300	Harmful effects of immunoglobulin
Y59.800	Harmful effects of vaccines and biological products, other specific
Y59.900	Harmful effects of vaccines or biological products
Y60.000	Unintentional cutting, acupuncture, perforation or bleeding during operation
Y60.100	Unintentional cutting, acupuncture, perforation or bleeding during infusion or blood transfusion

Y60.200	Unintentional cutting, acupuncture, perforation or bleeding during renal dialysis or other perfusion
Y60.300	Unintentional cutting, acupuncture, perforation or bleeding during injection or artificial immunity
Y60.400	Unintentional cutting, puncture, perforation or bleeding during endoscopy
Y60.500	Unintentional cutting, acupuncture, perforation or bleeding during cardiac catheterization
Y60.600	Unintentional cutting, puncture, perforation or bleeding during suction, puncture and other catheter insertion operations
Y60.700	Unintentional cutting, acupuncture, perforation or bleeding during enema
Y60.800	Unintentional cutting, acupuncture, perforation or bleeding in other operations and medical treatment
Y60.900	Unintentional cutting, acupuncture, perforation or bleeding in surgery and medical treatment
Y61.000	Foreign body accidentally left in the body during operation
Y61.100	Foreign bodies accidentally left in the body during infusion or blood transfusion
Y61.200	Foreign bodies accidentally left in the body during renal dialysis or other perfusion
Y61.300	Foreign body accidentally left in the body during injection or artificial immunity
Y61.400	Foreign body accidentally left in the body during endoscopy
Y61.500	Foreign body accidentally left in the body during cardiac catheterization
Y61.600	Foreign bodies accidentally left in the body during aspiration, puncture and other catheter insertion operations
Y61.700	Foreign matters accidentally left in the body when removing the catheter or stuffing
Y61.800	Foreign bodies accidentally left in the body during other operations and medical treatment
Y61.900	Foreign bodies accidentally left in the body during surgery and medical treatment
Y62.000	Failure of aseptic prophylaxis during surgery
Y62.100	Failure of sterile preventive measures in transfusion or blood transfusion
Y62.200	Failure of aseptic prophylaxis during renal dialysis or other perfusion
Y62.300	Failure of sterile preventive measures in injection or artificial immunization
Y62.400	Failure of sterility prevention measures in endoscopy
Y62.500	Failure of aseptic prophylaxis during cardiac catheterization
Y62.600	Failure of aseptic precautions in aspiration, puncture and other catheter insertion operations
Y62.800	Failure of sterile precautions in other operations and medical care
Y62.900	Failure of sterile preventive measures in surgery and medical treatment
Y63.000	To give excessive blood or other liquids in a blood transfusion or infusion
Y63.100	Use of improperly diluted liquid in infusion
Y63.200	Give excessive radiation during treatment

Y63.300	Unintentional exposure of patients to radiation in medical treatment
Y63.400	Improper dosage in electroconvulsive or insulin shock treatment
Y63.500	Improper temperature of local dressing and stuffing
Y63.600	Failure to give necessary drugs, medicaments or biological products
Y63.800	Improper dosage in other operations and medical treatment
Y63.900	Improper dosage in surgery and medical treatment
Y64.000	Medical or biological materials used for blood transfusion or infusion are contaminated
Y64.100	Contamination of medical or biological materials for injection or immunization
Y64.800	Any other form of medical or biological material is contaminated
Y64.900	Contamination of medical or biological materials
Y65.000	Use of mismatched blood in blood transfusion
Y65.100	Using the wrong liquid in infusion
Y65.200	Improper suture or ligation during operation
Y65.300	Incorrect placement of endotracheal tube during anesthesia operation
Y65.400	Improper insertion or removal of other catheters or instruments
Y65.500	Implementation of inappropriate surgery
Y65.800	Other specific accidents in surgery and medical treatment
Y66.x00	No surgery or medical treatment

Y69.x00	Accidents in surgery and medical treatment
Y70.000	Anesthesia diagnosis and monitoring device related to harmful events
Y70.100	Anesthetic treatment and rehabilitation devices related to harmful events (non operative)
Y70.200	Anesthetic prostheses and other implants, materials and accessories related to hazardous events
Y70.300	Anesthetic surgical instruments, materials and devices (including suture) related to hazardous events
Y70.800	Anesthetic multipurpose devices related to hazardous events, which cannot be classified elsewhere
Y71.000	Cardiovascular diagnostic and monitoring devices related to harmful events
Y71.100	Cardiovascular treatment and rehabilitation devices related to harmful events (non operative)
Y71.200	Cardiovascular prostheses and other implants, materials and accessory devices related to harmful events
Y71.300	Cardiovascular surgical instruments, materials and devices (including suture) related to harmful events
Y71.800	Cardiovascular multipurpose devices related to harmful events, which cannot be classified elsewhere
Y72.000	Otolaryngology diagnosis and monitoring device related to harmful events
Y72.100	Otolaryngology treatment and rehabilitation device related to harmful events (non operative)
Y72.200	Otolaryngology prostheses and other implants, materials and accessory devices related to harmful events

Y72.300	Otolaryngology surgical instruments, materials and devices (including sutures) related to harmful events
Y72.800	Otolaryngology multi-purpose devices related to harmful events, which cannot be classified elsewhere
Y73.000	Gastroenterology and urology diagnosis and monitoring device related to harmful events
Y73.100	Gastroenterology and urology treatment and rehabilitation devices related to harmful events (non operative)
Y73.200	Gastroenterology and urology prostheses and other implants, materials and accessory devices related to harmful events
Y73.300	Gastroenterology and urology surgical instruments, materials and devices (including suture) related to harmful events
Y73.800	Gastroenterology and urology multipurpose devices related to harmful events, which cannot be classified elsewhere
Y74.000	Diagnostic and monitoring devices for general hospitals and individuals related to hazardous events
Y74.100	Treatment and rehabilitation devices for general hospitals and individuals related to harmful events (non operative)
Y74.200	Prosthetics and other implants, materials and accessories for general hospitals and individuals related to hazardous events
Y74.300	Surgical instruments, materials and devices (including suture) for general hospitals and individuals related to hazardous events
Y74.800	Multi purpose devices for general hospitals and personal use related to hazardous events, which cannot be classified elsewhere

Y75.000	Neurological diagnosis and monitoring device related to harmful events
Y75.100	Neurological treatment and rehabilitation devices related to harmful events (non operative)
Y75.200	Neurological prostheses and other implants, materials and accessories related to harmful events
Y75.300	Neurological surgical instruments, materials and devices (including suture) related to harmful events
Y75.800	Neurological multipurpose devices related to harmful events, which cannot be classified elsewhere
Y76.000	Gynecology and obstetrics diagnosis and monitoring device related to harmful events
Y76.100	Gynecology and obstetrics treatment and rehabilitation devices related to harmful events (non operative)
Y76.200	Gynecological and obstetric prostheses and other implants, materials and accessories related to harmful events
Y76.300	Gynecological and obstetric surgical instruments, materials and devices (including suture) related to harmful events
Y76.800	Gynecology and obstetrics multipurpose devices related to harmful events, which cannot be classified elsewhere
Y77.000	Ophthalmic diagnostic and monitoring devices related to hazardous events
Y77.100	Ophthalmic treatment and rehabilitation devices related to harmful events (non operative)
Y77.200	Ophthalmic prostheses and other implants, materials and accessories related to hazardous events
Y77.300	Ophthalmic surgical instruments, materials and devices (including sutures) related to hazardous events

Y77.800	Ophthalmic multipurpose devices related to hazardous events, which cannot be classified elsewhere
Y78.000	Radiological diagnostic and monitoring devices related to hazardous events
Y78.100	Radiological treatment and rehabilitation devices related to harmful events (non operative)
Y78.200	Radiological prostheses and other implants, materials and accessory devices related to hazardous events
Y78.300	Radiological surgical instruments, materials and devices (including suture) related to hazardous events
Y78.800	Radiological multipurpose devices related to hazardous events, which cannot be classified elsewhere
Y79.000	Orthopaedic diagnosis and monitoring device related to harmful events
Y79.100	Orthopedic treatment and rehabilitation devices related to harmful events (non operative)
Y79.200	Orthopedic prostheses and other implants, materials and accessories related to harmful events
Y79.300	Orthopedic surgical instruments, materials and devices (including suture) related to harmful events
Y79.800	Orthopedic multipurpose devices related to harmful events, which cannot be classified elsewhere
Y80.000	Physical therapy diagnosis and monitoring device related to harmful events
Y80.100	Physiotherapy and rehabilitation devices related to harmful events (non operative)
Y80.200	Physiotherapy prostheses and other implants, materials and accessories related to hazardous events

Y80.300	Physical therapy surgical instruments, materials and devices (including suture) related to harmful events
Y80.800	Physical therapy multipurpose device related to harmful events, which cannot be classified elsewhere
Y81.000	Diagnostic and monitoring devices for general surgery and plastic surgery related to harmful events
Y81.100	General surgery and plastic surgery treatment and rehabilitation devices related to harmful events (non surgical)
Y81.200	General surgical and plastic prostheses and other implants, materials and accessories related to harmful events
Y81.300	General surgical and plastic surgical instruments, materials and devices (including suture) related to harmful events
Y81.800	General surgery and plastic surgery multipurpose devices related to harmful events, which cannot be classified elsewhere
Y82.000	Other medical diagnosis and monitoring devices related to hazardous events
Y82.100	Other medical treatment and rehabilitation devices related to harmful events (non operative)
Y82.200	Other medical prostheses and other implants, materials and accessories related to hazardous events
Y82.300	Other medical surgical instruments, materials and devices (including suture) related to hazardous events
Y82.800	Other medical multipurpose devices related to hazardous events, which cannot be classified elsewhere
Y83.000	The whole organ transplantation operation is the cause of the patient's abnormal reaction or subsequent

	complications, but the accident was not mentioned at the time of operation
Y83.100	The artificial internal device implantation operation is the cause of the patient's abnormal reaction or subsequent complications, but the accident was not mentioned at the time of operation
Y83.200	Anastomosis, shunt or transplantation operation is the cause of the patient's abnormal reaction or subsequent complications, but the accident was not mentioned at the time of operation
Y83.300	The external stoma formation operation is the cause of the patient's abnormal reaction or subsequent complications, but the accident was not mentioned at the time of operation
Y83.400	The reconstruction operation is the cause of the patient's abnormal reaction or subsequent complications, but the accident was not mentioned at the time of operation
Y83.500	Limb amputation is the cause of the patient's abnormal reaction or subsequent complications, but the accident was not mentioned at the time of operation
Y83.600	The removal of organs (partially) (completely) is the cause of the patient's abnormal reaction or subsequent complications, but the accident was not mentioned at the time of operation
Y83.800	The surgical operation is the cause of the patient's abnormal reaction or subsequent complications, and the accident was not mentioned at the time of operation
Y83.900	The surgical operation is the cause of the patient's abnormal reaction or subsequent complications, but the accident was not mentioned at the time of operation
Y84.000	Cardiac catheterization is the cause of the patient's abnormal reaction or subsequent complications, but
	accidents were not mentioned at the time of operation
Y84.100	Renal dialysis is the cause of the patient's abnormal reaction or subsequent complications, but the accident was not mentioned at the time of operation
Y84.200	Radiological procedures and radiotherapy are the causes of patients' abnormal reactions or subsequent complications, but accidents were not mentioned at the time of operation
Y84.300	Shock treatment is the cause of the patient's abnormal reaction or subsequent complications, but the accident was not mentioned at the time of operation
Y84.400	Fluid aspiration is the cause of the patient's abnormal reaction or subsequent complications, but the accident was not mentioned at the time of operation
Y84.500	The insertion of gastric or duodenal probe is the cause of the patient's abnormal reaction or subsequent complications, but the accident was not mentioned at the time of operation
Y84.600	As the cause of the patient's abnormal reaction or subsequent complications, urinary catheterization was not mentioned at the time of operation
Y84.700	Blood sampling is the cause of the patient's abnormal reaction or subsequent complications, but the accident was not mentioned at the time of operation
Y84.800	The medical operation is the cause of the patient's abnormal reaction or subsequent complications, and the accident was not mentioned at the time of operation
Y84.900	The medical operation is the cause of the patient's abnormal reaction or subsequent complications, but the accident was not mentioned at the time of operation

Y85.000	Aftereffects of motor vehicle accidents
Y85.900	Sequela of transportation accident, other and unspecified
Y86.x00	Aftereffects of accidents, others
Y87.000	Sequela of intentional self harm
Y87.100	Sequela of victimization
Y87.200	Sequela of events with uncertain intentions
Y88.000	Sequela of harmful effects caused by the use of drugs, medicaments and biological products in treatment
Y88.100	The sequela of the patient's accident during the operation and medical operation
Y88.200	Sequela of harmful events related to the use of medical devices in diagnosis and treatment
Y88.300	The sequela of surgery and medical operation are the cause of the patient's abnormal reaction or subsequent complications, but the accident was not mentioned at the time of operation
Y89.000	Legally disposed sequela
Y89.100	Aftereffects of combat operations
Y89.900	Sequela of external causes
Y90.000	Blood alcohol level is lower than 20mg/100ml
Y90.100	The blood alcohol level is 20~39mg/100ml
Y90.200	The blood alcohol level is 40-59mg/100ml
Y90.300	The blood alcohol level is 60-79mg/100ml
Y90.400	The blood alcohol level is 80~99mg/100ml
Y90.500	The blood alcohol level is 100~119mg/100ml
Y90.600	The blood alcohol level is 120~199mg/100ml
Y90.700	The blood alcohol level is 200~239mg/100ml
Y90.800	The blood alcohol level is 240mg/100ml or above
Y90.900	Alcohol in blood
Y91.000	Mild alcoholism
Y91.100	Moderate alcoholism
Y91.200	Severe alcoholism
Y91.300	Extremely serious alcoholism
Y91.900	Alcohol effect
Y95.x00	Iatrogenic condition
Y96.x00	Work related information
Y97.x00	Information related to environmental pollution
Y98.x00	Lifestyle related information

Supplementary Table 3. ICD Code of Severe Trauma Complications

ICD-10 encoding	Name of disease diagnosis
S00.000	Superficial scalp injury
S00.000x053	Organization of scalp hematoma
S00.001	Scalp contusion
S00.002	Scalp abrasions
S00.003	Foreign body on scalp
S00.004	Scalp hematoma

S00.100	Contusion of eyelid and periocular area
S00.100x001	blepharal contusion
S00.100x003	Eyelid congestion
S00.100x006	Contusion of eyebrow arch
S00.101	Contusion of periocular region
S00.102	Frontal hematoma
S00.200	Other superficial injuries of eyelid and periocular area
S00.201	Superficial injury of orbital region
S00.202	Eyelid hematoma
S00.300	Superficial nasal injury
S00.300x001	Superficial injury of nose
S00.300x051	Nasal contusion
S00.302	Nosebleed
S00.400	Superficial ear injury
S00.400x051	Contusion of auricle
S00.400x052	External ear contusion
S00.400x053	Auricular hematoma
S00.401	Contusion of auricle
S00.402	Hematoma of auricle
S00.403	Contusion of tympanum
S00.404	Contusion of tympanic membrane
S00.500	Superficial injury of lips and mouth
S00.500x051	Contusion of lip
S00.500x052	Contusion of mandible
S00.501	Superficial oral injury
S00.700	Multiple superficial head injuries
S00.800	Superficial injuries to other parts of the head

S00.800x041	Superficial facial injury with foreign body
S00.800x053	Contusion of cheek
S00.800x054	Contusion of lower jaw
S00.800x055	Contusion of forehead
S00.800x056	Temporal contusion
S00.801	Facial soft tissue contusion
S00.802	Facial abrasions
S00.803	Facial contusion
S00.804	Superficial foreign body on face
S00.900	Superficial head injury
S00.900x001	Superficial head injury
S01.000	Open wound of scalp
S01.000x002	Laceration of eyebrow arch
S01.001	Scalp laceration
S01.100	Open wound of eyelid and periocular area
S01.100x001	Open injury of orbit
S01.100x002	Foreign body of eyelid caused by trauma
S01.101	Eyelid laceration
S01.102	Open eyelid foreign body
S01.103	Orbital laceration
S01.200	Open wound of nose
S01.200x011	Open wound of nasal epidermis
S01.200x021	Open wound of nostril
S01.200x031	Open injury of nasal septum
S01.200x091	Open wound of nose with sphenoid sinus foreign body
S01.200x092	Traumatic nasal defect
S01.300	Open wound of ear

S01.300x002	Open injury of ear canal
S01.300x011	Open injury of external ear wing
S01.300x012	Open injury of auricle
S01.300x031	Open injury of tragus
S01.300x051	Open injury of eustachian tube
S01.300x061	Open injury of auditory ossicles
S01.300x071	Open injury of middle ear
S01.300x081	Open injury of cochlea
S01.301	Open external auditory canal injury
S01.302	Open postauricular injury with foreign body
S01.400	Open wound of cheek and temporomandibular region
S01.400x011	Open injury of cheek
S01.400x021	Open injury of maxilla
S01.400x031	Open injury of palate
S01.401	Open temporomandibular injury
S01.500	Open wound of lips and mouth
S01.500x001	Open mouth injury
S01.500x021	Open injury of oral mucosa
S01.500x022	Open injury in cheek
S01.500x031	Open injury of gingiva
S01.500x042	Open injury of tongue and floor of mouth
S01.500x051	Open injury of upper palate
S01.500x052	Open injury of soft palate
S01.501	Laceration of tongue
S01.502	Open tongue injury
S01.503	Gingival laceration
S01.504	Open lip injury

S01.505	Penetrating wound of soft palate
S01.506	Laceration of lip
S01.700	Multiple open wounds on the head
S01.800	Open wounds in other parts of the head
S01.800x011	Open craniocerebral injury with fracture
S01.800x021	Open craniocerebral injury with dislocation
S01.800x031	Open craniocerebral injury with intracranial injury
S01.800x081	open brain injury
S01.800x082	Intracranial foreign body
S01.800x083	Open injury of skull
S01.800x085	Facial foreign body
S01.800x086	Open injury of forehead
S01.800x087	Open injury of lower jaw
S01.801	Open intracranial foreign body
S01.802	Facial laceration
S01.803	Open facial injury
S01.804	Open rupture of parotid duct
S01.900	Open wound on the head
S02.000	Skull fornix fracture
S02.000x003	Squamous fracture of temporal bone
S02.000x004	Scaly fracture of frontal bone and temporal bone
S02.000x005	Fracture of frontal bone and parietal bone
S02.001	Frontal bone fracture
S02.002	Fracture of parietal bone
S02.011	Open fracture of frontal bone
S02.012	Open fracture of parietal bone

S02.100	Fracture of skull base
S02.100x001	Skull base fracture
S02.100x002	Fracture of anterior cranial fossa
S02.100x003	Fracture of middle cranial fossa
S02.100x004	Posterior fossa fracture
S02.100x006	Orbital roof fracture
S02.100x008	Frontal sinus fracture
S02.100x009	Sphenoid fracture
S02.101	Occipital fracture
S02.102	Temporal bone fracture
S02.103	Ethmoid sinus fracture
S02.111	Open skull base fracture
S02.112	Open occipital fracture
S02.113	Open temporal bone fracture
S02.114	Open fracture of ethmoid sinus
S02.200	Nasal bone fracture
S02.200x002	Fracture of nasal septum
S02.211	Open nasal bone fracture
S02.300	Orbital floor fracture
S02.300x002	Orbital floor comminuted fracture
S02.311	Open fracture of orbital floor
S02.400	Zygomatic and maxillary fractures
S02.400x001	Zygomatic arch fracture
S02.400x003	Maxillary fracture
S02.400x005	Maxillary sinus fracture
S02.401	Zygomatic fracture
S02.411	Open maxillary fracture
S02.412	Open zygomatic fracture
S02.500	Tooth fracture

S02.500x001	Traumatic tooth fracture
S02.500x002	Traumatic tooth breakage
S02.501	Traumatic tooth loss
S02.600	Mandibular fracture
S02.600x011	Condylar fracture
S02.600x021	Fracture of inferior condyle
S02.600x031	Fracture of coronoid process of mandible
S02.600x041	Mandibular ramus fracture
S02.600x051	Fracture of jaw angle
S02.600x061	Fibrochondral body fracture of mandible
S02.600x081	Mandibular body fracture
S02.600x091	Compound fracture of mandible
S02.600x101	Open condylar fracture
S02.611	Open mandibular fracture
S02.700	Multiple fractures involving skull and facial bone
S02.700x001	Multiple fracture of skull
S02.700x002	Multiple fractures of skull and facial bone
S02.700x004	naso-orbital-ethmoid fracture
S02.701	Multiple facial bone fractures
S02.711	Open multiple facial bone fractures
S02.712	Open multiple skull fractures
S02.800	Skull and facial fractures, others
S02.800x003	Fracture of upper palate
S02.801	Orbital bone fracture
S02.802	Alveolar bone fracture
S02.803	Palatal fracture
S02.810	Specifically refers to open skull and facial bone fractures

S02.811	Open fracture of orbital bone
S02.812	Open alveolar bone fracture
S02.813	Open fracture of palate
S02.900	Skull and facial fractures
S02.900x002	Skull fracture
S02.901	Facial bone fracture
S02.902	Depressed fracture of skull
S02.911	Open skull fracture
S02.912	Open facial bone fracture
S03.000	Dislocation of jaw joint
S03.000x001	Dislocation of jaw cartilage
S03.000x002	Dislocation of mandibular joint
S03.000x003	Temporomandibular joint dislocation
S03.100	Dislocation of nasal septal cartilage
S03.200	Tooth dislocation
S03.200x001	Traumatic tooth dislocation
S03.300	Dislocation of other and unspecified parts of the head
S03.301	Head dislocation
S03.400	Sprain and strain of jaw joint
S03.400x001	Temporomandibular joint injury
S03.400x002	Temporomandibular ligament injury
S03.500	Sprain and strain of joints and ligaments in other and unspecified parts of the head
S03.501	Sprain and strain of head joints and ligaments
S04.000	Injury of optic nerve and optic pathway
S04.000x001	Optic nerve injury
S04.000x002	Optic chiasma injury

S04.000x003	Visual pathway damage
S04.000x004	Damage of visual cortex
S04.100	Injury of oculomotor nerve
S04.200	Trochlear nerve injury
S04.300	Trigeminal nerve injury
S04.400	Abducent nerve injury
S04.400x001	Abducent nerve injury
S04.500	Facial nerve injury
S04.501	Facial nerve rupture
S04.502	Injury of infraorbital nerve
S04.600	Auditory nerve injury
S04.700	Injury of accessory nerve
S04.800	Brain nerve damage, others
S04.801	Hypoglossal nerve injury
S04.802	Olfactory nerve injury
S04.803	Injury of glossopharyngeal nerve
S04.804	Vagus nerve injury
S04.900	Brain Nerve Injury
S05.000	Damage of conjunctiva and corneal abrasion, no foreign body mentioned
S05.000x002	Conjunctival injury
S05.001	Corneal abrasion
S05.002	Corneal abrasion
S05.100	Contusion of eyeball and orbital tissue
S05.100x004	Contusion of eyeball
S05.101	Orbital contusion
S05.102	Traumatic hyphema
S05.103	contusion of lens
S05.104	Scleral contusion

S05.200	Eye laceration and rupture with prolapse or loss of intraocular tissue
S05.200x001	Traumatic vitreous incarceration
S05.200x002	Traumatic vitreous overflow
S05.200x003	Traumatic iridodialysis
S05.200x004	Traumatic iris defect
S05.200x005	Traumatic incarceration of lens
S05.201	Corneal penetrating injury with incarceration of iris
S05.202	Corneal penetrating injury with iris prolapse
S05.203	Corneal penetrating injury with lens incarceration
S05.204	Corneal penetrating injury with vitreous incarceration
S05.205	Traumatic iris prolapse
S05.206	Traumatic incarceration of iris
S05.207	Traumatic iris hernia
S05.208	Traumatic ciliary body prolapse
S05.209	Traumatic vitreous prolapse
S05.210	Traumatic vitreous hernia
S05.300	Eye laceration without prolapse or loss of intraocular tissue
S05.300x003	Conjunctival laceration
S05.300x004	Eyeball rupture
S05.300x005	Traumatic anterior chamber angle splitting
S05.300x010	Laceration of eye
S05.300x011	Lamellar laceration of cornea
S05.301	Corneal laceration
S05.302	Full-thickness corneal laceration
S05.303	Iris laceration
S05.304	Scleral laceration

S05.305	Laceration of ciliary body
S05.400	Orbital penetrating injury with or without foreign body
S05.400x001	Intraorbital foreign body
S05.400x002	Eyemuscle foreign body
S05.401	Orbital penetrating wound
S05.500	Penetrating injury of eyeball with foreign body
S05.500x001	Intraocular foreign body
S05.500x002	Penetrating injury of eyeball with magnetic foreign body
S05.500x003	Penetrating injury of eyeball with non-magnetic foreign body
S05.600	Penetrating injury of eyeball without foreign body
S05.600x002	Penetrating injury of eyeball
S05.601	Penetrating wound of cornea
S05.602	Penetrating injury of iris
S05.603	Penetrating wound of lens
S05.604	Penetrating wound of sclera
S05.605	Penetrating injury of retina
S05.700	Eye avulsion injury
S05.800	Other eye and orbital injuries
S05.800x001	Traumatic iris detachment
S05.800x007	Rupture of extraocular muscles
S05.800x008	Rupture of medial rectus muscle
S05.800x009	Rupture of external rectus muscle
S05.801	Ocular concussion
S05.802	Ocular contusion
S05.803	Corneal injury
S05.804	Iris injury
S05.805	Lens injury

S05.806	Traumatic dislocation of lens
S05.807	Scleral injury
S05.808	Retinal concussion
S05.809	Retinal injury
S05.810	Laceration of lacrimal canaliculus
S05.811	Lacrimal duct injury
S05.812	Shock wave blindness
S05.900	Eye and orbital injuries
S05.900x003	Open eye injury
S05.901	Eye injury
S05.902	Traumatic blindness
S05.903	Vitreous injury
S06.000	cerebral concussion
S06.100	Traumatic cerebral edema
S06.100x001	traumatic brain edema
S06.200	Diffuse brain injury
S06.200x001	Diffuse brain injury
S06.200x002	Diffuse cerebellar injury
S06.200x011	Diffuse brain injury with hemorrhage
S06.200x021	Diffuse cerebellar injury with hemorrhage
S06.200x031	Multiple intracerebral hemorrhage
S06.200x032	Multiple cerebral hematoma
S06.200x033	Multiple cerebellar hematoma
S06.200x081	Multiple cerebral contusion and laceration
S06.200x082	Multiple cerebellar contusion and laceration
S06.201	Brain stem contusion
S06.202	Cerebral contusion

S06.203	Laceration of brain
S06.204	Traumatic brain hernia
S06.205	Traumatic brain compression
S06.206	Diffuse axonal injury
S06.211	Open brain contusion
S06.300	Local brain injury
S06.300x001	Focal brain injury
S06.300x002	Focal cerebellar injury
S06.300x011	Focal cerebral contusion with hemorrhage
S06.300x021	Focal cerebellar contusion with hemorrhage
S06.300x031	Focal cerebral contusion with hematoma
S06.300x032	Focal cerebral contusion with massive hemorrhage
S06.300x041	Focal cerebellar contusion with hematoma
S06.300x042	Focal cerebellar contusion with massive hemorrhage
S06.300x081	Focal cerebral contusion and laceration
S06.300x082	Focal cerebellar contusion and laceration
S06.301	Traumatic focal cerebral hemorrhage
S06.302	Traumatic cerebral hematoma
S06.310	Open focal brain injury
S06.400	Epidural hemorrhage
S06.400x001	Traumatic epidural hematoma
S06.400x002	Traumatic epidural hemorrhage
S06.401	Traumatic closed epidural hematoma
S06.410	Open epidural hemorrhage
S06.500	Traumatic subdural hemorrhage

S06.500x001	Traumatic subdural hemorrhage
S06.500x002	Traumatic subdural hematoma
S06.500x004	Acute traumatic subdural hematoma
S06.500x005	Subacute traumatic subdural hemorrhage
S06.500x006	Subacute traumatic subdural hematoma
S06.501	Traumatic acute subdural hemorrhage
S06.510	Open subdural hemorrhage
S06.600	Traumatic subarachnoid hemorrhage
S06.600x001	Traumatic subarachnoid hemorrhage
S06.600x002	Traumatic subarachnoid hematoma
S06.600x011	Open subarachnoid hemorrhage
S06.610	Open subarachnoid hemorrhage
S06.700	Intracranial injury with prolonged coma
S06.700x001	Mild closed craniocerebral injury
S06.700x002	Closed craniocerebral injury medium
S06.700x003	Severe closed craniocerebral injury
S06.700x004	Closed craniocerebral injury
S06.700x005	Mild open craniocerebral injury
S06.700x006	Open craniocerebral injury medium
S06.700x007	Severe open craniocerebral injury
S06.700x008	Very severe open craniocerebral injury
S06.710	Open intracranial injury with prolonged coma
S06.800	Intracranial injuries, others
S06.800x002	traumatic intracerebral hematoma
S06.800x004	Traumatic cerebellar hematoma
S06.800x005	Traumatic cerebellar contusion
S06.800x007	Traumatic intracranial hematoma
S06.800x009	traumatic intracranial aneurysm
S06.800x010	Traumatic cerebral infarction
S06.800x011	Traumatic intracranial pneumatosis
S06.800x012	Traumatic hydrocephalus
S06.800x013	Traumatic subdural effusion
S06.801	Traumatic cerebellar hemorrhage
S06.802	Traumatic cerebral hemorrhage
S06.803	Traumatic brain stem hemorrhage
S06.804	Traumatic intracranial hemorrhage
S06.805	Traumatic intracranial cavernous sinus injury
S06.811	Open cerebral hemorrhage
S06.812	Open brainstem hemorrhage
S06.813	Open cerebellar hemorrhage
S06.814	Open intracranial hemorrhage
S06.900	Intracranial injury
S06.900x002	Traumatic brain injury
S06.901	Brain stem injury
S06.910	Open intracranial injury
S06.911	Open brain stem injury
S06.912	Open intracranial cavernous sinus injury
S07.000	Facial crush injury
S07.000x001	Face thermal crush injury
S07.100	Cranial crush injury
S07.100x001	Thermal crush injury of skull
S07.800	Crushing injury of other parts of the head
S07.900	Head crush injury
S07.900x001	Head thermal crush injury

S08.000	Scalp avulsion
S08.100	Traumatic ototomy
S08.100x001	Traumatic ototomy
S08.800	Traumatic amputation of other parts of the head
S08.801	Traumatic rhinotomy
S08.900	Traumatic amputation of the head
S09.000	Head vascular injury, which cannot be classified elsewhere
S09.000x001	Head vascular injury
S09.100	Head muscle and tendon injuries
S09.100x001	Head muscle injury
S09.101	Head tendon injury
S09.200	Traumatic rupture of eardrum
S09.200x001	Traumatic perforation of tympanic membrane
S09.700	Multiple head injuries
S09.800	Other specific head injuries
S09.800x002	Traumatic primary teeth injury
S09.800x003	Traumatic sinus hematocele
S09.801	Traumatic nasal septum hematoma
S09.900	Head injury
S09.900x006	Lip injury
S09.901	Facial injury
S09.902	Eyebrow injury
S09.903	Nasal injury
S09.904	Ear injury
S09.905	Auricle injury
S09.906	Tongue injury
S09.907	Salivary gland injury

S10.000	Throat contusion
S10.000x003	Contusion of pharynx
S10.001	Laryngeal contusion
S10.002	Vocal cord contusion
S10.003	Contusion of cervical esophagus
S10.004	Contusion of trachea
S10.100	Other and unspecified superficial injuries of throat
S10.101	Pharyngeal hematoma
S10.102	Superficial injury of throat
S10.700	Multiple superficial injuries in the neck
S10.800	Superficial injuries in other parts of the neck
S10.801	Superficial injury of epiglottis
S10.900	Superficial injury of neck
S10.900x001	Superficial injury of neck
S10.900x041	Superficial injury of neck with foreign body
S10.901	neck injury
S10.902	Foreign body in neck
S11.000	Open wound of neck involving throat and trachea
S11.001	Open trachea injury
S11.002	Open laryngeal injury
S11.003	Open cervical trachea rupture
S11.004	Penetrating injury of larynx and trachea
S11.100	Open wound of neck involving thyroid
S11.100x001	Open injury of thyroid
S11.200	Open wound of neck involving pharynx and cervical esophagus

S11.201	Open pharyngeal injury
S11.202	Open cervical esophageal injury
S11.700	Multiple open wounds on the neck
S11.700x001	Multiple open injuries of neck
S11.800	Open wounds in other parts of the neck
S11.800x011	Open neck injury with cervical fracture
S11.800x021	Open neck injury with cervical dislocation
S11.800x081	Open injury of epiglottis
S11.800x082	Open injury of supraclavicular region
S11.900	Open wound of neck
S11.900x001	Open injury of neck
S12.000	Fracture of the first cervical vertebra
S12.000x002	atlas fracture
S12.010	Open fracture of the first cervical vertebra
S12.100	Fracture of the second cervical vertebra
S12.100x001	Ring vertebra fracture (Jefferson fracture)
S12.100x002	Axis fracture
S12.100x003	Axis pedicle fracture [Hangman fracture]
S12.110	Open fracture of the second cervical vertebra
S12.200	Fracture of cervical vertebra, other specific
S12.200x001	Fracture of odontoid process of axis
S12.200x002	Axis fracture with dislocation (Hangman fracture)
S12.200x011	Cervical vertebra fracture C3
S12.200x021	Cervical vertebra fracture C4
S12.200x031	Cervical vertebra fracture C5

S12.200x041	Cervical vertebra fracture C6
S12.200x051	Cervical vertebra fracture C7
S12.210	Open fracture of cervical vertebra
S12.700	Multiple fractures of cervical vertebra
S12.700x001	Multiple fracture of cervical vertebra
S12.710	Open multiple cervical fractures
S12.800	Fractures in other parts of the neck
S12.800x001	Cricoid cartilage fracture
S12.800x002	Hyoid fracture
S12.800x003	Fracture of laryngeal cartilage
S12.800x004	Fracture of thyroid cartilage
S12.800x005	Fracture of cervical trachea cartilage
S12.801	Rupture of laryngeal cartilage
S12.802	Fracture of thyroid cartilage
S12.803	Hyoid fracture
S12.804	Cricoid cartilage rupture
S12.805	Fracture of trachea cartilage
S12.810	Open fracture of specific part of neck
S12.811	Open rupture of laryngeal cartilage
S12.812	Open rupture of thyroid cartilage
S12.813	Open hyoid fracture
S12.814	Open cricoid cartilage rupture
S12.815	Open trachea cartilage rupture
S12.900	Neck fracture
S12.900x001	Cervical vertebra fracture
S12.900x003	Fracture of cervical nerve arch
S12.900x004	Fracture of spinous process of cervical vertebra
S12.900x005	Fracture of cervical transverse process

S12.900x006	Fracture of cervical vertebra arch
S12.910	Open cervical fracture
S13.000	Traumatic rupture of cervical intervertebral disc
S13.000x001	Traumatic rupture of cervical intervertebral disc
S13.100	Cervical dislocation
S13.100x021	Cervical subluxation C2/C3
S13.100x022	Cervical dislocation C2/C3
S13.100x031	Cervical subluxation C3/C4
S13.100x032	Cervical dislocation C3/C4
S13.100x041	Cervical subluxation C4/C5
S13.100x042	Cervical dislocation C4/C5
S13.100x051	Cervical subluxation C5/C6
S13.100x052	Cervical dislocation C5/C6
S13.100x061	Cervical subluxation C6/C7
S13.100x062	Cervical dislocation C6/C7
S13.100x071	Cervicothoracic subluxation C7/T1
S13.100x072	Cervicothoracic dislocation C7/T1
S13.100x081	Atlanto occipital subluxation
S13.100x082	Dislocation of atlanto occipital joint
S13.101	Cervical subluxation
S13.102	Atlantoaxial subluxation
S13.103	Atlantoaxial dislocation
S13.104	Axis dislocation
S13.200	Dislocation of other and unspecified parts of the neck
S13.200x003	Dislocation of thyroid cartilage
S13.201	Cervical dislocation
S13.202	Dislocation of cricoarytenoid joint

S13.203	Dislocation of cricothyroid cartilage joint
S13.300	Multiple dislocation of neck
S13.400	Sprain and strain of cervical vertebra
S13.400x003	Sprain of anterior longitudinal ligament of neck
S13.400x005	Sprain of atlanto occipital joint
S13.400x006	Cervical joint locking
S13.401	Whiplash wound
S13.402	Sprain of cervical ligament
S13.403	Sprain of atlantoaxial joint
S13.500	Sprain and strain in thyroid region
S13.500x003	Sprain of cricoarytenoid ligament
S13.500x004	Sprain of cricothyroid joint
S13.500x005	Sprain of cricoid ligament
S13.501	Sprain of thyroid cartilage
S13.502	Sprain of cricoarytenoid joint
S13.600	Sprain and strain of joints and ligaments in other and unspecified parts of the neck
S13.601	Neck sprain
S14.000	Concussion and edema of cervical spinal cord
S14.001	Cervical spinal cord edema
S14.002	Cervical spinal cord concussion
S14.100	Other and unspecified injuries of cervical spinal cord
S14.100x011	Complete injury of cervical spinal cord
S14.100x021	Central cervical spinal cord injury syndrome
S14.100x022	Spinal cord central canal syndrome
S14.100x031	Cervical anterior cord syndrome

S14.100x032	Incomplete injury of cervical spinal cord
S14.100x033	Cervical posterior cord syndrome
S14.100x701	Cervical spinal cord function injury
S14.100x711	Cervical spinal cord function injury C1
S14.100x721	Cervical spinal cord function injury C2
S14.100x731	Cervical spinal cord function injury C3
S14.100x741	Cervical spinal cord function injury C4
S14.100x751	Cervical spinal cord function injury C5
S14.100x761	Cervical spinal cord function injury C6
S14.100x771	Cervical spinal cord function injury C7
S14.100x781	Cervicothoracic spinal cord function injury
S14.101	Cervical spinal cord injury
S14.200	Injury of Spinal Process Nerve Root of Cervical Spine
S14.200x001	Injury of cervical spinal nerve root
S14.300	Brachial plexus injury
S14.300x001	Injury of brachial plexus
S14.400	Injury of cervical peripheral nerves
S14.500	Cervical sympathetic nerve injury
S14.600	Injury of other and unspecified nerves in the neck
S14.601	Cervical nerve injury
S15.000	Carotid artery injury
S15.000x002	Traumatic carotid fistula
S15.000x011	Common carotid artery injury
S15.000x021	External carotid artery injury
S15.000x031	Injury of internal carotid artery
S15.001	Internal carotid artery laceration
S15.002	Common carotid artery laceration
S15.003	External carotid artery laceration
S15.004	Traumatic carotid aneurysm
S15.005	Traumatic carotid cavernous fistula
S15.100	Injury of vertebral artery
S15.200	External jugular vein injury
S15.300	Injury of internal jugular vein
S15.301	Rupture of internal jugular vein
S15.700	Multiple vascular injuries at the neck level
S15.800	Other vascular injuries at the neck level
S15.800x002	Traumatic vertebral arteriovenous fistula
S15.800x003	Traumatic thyroid vascular injury
S15.801	Traumatic carotid arteriovenous fistula
S15.900	Vascular injury at the neck level
S15.900x001	Neck vascular injury
S16.x00	Muscle and tendon injuries at the neck level
S16.x00x001	Neck muscle injury
S16.x00x002	Cervical tendon injury
S17.000	Crushing injury of larynx and trachea
S17.000x001	Crushing injury of larynx and trachea
S17.000x002	Laryngeal crush injury
S17.001	Crushing injury of trachea
S17.800	Crushing injury in other parts of the neck
S17.801	Crushing injury of throat
S17.900	Neck crush injury
S18.x00	Traumatic amputation at neck level
S18.x00x001	Behead
S19.700	Multiple injuries in the neck

S19.800	Other specific injuries to the neck
S19.800x002	Injury of cervical trachea
S19.800x004	Injury of cervical thoracic duct
S19.801	Laryngeal injury
S19.802	Laryngeal injury
S19.900	Neck injury
S20.000	Contusion of breast
S20.100	Other and unspecified superficial injuries of breast
S20.101	Superficial breast injury
S20.200	chest trauma
S20.200x003	Contusion of anterior sternum
S20.201	Contusion of chest wall
S20.202	Contusion of interscapular region
S20.300	Other superficial injuries of anterior chest wall
S20.300x001	Superficial injury of anterior chest wall
S20.301	Thoracic skin abrasion
S20.400	Other superficial injuries of posterior chest wall
S20.400x001	Superficial injury of posterior chest wall
S20.700	Multiple superficial chest injuries
S20.800	Superficial injuries to other and unspecified parts of the chest
S20.800x002	Superficial injury of anterior chest border
S20.801	Superficial injury of chest wall
S20.802	Superficial chest injury
S20.803	Scratch of chest wall
S21.000	Open wound of breast

S21.100	Open wound of chest wall
S21.100x002	Open injury of anterior sternum
S21.101	Open costal anterior wall injury
S21.200	Open wound of posterior chest wall
S21.200x001	Open injury of back
S21.200x002	External open injury of chest wall
S21.201	Open posterior chest wall injury
S21.202	Open posterior costal wall injury
S21.203	Open injury of interscapular region
S21.700	Multiple open wounds on chest wall
S21.800	Open wounds in other parts of the chest
S21.800x011	Open chest injury with fracture
S21.800x021	Open chest injury with dislocation
S21.800x031	Open chest injury with intrathoracic injury
S21.900	Open wound on chest
S21.900x001	Open injury of chest wall
S21.900x003	Traumatic foreign body in chest
S21.901	Open chest injury
S22.000	Thoracic vertebra fracture
S22.000x003	Compression fracture of thoracic vertebra
S22.000x005	Fracture of thoracic spinal nerve arch
S22.000x006	Thoracic spine process fracture
S22.000x007	Fracture of transverse process of thoracic vertebra
S22.000x009	Fracture of thoracic vertebral arch
S22.000x011	Thoracic vertebra fracture T1/T2
S22.000x021	Thoracic vertebra fracture T3/T4

S22.000x031	Thoracic vertebra fracture T5/T6
S22.000x041	Thoracic vertebra fracture T7/T8
S22.000x051	Thoracic vertebra fracture T9/T10
S22.000x061	Thoracic vertebra fracture T11/T12
S22.010	Open thoracic vertebral fracture
S22.100	Multiple fractures of thoracic vertebrae
S22.110	Open multiple thoracic vertebral fractures
S22.200	Sternal fracture
S22.210	Open sternal fracture
S22.300	Rib fracture
S22.300x011	Fracture of the first rib
S22.310	Open rib fracture
S22.400	Multiple fractures of rib
S22.400x011	Multiple fracture of rib with first rib fracture
S22.400x021	Two rib fractures without the first rib fracture
S22.400x031	Three rib fractures without first rib fracture
S22.400x041	More than four rib fractures without the first rib fracture
S22.410	Open multiple rib fractures
S22.500	Flail chest
S22.800	Bone fracture of other parts of the thorax
S22.810	Open fracture of specific thoracic region
S22.900	Bone fracture of thorax
S22.900x001	Thoracic fracture
S22.910	Open thoracic fracture
S23.000	Traumatic rupture of thoracic intervertebral disc

S23.100	Dislocation of thoracic vertebra
S23.100x011	Thoracic vertebra dislocation T1/T2
S23.100x012	Thoracic vertebra dislocation T2/T3
S23.100x021	Thoracic vertebra dislocation T3/T4
S23.100x022	Thoracic vertebra dislocation T4/T5
S23.100x031	Thoracic vertebra dislocation T5/T6
S23.100x032	Thoracic vertebra dislocation T6/T7
S23.100x041	Thoracic vertebra dislocation T7/T8
S23.100x042	Thoracic vertebra dislocation T8/T9
S23.100x051	Thoracic vertebra dislocation T9/T10
S23.100x052	Thoracic vertebra dislocation T10/T11
S23.100x061	Thoracic vertebra dislocation T11/T12
S23.100x071	Thoracolumbar dislocation T12/L1
S23.101	Traumatic thoracic disc herniation
S23.200	Dislocation of other and unspecified parts of the chest
S23.200x001	Dislocation of costal joint
S23.200x004	Thoracic tracheal dislocation
S23.200x005	Swordbone cartilage dislocation
S23.201	Dislocation of trachea
S23.202	Dislocation of costal cartilage
S23.203	Sternal dislocation
S23.300	Thoracic spine sprain and strain
S23.400	Rib and sternum sprain and strain
S23.401	Sternum sprain and strain
S23.500	Sprain and strain of other and unspecified parts of the chest
S23.501	Chest sprain
S24.000	Thoracic spinal cord concussion and edema

S24.000x002	Thoracic spinal cord concussion
S24.001	Thoracic spinal cord edema
S24.100	Other and unspecified injuries to the thoracic spinal cord
S24.100x011	Thoracic spinal cord injury
S24.100x021	Anterior funicular syndrome of thoracic spinal cord
S24.100x022	Central thoracic spinal cord injury syndrome
S24.100x023	Incomplete injury of thoracic spinal cord
S24.100x024	Thoracic posterior cord syndrome
S24.100x701	Thoracic spinal cord function injury
S24.100x711	Thoracic spinal cord function injury T1
S24.100x721	Thoracic spinal cord function injury T2/T3
S24.100x731	Thoracic spinal cord function injury T4/T5
S24.100x741	Thoracic spinal cord function injury T6/T7
S24.100x751	Thoracic spinal cord function injury T8/T9
S24.100x761	Thoracic spinal cord function injury T10/T11
S24.100x771	Thoracic spinal cord function injury T12
S24.101	Thoracic spinal cord injury
S24.200	Thoracic spinal nerve root injury
S24.300	Peripheral thoracic nerve injury
S24.300x001	Intercostal nerve injury
S24.400	Thoracic sympathetic nerve injury
S24.400x001	Injury of cardiac plexus
S24.400x002	Injury of esophageal plexus
S24.400x003	Injury of pulmonary plexus

S24.400x004	Stellate plexus injury
S24.400x005	Thoracic sympathetic ganglion injury
S24.500	Injuries to other nerves in the chest
S24.500x001	Phrenic nerve injury
S24.600	Injury of thoracic nerves
S25.000	Thoracic aorta injury
S25.001	Traumatic thoracic aortic aneurysm
S25.100	Injury of innominate artery or subclavian artery
S25.100x002	Innominate artery injury
S25.101	Injury of subclavian artery
S25.200	Injury of superior vena cava
S25.200x001	Vena cava injury
S25.201	Traumatic rupture of superior vena cava
S25.300	Injury of innominate vein or subclavian vein
S25.300x001	Injury of innominate vein
S25.301	Injury of subclavian vein
S25.400	Pulmonary vascular injury
S25.401	Traumatic pulmonary artery rupture
S25.500	Intercostal vascular injury
S25.501	Traumatic intercostal artery rupture
S25.700	Multiple vascular injuries in chest
S25.800	Injury of other blood vessels in the chest
S25.800x001	Breast artery injury
S25.800x003	Breast vein injury
S25.801	Azygos vein injury
S25.802	Traumatic rupture of breast artery

S25.900	Injury of thoracic blood vessels
S25.900x001	Thoracic vascular injury
S26.000	Cardiac injury with pericardium
S26.000x001	Traumatic pericardium
S26.000x002	Traumatic pericardial tamponade
S26.010	Open pericardium
S26.800	Other cardiac injuries
S26.800x011	Cardiac contusion
S26.800x021	Cardiac laceration
S26.800x031	Cardiac laceration with ventricular penetration
S26.800x082	Penetrating injury of heart
S26.800x083	Traumatic cardiac rupture
S26.801	Traumatic pericardial rupture
S26.810	Open heart specific injury
S26.811	Open heart penetrating injury
S26.812	Open heart rupture
S26.813	Cardiac foreign body
S26.900	Heart injury
S26.910	Open heart injury
S27.000	Traumatic pneumothorax
S27.010	Open pneumothorax
S27.100	traumatic haemothorax
S27.110	Open hemothorax
S27.200	Traumatic hemopneumothorax
S27.210	open pneumothorax
S27.300	Other lung injuries
S27.300x012	Pulmonary hematoma
S27.300x081	Foreign body in lung caused by trauma

S27.301	Pulmonary contusion
S27.302	Traumatic pulmonary rupture
S27.303	Traumatic rupture of pulmonary ligament
S27.310	Open lung specific injury
S27.311	Open pulmonary rupture
S27.312	Open pulmonary foreign body
S27.313	Penetrating lung injury
S27.400	Bronchial injury
S27.400x001	Rupture of main bronchus
S27.401	Traumatic bronchial rupture
S27.410	Open bronchial injury
S27.500	Thoracic trachea injury
S27.501	Traumatic thoracic tracheal rupture
S27.510	Open chest trachea injury
S27.600	Pleural injury
S27.610	Open pleural injury
S27.700	Multiple injuries of intrathoracic organs
S27.700x001	Multiple organ injuries in chest
S27.710	Open multiple injuries of intrathoracic organs
S27.800	Intrathoracic organ damage, other specific
S27.800x013	Traumatic mediastinal hematoma
S27.801	Scratch of esophageal mucosa
S27.802	Thoracic esophageal injury
S27.803	Cardiac injury
S27.804	Traumatic diaphragmatic rupture
S27.805	Traumatic diaphragmatic hernia
S27.806	Thoracic lymphatic injury

S27.807	Thymus injury
S27.808	Traumatic pleural effusion
S27.810	Open, especially intrathoracic organ injury
S27.811	Esophageal foreign body perforation
S27.812	Open diaphragmatic rupture
S27.900	Injury of intrathoracic organs
S27.910	Open thoracic foreign body
S28.000	Chest crush injury
S28.100	Partial traumatic thoracotomy
S29.000	Muscle and tendon injuries at the chest level
S29.000x001	Thoracic tendon injury
S29.000x002	Chest muscle injury
S29.700	Multiple chest injuries
S29.700x001	Traumatic rupture of thoracic duct
S29.800	Other specific injuries to the chest
S29.900	Chest injury
S30.000	Contusion of lower back and pelvis
S30.000x001	back contusion
S30.000x003	Lumbar contusion
S30.000x004	Contusion of sacral region
S30.001	Low back contusion
S30.002	Sacrococcygeal contusion
S30.003	Contusion of buttock
S30.100	Contusion of abdominal wall
S30.100x001	Abdominal contusion
S30.100x002	Contusion of flank
S30.100x004	Contusion of iliac region

S30.100x005	Contusion of inguinal region
S30.100x007	Hematoma of iliac fossa
S30.101	Traumatic iliac hematoma
S30.102	Traumatic iliopsoas hematoma
S30.104	Contusion of groin
S30.200	Contusion of external genitalia
S30.200x005	Contusion of perineum
S30.200x006	Contusion of vulva
S30.200x007	Contusion of labia majora
S30.200x008	Contusion of labia minora
S30.200x010	Traumatic hematoma of vulva
S30.201	Traumatic perineal hematoma
S30.202	Traumatic scrotal hematoma
S30.203	Traumatic epididymal hematoma
S30.205	Contusion of scrotum
S30.206	Penis contusion
S30.207	Vaginal contusion
S30.208	Testicular contusion
S30.700	Multiple superficial injuries of abdomen, lower back and pelvis
S30.800	Other superficial injuries to the abdomen, lower back and pelvis
S30.800x001	Superficial injury of hip
S30.800x002	Superficial injury of upper abdomen
S30.800x003	Superficial injury of external genitalia
S30.800x004	Superficial injury of flank
S30.801	Superficial foreign body of abdominal wall
S30.900	Superficial injury of abdomen, lower back and pelvis

S30.900x001	Superficial abdominal injury
S30.900x002	Superficial injury of lower back
S30.900x003	Superficial injury of pelvis
S31.000	Open wound of lower back and pelvis
S31.000x003	Open injury of sacrum
S31.000x004	Open injury of pelvis
S31.000x005	Open injury of lower back
S31.000x006	Open injury of buttock with foreign body
S31.001	Traumatic laceration of perineum
S31.002	Skin avulsion injury of lumbar and back
S31.003	Open perineal injury
S31.004	Open hip injury
S31.005	Open lumbar and back injury
S31.006	Foreign body in buttock
S31.100	Open wound of abdominal wall
S31.100x002	Open injury of upper abdomen
S31.100x003	Open injury of flank
S31.100x005	Open injury of iliac region
S31.100x006	Open injury of inguinal region
S31.100x007	Open injury of vulva
S31.101	Open costal injury
S31.102	Open inguinal injury
S31.200	Open wound of penis
S31.300	Open wound of scrotum and testis
S31.300x001	Open injury of scrotum
S31.300x002	Testicular rupture
S31.301	Open testicular injury
S31.400	Open wound of vagina and vulva

S31.400x001	Open injury of vagina
S31.400x002	Open injury of clitoris
S31.400x003	Open injury of vulva
S31.401	Traumatic laceration of vulva
S31.402	Hymen laceration
S31.500	Open wound of external genitalia, other and unspecified
S31.501	Open external genital injury
S31.700	Multiple open wounds in abdomen, lower back and pelvis
S31.800	Open wounds in other and unspecified parts of the abdomen
S31.800x003	Traumatic abdominal foreign body
S31.800x011	Open injury of lower back with fracture
S31.800x012	Open injury of pelvis with fracture
S31.800x021	Open injury of lower back with dislocation
S31.800x022	Open injury of pelvis with dislocation
S31.800x031	Open abdominal injury with intra-abdominal organ injury
S31.801	Open abdominal injury
S31.802	Open abdominal foreign body
S31.803	Penetrating wound of vagina and rectum
S31.804	Traumatic laceration of anal sphincter
S31.805	Traumatic anal laceration
S32.000	Lumbar fracture
S32.000x002	Lumbar compression fracture
S32.000x011	Lumbar fracture L1
S32.000x021	Lumbar fracture L2
S32.000x031	Lumbar fracture L3
S32.000x041	Lumbar fracture L4

S32.000x051	Lumbar fracture L5
S32.010	Open lumbar fracture
S32.100	Sacral fracture
S32.110	Open sacral fracture
S32.200	Coccyx fracture
S32.210	Open coccyx fracture
S32.300	Iliac fracture
S32.310	Open iliac fracture
S32.400	Acetabular fracture
S32.410	Open acetabular fracture
S32.500	Pubic fracture
S32.500x002	Fracture of pubic branch
S32.500x003	Fracture of pubic symphysis
S32.510	Open pubic fracture
S32.700	Multiple fractures of lumbar spine and pelvis
S32.701	Multiple pelvic fractures
S32.702	Multiple lumbar fractures
S32.710	Open multiple fractures of lumbar spine and pelvis
S32.711	Open multiple pelvic fractures
S32.712	Open multiple lumbar fractures
S32.800	Fractures of the lumbar spine and other unspecified parts of the pelvis
S32.800x021	Lumbosacral spinous process fracture
S32.800x022	Fracture of lumbosacral transverse process
S32.800x023	Fracture of lumbosacral arch
S32.800x024	Lumbosacral vertebra fracture
S32.800x091	Fracture of pelvic joint
S32.800x092	Lateral compression fracture of pelvis
S32.800x093	Open book fracture of pelvis
S32.800x094	Vertical shear fracture of pelvis
S32.800x095	Malgeney fracture
S32.801	Ischial fracture
S32.802	Pelvic fracture
S32.803	Lumbosacral spinal fracture
S32.810	Open fracture of lumbar spine and pelvis at specific points
S32.811	Open ischial fracture
S32.812	open pelvic fracture
S32.813	Open fracture of lumbosacral spine
S33.000	Traumatic rupture of lumbar intervertebral disc
S33.100	Lumbar dislocation
S33.100x011	Lumbar dislocation L1/L2
S33.100x021	Lumbar dislocation L2/L3
S33.100x031	Lumbar dislocation L3/L4
S33.100x041	Lumbar dislocation L4/L5
S33.100x051	Lumbosacral dislocation L5/S1
S33.200	Dislocation of sacroiliac joint and sacrococcygeal joint
S33.200x001	Dislocation of coccyx
S33.200x002	Dislocation of sacrum
S33.200x003	Dislocation of sacroiliac joint
S33.201	Dislocation of sacrococcygeal joint
S33.300	Dislocation of the lumbar spine and other unspecified parts of the pelvis
S33.300x001	Dislocation of pubic symphysis
S33.300x004	Dislocation of pelvis

S33.301	Dislocation of lumbar spine and pelvis
S33.400	Traumatic rupture of pubic symphysis
S33.400x001	Traumatic rupture of pubic symphysis
S33.500	Lumbar sprain and strain
S33.500x011	Lumbosacral joint sprain
S33.501	Lumbar sprain
S33.502	Lumbar sprain
S33.600	Sprain and strain of sacroiliac joint
S33.600x001	Sprain of sacroiliac joint
S33.601	Sprain of sacral joint
S33.700	Sprain and strain of lumbar spine and other unspecified parts of pelvis
S33.700x001	Sacrococcygeal ligament strain
S33.700x002	Injury of supraspinous ligament
S33.700x003	Lumbar joint sprain
S33.701	Sprain of sacroiliac region
S33.702	Sprain of pubic symphysis
S33.703	Sprain and strain of lumbar spine and pelvis
S34.000	Lumbar spinal cord concussion and edema
S34.000x002	Lumbar spinal cord concussion
S34.001	Lumbar spinal cord edema
S34.100	Other injuries of lumbar spinal cord
S34.100x001	Lumbar spinal cord injury
S34.100x002	Complete injury of lumbar spinal cord
S34.100x003	Incomplete injury of lumbar spinal cord
S34.100x701	Lumbar spinal cord function injury
S34.100x711	Lumbar spinal cord function injury L1
S34.100x721	Lumbar spinal cord function injury L2

S34.100x731	Lumbar spinal cord function injury L3
S34.100x741	Lumbar spinal cord function injury L4
S34.100x751	Lumbar spinal cord function injury L5
S34.100x761	Sacral spinal cord function injury
S34.200	Injury of lumbosacral nerve root
S34.200x001	Sacral spinal nerve root injury
S34.200x002	Injury of lumbar spinal nerve root
S34.300	Cauda equina injury
S34.400	Lumbosacral plexus injury
S34.500	Waist, sacrum and pelvic sympathetic nerve injury
S34.500x001	Abdominal sympathetic ganglion injury
S34.500x004	Sympathetic nerve injury of inferior mesenteric plexus
S34.500x005	Sympathetic nerve injury of superior mesenteric plexus
S34.500x007	Waist, sacrum and pelvic sympathetic nerve injury
S34.501	Celiac plexus injury
S34.502	Hypogastric plexus injury
S34.503	Mesenteric plexus injury
S34.504	Visceral nerve injury
S34.600	Abdominal, lower back and pelvic peripheral nerve injuries
S34.601	Injury of lower dorsal peripheral nerve
S34.602	Peripheral pelvic nerve injury
S34.800	Other and unspecified nerve injuries at the abdominal, lower back and pelvic levels
S34.800x001	Lumbosacral nerve injury
S34.801	Abdominal nerve injury
S34.802	Injury of inferior dorsal nerve

S34.803	Pelvic nerve injury
S35.000	Abdominal aorta injury
S35.001	Traumatic abdominal aortic aneurysm
S35.100	Injury of inferior vena cava
S35.100x003	Hepatic vein injury
S35.101	Traumatic rupture of inferior vena cava
S35.102	Traumatic rupture of hepatic vein
S35.200	Celiac or mesenteric artery injury
S35.200x001	Celiac artery injury
S35.200x003	Gastroduodenal artery injury
S35.200x004	Hepatic artery injury
S35.200x005	Injury of inferior mesenteric artery
S35.200x006	Injury of superior mesenteric artery
S35.200x007	Splenic artery injury
S35.201	Mesenteric artery injury
S35.202	Gastric artery injury
S35.203	Traumatic rupture of gastric artery
S35.204	Traumatic rupture of hepatic artery
S35.205	Traumatic rupture of splenic artery
S35.300	Injury of portal vein or splenic vein
S35.300x001	Portal vein injury
S35.300x002	Splenic vein injury
S35.300x003	Injury of inferior mesenteric vein
S35.300x004	Injury of superior mesenteric vein
S35.300x005	Mesenteric vein injury
S35.301	Traumatic mesenteric vein rupture
S35.302	Traumatic rupture of splenic vein
S35.400	Renal vascular injury

S35.400x001	Renal artery injury
S35.400x002	Renal vein injury
S35.401	Traumatic rupture of renal vein
S35.402	Traumatic rupture of renal artery
S35.500	Injury of iliac vessels
S35.500x001	Injury of iliac artery
S35.500x002	Traumatic thrombosis of common iliac artery
S35.500x003	Traumatic iliac arteriovenous fistula
S35.500x004	Injury of iliac vein
S35.500x005	Uterine artery injury
S35.500x006	Injury of uterine vein
S35.500x007	Injury of inferior abdominal artery
S35.500x008	Injury of inferior abdominal vein
S35.501	Traumatic rupture of iliac artery
S35.502	Traumatic rupture of iliac vein
S35.503	Traumatic uterine arteriovenous rupture
S35.700	Multiple vascular injuries at the abdominal, lower back and pelvic levels
S35.700x001	Multiple vascular injuries in abdomen, lower back and pelvis
S35.700x003	Injury of presacral venous plexus
S35.700x004	Mesenteric vascular injury
S35.701	Multiple abdominal vascular injuries
S35.800	Other vascular injuries at the abdominal, lower back and pelvic levels
S35.800x001	Ovarian artery injury
S35.800x002	Injury of ovarian vein
S35.801	Ovarian arteriovenous injury

S35.900	Injury of blood vessels at the abdominal, lower back and pelvic levels
S35.900x001	Abdominal vascular injury
S35.901	Injury of inferior dorsal vessels
S35.902	Pelvic vascular injury
S35.903	Traumatic mesenteric vascular injury
S36.000	Splenic injury
S36.000x021	Tear of splenic capsule
S36.000x031	Spleen laceration with soft tissue injury
S36.000x081	Penetrating injury of spleen
S36.001	Traumatic splenic hematoma
S36.002	Traumatic rupture of spleen
S36.011	Open rupture of spleen
S36.100	Liver or gallbladder injury
S36.100x001	Liver injury
S36.100x011	Hepatic contusion
S36.100x013	Traumatic hepatic hematoma
S36.100x021	Laceration of liver
S36.100x031	Mild laceration of liver
S36.100x041	Moderate laceration of liver
S36.100x051	Severe laceration of liver
S36.100x081	Bile duct injury
S36.101	Gallbladder injury
S36.102	Traumatic liver rupture
S36.103	Traumatic rupture of common bile duct
S36.110	Open rupture of liver
S36.111	Open gallbladder injury
S36.112	Open bile duct injury
S36.113	Open common bile duct injury

S36.200	Pancreatic injury
S36.200x001	Pancreatic injury
S36.200x011	Pancreatic head injury
S36.200x021	Pancreatic body injury
S36.200x031	Pancreatic tail injury
S36.200x091	Pancreatic duct injury
S36.200x092	Injury of pancreas and pancreatic duct
S36.201	Traumatic pancreatic rupture
S36.202	Tear of pancreatic capsule
S36.210	Open pancreatic injury
S36.300	Gastric injury
S36.301	Traumatic gastric rupture
S36.310	Open gastric rupture
S36.400	Small intestine injury
S36.400x091	Jejunum injury
S36.400x093	Ileal injury
S36.400x095	Multiple injuries of small intestine
S36.401	Traumatic rupture of duodenum
S36.402	Traumatic jejunal rupture
S36.403	Traumatic rupture of ileum
S36.404	Traumatic rupture of small intestine
S36.405	injury of duodenum
S36.411	Open rupture of small intestine
S36.412	Open rupture of duodenum
S36.413	Open jejunal rupture
S36.414	Open ileal rupture
S36.500	Colon injury
S36.500x011	Ascending colon injury
S36.500x021	Transverse colon injury

S36.500x031	Descending colon injury
S36.500x041	Sigmoid injury
S36.500x091	Multiple injuries of colon
S36.500x092	Appendiceal injury
S36.500x093	Cecal injury
S36.501	Traumatic rupture of colon
S36.511	Open rupture of colon
S36.600	Rectal injury
S36.600x003	Multiple rectal injuries
S36.601	Traumatic rectal rupture
S36.611	Open rectal rupture
S36.700	Multiple intra-abdominal organ injuries
S36.701	Traumatic intra-abdominal multiple organ rupture
S36.800	Intraabdominal organ damage, others
S36.800x022	mesenteric injury
S36.801	Peritoneal injury
S36.802	Mesenteric laceration
S36.803	Traumatic retroperitoneal hematoma
S36.810	Open, especially intra-abdominal organ injury
S36.811	Open mesenteric hematoma
S36.812	Open mesenteric laceration
S36.813	Open retroperitoneal hematoma
S36.814	Open rupture of greater omentum
S36.900	Injury of intra-abdominal organs
S36.900x001	Intra-abdominal organ injury
S36.901	Traumatic intestinal rupture
S36.910	Open intra-abdominal organ injury

S37.000	Renal injury
S37.000x012	Contusion of renal capsule
S37.000x013	Contusion of renal pelvis
S37.000x015	Hematoma of renal cyst
S37.000x016	Hematoma of renal pelvis
S37.000x021	Renal laceration
S37.000x022	Rupture of renal capsule
S37.000x023	Laceration of renal pelvis
S37.000x031	Renal comminuted injury
S37.000x041	Renal pedicle injury
S37.001	Traumatic renal rupture
S37.002	Renal contusion
S37.003	Traumatic renal hematoma
S37.004	Traumatic perirenal hematoma
S37.010	Open renal injury
S37.011	Open renal rupture
S37.100	Ureteral injury
S37.101	Traumatic rupture of ureter
S37.111	Open ureteral rupture
S37.200	Bladder injury
S37.200x011	Contusion of bladder
S37.200x022	Extraperitoneal bladder rupture
S37.200x023	Rupture of intraperitoneal bladder
S37.200x024	Mixed bladder rupture
S37.200x081	Laceration of bladder
S37.201	Traumatic bladder rupture
S37.211	Open bladder rupture
S37.300	Urethral injury

S37.300x004	Complete urethral rupture
S37.300x005	Partial urethral rupture
S37.300x011	Injury of urethral membrane
S37.300x021	Injury of urethral cavernous body
S37.300x031	Injury of urethra and prostate
S37.300x081	Rupture of urethral bulb
S37.300x082	Contusion and laceration of urethral bulb
S37.300x083	Posterior urethral injury
S37.301	Traumatic urethral rupture
S37.302	Urethral contusion
S37.303	Urethral injury with stricture
S37.310	Open urethral injury
S37.400	Ovarian injury
S37.410	Open ovarian injury
S37.500	Fallopian tube injury
S37.510	Open tubal injury
S37.600	Uterine injury
S37.600x002	Traumatic rupture of uterus
S37.601	Traumatic cervical laceration
S37.602	Traumatic uterine perforation
S37.610	Open uterine injury
S37.700	Multiple pelvic organ injuries
S37.710	Open multiple pelvic organ injury
S37.800	Pelvic organ damage, others
S37.801	Vasectomy injury
S37.802	Seminal vesicle injury
S37.803	Adrenal injury
S37.804	Prostate injury

S37.810	Open, especially pelvic organ injury
S37.811	Open injury of vas deferens
S37.812	Open seminal vesicle injury
S37.813	Open adrenal injury
S37.814	Open prostate injury
S37.900	Injury of pelvic organs
S37.910	Open pelvic organ injury
S38.000	Crushing injury of external genitalia
S38.000x001	External genitalia thermal crush injury
S38.000x002	Thermal crush injury of penis
S38.001	Crush wound of penis
S38.100	Crushing injury of abdomen, lower back and other unspecified parts of pelvis
S38.100x002	Abdominal crush injury
S38.100x003	Crushing injury of lower back
S38.100x004	Pelvic crush injury
S38.100x011	Thermal crush injury of abdomen, lower back and pelvis
S38.101	Crushing injury of abdomen, lower back and pelvis
S38.200	Traumatic amputation of external genitalia
S38.200x001	Labia majora amputation
S38.200x002	Labia minora amputation
S38.200x003	Penile amputation
S38.200x004	Scrotal amputation
S38.200x005	Testicular amputation
S38.200x006	Vulva amputation
S38.300	Traumatic amputation of abdomen, lower back and other unspecified parts of pelvis
S38.300x001	Torso amputation

S38.300x002	Abdominal amputation
S38.301	Traumatic thoracotomy
S38.302	Traumatic lower dorsal amputation
S38.303	Traumatic pelvic amputation
S39.000	Abdominal, lower back and pelvic muscle and tendon injuries
S39.000x001	Abdominal muscle injury
S39.000x002	Abdominal tendon injury
S39.000x003	Lower back muscle injury
S39.000x004	Injury of tendon of inferior dorsal muscle
S39.000x005	Pelvic muscle injury
S39.000x006	Pelvic tendon injury
S39.001	Rupture of rectus abdominis
S39.002	Open rupture of psoas major muscle
S39.003	Lower back soft tissue injury
S39.004	Pelvic soft tissue injury
S39.600	Abdominal organs accompanied by pelvic organ damage
S39.600x001	Abdominal organs with pelvic organ damage
S39.700	Multiple injuries in abdomen, lower back and pelvis
S39.800	Other specific injuries to the abdomen, lower back and pelvis
S39.800x001	Abdominal soft tissue injury
S39.800x002	Lower back soft tissue injury
S39.800x004	Soft tissue injury of abdomen, lower back and pelvis
S39.900	Abdominal, lower back and pelvis injuries
S39.900x002	Lower back injury
S39.900x004	Inguinal injury

S39.900x007	Scrotal injury
S39.900x009	Epididymal injury
S39.900x010	Testicular injury
S39.901	Hymen damage
S39.902	Vaginal injury
S39.903	Perineal injury
S39.904	Penis injury
S39.905	Anal injury
S39.906	Lumbar injury
S39.907	Abdominal injury
S39.908	Pelvic injury
S39.909	Metallic foreign body in abdomen
S39.910	Lumbar soft tissue injury
S39.911	Abdominal soft tissue injury
S39.912	Hip soft tissue injury
S40.000	Contusion of shoulder and upper arm
S40.000x001	Contusion of shoulder
S40.000x002	Contusion of scapular region
S40.000x003	Contusion of axillary region
S40.001	Upper arm contusion
S40.700	Multiple superficial injuries of shoulder and upper arm
S40.701	Multiple contusions of shoulder and arm
S40.800	Other superficial injuries of shoulder and upper arm
S40.800x011	Shoulder scratch
S40.800x012	Upper arm abrasions
S40.800x021	Shoulder blister
S40.800x022	Vesicle of upper arm

S40.800x031	Shoulder insect bite
S40.800x032	Upper armworm bite
S40.800x041	Superficial foreign body of shoulder
S40.800x042	Foreign body on the surface of upper arm
S40.900	Superficial injury of shoulder and upper arm
S41.000	Open wound of shoulder
S41.000x002	Open injury of scapular band
S41.100	Open wound of upper arm
S41.700	Multiple open wounds on shoulder and upper arm
S41.800	Open wound of other and unspecified parts of scapular band
S41.800x001	Open injury of axilla
S41.800x011	Open injury of shoulder with fracture
S41.800x012	Open injury of upper arm with fracture
S41.800x021	Open injury of shoulder with dislocation
S41.800x022	Open injury of upper arm with dislocation
S41.801	Open injury of scapular region
S41.802	Open shoulder belt injury
S42.000	Clavicle fracture
S42.000x011	Fracture of sternal end of clavicle
S42.000x021	Fracture of clavicular shaft
S42.000x031	Fracture of acromion end of clavicle
S42.000x091	Multiple fracture of clavicle
S42.010	Open clavicular fracture
S42.100	Scapular fracture
S42.100x011	Fracture of scapular body
S42.100x021	Acromion fracture

S42.100x031	Fracture of coracoid process of scapula
S42.100x041	Fracture of scapular neck and glenoid
S42.100x042	Glenoid fracture of shoulder
S42.100x091	Multiple fractures of scapula
S42.110	Open scapular fracture
S42.200	Fracture of upper humerus
S42.200x001	proximal humeral fractures
S42.200x011	Separation of proximal humeral epiphysis
S42.200x031	Fracture of anatomical neck of humerus
S42.200x041	Fracture of greater tubercle of humerus
S42.200x091	Fracture of humeral tubercle
S42.200x092	Multiple fractures of proximal humerus
S42.200x101	Fracture of humeral neck
S42.202	Fracture of surgical neck of humerus
S42.203	Fracture of humeral head
S42.210	Open fracture of upper humerus
S42.300	Fracture of humeral shaft
S42.300x002	Multiple fractures of humeral shaft
S42.301	Fracture of humerus
S42.310	Open fracture of humeral shaft
S42.311	Open fracture of humerus
S42.400	Fracture of lower end of humerus
S42.400x001	Fracture of distal humerus
S42.400x041	Fracture of medial epicondyle of humerus
S42.400x042	Fracture of lateral epicondyle of humerus
S42.400x043	Separation of distal humeral epiphysis
S42.400x051	T-shaped fracture of distal humerus

S42.400x091	Multiple fractures of distal humerus
S42.400x092	Fracture of trochlear of humerus
S42.400x093	Fracture of capitulum of humerus
S42.401	Supracondylar fracture of humerus
S42.402	Fracture of lateral condyle of humerus
S42.403	Intercondylar fracture of humerus
S42.404	Fracture of medial condyle of humerus
S42.410	Open fracture of lower humerus
S42.700	Multiple fractures of clavicle, scapula and humerus
S42.710	Open multiple fractures of clavicle, scapula and humerus
S42.800	Fracture of shoulder and other parts of upper arm
S42.810	Open fracture of shoulder and upper arm
S42.900	Fracture of scapular band
S42.910	Open shoulder fracture
S43.000	Dislocation of shoulder joint
S43.000x011	Anterior dislocation of humerus
S43.000x021	Posterior dislocation of humerus
S43.000x031	Subluxation of humerus
S43.000x041	Anterior dislocation of shoulder joint
S43.000x042	Posterior dislocation of shoulder joint
S43.001	shoulder subluxation
S43.002	Dislocation of glenohumeral joint
S43.100	Acromioclavicular joint dislocation
S43.200	Dislocation of sternoclavicular joint
S43.300	Dislocation of other and unspecified parts of scapular band
S43.301	Dislocation of scapula

S43.302	Dislocation of scapular band
S43.400	Sprain and strain of shoulder joint
S43.400x001	Sprain of shoulder joint
S43.400x003	Sprain of rotator cuff joint capsule
S43.400x004	Coracohumeral ligament sprain
S43.400x011	Tear of shoulder joint capsule
S43.400x012	Injury of anterior and posterior scapular glenoid (SLAP injury)
S43.400x013	Anterior and inferior injury of scapular glenoid (bankart injury)
S43.401	Strain of shoulder joint
S43.500	Sprain and strain of acromioclavicular joint
S43.500x001	Sprain of acromioclavicular joint
S43.500x002	Sprain of acromioclavicular ligament
S43.501	Injury of acromioclavicular ligament
S43.600	Thoracoclavicular joint sprain and strain
S43.601	Thoracoclavicular joint sprain
S43.700	Sprain and strain of other and unspecified parts of shoulder girdle
S43.700x002	Injury of glenoid lip of shoulder joint
S43.700x011	Sprain and strain in other parts of shoulder girdle
S43.701	Sprain of scapular band
S44.000	Ulnar nerve injury at upper arm level
S44.000x001	Ulnar nerve injury of upper arm
S44.100	Median nerve injury at upper arm level
S44.100x001	Injury of median nerve of upper arm
S44.101	Rupture of median nerve of upper arm
S44.200	Radial nerve injury at upper arm level

S44.200x001	Injury of radial nerve of upper arm
S44.300	Axillary nerve injury
S44.400	Myocutaneous nerve injury
S44.500	Dermatosensory nerve injury at shoulder and upper arm level
S44.500x001	Injury of medial arm cutaneous nerve
S44.500x002	Injury of medial forearm cutaneous nerve
S44.501	Upper arm cutaneous sensory nerve injury
S44.700	Multiple nerve injuries at the shoulder and upper arm levels
S44.700x001	Multiple nerve injuries of shoulder and upper arm
S44.701	Multiple nerve injuries of upper arm
S44.800	Other nerve injuries at the shoulder and upper arm levels
S44.800x001	Injury of suprascapular nerve
S44.900	Nerve injury at shoulder and upper arm level
S44.900x001	Nerve injury of shoulder and upper arm
S44.901	Upper arm nerve injury
S45.000	Axillary artery injury
S45.001	Traumatic rupture of axillary artery
S45.100	Injury of brachial artery
S45.101	Traumatic brachial artery injury
S45.200	Injury of axillary or brachial vein
S45.200x002	Injury of brachial vein
S45.201	Traumatic injury of axillary vein
S45.300	Superficial vein injury at shoulder and upper arm level
S45.300x001	Superficial vein injury of shoulder and upper arm
S45.300x002	Injury of superficial vein of shoulder

S45.301	Superficial vein injury of upper arm
S45.700	Multiple vascular injuries at the shoulder and upper arm levels
S45.700x001	Multiple vascular injuries of shoulder and upper arm
S45.701	Multiple vascular injuries of upper arm
S45.800	Other vascular injuries at the shoulder and upper arm levels
S45.900	Injury of blood vessels at the shoulder and upper arm levels
S45.900x001	Vascular injury of shoulder and upper arm
S46.000	Injury of rotator cuff tendon of shoulder
S46.002	Shoulder sleeve damage
S46.100	Injury of biceps longus muscle and tendon
S46.100x001	Injury of long head of biceps brachii
S46.100x002	Injury of tendon of long head of biceps brachii
S46.100x003	Injury of long head muscle and tendon of biceps brachii
S46.101	Tendon injury of biceps longus
S46.200	Muscle and tendon injuries in other parts of biceps
S46.200x001	Biceps brachii muscle injury
S46.200x002	Injury of biceps tendon
S46.200x003	Injury of biceps muscle and tendon
S46.201	Traumatic rupture of biceps brachii
S46.300	Injury of triceps muscle and tendon
S46.300x001	Muscle injury of triceps brachii
S46.300x002	Injury of triceps tendon
S46.300x003	Injury of triceps muscle and tendon
S46.301	Traumatic rupture of triceps brachii

S46.700	Multiple muscle and tendon injuries at the shoulder and upper arm levels
S46.700x001	Multiple muscle injuries of shoulder and upper arm
S46.700x002	Multiple tendon injuries of shoulder and upper arm
S46.701	Multiple tendon injuries of shoulder
S46.702	Multiple tendon injuries of upper arm
S46.800	Other muscle and tendon injuries at the shoulder and upper arm levels
S46.800x001	Deltoid injury
S46.800x002	Injury of supraspinatus muscle
S46.800x003	Injury of supraspinatus tendon
S46.800x004	Injury of infraspinatus muscle
S46.800x005	Injury of infraspinatus tendon
S46.800x006	Injury of subscapularis muscle
S46.800x007	Injury of subscapularis tendon
S46.801	Traumatic rupture of supraspinatus muscle
S46.802	Traumatic rupture of deltoid muscle
S46.900	Injuries to muscles and tendons at the shoulder and upper arm levels
S46.900x001	Muscle injury of shoulder and upper arm
S46.900x002	Injury of shoulder and upper arm tendons
S47.x00	Crushing injury of shoulder and upper arm
S47.x00x002	Crushing injury of shoulder
S47.x00x011	Shoulder thermal crush injury
S47.x00x012	Upper arm thermal crush injury
S47.x01	Crushing injury of upper arm
S48.000	Traumatic amputation at shoulder joint

S48.000x001	Amputation of shoulder joint
S48.100	Traumatic amputation at the level between shoulder and elbow
S48.100x001	Upper arm cutoff
S48.900	Traumatic amputation at shoulder and upper arm level
S49.700	Multiple injuries of shoulder and upper arm
S49.800	Other specific injuries of shoulder and upper arm
S49.900	Shoulder and upper arm injuries
S49.900x001	shoulder injury
S49.901	Upper arm injury
S50.000	Contusion of elbow
S50.100	Contusion of other and unspecified parts of forearm
S50.101	Forearm contusion
S50.700	Multiple superficial injuries of forearm
S50.701	Multiple abrasions of forearm
S50.800	Other superficial injuries of forearm
S50.800x011	Forearm abrasion
S50.800x021	Vesicle of forearm
S50.800x031	Insect bite of forearm
S50.800x041	Forearm superficial foreign body
S50.800x081	Superficial injury of elbow joint
S50.900	Superficial injury of forearm
S50.901	Superficial injury of elbow
S51.000	Open wound of elbow
S51.700	Multiple open wounds on forearm
S51.800	Open wounds in other parts of the forearm

S51.800x011	Open injury of forearm with fracture
S51.800x021	Open injury of forearm with dislocation
S51.900	Open wound of forearm
S51.901	Open forearm injury
S52.000	Fracture of upper end of ulna
S52.000x001	Fracture of elbow joint
S52.000x002	Fracture of proximal ulna
S52.000x011	Fracture of olecranon of ulna
S52.000x012	Separation of olecranon epiphysis of ulna
S52.000x021	Fracture of coronoid process of ulna
S52.000x091	Multiple fractures of proximal ulna
S52.001	Olecranon fracture
S52.002	Monteggia fracture dislocation
S52.010	Open fracture of upper end of ulna
S52.011	Open fracture of olecranon
S52.100	Fracture of upper radius
S52.100x001	Fracture of proximal radius
S52.100x002	Separation of proximal radius epiphysis
S52.100x012	Separation of epiphysis of radial head
S52.100x091	Multiple fractures of proximal radius
S52.101	Fracture of radial head
S52.102	Fracture of radial neck
S52.110	Open fracture of upper radius
S52.200	Fracture of ulnar shaft
S52.200x011	Monteggia fracture
S52.201	Ulnar fracture
S52.210	Open fracture of ulnar shaft

S52.211	Open fracture of ulna
S52.300	Fracture of radial shaft
S52.300x011	Gai's fracture
S52.310	Open fracture of radial shaft
S52.400	Fracture of both ulna and radius diaphysis
S52.400x001	Fracture of shaft of radius and ulna
S52.410	Open fracture of ulna and radius shaft
S52.500	Fracture of lower end of radius
S52.500x001	Fracture of distal radius
S52.500x002	Fracture of styloid process of radius
S52.500x003	Separation of distal radius epiphysis
S52.500x011	Colles Fracture
S52.500x021	Barton fracture
S52.500x022	Smith fracture
S52.500x091	Intraarticular fracture of radius
S52.501	Flexion fracture of distal radius
S52.502	Straight fracture of lower radius
S52.510	Open fracture of distal radius
S52.600	Both ulna and lower radius fractures
S52.600x001	Distal ulna fracture with distal radius fracture
S52.600x002	Ulnar styloid process fracture with distal radius fracture
S52.610	Open fracture of distal radius of ulna
S52.700	Multiple fractures of forearm
S52.701	Closed fracture of radius and ulna
S52.710	Open multiple fractures of forearm
S52.711	Open fracture of radius and ulna
S52.800	Fracture of other parts of forearm

S52.800x002	Separation of distal ulnar epiphysis
S52.801	Fracture of radius
S52.802	Fracture of styloid process of ulna
S52.803	Fracture of ulnar head
S52.804	Fracture of lower end of ulna
S52.810	Open fracture of forearm
S52.811	Open fracture of radius
S52.812	Open fracture of styloid process of ulna
S52.813	Open fracture of ulnar head
S52.814	Open fracture of lower end of ulna
S52.900	Fracture of forearm
S53.000	Dislocation of radial head
S53.000x003	Subluxation of radial head
S53.001	Dislocation of radiohumeral joint
S53.002	Dislocation of radioulnar joint
S53.100	Dislocation of elbow joint
S53.100x011	Anterior dislocation of elbow joint
S53.100x021	Posterior dislocation of elbow joint
S53.100x031	Internal dislocation of elbow joint
S53.100x041	Lateral dislocation of elbow joint
S53.101	Ulnar humeral joint dislocation
S53.102	Dislocation of ulnar head
S53.200	Traumatic rupture of radial collateral ligament
S53.200x001	Rupture of radial collateral ligament
S53.300	Traumatic rupture of ulnar collateral ligament
S53.300x001	Rupture of ulnar collateral ligament
S53.400	Elbow sprain and strain

S53.400x012	Sprain of radial collateral ligament
S53.400x021	Sprain of ulnar collateral ligament
S53.400x031	Sprain of radiohumeral joint
S53.400x041	Ulnar humeral joint sprain
S53.401	Sprain of annular ligament of radius
S53.402	Elbow sprain
S54.000	Ulnar nerve injury at forearm level
S54.000x001	Ulnar nerve injury of forearm
S54.001	Rupture of ulnar nerve of forearm
S54.100	Median nerve injury at forearm level
S54.100x001	Median nerve injury of forearm
S54.101	Fracture of median nerve of forearm
S54.200	Radial nerve injury at forearm level
S54.200x001	Injury of radial nerve of forearm
S54.300	Dermatosensory nerve injury at forearm level
S54.300x001	Injury of cutaneous sensory nerve of forearm
S54.700	Multiple nerve injuries at forearm level
S54.700x001	Multiple nerve injuries of forearm
S54.800	Other nerve injuries at forearm level
S54.900	Nerve injury at forearm level
S54.900x001	Forearm nerve injury
S55.000	Ulnar artery injury at forearm level
S55.000x001	Ulnar artery injury of forearm
S55.100	Radial artery injury at forearm level
S55.100x001	Radial artery injury of forearm
S55.101	Traumatic rupture of radial artery
S55.200	Venous injury at forearm level

S55.200x001	Forearm vein injury
S55.700	Multiple vascular injuries at forearm level
S55.700x001	Multiple vascular injuries of forearm
S55.800	Other vascular injuries at forearm level
S55.900	Injury of blood vessels at forearm level
S55.900x001	Forearm vascular injury
S56.000	Injury of thumb flexor and tendon at forearm level
S56.000x001	Forearm flexor thumb injury
S56.000x002	Injury of flexor tendon of forearm thumb
S56.000x003	Injury of flexor and tendon of forearm thumb
S56.001	Fracture of flexor muscle of forearm thumb
S56.100	Other finger flexor and tendon injuries at forearm level
S56.100x001	Injury of flexor digitorum of forearm
S56.100x002	Injury of flexor tendon of forearm
S56.100x003	Injury of flexor digitorum and tendon of forearm
S56.200	Other flexor and tendon injuries at forearm level
S56.200x001	Forearm flexor injury
S56.200x002	Injury of flexor tendon of forearm
S56.200x003	Injury of flexor and tendon of forearm
S56.300	Injury of extensor or abductor thumb muscle and tendon at forearm level
S56.300x001	Injury of extensor muscle of forearm thumb
S56.300x002	Injury of extensor tendon of forearm thumb
S56.300x003	Injury of extensor muscle and tendon of forearm thumb
S56.300x004	Injury of abductor thumb muscle of forearm
S56.300x005	Forearm abductor tendon injury
S56.300x006	Injury of abductor and tendon of forearm thumb
S56.301	Fracture of abductor muscle and tendon of forearm thumb
S56.400	Other finger extensor and tendon injuries at forearm level
S56.400x001	Injury of extensor digitorum of forearm
S56.400x002	Injury of extensor tendon of forearm finger
S56.400x003	Injury of extensor muscle and tendon of forearm finger
S56.500	Other extensor and tendon injuries at forearm level
S56.500x001	Forearm extensor injury
S56.500x002	Injury of extensor tendon of forearm
S56.500x003	Injury of forearm extensor and tendon
S56.700	Multiple muscle and tendon injuries at forearm level
S56.700x001	Multiple muscle and tendon injuries of forearm
S56.800	Injuries to other muscles and tendons at the forearm level
S56.801	Injury of forearm muscles and tendons
S57.000	Elbow crush injury
S57.000x001	Crushing injury of elbow
S57.000x011	Elbow thermal crush injury
S57.800	Crushing injury of other parts of forearm
S57.900	Crushing injury of forearm
S57.900x001	Crushing injury of forearm
S57.900x011	Thermal crush injury of forearm
S58.000	Traumatic amputation at elbow level

S58.000x001	Traumatic amputation of elbow
S58.100	Traumatic amputation at the level between elbow and wrist
S58.100x001	Horizontal traumatic amputation between elbow and wrist
S58.900	Traumatic amputation at forearm level
S58.900x001	Traumatic amputation of forearm
S59.700	Multiple injuries of forearm
S59.800	Other specific injuries of forearm
S59.900	forearm injuries
S60.000	Finger contusion without nail damage
S60.000x001	Finger contusion
S60.100	Finger contusion with nail damage
S60.200	Contusion of other parts of wrist and hand
S60.201	Wrist contusion
S60.202	Hand contusion
S60.700	Multiple superficial injuries of wrist and hand
S60.701	Multiple superficial injuries of hands
S60.800	Other superficial injuries of wrist and hand
S60.800x011	Wrist and hand abrasions
S60.800x012	Wrist scratch
S60.800x021	Wrist and hand blisters
S60.800x022	Hand blisters
S60.800x023	Wrist blisters
S60.800x031	Wrist and hand insect bites
S60.800x032	Wrist insect bite
S60.800x033	Insect bites on hands

S60.800x041	Superficial foreign body of wrist and hand
S60.800x042	Foreign body on the surface of wrist
S60.800x043	Foreign body on the surface of hand
S60.801	Foreign body on the surface of finger
S60.900	Superficial injury of wrist and hand
S60.900x001	Superficial injury of wrist and hand
S60.900x002	Superficial wrist injury
S60.901	Superficial hand injury
S60.902	Hand scratch
S61.000	Open wound of finger without nail damage
S61.000x001	Open injury of finger
S61.000x002	Open injury of thumb
S61.100	Open wound of finger with nail damage
S61.100x002	Open injury of thumb with nail injury
S61.700	Multiple open wounds on wrists and hands
S61.701	Open multiple wrist injuries
S61.702	Open multiple hand injuries
S61.800	Open wounds on the wrist and other parts of the hand
S61.800x011	Open injury of wrist and hand with fracture
S61.800x012	Open injury of hand with fracture
S61.800x013	Open injury of wrist with fracture
S61.800x021	Open injury of wrist and hand with dislocation
S61.800x022	Open hand injury with dislocation
S61.800x023	Open injury of wrist with dislocation
S61.800x081	Open injury of palm

S61.900	Open wound of wrist and hand
S61.900x002	Open injury of wrist
S61.900x004	Hand explosion injury
S61.900x005	Hand degloving injury
S61.901	Open hand injury
S61.902	Teared gloves
S62.000	Fracture of scaphoid bone
S62.000x001	Fracture of scaphoid bone of wrist
S62.010	Open fracture of scaphoid bone of hand
S62.100	Carpal fracture, other special
S62.100x011	Lunate fracture
S62.100x021	Triangular fracture
S62.100x031	Fracture of bean bone
S62.100x041	Most angular bone fracture
S62.100x051	Small polygonal bone fracture
S62.100x061	Capitate fracture
S62.100x071	Hamate fracture
S62.100x091	Multiple fracture of carpus
S62.101	Fracture of carpal bone
S62.110	Open fracture of special finger carpus
S62.111	Open carpal fracture
S62.200	Fracture of the first metacarpal bone
S62.200x011	Fracture of the first metacarpal base
S62.200x021	Fracture of the first metacarpal shaft
S62.200x031	Fracture of the first metacarpal neck
S62.200x041	Fracture of the first metacarpal head
S62.201	Bennett fracture
S62.210	Open fracture of the first metacarpal bone
S62.300	Metacarpal fracture, others
S62.300x002	Metacarpal epiphysis separation
S62.300x011	Metacarpal basal fracture
S62.300x021	Metacarpal shaft fracture
S62.300x031	Fracture of metacarpal neck
S62.300x041	Fracture of metacarpal head
S62.301	Metacarpal fracture
S62.310	Open fracture of special metacarpal bone
S62.311	Open metacarpal fracture
S62.400	Multiple metacarpal fractures
S62.410	Open multiple metacarpal fractures
S62.500	Thumb fracture
S62.500x002	Separation of thumb epiphysis
S62.500x011	Proximal segment of thumb fracture
S62.500x021	Fracture of distal segment of thumb
S62.510	Open thumb fracture
S62.600	Finger fracture, others
S62.600x002	Separation of phalangeal epiphysis
S62.600x011	Fracture of proximal phalanx
S62.600x021	Fracture of middle phalanx
S62.600x031	Fracture of distal phalanx
S62.610	Open fracture of special finger bone
S62.611	Open phalangeal fracture
S62.700	Multiple fractures of fingers
S62.710	Open multiple phalangeal fractures
S62.800	Fractures of other and unspecified parts of wrist and hand
S62.801	Hand fracture
S62.802	Phalangeal fracture

S62.810	Open fracture of other and unspecified parts of wrist and hand
S62.811	Open hand fracture
S63.000	Dislocation of wrist joint
S63.000x002	Dislocation of distal radius joint
S63.000x003	Distal joint dislocation of ulna
S63.000x011	Dislocation of lower radioulnar joint
S63.000x021	Dislocation of radiocarpal joint
S63.000x031	Dislocation of carpal joint
S63.000x041	Dislocation of proximal metacarpal joint
S63.000x042	Dislocation of carpometacarpal joint
S63.000x081	Dislocation of carpal bone
S63.000x082	Dislocation of scaphoid bone of wrist
S63.000x083	Perilunate dislocation of scaphoid bone of wrist
S63.100	Dislocation of finger joint
S63.100x001	Dislocation of interphalangeal joint
S63.100x002	Dislocation of thumb joint
S63.100x011	Metacarpophalangeal joint dislocation
S63.100x012	Dislocation of distal metacarpal joint
S63.100x013	Dislocation of thumb metacarpal joint
S63.100x021	Dislocation of distal interphalangeal joint of finger
S63.200	Multiple dislocations of fingers
S63.200x001	Multiple dislocation of finger joint
S63.300	Traumatic rupture of wrist and wrist ligament
S63.300x001	Fracture of carpal collateral ligament
S63.300x002	Rupture of radiocarpal ligament
S63.300x003	Rupture of ulnar carpal ligament

S63.300x004	Fracture of wrist and wrist ligament
S63.400	Traumatic rupture of finger ligaments at metacarpophalangeal and interphalangeal joints
S63.400x001	Fracture of metacarpophalangeal joint ligament
S63.400x002	Fracture of collateral ligament of metacarpophalangeal joint
S63.400x003	Rupture of palmar ligament
S63.400x004	Fracture of palm plate
S63.400x005	Rupture of ligament of interphalangeal joint
S63.400x006	Rupture of collateral ligament of interphalangeal joint
S63.401	Traumatic rupture of metacarpal ligament
S63.500	Wrist sprain and strain
S63.500x002	Wrist joint injury
S63.500x011	Wrain of wrist joint
S63.500x012	Injury of carpal joint
S63.500x021	Sprain of radiocarpal joint
S63.500x022	Injury of radiocarpal joint
S63.500x031	Wrist palm joint sprain
S63.500x032	Injury of carpometacarpal joint
S63.500x081	Sprain of lower radioulnar joint
S63.500x082	Lower radioulnar joint injury
S63.500x101	Injury of triangular fibrocartilage of wrist joint
S63.501	Wrist sprain
S63.600	Finger sprain and strain
S63.600x001	Sprain of thumb
S63.600x002	Sprain of phalange
S63.601	Finger joint sprain

S63.602	Sprain of metacarpophalangeal joint
S63.700	Sprain and strain of other and unspecified parts of hands
S63.700x001	Sprain of middle wrist joint
S63.701	Hand joint sprain
S64.000	Ulnar nerve injury at wrist and hand level
S64.000x001	Ulnar nerve injury of wrist
S64.000x002	Ulnar nerve injury of hand
S64.100	Median nerve injury at wrist and hand level
S64.100x001	Median nerve injury of wrist
S64.100x002	Median nerve injury of hand
S64.200	Radial nerve injury at wrist and hand level
S64.200x001	Wrist radial nerve injury
S64.200x002	Hand radial nerve injury
S64.300	Injury of thumb and finger nerve
S64.400	Finger nerve injury, others
S64.400x001	Finger nerve injury
S64.700	Multiple nerve injuries at the wrist and hand level
S64.700x001	Multiple nerve injuries of wrist and hand
S64.800	Other nerve injuries at the wrist and hand level
S64.900	Nerve injuries at the wrist and hand levels
S64.900x001	Nerve injury of wrist and hand
S65.000	Ulnar artery injury at wrist and hand level
S65.000x001	Ulnar artery injury of hand
S65.000x002	Ulnar artery injury of wrist

S65.100	Radial artery injury at wrist and hand level
S65.100x001	Wrist radial artery injury
S65.100x002	Hand radial artery injury
S65.200	Injury of superficial palmar arteriovenous arch
S65.300	Injury of deep palmar arteriovenous arch
S65.400	Blood vessel injury of thumb
S65.401	Traumatic rupture of thumb artery
S65.500	Finger vascular injury, others
S65.501	Traumatic digital artery rupture
S65.700	Multivascular injuries at the wrist and hand levels
S65.700x001	Multiple vascular injuries of wrist and hand
S65.800	Other vascular injuries at the wrist and hand levels
S65.900	Injury of blood vessels at the wrist and hand level
S65.900x001	Injury of wrist and hand vessels
S66.000	Injury of flexor longus thumb and tendons at wrist and hand levels
S66.000x001	Injury of flexor longus muscle and tendon of wrist and hand
S66.000x002	Injury of flexor longus muscle of wrist and hand
S66.000x003	Injury of flexor longus tendon of wrist and hand
S66.000x004	Injury of flexor pollicis longus and tendon of wrist
S66.000x005	Injury of flexor thumb longus muscle of wrist
S66.000x006	Injury of flexor digitorum longus tendon of wrist
S66.000x007	Injury of flexor longus thumb and tendon of hand

S66.000x008	Injury of flexor longus thumb of hand
S66.000x009	Injury of flexor longus tendon of hand
S66.100	Other finger flexor and tendon injuries at wrist and hand levels
S66.100x001	Wrist and finger flexor and tendon injuries
S66.100x002	Wrist and finger flexor injuries
S66.100x003	Injury of flexor tendon of wrist and finger
S66.100x004	Injury of flexor and tendon of wrist
S66.100x005	Injury of flexor digitorum muscle of wrist
S66.100x006	Injury of flexor tendon of wrist
S66.100x007	Finger flexor and tendon injuries
S66.100x008	Finger flexor injury
S66.100x009	Injury of flexor tendon of hand
S66.200	Injury of extensor and tendon of thumb at wrist and hand level
S66.200x001	Injury of extensor muscle and tendon of wrist and hand
S66.200x002	Injury of extensor muscle of wrist and hand
S66.200x003	Injury of extensor tendon of wrist and hand
S66.200x004	Injury of extensor muscle and tendon of wrist
S66.200x005	Injury of extensor muscle of wrist
S66.200x006	Injury of extensor tendon of wrist
S66.200x007	Injury of extensor muscle and tendon of hand
S66.200x008	Injury of extensor muscle of hand
S66.200x009	Injury of extensor tendon of hand
S66.300	Other finger extensor and tendon injuries at the wrist and hand levels

S66.300x001	Injury of wrist and finger extensors and tendons
S66.300x002	Injury of wrist and finger extensors
S66.300x003	Injury of extensor tendon of wrist and finger
S66.300x004	Injury of extensor digitorum and tendon of wrist
S66.300x005	Injury of extensor digitorum muscle of wrist
S66.300x006	Injury of extensor digitorum tendon of wrist
S66.300x007	Injury of extensor digitorum and tendon of hand
S66.300x008	Hand extensor injury
S66.300x009	Hand extensor tendon injury
S66.400	Injury of internal thumb muscles and tendons at wrist and hand levels
S66.400x001	Injury of internal muscles and tendons of wrist and thumb
S66.400x002	Injury of internal muscles of wrist and thumb
S66.400x003	Internal tendon injury of wrist and thumb
S66.400x004	Injury of internal muscle and tendon of thumb at wrist
S66.400x005	Injury of internal thumb muscle of wrist
S66.400x006	Injury of internal tendon of thumb at wrist
S66.400x007	Injuries of internal thumb muscles and tendons
S66.400x008	Injury of internal thumb muscle of hand
S66.400x009	Injury of internal tendon of thumb
S66.500	Injuries to internal muscles and tendons of other fingers at the wrist and hand levels

S66.500x001	Injury of internal muscles and tendons of wrist and fingers
S66.500x002	Injury of intrinsic muscles of wrist and finger
S66.500x003	Internal tendon injury of wrist and finger
S66.500x004	Injury of internal muscle and tendon of wrist
S66.500x005	Injury of internal muscle of wrist finger
S66.500x006	Injury of internal tendon of wrist finger
S66.500x007	Injury of internal muscles and tendons of the hand
S66.500x008	Injury of internal muscle of hand
S66.500x009	Injury of internal tendon of hand
S66.600	Multiple flexor and tendon injuries at wrist and hand levels
S66.600x001	Multiple flexor and tendon injuries of wrist and hand
S66.601	Multiple rupture of flexor muscle of hand
S66.700	Multiple extensor and tendon injuries at the wrist and hand levels
S66.700x001	Multiple extensor and tendon injuries of wrist and hand
S66.800	Injuries to other muscles and tendons at the wrist and hand levels
S66.900	Muscle and tendon injuries at the wrist and hand levels
S66.900x001	Wrist and hand muscle and tendon injuries
S66.900x002	Wrist muscle injury
S66.900x003	Hand muscle injury
S66.900x004	Finger muscle injury
S67.000	Crushing injury of thumb and other fingers
S67.000x001	Crushing injury of thumb

S67.000x003	Crushing and contusion of fingers
S67.000x011	Thumb thermal crush injury
S67.000x012	Finger thermal crush injury
S67.001	Finger crush injury
S67.800	Crushing injury of wrist and hand in other and unspecified parts
S67.800x001	Wrist crush injury
S67.800x003	Crushing and contusion of hand
S67.800x011	Hand heat crush injury
S67.801	Hand crush injury
S68.000	Traumatic amputation of thumb (complete) (partial)
S68.000x002	Incomplete amputation of thumb
S68.001	Total amputation of thumb
S68.100	Traumatic amputation of single finger (complete) (partial), others
S68.100x001	Single finger incomplete cutoff
S68.100x002	Single finger complete disconnection
S68.200	Traumatic amputation (complete) (partial) of only two or more fingers
S68.200x001	Multi finger incomplete cutting
S68.201	Multiple fingers completely cut off
S68.300	Combined traumatic amputation of finger (part) with wrist and other parts of hand
S68.400	Traumatic amputation of hand at wrist level
S68.400x001	Traumatic amputation of wrist
S68.800	Traumatic amputation of wrist and other parts of hand
S68.800x001	Traumatic amputation of palm
S68.900	Traumatic amputation at wrist and hand level
S69.700	Multiple injuries of wrist and hand

S69.800	Other specific injuries to wrists and hands
S69.900	Wrist and hand injuries
S69.900x001	Wrist injury
S69.900x002	Hand injury
S69.900x003	Thumb injury
S69.900x004	Finger injury
S70.000	Hip contusion
S70.000x001	Hip contusion
S70.100	Thigh contusion
S70.700	Multiple superficial injuries of hip and thigh
S70.700x001	Multiple superficial injuries of thigh
S70.700x002	Multiple superficial injuries of hip
S70.800	Other superficial injuries of hip and thigh
S70.800x011	Hip Scratch
S70.800x012	Scratch of thigh
S70.800x021	Hip blisters
S70.800x022	Femoral vesicles
S70.800x031	Hip worm bite
S70.800x032	Femoral insect bite
S70.800x041	Superficial foreign body of hip
S70.800x042	Foreign body on the surface of thigh
S70.900	Superficial injury of hip and thigh
S70.900x001	Superficial injury of hip
S70.900x002	Superficial injury of thigh
S70.900x003	Superficial injury of thigh
S70.901	Thigh hematoma
S71.000	Open wound of hip

S71.000x001	Open injury of hip
S71.100	Open wound of thigh
S71.101	Avulsion injury of thigh
S71.700	Multiple open wounds on hip and thigh
S71.800	Open wounds in other and unspecified parts of the pelvic band
S71.800x011	Open injury of hip with fracture
S71.800x012	Open injury of thigh with fracture
S71.800x021	Open injury of hip with dislocation
S71.800x022	Open injury of thigh with dislocation
S71.801	Open pelvic band injury
S72.000	Femoral neck fracture
S72.000x011	Intracystic fracture of femoral joint
S72.000x021	Separation of femoral head epiphysis
S72.000x031	Subcapitular fracture of femoral neck
S72.000x041	Transcervical fracture of femoral neck
S72.000x051	Femoral neck basal fracture
S72.000x081	Femoral head fracture
S72.000x082	Femoral hip fracture
S72.010	Open femoral neck fracture
S72.100	Transtrochanteric fracture
S72.100x001	Fracture of greater trochanter of femur
S72.100x002	Fracture of lesser trochanter of femur
S72.100x011	Intertrochanteric fracture of femur
S72.101	Intertrochanteric fracture of femur
S72.110	Open intertrochanteric fracture of femur
S72.200	Subtrochanteric fracture
S72.200x001	Subtrochanteric fracture of femur

S72.210	Open subtrochanteric fracture of femur
S72.300	Femoral shaft fracture
S72.310	Open fracture of femoral shaft
S72.400	Lower femur fracture
S72.400x001	Distal femoral fracture
S72.400x012	Fracture of internal femoral condyle
S72.400x013	Fracture of external femoral condyle
S72.400x021	Separation of distal femoral epiphysis
S72.400x031	Supracondylar fracture of femur
S72.400x041	Femoral intercondylar fracture
S72.401	Fracture of femoral condyle
S72.410	Open fracture of distal femur
S72.700	Multiple fractures of femur
S72.710	Open multiple femoral fractures
S72.800	Fracture of other parts of femur
S72.810	Open fracture of femur at specific site
S72.900	Femoral fracture
S72.900x002	Separation of femoral epiphysis
S72.910	Open femoral fracture
S73.000	ddh
S73.000x002	Dislocation of hip joint
S73.000x003	Acetabular dislocation
S73.000x011	Posterior dislocation of hip joint
S73.000x021	Anterior dislocation of hip joint
S73.001	Subluxation of hip joint
S73.100	Hip sprain and strain
S73.100x001	Sprain of hip joint
S73.100x011	Sprain of iliofemoral ligament
S73.100x021	Sprain of ligament of iliac joint capsule

S73.101	Hip sprain
S74.000	Sciatic nerve injury at hip and thigh level
S74.000x001	Injury of sciatic nerve
S74.000x002	Hip sciatic nerve injury
S74.000x003	Injury of sciatic nerve of thigh
S74.100	Femoral nerve injury at hip and thigh level
S74.100x001	Femoral nerve injury
S74.100x002	Hip femoral nerve injury
S74.100x003	Femoral nerve injury of thigh
S74.200	Dermatosensory nerve injury at hip and thigh level
S74.200x001	Hip cutaneous sensory nerve injury
S74.200x002	Injury of sensory nerve of thigh skin
S74.700	Multiple nerve injuries at hip and thigh levels
S74.700x001	Multiple nerve injuries at hip
S74.700x002	Multiple nerve injuries of thigh
S74.800	Other nerve injuries at hip and thigh levels
S74.801	Obturator nerve injury
S74.900	Nerve injuries at hip and thigh levels
S74.900x001	Hip nerve injury
S74.900x002	Nerve injury of thigh
S75.000	Femoral artery injury
S75.000x002	Injury of superficial femoral artery
S75.000x003	Deep femoral artery injury
S75.000x004	Traumatic femoral aneurysm
S75.000x005	Traumatic femoral pseudoaneurysm
S75.001	Traumatic rupture of deep femoral artery

S75.100	Femoral vein injury at hip and thigh level
S75.100x001	Femoral vein injury
S75.100x002	Femoral vein injury of hip
S75.100x003	Femoral vein injury of thigh
S75.200	Injury of great saphenous vein at hip and thigh level
S75.200x001	Injury of great saphenous vein of thigh
S75.700	Multivascular injuries at hip and thigh levels
S75.700x001	Multiple vascular injuries in hip
S75.700x002	Multiple vascular injuries of thigh
S75.800	Other vascular injuries at hip and thigh levels
S75.900	Injury of blood vessels at hip and thigh levels
S75.900x001	Hip vascular injury
S75.900x002	Injury of thigh blood vessels
S75.901	Traumatic femoral arteriovenous fistula
S76.000	Hip muscle and tendon injuries
S76.000x002	Hip muscle injury
S76.000x003	Hip tendon injury
S76.100	Injury of quadriceps and tendon
S76.100x001	Injury of quadriceps femoris and tendon
S76.100x002	Muscle injury of quadriceps femoris
S76.100x003	Tendon injury of quadriceps femoris
S76.101	Tendon rupture of quadriceps femoris
S76.102	Injury of patellar ligament
S76.200	Injury of adductor muscle and tendon of thigh
S76.200x002	Muscle injury of adductor muscle of thigh
S76.200x003	Injury of adductor tendon of thigh
S76.300	Injury of posterior muscle groups and tendons at thigh level
S76.300x001	Injury of posterior thigh muscle group and tendon
S76.300x002	Muscle injury of posterior thigh muscles
S76.300x003	Tendon injury of posterior thigh muscles
S76.301	Injury of tendon at the back of thigh
S76.400	Other and unspecified muscle and tendon injuries at thigh level
S76.401	Injury of thigh tendon
S76.402	Rupture of thigh muscle
S76.700	Multiple muscle and tendon injuries at the hip joint and thigh level
S76.700x001	Multiple muscle and tendon injuries in the hip and thigh
S77.000	Hip crush injury
S77.000x011	Hip thermal crush injury
S77.100	Crushing injury of thigh
S77.100x001	Hot crush injury of thigh
S77.200	Hip with thigh crush injury
S77.200x001	Crushing injury of hip and thigh
S77.200x011	Hip with hot crush injury of thigh
S78.000	Traumatic amputation of hip
S78.000x001	Hip amputation
S78.100	Traumatic amputation at the level between hip and knee
S78.100x001	Thigh amputation
S78.900	Traumatic amputation at hip and thigh level
S79.700	Multiple injuries of hip and thigh

S79.701	Multiple thigh injuries
S79.800	Other specific injuries of hip and thigh
S79.800x001	Soft tissue injury around hip joint
S79.900	Hip and thigh injuries
S79.900x001	Hip injury
S79.901	Thigh injury
S79.902	Peri hip soft tissue injury
S80.000	Contusion of knee
S80.000x001	Knee contusion
S80.100	Contusion of other and unspecified parts of the lower leg
S80.100x002	Hematoma of lower leg
S80.101	Crural contusion
S80.700	Multiple superficial injuries of lower leg
S80.800	Other superficial injuries of the lower leg
S80.800x011	Crural bruising
S80.800x012	Knee bruising
S80.800x013	Abrasion of popliteal fossa
S80.800x021	Vesicle of calf
S80.800x022	Knee blister
S80.800x023	Popliteal vesicle
S80.800x031	Calf insect bite
S80.800x032	Knee insect bite
S80.800x033	Popliteus bite
S80.800x041	Foreign body on the surface of lower leg
S80.800x042	Superficial foreign body of knee
S80.800x043	Superficial foreign body in popliteal fossa
S80.900	Superficial injury of lower leg

S80.901	Hematoma of knee
S81.000	Open wound of knee
S81.700	Multiple open wounds on the lower leg
S81.800	Open wounds in other parts of the lower leg
S81.800x011	Open injury of lower leg with fracture
S81.800x021	Open injury of lower leg with dislocation
S81.800x081	Open injury of fibula
S81.800x082	Open injury of popliteal fossa
S81.800x083	Open injury of tibia
S81.900	Open wound of lower leg
S81.901	Avulsion injury of lower leg
S82.000	Patella fracture
S82.000x002	Fracture of patella cartilage
S82.000x003	Sleeve avulsion fracture of patella
S82.000x004	Sleeve fracture of patella
S82.010	Open patellar fracture
S82.100	Fracture of upper tibia
S82.100x011	Proximal tibia fracture with fibula fracture
S82.100x012	Tibial plateau with fibular fracture
S82.100x081	proximal tibia fracture
S82.100x082	Separation of proximal tibial epiphysis
S82.100x084	Fracture of tibial condyle
S82.100x085	Fracture of intercondylar spine of tibia
S82.100x086	Fracture of lateral condyle of tibia
S82.100x087	Tibial plateau fracture
S82.100x088	Tibial plateau with intercondylar fracture
S82.100x089	Tibial tubercle fracture

S82.101	Closed fracture of tibial plateau
S82.102	Tibial head fracture
S82.110	Open fracture of upper tibia
S82.111	Open fracture of tibial head
S82.200	Tibial shaft fracture
S82.200x011	Tibial shaft fracture with fibular fracture
S82.200x081	Tibial shaft fracture
S82.201	Fracture of tibiofibular shaft
S82.202	Tibial fracture
S82.203	Closed fracture of tibia and fibula
S82.210	Open fracture of tibial shaft
S82.211	Open tibia fracture
S82.212	Open tibiofibular shaft fracture
S82.300	Fracture of lower tibia
S82.300x011	Distal tibia fracture with fibula fracture
S82.300x012	Separation of lower tibiofibular epiphysis
S82.300x081	Fracture of distal tibia
S82.300x082	Separation of distal tibial epiphysis
S82.300x083	Pilon fracture
S82.301	Fracture of lower end of tibia and fibula
S82.310	Open fracture of lower tibia
S82.311	Open fracture of distal tibia and fibula
S82.400	Fibula fracture only
S82.400x001	Fibular fracture
S82.400x002	Separation of distal fibular epiphysis
S82.400x011	Fracture of proximal fibula
S82.400x012	Fracture of fibular head
S82.400x013	Fracture of fibular neck
S82.400x014	Fracture of fibular capitulum
S82.400x091	Multiple fracture of fibula
S82.401	Fracture of fibular shaft
S82.410	Open fibular fracture
S82.411	Open fracture of fibular shaft
S82.500	Medial malleolus fracture
S82.501	Tibial fracture involving ankle joint
S82.510	Open fracture of medial malleolus
S82.600	Fracture of lateral malleolus
S82.601	Fracture of fibula involving ankle joint
S82.610	Open fracture of lateral malleolus
S82.700	Multiple fractures of lower leg
S82.710	Open multiple fractures of lower leg
S82.800	Fracture of other parts of the lower leg
S82.800x081	Ankle fracture
S82.800x082	ankle fracture
S82.801	cotton fracture
S82.802	Bilateral ankle fracture
S82.803	Closed fracture of ankle
S82.810	Open fracture of specific part of lower leg
S82.811	Open trimalleolar fracture
S82.812	Open fracture of both ankles
S82.900	Crural fracture
S82.910	Open fracture of lower leg
S83.000	Dislocation of patella
S83.001	Subluxation of patella
S83.100	Dislocation of knee joint

S83.100x011	Anterior dislocation of proximal tibia
S83.100x012	Posterior dislocation of distal femur
S83.100x021	Posterior dislocation of proximal tibia
S83.100x031	Medial dislocation of proximal tibia
S83.100x041	Lateral dislocation of proximal tibia
S83.100x081	Dislocation of tibiofibular joint
S83.101	Subluxation of knee joint
S83.102	Proximal dislocation of tibiofibular joint
S83.200	Meniscus tear, recent
S83.200x001	Tear of meniscus of knee
S83.200x002	Barrel handle tear of lateral meniscus of knee
S83.200x003	Barrel handle tear of medial meniscus of knee
S83.200x004	Barrel handle tear of meniscus of knee
S83.200x005	Tear of medial meniscus of knee
S83.200x006	Tear of lateral meniscus of knee
S83.201	Medial meniscus injury of knee
S83.202	Injury of lateral meniscus of knee
S83.300	Tears of knee cartilage, recent
S83.300x001	Tear of cartilage of knee joint
S83.400	Sprain and strain involving the knee joint (fibula) (tibia) collateral ligament
S83.400x001	Sprain of collateral ligament of knee joint
S83.400x002	Rupture of collateral ligament of knee joint
S83.400x003	Injury of collateral ligament of knee joint
S83.400x011	Sprain of lateral collateral ligament of knee joint
S83.400x012	Injury of lateral collateral ligament of knee joint
S83.400x021	Sprain of medial collateral ligament of knee joint
S83.400x022	Injury of medial collateral ligament of knee joint
S83.400x031	Partial rupture of lateral collateral ligament of knee joint
S83.400x032	Complete rupture of lateral collateral ligament of knee joint
S83.400x041	Partial rupture of medial collateral ligament of knee joint
S83.400x042	Complete rupture of medial collateral ligament of knee joint
S83.401	Strain of collateral ligament of knee joint
S83.500	Sprain and strain involving the (anterior) (posterior) cruciate ligament of the knee
S83.500x001	Rupture of cruciate ligament of knee joint
S83.500x002	injuries of crucial ligaments
S83.500x003	Injury of cruciate ligament of knee joint
S83.500x011	Sprain of anterior cruciate ligament of knee joint
S83.500x012	Injury of anterior cruciate ligament of knee joint
S83.500x021	Sprain of posterior cruciate ligament of knee joint
S83.500x022	Injury of posterior cruciate ligament of knee joint
S83.500x031	Partial rupture of anterior cruciate ligament of knee joint
S83.500x032	Complete rupture of anterior cruciate ligament of knee joint
S83.500x041	Partial rupture of posterior cruciate ligament of knee joint
S83.500x042	Complete rupture of posterior cruciate ligament of knee joint
S83.501	Strain of cruciate ligament of knee joint

S83.600	Sprain and strain of other and unspecified parts of the knee
S83.600x002	Knee joint injury
S83.600x004	Sprain of proximal tibiofibular joint
S83.600x005	Injury of proximal tibiofibular joint
S83.600x006	Sprain of proximal tibiofibular ligament
S83.600x007	Injury of proximal tibiofibular ligament
S83.600x009	Rupture of patellar tendon
S83.601	Sprain of knee joint
S83.602	Tear of upper end of tibiofibular ligament
S83.603	Sprain of upper tibiofibular joint
S83.700	Injuries to multiple structures of the knee
S83.700x001	Lateral meniscus of knee with collateral ligament injury
S83.700x002	Lateral meniscus of knee with cruciate ligament injury
S83.700x003	Multiple injuries of knee joint
S83.700x004	Medial meniscus of knee with collateral ligament injury
S83.700x005	Medial meniscus of knee with cruciate ligament injury
S83.700x006	Multiple ligament injuries of knee joint
S84.000	Tibial nerve injury at the lower leg level
S84.000x001	Injury of posterior tibial nerve
S84.000x002	Tibial nerve injury
S84.100	Peroneal nerve injury at the lower leg level
S84.100x001	Peroneal nerve injury
S84.200	Dermatosensory nerve injury at the lower leg level

S84.200x001	Sensory nerve injury of lower leg skin
S84.700	Multiple nerve injuries at the lower leg level
S84.700x001	Multiple nerve injuries in the lower leg
S84.800	Other nerve injuries at the lower leg level
S84.800x001	Common peroneal nerve injury
S84.800x002	Sural nerve injury
S84.900	Nerve injury at the lower leg level
S84.900x001	Nerve injury of lower leg
S85.000	Popliteal artery injury
S85.100	Tibial artery injury (anterior) (posterior)
S85.100x002	Injury of anterior tibial artery
S85.101	Injury of posterior tibial artery
S85.102	Traumatic posterior tibial artery thrombosis
S85.200	Peroneal artery injury
S85.300	Injury of great saphenous vein at lower leg level
S85.300x001	Injury of great saphenous vein of lower leg
S85.400	Injury of small saphenous vein at lower leg level
S85.400x001	Injury of saphenous vein of lower leg
S85.500	Popliteal vein injury
S85.700	Multiple vascular injuries at the lower leg level
S85.700x001	Multiple vascular injuries in the lower leg
S85.800	Other vascular injuries at the lower leg level
S85.800x001	Injury of posterior tibial vessels

S85.801	Traumatic posterior tibial artery and vein injury
S85.900	Injury of blood vessels at the lower leg level
S85.900x001	Crural vascular injury
S86.000	Achilles tendon injury
S86.001	Achilles tendon rupture
S86.100	Other muscle and tendon injuries in the posterior muscle group at the calf level
S86.100x001	Injury of posterior calf muscle group and tendon
S86.100x002	Muscle injury of posterior calf muscles
S86.100x003	Tendon injury of posterior calf muscles
S86.200	Muscle and tendon injuries in the anterior muscle group at the calf level
S86.200x002	Tendon injury of anterior calf muscles
S86.201	Tendon injury of anterior horizontal muscle group of lower leg
S86.300	Muscle and tendon injuries in the peroneal muscle group at the calf level
S86.300x001	Injury of peroneal muscle group and tendon
S86.300x002	Injury of peroneal muscles
S86.300x003	Tendon injury of peroneal muscles
S86.300x004	Gastrocnemius rupture
S86.300x005	Injury of peroneal long and short muscles
S86.300x006	Traumatic peroneal tendon slippage
S86.301	Tendon injury of horizontal peroneal muscle group of lower leg
S86.700	Multiple muscle and tendon injuries at the calf level
S86.700x001	Multiple muscle and tendon injuries in the lower leg
S86.700x002	Tibiofibular tendon rupture

S86.701	Multiple tendon injuries at the lower leg level
S86.800	Other muscle and tendon injuries at the calf level
S86.900	Muscle and tendon injuries at the calf level
S86.901	Crural horizontal muscle injury
S87.000	Knee crush injury
S87.800	Crushing injury of other and unspecified parts of lower leg
S87.800x001	Crural thermal crush injury
S87.801	Crural crush injury
S88.000	Traumatic amputation at knee level
S88.000x001	Knee amputation
S88.100	Traumatic amputation at the level between knee and ankle
S88.100x001	Calf amputation
S88.900	Traumatic amputation at the lower leg level
S89.700	Multiple injuries of lower leg
S89.800	Other specific injuries of the lower leg
S89.900	Lower leg injury
S90.000	Ankle contusion
S90.100	Toe contusion without toenail damage
S90.200	Toe contusion with toenail damage
S90.300	Contusion of other and unspecified parts of the foot
S90.300x001	Injury of accessory navicular bone
S90.300x002	Osteochondral injury of talus
S90.300x003	Injury of posterior trigone of talus
S90.301	Foot contusion
S90.700	Multiple superficial injuries of ankle and foot

S90.800	Other superficial injuries of ankle and foot
S90.800x011	Ankle and foot abrasions
S90.800x012	Ankle scratch
S90.800x013	Foot abrasions
S90.800x021	Ankle and foot blisters
S90.800x022	Ankle blister
S90.800x023	Foot blister
S90.800x031	Ankle and foot insect bites
S90.800x032	Ankle insect bite
S90.800x033	Foot insect bite
S90.800x041	Superficial foreign body of ankle and foot
S90.800x042	Superficial foreign body of ankle
S90.800x043	Foreign body on the surface of foot
S90.900	Superficial injury of ankle and foot
S90.900x001	Superficial injury of ankle and foot
S90.900x002	Superficial ankle injury
S90.900x003	Superficial injury of foot
S90.901	Haematoma of toenail
S91.000	Open wound of ankle
S91.100	Open wound of toe without toenail damage
S91.200	Open wound of toe with nail damage
S91.300	Open wounds in other parts of the foot
S91.300x002	Foot degloving injury
S91.300x003	Open injury of heel
S91.300x811	Open injury of ankle and foot with fracture
S91.300x812	Open injury of ankle with fracture

S91.300x813	Open injury of foot with fracture
S91.300x821	Open injury of ankle and foot with dislocation
S91.300x822	Open injury of ankle with dislocation
S91.300x823	Open injury of foot with dislocation
S91.301	Open foot injury
S91.302	Skin laceration of foot
S91.303	Foot laceration
S91.700	Multiple open wounds on ankle and foot
S91.700x002	Multiple open injuries of ankle
S91.700x003	Multiple open injuries of foot
S92.000	Calcaneal fracture
S92.010	Open calcaneal fracture
S92.100	Talus fracture
S92.100x003	Fracture of talus body
S92.101	Talar neck fracture
S92.110	Open talus fracture
S92.200	Fracture of tarsal bone, others
S92.200x001	Tarsal fracture
S92.200x011	Scaphoid fracture
S92.200x081	Fracture of tarsal joint
S92.201	Fracture of cuboid bone
S92.202	Fracture of scaphoid bone of foot
S92.203	Wedge bone fracture (foot)
S92.210	Open fracture of tarsal bone of special finger
S92.300	Metatarsal fracture
S92.300x001	Fracture of tarsometatarsal joint
S92.300x003	Metatarsal basal fracture

S92.300x004	Metatarsal epiphysis injury
S92.310	Open metatarsal fracture
S92.400	Thumb fracture
S92.410	Open fracture of big toe
S92.500	Phalangeal fracture, others
S92.500x001	Phalangeal fracture
S92.500x002	Injury of phalangeal epiphysis
S92.510	Open fracture of special phalange
S92.700	Multiple fractures of foot
S92.700x001	Multiple fracture of foot bone
S92.710	Open multiple foot fractures
S92.900	Foot fracture
S92.910	Open foot fracture
S93.000	Ankle dislocation
S93.000x004	Talus dislocation
S93.000x005	Dislocation of fibula
S93.001	Subluxation of ankle joint
S93.002	Tibial talus joint dislocation
S93.003	Dislocation of distal tibiofibular joint
S93.100	Toe dislocation
S93.100x001	Dislocation of phalanx
S93.101	Dislocation of toe joint
S93.102	Metatarsophalangeal joint subluxation
S93.103	Metatarsophalangeal joint dislocation
S93.200	Ligament rupture at ankle and foot level
S93.200x001	Fracture of ankle and foot ligaments
S93.200x002	Fracture of ankle ligament
S93.200x003	Fracture of ligament of foot

S93.200x004	Fracture of anterior talofibular ligament of ankle
S93.200x005	Rupture of calcaneus fibular ligament
S93.300	Dislocation of other and unspecified parts of the foot
S93.300x001	Lisfranc damage
S93.300x011	Dislocation of tarsal bone
S93.300x021	Dislocation of middle tarsal joint
S93.300x031	Fracture and dislocation of tarsometatarsal joint [Lisfranc fracture and dislocation]
S93.300x032	Dislocation of tarsometatarsal joint
S93.300x081	Dislocation of talocavicular joint
S93.301	Dislocation of foot
S93.302	Metatarsal dislocation
S93.303	Dislocation of scaphoid bone of foot
S93.400	Ankle sprain and strain
S93.400x002	Ankle joint injury
S93.400x004	Sprain of medial collateral ligament of ankle
S93.400x012	Ankle triangle ligament injury
S93.400x021	Sprain of calcaneus fibular ligament
S93.400x022	Injury of calcaneal fibular ligament
S93.400x031	Distal tibiofibular ligament sprain
S93.400x032	Distal tibiofibular ligament injury
S93.400x041	Ankle cartilage injury
S93.401	Ankle sprain
S93.402	Injury of medial collateral ligament of ankle
S93.403	Triangular ligament rupture
S93.404	Sprain of triangular ligament
S93.405	Tear of distal tibiofibular ligament

S93.500	Sprain and strain of toe
S93.500x001	Sprain of interphalangeal joint
S93.500x002	Injury of interphalangeal joint
S93.500x003	Sprain of metatarsophalangeal joint
S93.500x004	Metatarsophalangeal joint injury
S93.500x005	Sprain of toe
S93.500x006	Toe injury
S93.600	Sprain and strain of other and unspecified parts of the foot
S93.600x001	Sprain of tarsal ligament
S93.600x002	Injury of tarsal ligament
S93.600x003	Sprain of tarsometatarsal ligament
S93.600x004	Injury of tarsometatarsal ligament
S93.601	Sprain of foot
S94.000	Injury of lateral plantar nerve
S94.100	Medial plantar nerve injury
S94.200	Injury of deep peroneal nerve at ankle and foot level
S94.200x001	Injury of deep peroneal nerve of ankle and foot
S94.200x002	End injury of external collateral branch of deep peroneal nerve
S94.300	Dermatosensory nerve injury at ankle and foot level
S94.300x001	Sensory nerve injury of ankle and foot skin
S94.700	Multiple nerve injuries at ankle and foot levels
S94.700x001	Multiple nerve injuries of ankle and foot
S94.800	Other nerve injuries at ankle and foot levels
S94.800x001	Injury of toe nerve

S94.900	Nerve injuries at the ankle and foot levels
S94.900x001	Injury of ankle and foot nerves
S95.000	Injury of dorsalis pedis artery
S95.100	Plantar artery injury
S95.200	Injury of dorsal vein of foot
S95.700	Multivascular injuries at ankle and foot levels
S95.700x001	Multiple vascular injuries of ankle and foot
S95.800	Other vascular injuries at ankle and foot levels
S95.900	Injury of blood vessels at ankle and foot level
S95.900x001	Injury of ankle and foot vessels
S96.000	Injury of flexor digitorum longus and tendon at ankle and foot level
S96.000x001	Injury of flexor longus and tendon of ankle and toe
S96.100	Injury of extensor digitorum longus and tendon at ankle and foot level
S96.100x001	Injury of extensor longus and tendon of ankle and toe
S96.100x002	Tendon injury of long hallucis pedis
S96.101	Rupture of extensor hallucis longus tendon
S96.102	Tendon rupture of extensor digitorum
S96.200	INNER MUSCLE AND TENDON INJURY AT ANKLE AND FOOT LEVEL
S96.200x001	Injury of internal muscles and tendons of ankle and foot
S96.700	Multiple muscle and tendon injuries at ankle and foot levels
S96.700x001	Multiple muscle and tendon injuries of ankle and foot

S96.701	Multiple tendon injuries at ankle and foot levels
S96.800	Other muscle and tendon injuries at ankle and foot levels
S96.800x001	Injury of posterior tibial tendon of ankle
S96.800x002	Injury of tendon of toe
S96.801	Toe tendon rupture
S96.900	Muscle and tendon injuries at ankle and foot levels
S96.900x002	Injuries to ankle and foot muscles and tendons
S97.000	Crushing injury of ankle
S97.000x001	Crushing injury of ankle
S97.000x011	Ankle thermal crush injury
S97.100	Crushing injury of toe
S97.100x001	Crushing injury of toe
S97.100x011	Thermal crush injury of toe
S97.800	Crushing injury of ankle and other parts of foot
S97.800x001	Crushing injury of foot
S97.800x002	Crushing injury of ankle and foot
S97.800x011	Foot thermal crush injury
S97.801	Foot crush injury
S98.000	Traumatic amputation of foot at ankle level
S98.000x001	Ankle amputation
S98.100	Traumatic amputation of one toe
S98.100x001	Single toe amputation
S98.200	Traumatic amputation of two or more toes
S98.200x001	Amputation of two toes
S98.200x002	Mutitoectomy

S98.300	Traumatic amputation of other parts of the foot
S98.400	Traumatic amputation of foot
S99.700	Multiple injuries of ankle and foot
S99.700x001	Multiple foot injuries
S99.700x002	Multiple injuries of ankle
S99.800	Other specific injuries of ankle and foot
S99.800x001	Avulsion injury of foot soft tissue
S99.900	Ankle and foot injuries
S99.900x001	Foot injury
S99.900x002	Ankle injury
T00.000	Superficial injury involving head and neck
T00.000x001	Superficial head and neck injuries
T00.100	Superficial injury involving chest with abdomen, lower back and pelvis
T00.100x001	Thorax with superficial injury of abdomen, lower back and pelvis
T00.200	Superficial injury involving multiple parts of upper limb
T00.200x001	Multiple superficial injuries of upper limbs
T00.300	Superficial injuries involving multiple parts of lower limbs
T00.300x001	Multiple superficial injuries of lower limbs
T00.600	Superficial injury involving upper limbs with multiple parts of lower limbs
T00.600x001	Multiple superficial injuries of upper and lower limbs
T00.800	Superficial injuries involving other complex parts of the body
T00.800x001	Superficial injury of compound body parts

T00.900	Multiple superficial damages
T00.900x001	Multiple superficial skin injuries
T00.900x002	Multiple superficial skin abrasions
T00.900x003	Multiple superficial skin blisters
T00.900x004	Multiple superficial skin bruises
T00.900x005	Multiple superficial skin contusions
T00.900x006	Multiple superficial hematoma of skin
T00.900x007	Multiple superficial non-toxic insect bites
T00.901	Multiple contusions
T00.902	Multiple skin damage
T01.000	Open wound involving head and neck
T01.000x001	Open head and neck injuries
T01.100	Open wound involving chest with abdomen, lower back and pelvis
T01.100x001	Open injury of chest with abdomen, lower back and pelvis
T01.101	Open thoracoabdominal injury
T01.200	Open wound involving multiple parts of upper limb
T01.200x001	Multiple open injuries of upper limbs
T01.300	Open wound involving multiple parts of lower limbs
T01.300x001	Multiple open injuries of lower limbs
T01.301	Lower limb skin avulsion
T01.302	Multiple lacerations of lower limbs
T01.600	Open wound involving upper limb and multiple parts of lower limb
T01.600x001	Multiple open injuries of upper and lower limbs
T01.800	Open wound involving other composite body parts

T01.800x001	Open injury of compound body parts
T01.900	Multiple open wounds
T01.901	Multiple puncture injuries
T01.902	Multiple animal bites
T01.903	Multiple cutting injuries
T01.904	Multiple laceration
T02.000	Involved head with neck fracture
T02.000x001	Head and neck fractures
T02.010	Open head with neck fracture
T02.100	Fracture involving chest with lower back and pelvis
T02.100x001	Multiple fracture of trunk
T02.110	Open multiple trunk fractures
T02.200	Fracture involving multiple parts of single upper limb
T02.200x001	Multiple fractures of single upper limb
T02.210	Open multiple fractures of single upper limb
T02.300	Fracture involving multiple parts of single lower limb
T02.300x001	Multiple fractures of single lower limb
T02.310	Open multiple fractures of single lower limb
T02.400	Fracture involving multiple parts of both upper limbs
T02.400x001	Multiple fractures of both upper limbs
T02.410	Open multiple fractures of both upper limbs
T02.500	Fracture involving multiple parts of both lower limbs
T02.500x001	Multiple fractures of both lower limbs
T02.510	Open multiple fractures of both lower limbs

T02.600	Fracture involving upper limb with multiple parts of lower limb
T02.600x001	Upper limb with multiple fractures of lower limb
T02.600x011	Multiple open fractures of upper limb with lower limb
T02.610	Open multiple limb fractures
T02.700	Fracture involving chest with lower back, pelvis and limbs
T02.700x001	Thorax with fracture of lower back, pelvis and extremities
T02.700x021	Pelvis with bone fracture of lower limb
T02.710	Open chest with fracture of lower back, pelvis and extremities
T02.800	Fracture involving other complex parts of the body
T02.800x001	Fracture of compound part of the body
T02.810	Open fracture of the body, especially the compound part
T02.900	Multiple fractures
T02.910	Open multiple fractures
T03.000	Involved head with dislocation, sprain and strain of neck
T03.000x001	Dislocation of head and neck
T03.000x002	Head and neck sprains
T03.000x003	Head and neck injuries
T03.100	Involve the chest with dislocation, sprain and strain of lower back and pelvis
T03.100x001	Thorax with dislocation of lower back and pelvis
T03.100x002	Thorax with lower back and pelvis sprain
T03.100x003	Thorax with injury of lower back and pelvis
T03.200	Dislocation, sprain and strain involving multiple parts of upper limbs

T03.200x001	Multiple dislocations of upper limbs
T03.200x002	Multiple sprains of upper limbs
T03.200x003	Multiple injuries of upper limbs
T03.300	Dislocation, sprain and strain involving multiple parts of lower limbs
T03.300x001	Multiple dislocations of lower limbs
T03.300x002	Multiple sprains of lower limbs
T03.300x003	Multiple injuries of lower limbs
T03.400	Involved upper limbs with dislocation, sprain and strain of multiple parts of lower limbs
T03.400x001	Multiple dislocations of upper and lower limbs
T03.400x002	Multiple sprains of upper and lower limbs
T03.400x003	Multiple injuries of upper and lower limbs
T03.800	Dislocation, sprain and strain involving other complex parts of the body
T03.800x001	Dislocation of complex parts of the body
T03.800x002	Sprain of compound parts of the body
T03.900	Multiple dislocations, sprains and strains
T03.900x001	Multiple dislocations
T03.900x002	Multiple sprains
T04.000	Crushing injury involving head and neck
T04.000x001	Head and neck crush injury
T04.000x011	Head with heat crush injury of neck
T04.100	Crushing injury involving chest with abdomen, lower back and pelvis
T04.100x001	Crushing injury of trunk
T04.100x011	Thorax with abdominal, lower back and pelvis thermal crush injury

T04.200	Crushing injury involving multiple parts of upper limb
T04.200x001	Multiple crush injuries of upper limbs
T04.200x011	Multi part heat crush injury of upper limb
T04.300	Crushing injury involving multiple parts of lower limbs
T04.300x001	Multiple crush injuries of lower limbs
T04.300x011	Heat crush injury of multiple parts of lower limb
T04.400	Crushing injury involving upper limbs with multiple parts of lower limbs
T04.400x001	Multiple crush injuries of upper and lower limbs
T04.400x011	Upper limb with multiple parts of lower limb thermal crush injury
T04.700	Thorax with crush injury of abdomen, lower back, pelvis and limbs
T04.700x001	Thorax with compression injury of abdomen, lower back and pelvis and extremities
T04.700x011	Thorax with heat crush injury to abdomen, lower back, pelvis and extremities
T04.800	Crushing injury involving other complex parts of the body
T04.800x001	Crushing injury of compound body parts
T04.900	Multiple crushing injuries
T04.900x011	Multiple thermal crush injury
T04.900x012	Systemic thermal crush injury
T04.901	Systemic crush injury
T05.000	Traumatic amputation of hands
T05.100	Traumatic amputation of one hand and the other arm [at any level except for the other hand]
T05.100x001	Traumatic amputation of hand and contralateral arm

T05.200	Traumatic amputation of both arms [at any level]
T05.200x001	Traumatic amputation of both arms
T05.300	Traumatic amputation of both feet
T05.300x002	Partial traumatic amputation of both feet
T05.400	Traumatic amputation of one foot and the other leg [at any level except foot]
T05.400x001	Traumatic amputation of foot and opposite leg
T05.500	Traumatic amputation of both lower legs [at any level]
T05.500x001	Traumatic amputation of both lower legs
T05.600	Traumatic amputation of any combination of upper and lower limbs [at any level]
T05.600x001	Traumatic amputation of upper and lower limbs
T05.800	Traumatic amputation involving other complex parts of the body
T05.800x001	Thoracic traumatic amputation
T05.800x002	Traumatic abdominal amputation
T05.800x003	Traumatic amputation of complex body parts
T05.900	Multiple traumatic amputations
T06.000	Brain nerve injury with nerve and spinal cord injury at the cervical level
T06.000x001	Brain nerve injury with cervical nerve and spinal cord injury
T06.100	Nerve and spinal cord injuries involving other parts of the body
T06.100x001	Multiple nerve and spinal cord injuries
T06.101	Peripheral spinal nerve injury
T06.200	Nerve injuries involving multiple parts of the body

T06.200x001	Multiple nerve injuries
T06.300	Vascular injuries involving multiple parts of the body
T06.300x001	Multiple vascular injuries
T06.400	Muscle and tendon injuries involving multiple parts of the body
T06.400x001	Multiple muscle and tendon injuries
T06.400x002	Multiple muscle injuries
T06.401	Multiple tendon injuries
T06.500	Thoracic organs with damage to abdominal and pelvic organs
T06.500x001	Open injury of intrathoracic organs with abdominal and pelvic organs
T06.500x002	Intrathoracic organs with abdominal and pelvic organ damage
T06.501	Multiple organ injury
T06.800	Other specific injuries involving multiple parts of the body
T06.800x001	Injuries in complex parts of the body
T07.x00	Multiple damages
T08.x00	Spinal fracture
T08.x10	Open spinal fracture
T09.000	Superficial injury of trunk
T09.000x011	Superficial abrasion of trunk
T09.000x021	Superficial blister of trunk
T09.000x031	Superficial insect bite of trunk
T09.000x041	Foreign body on superficial trunk
T09.000x051	Superficial contusion of trunk
T09.100	Open wound of trunk
T09.100x001	Open injury of trunk
T09.200	Dislocation, sprain and strain of trunk joints and ligaments

T09.200x001	Dislocation of trunk joints and ligaments
T09.200x002	Dislocation of trunk joint
T09.200x003	Dislocation of trunk ligament
T09.200x004	Torso joint and ligament sprain
T09.200x005	Torso joint sprain
T09.200x006	Sprain of trunk ligament
T09.200x007	Injury of trunk joints and ligaments
T09.200x008	Torso joint injury
T09.200x009	Injury of trunk ligament
T09.300	Spinal cord injury
T09.300x003	Complete spinal cord injury
T09.300x004	Central spinal cord injury syndrome
T09.300x005	Anterior cord syndrome
T09.300x006	Posterior cord syndrome
T09.300x007	Spinal hematoma
T09.301	Traumatic paraplegia
T09.400	Injury of trunk nerve, spinal nerve root and nerve plexus
T09.400x001	Spinal nerve injury
T09.400x002	Injury of spinal nerve root
T09.400x003	Injury of spinal nerve plexus
T09.500	Injury of trunk muscles and tendons
T09.500x001	Injury of trunk muscles and tendons
T09.500x002	Injury of trunk muscle
T09.500x003	Injury of trunk tendon
T09.600	Traumatic amputation of trunk
T09.800	Other specific injuries to the trunk
T09.900	Torso injury
T10.x00	Fracture of upper limb

T10.x10	Open fracture of upper limb
T11.000	Superficial injury of upper limb
T11.000x011	Superficial abrasion of upper limb
T11.000x021	Superficial vesicles of upper limbs
T11.000x031	Upper limb superficial insect bite
T11.000x041	Superficial foreign body of upper limb
T11.000x051	Superficial contusion of upper limb
T11.001	Upper limb abrasions
T11.100	Open wound of upper limb
T11.101	Skin laceration of upper limb
T11.102	Avulsion injury of upper limb
T11.200	Dislocation, sprain and strain of joints and ligaments of upper limbs
T11.200x001	Dislocation of joints and ligaments of upper limbs
T11.200x002	Dislocation of upper limb joint
T11.200x003	Dislocation of upper limb ligaments
T11.200x004	Sprain of joints and ligaments of upper limbs
T11.200x005	Sprain of upper limb joint
T11.200x006	Sprain of upper limb ligament
T11.200x007	Injuries of joints and ligaments of upper limbs
T11.200x008	Upper limb joint injury
T11.200x009	Ligament injury of upper limb
T11.300	Injury of upper limb nerves
T11.400	Injury of upper limb blood vessels
T11.500	Injury of upper limb muscles and tendons
T11.500x002	Upper limb muscle injury
T11.500x003	Upper limb tendon injury

T11.600	Traumatic amputation of upper limb
T11.600x001	Traumatic amputation of arm
T11.800	Other specific injuries of upper limbs
T11.900	Upper extremity injury
T12.x00	Lower limb fracture
T12.x10	Open fracture of lower limb
T13.000	Superficial injury of lower limb
T13.000x011	Superficial abrasion of lower limb
T13.000x021	Superficial vesicles of lower limbs
T13.000x031	Lower limb superficial insect bite
T13.000x041	Superficial foreign body of lower limb
T13.000x051	Superficial contusion of lower limbs
T13.001	Hematoma of lower limbs
T13.100	Open wound of lower limb
T13.100x003	Avulsion injury of lower limb
T13.100x004	Exfoliation injury of lower limb
T13.101	Lower limb skin laceration
T13.200	Dislocation, sprain and strain of joints and ligaments of lower limbs
T13.200x002	Lower limb joint dislocation
T13.200x003	Dislocation of ligaments of lower limbs
T13.200x005	Lower limb joint sprain
T13.200x006	Lower limb ligament sprain
T13.200x007	Injuries of joints and ligaments of lower limbs
T13.200x008	Lower limb joint injury
T13.200x009	Lower limb ligament injury
T13.201	Dislocation of joints and ligaments of lower limbs

T13.202	Sprain of joints and ligaments of lower limbs
T13.203	Strain of joints and ligaments of lower limbs
T13.300	Nerve injury of lower limbs
T13.400	Injury of lower limb blood vessels
T13.500	Injury of lower limb muscles and tendons
T13.501	Lower limb muscle injury
T13.502	Lower limb tendon injury
T13.600	Traumatic amputation of lower limb
T13.800	Other specific injuries of lower limbs
T13.900	Lower limb injury
T14.000	Superficial damage
T14.000x001	Superficial body injury
T14.000x002	Superficial haematoma of the body
T14.000x003	Surfer tubercle
T14.000x011	Superficial body abrasion
T14.000x021	Superficial blister
T14.000x031	Superficial insect bite
T14.000x041	Superficial foreign body
T14.000x051	Superficial body contusion
T14.001	skin contusion
T14.002	Non toxic spider bite
T14.003	Subcutaneous hematoma
T14.100	Open wound
T14.101	Skin laceration
T14.200	Body fracture
T14.210	Open fracture
T14.300	Dislocation, sprain and strain

T14.400	Nerve injury
T14.500	Vascular injury
T14.501	Traumatic aneurysm
T14.600	Tendon and muscle injuries
T14.601	Tendon injury
T14.602	Muscle injury
T14.700	Crushing injury and traumatic amputation
T14.701	Crush injury
T14.702	Traumatic amputation
T14.800	Other damages
T14.900	damage
T15.000	Corneal foreign body
T15.100	Foreign body of conjunctival capsule
T15.100x001	Foreign body of conjunctiva
T15.100x002	Non traumatic eyelid foreign body
T15.101	Foreign body of eyelid
T15.800	Foreign bodies in other and multiple parts of the external eye
T15.800x001	Foreign body of eyeball
T15.800x002	Foreign body in lacrimal point
T15.800x003	Multiple foreign bodies in the external eye
T15.801	Multiple foreign bodies in the external eye
T15.900	Foreign body of external eye
T16.x00	Intraauricular foreign body
T16.x00x001	foreign bodies
T16.x00x002	Foreign body in middle ear
T17.000	Foreign body in sinus
T17.001	Foreign body in maxillary sinus

T17.002	Foreign body in ethmoid sinus
T17.100	Foreign body in nostril
T17.101	Foreign body in nasal cavity
T17.200	Foreign body in pharynx
T17.200x001	Foreign body in nasopharynx
T17.300	Foreign body in larynx
T17.400	Foreign body in trachea
T17.500	Foreign body in bronchus
T17.501	plastic bronchitis
T17.800	Foreign bodies in other and multiple parts of respiratory tract
T17.801	Multiple foreign body in respiratory tract
T17.802	Foreign body in bronchiole
T17.803	Pulmonary mucus embolism
T17.804	Foreign body in lung
T17.900	Foreign body in respiratory tract
T17.901	Asphyxia due to inhalation of foreign matters
T18.000	Intraoral foreign body
T18.001	Foreign body in oral soft tissue
T18.002	Foreign body of tongue
T18.100	Foreign body in esophagus
T18.200	Intragastric foreign body
T18.300	Foreign body in small intestine
T18.300x003	Foreign body in jejunum
T18.301	Foreign body of duodenum
T18.400	Foreign body in colon
T18.500	Foreign body in anus and rectum
T18.500x004	Foreign body in rectosigmoid junction

T18.501	Intraanal foreign body
T18.502	Foreign body in rectum
T18.800	Foreign bodies in other and multiple parts of digestive tract
T18.801	Multiple foreign bodies in digestive tract
T18.900	Foreign body in digestive tract
T19.000	Foreign body in urethra
T19.100	Foreign body in bladder
T19.200	Foreign body in vulva and vagina
T19.201	Foreign body of vulva
T19.202	Foreign body in vagina
T19.300	Intrauterine foreign body [any part]
T19.800	Foreign bodies in other and multiple parts of urogenital tract
T19.800x001	Foreign body in ureter
T19.800x002	Foreign body in penis
T19.801	Multiple foreign bodies in genitourinary tract
T19.900	Foreign body in genitourinary tract
T20.000	Burns to head and neck
T20.000x002	Burn of head
T20.000x003	Neck burn
T20.000x004	Scalp burn
T20.000x006	Nose burn
T20.000x007	Temporal burn
T20.000x008	Burn of lips
T20.000x010	Eye with head burn
T20.000x011	Eye with neck burn
T20.000x012	Eye with facial burn

T20.000x013	Ear scald
T20.000x014	Facial scald
T20.000x015	Chemical burns on the face
T20.000x021	Scald of head and neck
T20.000x022	Chemical burns to head and neck
T20.002	Ear burn
T20.003	Facial burn
T20.100	First degree burn of head and neck
T20.100x002	First degree burn of head
T20.100x003	First degree burn of neck
T20.100x004	First degree burn of scalp
T20.100x005	First degree facial burn
T20.100x006	First degree burn of nose
T20.100x007	First degree burn of temporal region
T20.100x008	First degree burn of lip
T20.100x009	First degree burn of ear
T20.100x010	Eye with first degree burn of head
T20.100x011	First degree burn of eye with neck
T20.100x012	First degree burn of eye and face
T20.100x021	First degree scald of head and neck
T20.100x022	First degree chemical burn of head and neck
T20.200	Second degree burn of head and neck
T20.200x002	Second degree burn of head
T20.200x003	Second degree burn of neck
T20.200x004	Second degree burn of scalp
T20.200x006	Second degree burn of nose
T20.200x007	Second degree burn of temporal region
T20.200x008	Second degree burn of lip

T20.200x009	Second degree burn of ear
T20.200x010	Eye with second degree burn of head
T20.200x011	Second degree burn of eye with neck
T20.200x012	Second degree burn of eye with face
T20.200x021	Second degree scald of head and neck
T20.200x022	Second degree chemical burn of head and neck
T20.200x023	Second degree facial scald
T20.200x024	Second degree chemical burn on face
T20.201	Second degree burn of face
T20.300	Third degree burn of head and neck
T20.300x002	Third degree burn of head
T20.300x003	Third degree burn of neck
T20.300x004	Third degree burn of scalp
T20.300x005	Third degree facial burn
T20.300x006	Third degree burn of nose
T20.300x007	Third degree burn of temporal region
T20.300x008	Third degree burn of lip
T20.300x009	Third degree burn of ear
T20.300x010	Eye with third degree burn of head
T20.300x011	Third degree burn of eye with neck
T20.300x012	Third degree burn of eye with face
T20.300x021	Third degree scald of head and neck
T20.300x022	Third degree chemical burn of head and neck
T20.400	Head and neck corrosion
T20.400x002	Head corrosion
T20.400x003	Corrosive injury of neck
T20.400x004	Scalp corrosion
T20.400x005	Facial erosion

T20.400x006	Nasal corrosion
T20.400x007	Temporal corrosion
T20.400x008	Lip corrosion
T20.400x009	Ear corrosion
T20.400x010	Eye with head corrosion
T20.400x011	Eye with corrosion injury of neck
T20.400x012	Eye with surface corrosion
T20.401	Chemical burn of ear
T20.500	First degree corrosion injury of head and neck
T20.500x002	Head was once corroded
T20.500x003	First degree corrosion wound on neck
T20.500x004	Once corroded scalp
T20.500x005	Once corrosive wound on face
T20.500x006	Once corrosive wound on nose
T20.500x007	Temporal corrosion
T20.500x008	Once corroded lips
T20.500x009	Ear was once corroded
T20.500x010	Primary corrosion injury of eye and head
T20.500x011	First degree corrosion injury of eye and neck
T20.500x012	One degree of corrosion injury on eye companion surface
T20.600	Second degree corrosion injury of head and neck
T20.600x002	Second degree corrosion of head
T20.600x003	Secondary corrosion injury of neck
T20.600x004	Second degree corrosion injury of scalp
T20.600x005	Second degree corrosion injury on face

T20.600x006	Second degree corrosion wound of nose
T20.600x007	Secondary corrosion injury of temporal region
T20.600x008	Secondary corrosion injury of lip
T20.600x009	Second degree corrosion injury of ear
T20.600x010	Eye with secondary corrosion injury of head
T20.600x011	Eye with secondary corrosion injury of neck
T20.600x012	Secondary corrosion injury of eye associated surface
T20.700	Third degree corrosion injury of head and neck
T20.700x002	Third degree corrosion of head
T20.700x003	Third degree corrosion injury of neck
T20.700x004	Third degree corrosion of scalp
T20.700x005	Third degree facial corrosion
T20.700x006	Third degree corrosion wound of nose
T20.700x007	Third degree corrosion injury of temporal region
T20.700x008	Third degree corrosion injury on lips
T20.700x009	Third degree corrosion injury of ear
T20.700x010	Eye with third degree corrosion injury of head
T20.700x011	Eye with third degree corrosion injury of neck
T20.700x012	Third degree corrosion injury of eye associated surface
T21.000	Burn of trunk
T21.000x011	Burn of breast
T21.000x021	Burn of chest wall
T21.000x031	Abdominal wall burn
T21.000x032	Hypochondriac burn

T21.000x033	Inguinal burn
T21.000x041	Bum of buttock
T21.000x042	Back burn
T21.000x043	Burn of interscapular region
T21.000x051	Burn of labia majora
T21.000x052	Burn of labia minora
T21.000x053	Burn of penis
T21.000x054	Perineal burn
T21.000x055	Scrotal burn
T21.000x056	Testicular burn
T21.000x057	Vulvar burn
T21.000x091	Anal burn
T21.100	First degree burn of trunk
T21.100x011	First degree burn of breast
T21.100x021	First degree burn of chest wall
T21.100x031	First degree burn of abdominal wall
T21.100x032	First degree burn of flank
T21.100x033	First degree burn of groin
T21.100x041	First degree burn of buttock
T21.100x042	First degree burn of back
T21.100x043	First degree burn of interscapular region
T21.100x051	First degree burn of labia majora
T21.100x052	First degree burn of labia minora
T21.100x053	First degree burn of penis
T21.100x054	First degree burn of perineum
T21.100x055	First degree burn of scrotum
T21.100x056	First degree burn of testis
T21.100x057	First degree burn of vulva

T21.100x091	First degree burn of anus
T21.100x101	Once scalded trunk
T21.100x102	One degree of chemical burn on the trunk
T21.200	Second degree burn of trunk
T21.200x011	Second degree burn of breast
T21.200x021	Second degree burn of chest wall
T21.200x031	Second degree burn of abdominal wall
T21.200x032	Second degree burn of flank
T21.200x033	Second degree burn of groin
T21.200x041	Second degree burn of buttock
T21.200x042	Second degree burn of back
T21.200x043	Second degree burn of interscapular region
T21.200x051	Second degree burn of labia majora
T21.200x052	Second degree burn of labia minora
T21.200x053	Second degree burn of penis
T21.200x054	Second degree burn of perineum
T21.200x055	Second degree burn of scrotum
T21.200x056	Second degree burn of testis
T21.200x057	Second degree burn of vulva
T21.200x091	Second degree burn of anus
T21.200x101	Second degree scald of trunk
T21.200x102	Second degree chemical burn of trunk
T21.300	Third degree burn of trunk
T21.300x011	Third degree burn of breast
T21.300x021	Third degree burn of chest wall
T21.300x031	Third degree burn of abdominal wall
T21.300x032	Third degree burn of flank
T21.300x033	Third degree burn of groin

T21.300x041	Third degree burn of buttock
T21.300x042	Third degree burn of back
T21.300x043	Third degree burn of interscapular region
T21.300x051	Third degree burn of labia majora
T21.300x052	Third degree burn of labia minora
T21.300x053	Third degree burn of penis
T21.300x054	Third degree burn of perineum
T21.300x055	Third degree burn of scrotum
T21.300x056	Third degree burn of testis
T21.300x057	Third degree burn of vulva
T21.300x091	Third degree burn of anus
T21.300x101	Third degree scald of trunk
T21.300x102	Third degree chemical burn of trunk
T21.400	Torso corrosion
T21.400x011	Corrosive wound of breast
T21.400x021	Thoracic wall corrosion
T21.400x031	Corrosive wound of abdominal wall
T21.400x032	Flank corrosion
T21.400x033	Inguinal corrosion
T21.400x041	Hip corrosion injury
T21.400x042	Back corrosion
T21.400x043	Corrosion wound of scapular region
T21.400x051	Corrosion of labia majora
T21.400x052	Corrosion wound of labia minora
T21.400x053	Penis corrosion
T21.400x054	Perineal corrosion
T21.400x055	Corrosive wound of scrotum
T21.400x056	Testicular corrosion

T21.400x057	Vulva corrosion
T21.400x091	Erosion of anus
T21.500	One degree of corrosion injury to the trunk
T21.500x011	Once corrosive wound of breast
T21.500x021	Once corrosive wound of chest wall
T21.500x031	First degree corrosion of abdominal wall
T21.500x032	One degree corrosion damage on flank
T21.500x033	One degree of corrosion of groin
T21.500x041	Once corrosive wound on hip
T21.500x042	One degree of corrosion on the back
T21.500x043	One degree corrosion wound in the interscapular region
T21.500x051	The labia majora was once corroded
T21.500x052	The labia minora was once corroded
T21.500x053	Once corroded penis
T21.500x054	First degree corrosion of perineum
T21.500x055	Once corrosive wound of scrotum
T21.500x056	First degree corrosion injury of testis
T21.500x057	One degree of vulva corrosion
T21.500x091	Once corrosive wound of anus
T21.600	Second degree corrosion injury of trunk
T21.600x011	Secondary corrosion injury of breast
T21.600x021	Secondary corrosion injury of chest wall
T21.600x031	Second degree corrosion of abdominal wall
T21.600x032	Secondary corrosion of flank
T21.600x033	Second degree corrosion of groin

T21.600x041	Second degree corrosion injury of hip
T21.600x042	Second degree corrosion on the back
T21.600x043	Second degree corrosion injury of scapular region
T21.600x051	Second degree corrosion of labia majora
T21.600x052	Secondary corrosion of labia minora
T21.600x053	Secondary corrosion injury of penis
T21.600x054	Secondary corrosion injury of perineum
T21.600x055	Secondary corrosion wound of scrotum
T21.600x056	Secondary corrosion injury of testis
T21.600x057	Secondary corrosion of vulva
T21.600x091	Second degree corrosion wound of anus
T21.700	Third degree corrosion injury of trunk
T21.700x011	Third degree corrosion wound of breast
T21.700x021	Third degree corrosion injury of chest wall
T21.700x031	Third degree corrosion of abdominal wall
T21.700x032	Third degree corrosion of flank
T21.700x033	Third degree corrosion of groin
T21.700x041	Third degree corrosion injury of hip
T21.700x042	Third degree corrosion on the back
T21.700x043	Third degree corrosion wound of scapular region
T21.700x051	Third degree corrosion of labia majora
T21.700x052	Third degree corrosion of labia minora
T21.700x053	Third degree corrosion injury of penis
T21.700x054	Third degree corrosion of perineum
T21.700x055	Third degree corrosion wound of scrotum

T21.700x056	Third degree corrosion injury of testis
T21.700x057	Third degree corrosion of vulva
T21.700x091	Third degree corrosion wound of anus
T22.000	Burns to shoulders and upper limbs, except wrists and hands
T22.000x001	Burns of shoulder and upper limbs
T22.000x002	Upper extremity burn
T22.000x003	Shoulder burn
T22.000x004	Burn of scapular region
T22.000x005	Arm burn
T22.000x006	Axillary burn
T22.100	First degree burn of shoulder and upper limb, except wrist and hand
T22.100x001	First degree burn of shoulder and upper limb
T22.100x002	First degree burn of upper limb
T22.100x003	First degree burn of shoulder
T22.100x004	First degree burn of scapular region
T22.100x005	First degree burn of arm
T22.100x006	First degree burn of axilla
T22.100x011	Once scalded shoulder and upper limb
T22.100x012	One degree chemical burn of shoulder and upper limb
T22.200	Second degree burn of shoulder and upper limb, except wrist and hand
T22.200x001	Second degree burn of shoulder and upper limb
T22.200x002	Second degree burn of upper limb
T22.200x003	Second degree burn of shoulder
T22.200x004	Second degree burn of scapular region
T22.200x005	Second degree burn of arm
T22.200x006	Second degree burn of axilla

T22.200x011	Second degree scald of shoulder and upper limb
T22.200x012	Second degree chemical burn of shoulder and upper limb
T22.300	Third degree burn of shoulder and upper limb, except wrist and hand
T22.300x001	Third degree burn of shoulder and upper limb
T22.300x002	Third degree burn of upper limb
T22.300x003	Third degree burn of shoulder
T22.300x004	Third degree burn of scapular region
T22.300x005	Third degree burn of arm
T22.300x006	Third degree burn of axilla
T22.300x011	Third degree scald of shoulder and upper limb
T22.300x012	Third degree chemical burn of shoulder and upper limb
T22.400	Corrosive injuries to shoulders and upper limbs, except wrists and hands
T22.400x001	Corrosion injury of shoulder and upper limb
T22.400x002	Corrosion injury of upper limb
T22.400x003	Shoulder corrosion
T22.400x004	Corrosion injury of scapular region
T22.400x005	Arm corrosion
T22.400x006	Axillary corrosion
T22.500	Once corrosive injury to shoulder and upper limb, except wrist and hand
T22.500x001	Once corrosive wound on shoulder and upper limb
T22.500x002	First degree corrosion injury of upper limb
T22.500x003	Shoulder once corroded
T22.500x004	One degree corrosion wound in scapular region

T22.500x005	One degree corrosion of arm
T22.500x006	First degree corrosion injury of axilla
T22.600	Second degree corrosion injury of shoulder and upper limb, except wrist and hand
T22.600x001	Second degree corrosion injury of shoulder and upper limb
T22.600x002	Second degree corrosion injury of upper limb
T22.600x003	Second degree corrosion of shoulder
T22.600x004	Secondary corrosion injury of scapular region
T22.600x005	Second degree corrosion of arm
T22.600x006	Secondary corrosion injury of axilla
T22.700	Third degree corrosion injury to shoulder and upper limb, except wrist and hand
T22.700x001	Third degree corrosion injury of shoulder and upper limb
T22.700x002	Third degree corrosion injury of upper limb
T22.700x003	Third degree corrosion of shoulder
T22.700x004	Third degree corrosion injury of scapular region
T22.700x005	Third degree corrosion of arm
T22.700x006	Third degree corrosion injury of axilla
T23.000	Burns of wrists and hands of unspecified degree
T23.000x001	Wrist and hand burns
T23.000x002	Burn of wrist
T23.000x003	Hand burns
T23.000x004	Palm burn
T23.000x005	Thumb burn
T23.000x006	Finger burn

T23.000x007	Nail burn
T23.100	First degree burn of wrist and hand
T23.100x002	First degree burn of wrist
T23.100x003	First degree burn of hand
T23.100x004	First degree burn of palm
T23.100x005	First degree burn of thumb
T23.100x006	First degree burn of fingers
T23.100x007	First degree burn of nail
T23.100x011	Wrist and hand once scalded
T23.100x012	First degree chemical burns on wrist and hand
T23.200	Second degree burn of wrist and hand
T23.200x002	Second degree burn of wrist
T23.200x003	Second degree burn of hand
T23.200x004	Second degree burn of palm
T23.200x005	Second degree burn of thumb
T23.200x006	Second degree burn of finger
T23.200x007	Second degree burn of nail
T23.200x011	Second degree scald of wrist and hand
T23.200x012	Second degree chemical burn of wrist and hand
T23.300	Third degree burn of wrist and hand
T23.300x002	Third degree burn of wrist
T23.300x003	Third degree burn of hand
T23.300x004	Third degree burn of palm
T23.300x005	Third degree burn of thumb
T23.300x006	Third degree burn of finger
T23.300x007	Third degree burn of nail
T23.300x011	Third degree scald of wrist and hand
T23.300x012	Third degree chemical burn of wrist and hand
T23.300x013	Deep third degree burn of wrist and hand
T23.300x014	Third degree deep scald of wrist and hand
T23.300x015	Deep third degree chemical burn of wrist and hand
T23.400	Wrist and hand corrosion
T23.400x002	Wrist corrosion
T23.400x003	Hand corrosion
T23.400x004	Palm corrosion
T23.400x005	Corrosion injury of thumb
T23.400x006	Finger corrosion
T23.400x007	Nail corrosion
T23.500	Wrist and hand once corroded
T23.500x002	Wrist was once corroded
T23.500x003	Hand was once corroded
T23.500x004	Palm was once corroded
T23.500x005	Once corroded thumb
T23.500x006	Finger once corroded
T23.500x007	Nail once corroded
T23.600	Secondary corrosion injury of wrist and hand
T23.600x002	Second degree corrosion injury of wrist
T23.600x003	Second degree corrosion injury of hand
T23.600x004	Second degree corrosion injury of palm
T23.600x005	Second degree corrosion injury of thumb
T23.600x006	Second degree corrosion of fingers
T23.600x007	Secondary corrosion of nail
T23.700	Third degree corrosion injury of wrist and hand

T23.700x002	Third degree corrosion injury of wrist
T23.700x003	Third degree corrosion injury of hand
T23.700x004	Third degree corrosion of palm
T23.700x005	Third degree corrosion injury of thumb
T23.700x006	Third degree corrosion of fingers
T23.700x007	Third degree corrosion of nail
T24.000	Burns of the hip and lower limbs, except the ankle and foot
T24.000x001	Hip and lower limb burns
T24.000x002	Hip burn
T24.000x003	Burn of lower limbs
T24.000x004	Calf burn
T24.100	First degree burn of the hip and lower limbs, except the ankle and foot
T24.100x001	First degree burn of hip and lower limb
T24.100x002	First degree burn of hip
T24.100x003	First degree burn of lower limb
T24.100x004	First degree burn of lower leg
T24.100x011	Once scalded hip and lower limbs
T24.100x012	First degree chemical burn of hip and lower limb
T24.200	Second degree burn of hip and lower limb, except ankle and foot
T24.200x001	Second degree burn of hip and lower limb
T24.200x002	Second degree burn of hip
T24.200x003	Second degree burn of lower limb
T24.200x004	Second degree burn of lower leg
T24.200x011	Second degree scald of hip and lower limb
T24.200x012	Second degree chemical burn of hip and lower limb
T24.300	Third degree burn of hip and lower limb, except ankle and foot
T24.300x001	Third degree burn of hip and lower limb
T24.300x002	Third degree burn of hip
T24.300x003	Third degree burn of lower limb
T24.300x004	Third degree burn of lower leg
T24.300x011	Third degree scald of hip and lower limb
T24.300x012	Third degree chemical burn of hip and lower limb
T24.400	Corrosion injury of hip and lower limb, except ankle and foot
T24.400x001	Corrosion injury of hip and lower limb
T24.400x002	Hip corrosion
T24.400x003	Lower limb corrosion injury
T24.400x004	Crural corrosion
T24.500	Once corrosive injury of hip and lower limb, except for ankle and foot
T24.500x001	First degree corrosion injury of hip and lower limb
T24.500x002	One time corrosion wound of hip
T24.500x003	First degree corrosion injury of lower limb
T24.500x004	One time corrosion injury of lower leg
T24.600	Second degree corrosion injury of hip and lower limb, except ankle and foot
T24.600x001	Second degree corrosion injury of hip and lower limb
T24.600x002	Second degree corrosion injury of hip
T24.600x003	Secondary corrosion injury of lower limb
T24.600x004	Secondary corrosion injury of lower leg

T24.700	Third degree corrosion injury of hip and lower limb, except ankle and foot
T24.700x001	Third degree corrosion injury of hip and lower limb
T24.700x002	Third degree corrosion injury of hip
T24.700x003	Third degree corrosion injury of lower limb
T24.700x004	Third degree corrosion injury of lower leg
T25.000	Burns to ankle and foot
T25.000x002	Burn of ankle
T25.000x003	Foot burn
T25.100	First degree burn of ankle and foot
T25.100x002	First degree burn of ankle
T25.100x003	First degree burn of foot
T25.100x011	Once scalded ankle and foot
T25.100x012	First degree chemical burn of ankle and foot
T25.200	Second degree burn of ankle and foot
T25.200x002	Second degree burn of ankle
T25.200x003	Second degree burn of foot
T25.200x011	Second degree scald of ankle and foot
T25.200x012	Secondary chemical burn of ankle and foot
T25.300	Third degree burn of ankle and foot
T25.300x002	Third degree burn of ankle
T25.300x003	Third degree burn of foot
T25.300x011	Third degree scald of ankle and foot
T25.300x012	Third degree chemical burn of ankle and foot
T25.400	Corrosion injury of ankle and foot
T25.400x002	Corrosion injury of ankle
T25.400x003	Foot corrosion

T25.500	First degree corrosion injury of ankle and foot
T25.500x002	Once corroded ankle
T25.500x003	One time corrosion injury on foot
T25.600	Secondary corrosion injury of ankle and foot
T25.600x002	Second degree corrosion injury of ankle
T25.600x003	Second degree corrosion injury of foot
T25.700	Third degree corrosion injury of ankle and foot
T25.700x002	Third degree corrosion injury of ankle
T25.700x003	Third degree corrosion injury of foot
T26.000	Burns of eyelid and periocular area
T26.001	Eyelid burn
T26.001x011	Scald of eyelid
T26.001x012	Chemical burn of eyelid
T26.001x013	Scald around eyes
T26.001x014	Chemical burns around the eye
T26.002	Burn of periocular region
T26.100	Burns of cornea and conjunctival sac
T26.100x001	Burns of cornea and conjunctiva
T26.100x003	Conjunctival burn
T26.101	Corneal burn
T26.102	Burn of conjunctival sac
T26.200	Burn accompanied by eyeball rupture and destruction
T26.200x001	Eye burn with eyeball rupture
T26.300	Burns to other parts of eyes and appendages
T26.301	Scleral burn
T26.400	Burns of eyes and appendages

T26.400x001	Ocular burn
T26.400x011	Eye and accessory burns
T26.400x012	Chemical burns to eyes and appendages
T26.400x013	Ocular scald
T26.400x014	Chemical burn of eyeball
T26.401	Eyeball burn
T26.500	Corrosive wound of eyelid and periocular area
T26.500x002	Eyelid corrosion
T26.500x003	Periocular corrosion
T26.600	Corrosive wound of cornea and conjunctival capsule
T26.600x001	Corrosive wound of cornea and conjunctiva
T26.600x002	Corneal erosion
T26.600x003	Conjunctival corrosion
T26.601	Acid burn of cornea with conjunctiva
T26.602	Chemical burn of cornea
T26.603	Alkaline burn of cornea
T26.604	Acid burn of cornea
T26.605	Acid burn of conjunctiva
T26.700	Corrosive injury accompanied by eyeball rupture and destruction
T26.700x001	Eye corrosion injury with eyeball rupture
T26.800	Other parts of eyes and appendages are corroded
T26.800x001	Scleral corrosion
T26.900	Eye and accessory corrosion
T26.900x001	Eye corrosion
T26.901	Acid burn of eyeball

T26.902	Alkaline burn of eyeball
T27.000	Burns of larynx and trachea
T27.000x002	Laryngeal burn
T27.000x003	Trachea burn
T27.100	Burns involving larynx, trachea and lung
T27.100x001	Burns of larynx, trachea and lung
T27.200	Burns in other parts of the respiratory tract
T27.200x001	Thoracic burn
T27.300	Respiratory tract burn
T27.400	Corrosion injury of throat and trachea
T27.400x002	Throat corrosion
T27.400x003	Corrosion injury of trachea
T27.401	Chemical burn of larynx
T27.402	Chemical burn of trachea
T27.500	Corrosive injury involving throat, trachea and lung
T27.500x001	Corrosive injury of larynx, trachea and lung
T27.600	Corrosion injury of other parts of respiratory tract
T27.600x001	Thoracic corrosion injury
T27.700	Corrosive injury of respiratory tract
T28.000	Burns of mouth and pharynx
T28.000x002	Oral burn
T28.000x003	Pharyngeal burn
T28.100	Esophageal burn
T28.200	Burns in other parts of digestive tract
T28.200x001	Gastric burn
T28.200x002	Burn of digestive tract

T28.300	Internal burn of genitourinary organs
T28.300x001	Burns of vagina and uterus
T28.300x002	Vaginal burn
T28.300x003	Uterine burn
T28.400	Burns of internal organs, other and unspecified
T28.401	Burn of internal organs
T28.500	Corrosive wound of mouth and pharynx
T28.500x002	Oral erosion
T28.500x003	Pharyngeal corrosion
T28.501	Chemical burn of oral mucosa
T28.502	Chemical burn of pharynx
T28.600	Esophageal corrosion injury
T28.700	Corrosion injury in other parts of digestive tract
T28.700x001	Gastric corrosion
T28.700x002	Corrosive injury of digestive tract
T28.701	Chemical burn of stomach
T28.702	Corrosive injury of intestine
T28.800	Internal corrosion of genitourinary organs
T28.800x001	Corrosive wound of vagina and uterus
T28.800x002	Corrosive wound of vagina
T28.800x003	Erosive wound of uterus
T28.900	Corrosion of internal organs, others
T28.900x001	Internal organ corrosion
T28.901	Chemical burn of internal organs
T29.000	Burns at multiple sites
T29.000x001	Multiple burns

T29.100	Burns on multiple parts, and the mentioned burns do not exceed one degree
T29.100x001	Multiple first degree burns
T29.100x011	Several first degree burns
T29.200	Burns on multiple parts, and the mentioned burns are not more than two degrees
T29.200x001	Multiple second degree burns
T29.200x011	Multiple secondary burns
T29.300	Burns on multiple parts, at least one third degree burn
T29.300x001	Multiple third degree burns
T29.300x011	Multiple burns At least one third degree burn
T29.400	Corrosion damage of multiple parts
T29.400x001	Multiple corrosion damages
T29.500	Corrosion damage of multiple parts, and the corrosion damage mentioned shall not exceed one degree
T29.500x001	Several primary corrosion damages
T29.600	Corrosion damage at multiple parts, and the corrosion damage mentioned shall not exceed two degrees
T29.600x001	Multiple secondary corrosion injuries
T29.700	Corrosion damage at multiple parts, and at least one third degree corrosion damage
T29.700x001	Multiple third degree corrosion injuries
T30.000	Body burns
T30.000x001	burn
T30.100	First degree burn
T30.100x011	First degree scald
T30.200	Second degree burn
T30.200x011	Second degree scald

T30.300	Third degree burn
T30.300x011	Third degree scald
T30.400	Body corrosion
T30.400x001	Corrosion damage
T30.500	First degree corrosion damage
T30.600	Secondary corrosion damage
T30.700	Third degree corrosion damage
T31.000	Burns involving less than 10% of body surface
T31.000x001	Burns on body surface less than 10%
T31.100	Burns involving 10%~19% of body surface
T31.100x001	Burns on body surface 10-19%
T31.200	Burns involving 20%~29% of body surface
T31.200x001	Burns on body surface 20-29%
T31.300	Burns involving 30%~39% of body surface
T31.300x001	Burns of 30-39% of body surface
T31.400	Burns involving 40%~49% of body surface
T31.400x001	40-49% burn on body surface
T31.500	Burns involving 50%~59% of body surface
T31.500x001	Burns on body surface 50-59%
T31.600	Burns involving 60%~69% of body surface
T31.600x001	Burns on body surface 60-69%
T31.700	Burns involving 70%~79% of body surface
T31.700x001	70-79% burn on body surface
T31.800	Burns involving 80%~89% of body surface

T31.800x001	80-89% of body surface burns
T31.900	Burns involving 90% or more of the body surface
T31.900x001	Burns greater than 90% of the body surface
T32.000	Corrosion damage involving less than 10% of body surface
T32.000x001	Corrosion damage on body surface less than 10%
T32.100	Corrosion injury involving 10%~19% of body surface
T32.100x001	10-19% of the body surface is corroded
T32.200	Corrosion damage involving 20%~29% of body surface
T32.200x001	20-29% of the body surface is corroded
T32.300	Corrosion damage involving 30%~39% of body surface
T32.300x001	30-39% of body surface corrosion
T32.400	Corrosion damage involving 40%~49% of body surface
T32.400x001	40-49% of the body surface is corroded
T32.500	Corrosion damage involving 50%~59% of body surface
T32.500x001	50-59% corrosion damage on body surface
T32.600	Corrosion damage involving 60%~69% of body surface
T32.600x001	60-69% of the body surface is corroded
T32.700	Corrosion damage involving 70%~79% of body surface
T32.700x001	70-79% of the body surface is corroded
T32.800	80%~89% corrosion damage on body surface
T32.800x001	80-89% of the body surface is corroded
T32.900	Corrosion damage involving 90% or more of the body surface

T32.900x001	Corrosion damage greater than 90% on the body surface
T33.000	Superficial frostbite of head
T33.100	Superficial frostbite of neck
T33.200	Superficial frostbite of chest
T33.300	Superficial frostbite of abdominal wall, lower back and pelvis
T33.300x001	Superficial frostbite of abdominal wall
T33.300x002	Superficial frostbite of back
T33.300x003	Pelvic superficial frostbite
T33.400	Superficial frostbite of arm
T33.500	Superficial frostbite of wrist and hand
T33.500x002	Superficial frostbite of wrist
T33.500x003	Superficial frostbite of hands
T33.600	Superficial frostbite of hip and thigh
T33.600x002	Superficial frostbite of hip
T33.600x003	Superficial frostbite of thigh
T33.700	Superficial frostbite of knee and lower leg
T33.700x002	Superficial frostbite of knee
T33.700x003	Superficial frostbite of lower leg
T33.800	Superficial frostbite of ankle and foot
T33.800x002	Superficial frostbite of ankle
T33.800x003	Superficial frostbite of foot
T33.900	Superficial frostbite, other and unspecified
T33.900x003	Superficial frostbite of trunk
T33.901	Superficial frostbite
T34.000	Head frostbite with tissue necrosis
T34.100	Cervical frostbite with tissue necrosis
T34.200	Chest frostbite with tissue necrosis

T34.300	Frostbite of abdominal wall, lower back and pelvis with tissue necrosis
T34.300x001	Frostbite of abdominal wall with tissue necrosis
T34.300x002	Frostbite of back with tissue necrosis
T34.300x003	Pelvic frostbite with tissue necrosis
T34.400	Frostbite of arm with tissue necrosis
T34.500	Frostbite of wrist and hand with tissue necrosis
T34.500x002	Frostbite of wrist with tissue necrosis
T34.500x003	Hand frostbite with tissue necrosis
T34.600	Frostbite of hip and thigh with tissue necrosis
T34.600x002	Hip frostbite with tissue necrosis
T34.600x003	Frostbite of thigh with tissue necrosis
T34.700	Frostbite of knee and lower leg with tissue necrosis
T34.700x002	Frostbite of knee with tissue necrosis
T34.700x003	Frostbite of lower leg with tissue necrosis
T34.800	Frostbite of ankle and foot with tissue necrosis
T34.800x002	Frostbite of ankle with tissue necrosis
T34.800x003	Frostbite of foot with tissue necrosis
T34.900	Frostbite with tissue necrosis, other and unspecified
T34.900x002	Frostbite with tissue necrosis
T34.900x003	Frostbite of trunk with tissue necrosis
T35.000	Superficial frostbite involving multiple parts of the body
T35.000x001	Multiple superficial frostbite
T35.100	Frostbite involving multiple parts of the body with tissue necrosis
T35.100x001	Multiple frostbite with tissue necrosis

T35.200	Frostbite of head and neck
T35.300	Frostbite of chest, abdomen, lower back and pelvis
T35.300x001	Frostbite of trunk
T35.300x002	Chest frostbite
T35.300x003	Abdominal frostbite
T35.300x004	Back frostbite
T35.300x005	Pelvic frostbite
T35.300x006	Frostbite of abdominal wall, lower back and pelvis
T35.400	Frostbite of upper limbs
T35.500	Frostbite of lower limbs
T35.600	Frostbite involving multiple parts of the body
T35.600x001	Multiple frostbite
T35.700	frostbite
T35.700x002	Cold injury
T35.700x003	Occupational frostbite
T35.700x004	Systemic cold injury
T35.700x005	Local frostbite
T35.700x006	Local once frostbite
T35.700x007	Local secondary frostbite
T35.700x008	Partial third degree frostbite
T35.700x009	Partial fourth degree frostbite
T36.000	Penicillins Poisoning
T36.100	Cephalosporins and others β Poisoning by lactam antibiotics
T36.100x003	β Poisoning by lactam antibiotics
T36.101	Poisoning by cephalosporins
T36.102	Poisoning by cephalosporin

T36.200	Chloramphenicol poisoning
T36.300	Macrolide poisoning
T36.300x001	Erythromycin poisoning
T36.400	Tetracycline poisoning
T36.500	Poisoning by aminoglycosides
T36.500x003	Streptomycin poisoning
T36.501	Poisoning by amikacin
T36.502	Gentamicin poisoning
T36.600	Rifamycin poisoning
T36.700	Systemic antifungal antibiotic poisoning
T36.800	Systemic antibiotic poisoning, others
T36.900	Systemic antibiotic poisoning
T36.900x001	Antibiotic poisoning
T37.000	Sulfonamide poisoning
T37.100	Poisoning by antimycobacterial drugs
T37.100x001	Poisoning by ramifene
T37.200	Antimalarial and drug poisoning with effect on other blood protozoa
T37.300	Antigen worm drug poisoning, others
T37.300x001	Poisoning by antigen living substance
T37.400	Poisoning by worm repellent
T37.500	Poisoning by antiviral drugs
T37.800	Poisoning by systemic anti infective and antiparasitic drugs, other specific
T37.800x001	Poisoning by hydroxyquinoline derivative
T37.900	Systemic anti infective and antiparasitic poisoning
T37.900x001	Systemic anti infective poisoning
T37.900x002	Systemic antiparasitic poisoning

T38.000	Poisoning by sugar [adrenal] corticosteroids and their synthetic analogues
T38.000x001	Iatrogenic steroid diabetes
T38.100	Poisoning by thyroid hormones and their substitutes
T38.100x001	Poisoning by thyroid hormone and its derivatives
T38.200	Poisoning by antithyroid drugs
T38.300	Insulin and oral hypoglycemia [anti diabetes] drug poisoning
T38.300x001	Iatrogenic hyperinsulinemia
T38.300x003	Poisoning by oral antidiabetic drugs
T38.301	Insulin poisoning
T38.400	Poisoning by oral contraceptives
T38.401	Gossypol poisoning
T38.500	Estrogen and progesterone poisoning, others
T38.500x001	Estrogen poisoning
T38.500x002	Progesterone poisoning
T38.501	Diethylstilbestrol poisoning
T38.600	Poisoning caused by anti gonadotropins, anti estrogen drugs and anti androgen drugs, which cannot be classified elsewhere
T38.600x001	Tamoxifen poisoning
T38.700	Poisoning by androgens and similar drugs that promote metabolism
T38.800	Poisoning by hormones and their synthetic substitutes, other and unspecified
T38.800x001	Poisoning of anterior pituitary hormones
T38.801	Poisoning by hormones and their synthetic substitutes
T38.900	Poisoning by hormone antagonists, other and unspecified

T38.901	Poisoning by hormone antagonists
T39.000	Poisoning by salicylate
T39.100	Poisoning by 4-aminophenol derivative
T39.101	Poisoning by acetaminophen
T39.200	Poisoning by pyrazolone derivative
T39.200x001	Poisoning by metamizole
T39.201	Poisoning by aminopyrine
T39.300	Poisoning by non steroid anti-inflammatory drugs [NSAID]
T39.300x002	Poisoning by tramadol
T39.300x003	Indomethacin poisoning
T39.400	Poisoned by anti rheumatic drugs, which cannot be classified elsewhere
T39.400x001	Poisoning by antirheumatic drugs
T39.800	Poisoning by non opioid analgesics and antipyretics, others, which cannot be classified elsewhere
T39.801	Poisoning by Tongkening
T39.802	Poisoning by the root of kidney bean
T39.900	Poisoning by non opioid analgesics, antipyretics and anti rheumatic drugs
T39.901	Poisoning by non opioid analgesics
T39.902	Antipyretic poisoning
T40.000	Opioid poisoning
T40.100	Heroin poisoning
T40.200	Opioid poisoning, other
T40.200x001	Codeine poisoning
T40.201	Morphine poisoning
T40.300	Poisoning by Meisantong
T40.400	Poisoning by synthetic narcotics, others
T40.400x002	Aristolochia (ten thousand zhang long) poisoning

T40.401	Poisoning by dolantine
T40.500	Cocaine poisoning
T40.600	Poisoning by narcotics, others
T40.601	Poisoning by narcotics
T40.700	Cannabis (derivative) poisoning
T40.700x001	cannabis intoxication
T40.800	Diethyllysergamide [LSD] poisoning
T40.900	Hallucinogen [Hallucinogen] poisoning, other and unspecified
T40.900x001	Poisoning by poisonous alkali of South American cactus
T40.900x002	Poisoning by dimethyl-4-hydroxytryptamine
T40.900x003	Cilosibine poisoning
T40.901	Hallucinogenic drug poisoning
T41.000	Inhalation anesthetic poisoning
T41.100	Poisoning by intravenous anesthetics
T41.100x002	Poisoning by thiobarbital salts
T41.200	Poisoning by general anesthetics, other and unspecified
T41.200x002	Poisoning by ketamine
T41.201	Poisoning by general anesthetic
T41.300	Toxicosis of local Anaesthetic
T41.400	Poisoning by narcotics
T41.500	Therapeutic gas poisoning
T41.500x001	Therapeutic oxygen poisoning
T41.500x003	Therapeutic carbon dioxide poisoning
T41.501	Oxygen poisoning, accident
T42.000	Poisoning by hydantoin derivative
T42.001	Poisoning by phenytoin sodium

T42.100	Poisoning by iminodistyrene
T42.101	Poisoning by carbamazepine
T42.200	Poisoning by succinimide and oxazolidinedione
T42.200x001	Oxazolidine copper poisoning
T42.200x002	Poisoning by succinimide
T42.300	Poisoning by barbiturate salts
T42.301	Poisoning by phenobarbital
T42.302	Tachysomia poisoning
T42.400	Benzenediazepines poisoning
T42.401	Diazepam poisoning
T42.402	Poisoning by Jiajing Diazepam
T42.403	Poisoning by salbutamol
T42.404	Poisoning by Limianning
T42.405	Nitrazepam poisoning
T42.406	Poisoning by clozapine
T42.500	Poisoning by mixed antiepileptic drugs, which cannot be classified elsewhere
T42.500x001	Mixed antiepileptic drug poisoning
T42.600	Poisoning by antiepileptic drugs and sedative hypnotics, others
T42.600x002	Zopiclone poisoning
T42.600x004	Poisoning by sodium valproate
T42.600x005	Valproic acid poisoning
T42.600x006	Poisoning by phenethylpiperidone
T42.601	Poisoning by methaqualone
T42.602	Poisoning by phenanthrol
T42.700	Poisoning by antiepileptic drugs and sedative hypnotics
T42.700x001	Hypnotic poisoning

T42.700x003	Poisoning by tranquilizers
T42.701	poisoning of sedativehypnotic drugs
T42.702	Poisoning by sleeping pills
T42.800	Poisoning by antiparkinsonian drugs and other central nervous system muscle tone inhibitors
T42.800x001	Poisoning by antiparatic drugs
T42.800x002	Poisoning by central nervous system muscle tension inhibitor
T42.800x003	Amantadine poisoning
T43.000	Poisoning by tricyclic and tetracyclic antidepressants
T43.000x002	Poisoning by tricyclic antidepressant
T43.000x003	Poisoning by tetracycline antidepressant
T43.001	Poisoning by amitriptyline
T43.002	Poisoning by doxepin
T43.100	Monoamine oxidase inhibitor antidepressant poisoning
T43.200	Antidepressant poisoning, other and unspecified
T43.200x001	Poisoning by fluoxetine
T43.201	Poisoning by antidepressants
T43.300	Poisoning by phenthiazide antipsychotics and antipsychotics
T43.300x001	Perphenazine poisoning
T43.300x003	Poisoning by phenol thiazide type antipsychotic drugs
T43.301	Fenergan poisoning
T43.302	Poisoning by chlorpromazine
T43.400	Poisoning by butylbenzene and thioanthracene
T43.400x002	Butyryl benzene poisoning
T43.400x003	Poisoning by thioanthracene antipsychotic

T43.401	Poisoning by haloperidol
T43.500	Poisoning by antipsychotics and tranquilizers, other and unspecified
T43.500x001	Tranquility poisoning
T43.500x002	Poisoning by quetiapine fumarate
T43.500x003	Lithium carbonate poisoning
T43.500x004	Poisoning by antipsychotics
T43.500x005	Poisoning by pentafluridol
T43.501	Poisoning by antipsychotics and antipsychotics
T43.502	Poisoning by Miaoltong
T43.600	Mental stimulant poisoning with abuse potential
T43.600x003	Methamphetamine poisoning
T43.600x004	Ecstasy poisoning
T43.601	Caffeine poisoning
T43.800	Poisoning caused by drugs that have an impact on the spirit, and others that cannot be classified elsewhere
T43.900	Poisoning by drugs with mental effects
T44.000	Poisoning by anticholinesterase agent
T44.001	Poisoning by pyrimethamine
T44.100	Parasympathetic drugs [cholinergic drugs] poisoning, others
T44.100x001	Parasympathetic intoxication
T44.200	Poisoned by ganglion blockers, which cannot be classified elsewhere
T44.200x001	Poisoning by ganglion blockers
T44.300	Parasympathetic depressants [anticholinergics and anti muscarinic drugs] those poisoned by antispasmodics, others, which cannot be classified elsewhere
T44.301	Atropine poisoning
T44.302	Anisodamine poisoning

T44.303	Poisoning by artan
T44.400	Mainly α Poisoned by a highly effective adrenergic receptor drug, which cannot be classified elsewhere
T44.400x001	α Poisoning by highly effective adrenergic receptor drugs
T44.400x002	Alamin poisoning
T44.500	Mainly β Poisoned by a highly effective adrenergic receptor drug, which cannot be classified elsewhere
T44.500x001	β Poisoning by highly effective adrenergic receptor drugs
T44.600	α Poisoning by adrenergic receptor antagonist, which cannot be classified elsewhere
T44.600x001	α Poisoning by adrenergic receptor antagonist
T44.700	β Poisoning by adrenergic receptor antagonist, which cannot be classified elsewhere
T44.700x001	β Poisoning by adrenergic receptor antagonist
T44.701	Poisoning by propranolol
T44.800	Central effect and adrenergic neuron blocker poisoning, which cannot be classified elsewhere
T44.800x001	Central effects and adrenergic neuron blocker poisoning
T44.900	Other and unspecified drug poisoning mainly affecting the autonomic nervous system
T44.900x001	α and β Poisoning by adrenergic receptor drugs
T44.900x002	Ephedrine poisoning
T44.900x003	Poisoning by angiotensin receptor inhibitor
T44.901	Drug poisoning mainly affecting autonomic nervous system
T45.000	Poisoning by antiallergic drugs and antiemetics

T45.000x001	Poisoning by antiallergic drugs
T45.001	Poisoning by antiemetic
T45.002	Metoclopramide poisoning
T45.003	Poisoning by Chenghuining
T45.100	Poisoning by antineoplastic drugs and immunosuppressants
T45.100x001	Antineoplastic antibiotic poisoning
T45.100x002	Poisoning by cytarabine
T45.100x003	Cyclosporine poisoning
T45.100x004	Hypermethotrexaemia
T45.101	Poisoning by methotrexate
T45.102	Poisoning by vincristine
T45.200	Vitamin poisoning, which cannot be classified elsewhere
T45.200x001	Vitamin poisoning
T45.201	Poisoning by vitamin A
T45.202	Vitamin D poisoning
T45.300	Enzyme poisoning, which cannot be classified elsewhere
T45.300x001	Enzyme poisoning
T45.400	Poisoning by iron and its compounds
T45.500	Anticoagulant poisoning
T45.500x002	Poisoning by anticoagulants
T45.501	Poisoning by new anticoagulant tablets
T45.600	Affect the poisoning of fibrinolytic drugs
T45.700	Poisoning by anticoagulant antagonists, vitamin K and other clotting drugs
T45.700x001	Poisoning by anticoagulant antagonist
T45.700x002	Poisoning by vitamin K
T45.700x003	Coagulant poisoning

T45.800	Poisoning, others are mainly systemic and hematological preparations
T45.800x001	Natural blood poisoning
T45.800x002	Poisoning of blood products
T45.800x003	Plasma substitute poisoning
T45.900	Mainly systemic and hematological agent poisoning
T46.000	Poisoning by cardiac excitatory glycosides and similar drugs
T46.001	Digoxin poisoning
T46.002	Digitalis poisoning
T46.100	Poisoning by calcium channel blockers
T46.100x001	Poisoning by verapamil
T46.200	Poisoning by antiarrhythmic drugs, others, which cannot be classified elsewhere
T46.200x001	Poisoning by antiarrhythmic drugs
T46.300	Coronary vasodilator poisoning, which cannot be classified elsewhere
T46.300x002	Poisoning by coronary vasodilator
T46.300x003	Poisoning by dipyridamole
T46.301	Nitroglycerin poisoning
T46.302	Imdor poisoning
T46.400	Poisoning by angiotensin-converting enzyme inhibitor
T46.500	Poisoned by antihypertensive drugs, others, not to be classified elsewhere
T46.500x002	Poisoning by guanethidine
T46.500x003	Poisoning by Rauwolfia
T46.500x004	Poisoning by reserpine
T46.500x005	Poisoning by antihypertensive drugs
T46.501	Poisoning by clonidine
T46.600	Poisoning by anti hyperlipidemia and anti atherosclerosis drugs
T46.600x001	Poisoning by anti atherosclerosis drugs
T46.700	Poisoning by peripheral vasodilator
T46.700x001	Poisoning by compound hypotensive tablets
T46.700x002	Nicotinic acid poisoning
T46.800	Poisoning by anti varicose drugs (including sclerosing agents)
T46.800x001	Poisoning by anti varicose drugs
T46.900	Poisoning by other agents mainly affecting cardiovascular system
T46.900x001	Aconitine poisoning
T46.901	Poisoning of preparations mainly affecting cardiovascular system
T47.000	Poisoning by histamine H2 receptor antagonist
T47.100	Poisoning by antacids and anti gastric secreting drugs, others
T47.100x001	Poisoning by antacids and anti gastric secreting drugs
T47.200	Irritating laxative poisoning
T47.200x002	Aloe poisoning
T47.300	Poisoning by saline and osmotic laxative
T47.300x001	Pervious laxative poisoning
T47.400	Poisoning by laxative, others
T47.400x001	Poisoning by intestinal relaxant
T47.500	Digestive drug poisoning
T47.600	Poisoning by antidiarrheal
T47.700	Poisoning by emetics
T47.800	Poisoning by other agents mainly affecting gastrointestinal system
T47.900	Poisoning of preparations mainly affecting gastrointestinal system
T48.000	Poisoning by oxytocin

T48.100	Skeletal muscle relaxant [neuromuscular blocker] poisoning
T48.100x001	Poisoning by skeletal muscle relaxant
T48.200	Poisoning of other and unspecified preparations mainly acting on muscles
T48.201	Poisoning by acting on muscle preparations
T48.300	Poisoning by antitussive
T48.400	Poisoning by expectorants
T48.500	Poisoning by anti cold drugs
T48.600	Poisoned by anti asthma drugs, which cannot be classified elsewhere
T48.600x002	Datura poisoning
T48.600x003	Poisoning by salbutamol
T48.601	Poisoning by aminophylline
T48.602	Poisoning by amlodipine
T48.603	Poisoning by clenbuterol
T48.700	Poisoning of other and unspecified preparations mainly acting on respiratory system
T48.701	Poisoning by respiratory system agents
T49.000	Local antifungal, anti infective and anti-inflammatory drug poisoning, which cannot be classified elsewhere
T49.000x003	Iodine intoxication
T49.000x005	Poisoning by bromogeramine
T49.001	Cresol poisoning
T49.002	Poisoning by mercurous chloride
T49.003	Lysus poisoning
T49.100	Poisoning by antipruritic drugs
T49.200	Poisoning by local astringent and local detergent
T49.200x001	Poisoning by local astringent

T49.201	Local detergent poisoning
T49.300	Poisoning by lubricant, palliative and protective drugs
T49.300x001	Lubricant poisoning
T49.300x002	Poisoning by moderator
T49.300x003	Poisoning by gastric mucosal protective agent
T49.400	Poisoning by cuticle separating agents, cuticle hyperplasia agents and other drugs and preparations for hair treatment
T49.400x001	Poisoning by cuticle separating drug
T49.400x002	Poisoning by cuticle hyperplasia drugs
T49.400x003	Poisoning of drugs and preparations for hair treatment
T49.500	Ophthalmic drugs and preparations poisoning
T49.600	Poisoning by otorhinolaryngological drugs and preparations
T49.700	Dental drug poisoning, topical
T49.700x001	Local drug poisoning in stomatology department
T49.800	Poisoning by local preparation, others
T49.800x001	Poisoning by spermicide
T49.801	Cosmetic poisoning
T49.900	Poisoning by local preparation
T50.000	Poisoning by halocorticoids and their antagonists
T50.000x001	Poisoning by mineralocorticoids and their antagonists
T50.100	Diuretic poisoning
T50.100x001	Loop diuretic poisoning
T50.200	Poisoning by carbonic acid dehydratase inhibitors, benzothiadiazines and other diuretics

T50.200x001	Poisoning by acetamide
T50.200x002	Mercury diuretic poisoning
T50.300	Poisoning by electrolyte, heat and water balance agent
T50.300x001	Poisoning by potassium chloride
T50.300x002	Oral rehydration salt poisoning
T50.400	Drug poisoning affecting uric acid metabolism
T50.400x001	Poisoning by uric acid metabolite
T50.500	Poisoning by appetite inhibitor
T50.600	Poisoned by antidote and chelating agent, which cannot be classified elsewhere
T50.600x001	Poisoning by antialcoholic drugs
T50.600x002	Poisoning by antidote
T50.600x003	Chelating agent poisoning
T50.700	Poisoning by stimulants and opioid receptor antagonists
T50.700x001	Excitator poisoning
T50.700x002	Poisoning by opioid receptor antagonist
T50.800	Diagnostic agent poisoning
T50.900	Poisoning by drugs, pharmaceuticals and biological products, other and unspecified
T50.900x001	Drug poisoning
T50.900x002	Acidifier poisoning
T50.900x003	Alkaline poisoning
T50.900x004	Immunoglobulin poisoning
T50.900x005	Poisoning by immune agents
T50.900x006	Lipid regulating drug poisoning
T50.900x007	Poisoning by parathyroid hormones
T51.000	Toxic effect of ethanol

T51.000x001	Alcoholism
T51.001	alcoholism
T51.100	Toxic effect of methanol
T51.100x001	Methanol poisoning
T51.200	Toxic effect of 2-propanol
T51.200x001	Isopropyl alcohol poisoning
T51.300	Toxic effect of fusel oil
T51.300x001	Fusel oil poisoning
T51.300x002	Poisoning by amyl alcohol
T51.300x003	Butyl alcohol poisoning
T51.300x004	Poisoning by propyl alcohol
T51.800	Toxic effects of alcohols, others
T51.800x001	Poisoning by sodium trichloropyridinol
T51.900	Toxic effect of alcohol
T51.900x001	Alcoholism
T52.000	Toxic effects of petroleum products
T52.000x001	Poisoning by petroleum products
T52.000x002	Naphtha poisoning
T52.000x003	Kerosene poisoning
T52.000x004	Gasoline poisoning
T52.000x005	Ether poisoning
T52.000x006	Poisoning by petroleum essence
T52.100	Toxic effects of benzene
T52.101	Benzene poisoning
T52.200	Toxic effects of benzene analogues
T52.200x001	Poisoning by benzene compounds
T52.200x002	Toluene poisoning
T52.200x003	Xylene poisoning
T52.300	Toxic effects of aliphatic diols

T52.300x001	Aliphatic diols poisoning
T52.400	Toxic effects of ketones
T52.400x001	Ketosis
T52.800	Toxic effects of other organic solvents
T52.800x001	Dimethyl Formamide Poisoning
T52.800x002	Poisoning by formaldehyde aqueous solution
T52.800x003	Poisoning by dichloroethane
T52.800x004	n-hexane poisoning
T52.800x005	Banana water poisoning
T52.800x006	Poisoning by dimethyl sulfate
T52.900	Toxic effects of organic solvents
T52.900x001	Poisoning by organic solvent
T53.000	Toxic effect of carbon tetrachloride
T53.000x001	Carbon tetrachloride poisoning
T53.000x002	Tetrachloromethane poisoning
T53.100	Toxic effect of chloroform
T53.100x001	Chloroform poisoning
T53.100x002	Poisoning by chloroform
T53.200	Toxic effect of trichloroethylene
T53.200x001	Poisoning by trichloroethylene
T53.200x002	Poisoning by trichloroethane
T53.300	Toxic effect of tetrachloroethylene
T53.300x001	Perchloroethylene poisoning
T53.300x002	Tetrachloroethylene poisoning
T53.400	Toxic effect of dichloromethane
T53.400x001	Dichloromethane poisoning
T53.500	Toxic effects of chlorofluorocarbons
T53.500x001	Poisoning by chlorofluorocarbons

T53.600	Toxic Effects of Other Halogen Derivatives of Alicyclic Hydrocarbons
T53.600x001	Poisoning by vinyl chloride
T53.600x002	Poisoning by trichloropropane
T53.600x003	Poisoning by chloroprene
T53.600x004	Poisoning by allyl chloride
T53.700	Toxic Effects of Other Halogen Derivatives of Aromatic Hydrocarbons
T53.700x001	Poisoning by chlorophenol
T53.900	Toxic Effects of Halogen Derivatives of Alicyclic Hydrocarbons and Aromatic Hydrocarbons
T54.000	Toxic effects of phenol and its analogues
T54.000x001	Poisoning by phenol and its analogues
T54.000x002	Phenol poisoning
T54.100	Toxic effects of corrosive organic compounds, others
T54.200	Toxic effects of corrosive acids and acid like substances
T54.200x001	Sulfuric acid chemical injury
T54.200x002	Nitrite chemical injury
T54.200x003	Hydrochloric acid chemical injury
T54.200x004	Acid chemical injury
T54.201	Sulfuric acid poisoning
T54.202	Acid poisoning
T54.203	Nitrite poisoning
T54.300	Toxic effects of corrosive alkali and alkali like substances
T54.300x002	Potassium hydroxide chemical injury
T54.300x003	Sodium hydroxide chemical injury
T54.301	Caustic alkali poisoning
T54.900	Toxic effects of corrosive substances

T54.900x001	Chemical injury caused by corrosive substances
T54.900x002	Brine poisoning
T55.x00	Toxic effects of soaps and detergents
T55.x00x001	Soap poisoning
T55.x00x002	Detergent poisoning
T55.x00x003	Detergent poisoning
T56.000	Toxic effects of lead and its compounds
T56.000x002	Poisoning by lead compounds
T56.000x003	Tetraethyl lead poisoning
T56.001	lead poisoning
T56.100	Toxic effects of mercury and its compounds
T56.100x002	Poisoning by mercury compounds
T56.101	Mercury poisoning
T56.200	Toxic effects of chromium and its compounds
T56.200x001	Chromium poisoning
T56.200x002	Poisoning by chromium compounds
T56.300	Toxic effects of cadmium and its compounds
T56.300x001	Cadmium poisoning
T56.300x002	Poisoning by cadmium compounds
T56.400	Toxic effects of copper and its compounds
T56.400x002	Poisoning by copper compounds
T56.401	Copper poisoning
T56.500	Toxic effects of zinc and its compounds
T56.500x001	Zinc poisoning
T56.500x002	Poisoning by zinc compounds
T56.600	Toxic effects of tin and its compounds

T56.600x001	Tin poisoning
T56.600x002	Poisoning by tin compounds
T56.700	Toxic effects of beryllium and its compounds
T56.700x002	Poisoning by beryllium compound
T56.800	Toxic effects of metals, others
T56.800x001	Vanadium poisoning
T56.800x002	Poisoning by vanadium compounds
T56.800x003	Uranium poisoning
T56.800x004	Poisoning by uranium compounds
T56.800x005	nickel car-bonyI poisoning
T56.800x006	Indium poisoning
T56.800x007	Indium compound poisoning
T56.801	Thallium poisoning
T56.900	Toxic effects of metals
T56.900x001	Metal poisoning
T56.900x002	Metal fume heat
T56.900x003	Metal vapor poisoning
T57.000	Toxic effects of arsenic and its compounds
T57.000x001	Arsenism
T57.000x002	Arsenic poisoning
T57.000x003	Arsine poisoning
T57.001	arsenic poisoning
T57.100	Toxic effects of phosphorus and its compounds
T57.100x001	Phosphorus poisoning
T57.100x002	Poisoning by phosphorus compounds
T57.100x003	Phosphine poisoning
T57.100x004	Zinc phosphide poisoning

T57.100x005	Aluminum phosphide poisoning
T57.200	Toxic effects of manganese and its compounds
T57.200x001	Manganese poisoning
T57.200x002	Poisoning by manganese compounds
T57.201	Manganide poisoning
T57.300	Toxic effect of hydrogen cyanide
T57.300x001	Hydrogen cyanide poisoning
T57.800	Toxic effects of inorganic substances, other specific
T57.800x002	Poisoning by barium compound
T57.800x003	Barium poisoning
T57.900	Toxic effects of inorganic substances
T58.x00	Toxic effects of carbon monoxide
T58.x00x001	carbon monoxide poisoning
T59.000	Toxic effects of nitrogen oxides
T59.000x001	Nitrogen poisoning
T59.000x002	Nitrogen oxide poisoning
T59.100	Toxic effect of sulfur dioxide
T59.100x001	Sulfur dioxide poisoning
T59.101	Smoke poisoning
T59.200	Toxic effect of formaldehyde
T59.200x001	Formaldehyde poisoning
T59.300	Toxic effects of tear gas
T59.300x001	Tear gas poisoning
T59.400	Toxic effect of chlorine
T59.401	Chlorine poisoning
T59.500	Toxic effects of fluorine gas and hydrogen fluoride
T59.500x001	Fluorine poisoning

T59.500x002	Fluoride poisoning
T59.600	Toxic effect of hydrogen sulfide
T59.601	Hydrogen sulfide poisoning
T59.700	Toxic effects of carbon dioxide
T59.700x001	Carbon dioxide poisoning
T59.800	Toxic effects of gases, smoke and vapours, other specific
T59.800x001	Biogas poisoning
T59.800x002	LPG poisoning
T59.800x004	Natural gas poisoning
T59.800x005	Methyl bromide poisoning
T59.800x006	Mustard gas poisoning
T59.800x007	Phosgene poisoning
T59.800x008	Monomethylamine Poisoning
T59.800x009	Bromopropane poisoning
T59.800x010	Ethylene oxide poisoning
T59.801	Ammonia poisoning
T59.802	Liquefied gas poisoning
T59.803	Total hydrocarbon oil vapor poisoning
T59.900	Toxic effects of gases, fumes and vapours
T59.900x001	Irritating gas poisoning
T59.900x002	Poisoning by aerosol
T59.900x003	Volatile poisoning
T59.900x004	Mixed gas poisoning
T59.900x005	Asphyxiating gas poisoning
T60.000	Toxic effects of organophosphate and carbamate insecticides
T60.000x003	Phoxim poisoning
T60.000x004	Carbamate insecticide poisoning

T60.001	Organophosphorus poisoning
T60.002	Dichlorvos poisoning
T60.100	Toxic effects of halogenated pesticides
T60.100x001	Halogenated insecticide poisoning
T60.101	Deltamethrin poisoning
T60.200	Toxic effects of pesticides, others
T60.200x001	Poisoning by cockroach killing drug
T60.300	Toxic effects of herbicides and fungicides
T60.300x001	Poisoning by herbicide
T60.300x002	Fungicide poisoning
T60.300x003	Poisoning by chloroacetic acid
T60.400	Toxic effect of rodenticide
T60.400x003	Thallium poisoning
T60.401	Poisoning by rodenticide
T60.800	Toxic effects of crop pesticides, others
T60.900	Toxic effects of pesticides
T60.900x001	Pesticide poisoning
T60.900x002	pesticide poisoning
T60.900x003	Poisoning by wood preservatives
T61.000	Fish poisoning
T61.001	Fish gall poisoning
T61.100	Poisoning by mackerel
T61.100x002	Histamine like syndrome
T61.200	Poisoning of fish and aquatic shellfish, others
T61.200x001	Fish poisoning
T61.200x003	Shellfish poisoning
T61.201	Globefish poisoning

T61.800	Toxic effects of marine products, others
T61.900	Toxic effects of marine products
T61.900x001	Poisoning of seafood
T62.000	Toxic effects of mushroom intake
T62.000x001	Mushroom poisoning
T62.000x002	Mushroom poisoning
T62.001	Poisoned by eating poisonous mushrooms
T62.002	Boletus poisoning
T62.100	Toxic effects of intake of berries
T62.100x001	Berry poisoning
T62.200	Toxic effects of ingestion of other plants (or parts of plants)
T62.200x002	Poisoning by castor bean
T62.200x003	Botanical poisoning
T62.202	Solanum fruit poisoning
T62.800	Toxic effects of other specifically harmful substances in food intake
T62.800x002	Nitrite poisoning
T62.801	Poisoning by bitter almond
T62.802	Lentil poisoning
T62.900	Toxic effects of harmful substances in food intake
T62.900x002	food poisoning
T63.000	Toxic effect of snake venom
T63.000x001	Poisoning by snake venom
T63.001	Poisonous snake bite
T63.100	Toxic effects of reptile venom, others
T63.100x001	Poisoning by lizard venom
T63.200	Toxic effect of scorpion venom

T63.200x001	Poisoning by scorpion venom
T63.300	Toxic effect of spider venom
T63.300x001	Spider venom poisoning
T63.400	Toxic effects of arthropod venom, others
T63.400x002	Poisonous insect bites
T63.400x003	Poisonous insect sting
T63.400x004	Sandfly Bite
T63.401	Bee sting
T63.402	Arthropod bites
T63.500	Toxic effects in contact with fish
T63.500x001	Poisoning after contact with fish
T63.600	Toxic effects of contact with other marine animals
T63.600x001	Poisoning after contact with jellyfish
T63.600x002	Poisoned after contacting sea anemone
T63.600x003	Poisoning after contact with aquatic shellfish
T63.600x004	Poisoning after contact with marine animals
T63.600x005	Poisoned after contacting starfish
T63.800	Toxic effects in contact with other toxic animals
T63.800x001	Amphibian venom poisoning
T63.900	Toxic effects in contact with toxic animals
T64.x00	Toxic effects of aflatoxin and other mycotoxins on food
T64.x00x001	Aflatoxin poisoning
T64.x00x002	Toxic effect of mycotoxin on food
T64.x01	Toxic effects of other mycotoxins on food

T64.x02	Toxic effect of aflatoxin on food
T65.000	Toxic effect of cyanide
T65.000x001	Cyanide poisoning
T65.100	Toxic effect of Shi Di Nian and its salts
T65.100x001	Poisoning by strychnine and its salts
T65.200	Toxic effects of tobacco and nicotine
T65.200x001	Tobacco poisoning
T65.200x002	Nicotine poisoning
T65.300	Toxic effects of nitrogen derivatives and amine derivatives of benzene and its analogues
T65.300x001	Aniline poisoning
T65.300x002	Nitrobenzene poisoning
T65.300x003	Poisoning by trinitrotoluene
T65.300x004	Poisoning by nitro compounds
T65.400	Toxic effect of carbon disulfide
T65.400x001	Carbon disulfide poisoning
T65.500	Toxic effects of nitroglycerin and other nitric acids and esters
T65.500x001	Nitroglycerol poisoning
T65.500x002	Poisoning by trinitroglycerin
T65.501	Specifically refers to the toxic effects of nitric acid and esters
T65.600	Toxic effects of paints and dyes, which cannot be classified elsewhere
T65.600x001	Varnish poisoning
T65.600x002	Paint poisoning
T65.600x003	Dye poisoning
T65.800	Toxic effects of substances, others specifically
T65.800x002	Poisoning by hair dye

T65.800x003	Poisoning by potassium permanganate
T65.800x004	Dimethylhydrazine Poisoning
T65.800x005	Poisoning by disinfectant
T65.800x006	Acrylamide poisoning
T65.800x007	Methyl iodide poisoning
T65.801	Humidifier disinfectant poisoning
T65.900	Toxic effects of substances
T65.900x001	Antifreeze poisoning
T65.901†	Toxic dementia
T66.x00	Effect of radiation
T66.x00x001	Radiation sickness
T66.x00x002	Occupational radiation sickness
T66.x01	Radiation damage
T66.x02	Radiation optic nerve damage
T67.000	Heat stroke and sunstroke
T67.000x001	Heat apoplexy
T67.000x002	Heat stroke
T67.001	Thermal fever
T67.002	Sunstroke
T67.100	Febrile syncope
T67.100x002	Thermal collapse
T67.200	Heat cramp
T67.300	Dehydration heat exhaustion
T67.300x001	Heatstroke dehydration
T67.300x002	Dehydrative heat exhaustion
T67.400	Heat exhaustion caused by salt deficiency
T67.400x001	Heat exhaustion due to salt deficiency
T67.500	Heat exhaustion

T67.500x001	Heat exhaustion
T67.600	Transient heat exhaustion
T67.700	Heatstroke edema
T67.800	Other effects of heat and light
T67.900	Effect of heat and light
T67.901	Heat stroke
T68.x00	Hypothermia
T68.x00x002	Accidental hypothermia
T69.000	Soak hands and feet
T69.000x001	Soak hands
T69.000x002	Soaking foot
T69.000x003	Trench foot
T69.000x004	Hunting reaction
T69.100	chilblains
T69.100x002	Chilblain of auricle
T69.100x003	Chilblain of foot
T69.100x004	Facial chilblain
T69.100x005	Chilblain of hand
T69.800	Other specific effects of cooling
T69.800x001	Chap
T69.800x002	Chapped hand
T69.800x003	Chapped foot
T69.900	Cooling effect
T70.000	Aerootitis media
T70.100	Aviation sinusitis
T70.200	Other and unspecified effects of high altitude
T70.200x005	Barotrauma
T70.200x006	Alpine disease

T70.200x007	High altitude heart disease
T70.201	High altitude pulmonary edema
T70.202	High altitude hypertension
T70.203	High altitude cerebral edema
T70.204	Mountain sickness
T70.205	Aviation disease
T70.206	Pilot disease (caused by change of flight air pressure)
T70.207	High altitude effect
T70.300	Diver's disease [decompression sickness]
T70.300x002	Diver paralyzed
T70.300x004	Diver paralysis
T70.400	Effect of high pressure liquid
T70.800	Other effects of air pressure and water pressure
T70.800x001	Shock wave injury syndrome
T70.900	Effect of air pressure and water pressure
T71.x00	Extrinsic asphyxia
T71.x00x001	Anoxic asphyxia
T71.x00x002	Traumatic asphyxia
T71.x00x003	Strangulation asphyxia
T71.x00x004	Mechanical asphyxia
T73.000	starvation effect
T73.000x001	Hunger strike
T73.100	Thirst effect
T73.200	Failure caused by exposure to adverse environment
T73.300	Exhaustion caused by overwork
T73.800	Other effects lacking

T73.900	Effect of lack
T74.000	Neglected or abandoned
T74.000x001	Neglected syndrome
T74.000x002	Abandonment syndrome
T74.100	Physical abuse
T74.100x001	Child abuse syndrome
T74.100x002	Child abuse syndrome
T74.100x003	Spouse abuse syndrome
T74.100x004	Somatic Abuse Syndrome
T74.200	sexual abuse
T74.800	Abuse syndrome, other
T74.800x001	Mixed maltreatment syndrome
T74.900	Abuse syndrome
T74.900x001	Adult Abuse Syndrome
T74.900x002	Child abuse syndrome
T75.000	Lightning effect
T75.000x001	Lightning strike
T75.000x002	Lightning shock
T75.100	Drowning and non fatal drowning
T75.100x001	drowning
T75.100x002	Swimmer's cramp
T75.101	Drowning pulmonary edema
T75.200	Vibration effect
T75.200x001	Air hammer syndrome
T75.200x002	Subsonic vertigo
T75.200x003	Traumatic vasospasm syndrome
T75.200x004	Local vibration disease
T75.200x005	Arm vibration disease

T75.300	Motion sickness
T75.300x002	Airsickness
T75.300x003	Seasickness
T75.300x004	Carsickness
T75.400	Current effect
T75.400x001	Electric injury
T75.800	Other specific effects of external factors
T75.800x001	Anomalous gravity effect
T75.800x002	Weightlessness effect
T78.000	Allergic shock caused by harmful food reaction
T78.000x001	Food induced anaphylactic shock
T78.100	Harmful food reactions, others that cannot be classified elsewhere
T78.100x001	Food induced allergic reaction
T78.100x011	food allergy
T78.101	Milk allergy
T78.200	Anaphylactic shock
T78.201	Hexheimer reaction
T78.300	Angioneurotic edema
T78.300x003	Vaso neurogenic reaction of auricle
T78.300x004	Giant urticaria
T78.301	Acute idiopathic edema
T78.400	allergy
T78.400x002	Anaphylactic reaction
T78.800	Harmful effects, others that cannot be classified elsewhere
T78.900	Harmful effect
T79.000	Air embolism (traumatic)
T79.100	Fat embolism (traumatic)

T79.100x002	Traumatic cerebral fat embolism
T79.101	Fat embolism syndrome
T79.200	Secondary and recurrent bleeding from trauma
T79.201	Traumatic recurrent hemorrhage
T79.202	Traumatic secondary bleeding
T79.300	Wound infection after trauma, which cannot be classified elsewhere
T79.300x001	Post traumatic wound infection
T79.400	Traumatic shock
T79.500	Traumatic anuria
T79.500x002	Renal failure after extrusion
T79.501	Crush syndrome
T79.600	Traumatic muscle ischemia
T79.600x003	Lacunar syndrome
T79.600x004	Upper limb osteofascial compartment syndrome
T79.600x006	Lower limb osteofascial compartment syndrome
T79.601	Traumatic osteofascial compartment syndrome
T79.602	Folkman ischemic contracture
T79.603	Abdominal compartment syndrome
T79.700	Traumatic subcutaneous emphysema
T79.800	Other early complications of trauma
T79.800x001	Traumatic lipoliquefaction
T79.800x002	Traumatic necrosis of lower limbs
T79.800x003	Traumatic necrosis of fingers
T79.800x004	Traumatic necrosis of scalp
T79.800x005	Traumatic coagulopathy
T79.800x006	Traumatic hypotony
T79.800x007	Traumatic pneumonia

T79.801	Traumatic meningitis
T79.900	Early complications of trauma
T81.601	Chemical peritonitis
T90.503	Traumatic epilepsy
T92.600x003	Traumatic bone defect of upper limb
T98.200x011	Open injury with foreign body
T98.200x012	Open injury with foreign body and infection
T98.200x021	Open injury with infection
T98.200x031	Delayed healing of open injury
T98.200x032	Delayed treatment of open injury
T98.200x033	Poor healing of open wound
G62.809	Traumatic peripheral neuropathy
G93.200x001	Intracranial hypertension
G93.501	Cerebral hernia
G93.808	Ventricular dilation
G95.106	Spinal cord edema
G95.200	Spinal cord compression
G96.000x006	Traumatic cerebrospinal fluid leakage
G96.001	Cerebrospinal rhinorrhea
G96.002	Cerebrospinal fluid otorrhea
H18.800x009	Corneal epithelial injury
H20.802	Traumatic iridocyclitis
H31.403	Traumatic choroidal detachment
H33.302	Traumatic retinal tear
H33.503	Traumatic retinal detachment
H35.703	Traumatic chorioretinopathy
H40.301	Traumatic glaucoma

H83.301	Acoustic trauma
H91.801	traumatic deafness
I31.800x003	Pericardial pneumatosis
I63.908	Traumatic cerebral infarction
I74.303	Traumatic femoral artery thrombosis
J38.702	Traumatic laryngeal web
J81.x00x001	Traumatic wet lung
K05.500x001	occlusal trauma
K06.200	Injury related gingival and edentulous ridge damage
K12.101	Traumatic oral mucosal ulcer
K14.000x006	Traumatic ulcer of tongue
K20.x00x003	Traumatic esophagitis
K22.207	Traumatic esophageal stricture
K22.301	Esophageal rupture
K85.802	Acute Traumatic Pancreatitis, Mild
K85.815	Acute Traumatic Pancreatitis, Severe
M12.500	Traumatic arthropathy
M25.000	Joint hematocele
M41.501	Traumatic scoliosis
M43.101	Traumatic spondylolisthesis
M48.300	Traumatic spondylosis
M48.304	Traumatic lumbar spondylopathy
M89.820	Bone exposure
R40.200	coma
R57.100	Hypovolemic shock
R57.101	Hemorrhagic shock
